# Co-occurrence patterns of esophageal and stomach cancer across 204 countries and territories: a spatial correspondence and systematic analysis

**DOI:** 10.3389/fonc.2025.1613839

**Published:** 2025-10-17

**Authors:** Jiayao Xu, Jiabei Gong, Huiqiong Han, Zehua Wang, Wenjia Wang, Lei Wang, Xin Sui, Guanyu Chen, Yongxu Jia, Yanru Qin

**Affiliations:** 1 Department of Oncology, The First Affiliated Hospital of Zhengzhou University, Zhengzhou, China; 2 Department of Nephropathy, The First Affiliated Hospital of Zhengzhou University, Zhengzhou, China; 3 Department of Experimental Orofacial Medicine, University of Marburg, Marburg, Germany

**Keywords:** esophageal cancer, stomach cancer, co-occurrence patterns, age-period-cohort model, BAPC model, prediction

## Abstract

**Background:**

Esophageal and gastric cancers are common malignant tumors of the digestive tract worldwide, characterized by a substantial disease burden and significant regional disparities. While these cancers share anatomical proximity, risk factors, and pathogenic mechanisms to some extent, there remains a lack of comprehensive and up-to-date global comparative studies on their co-occurrence patterns and burden trends.

**Methods:**

Using primary data from the Global Burden of Disease (GBD) 2021 study, we defined and categorized global co-occurrence patterns of esophageal and gastric cancers based on quartile methods. Descriptive analysis, correlation analysis, age-period-cohort modeling, decomposition analysis, and predictive modeling were employed to thoroughly examine the disease burden of both cancers across 204 countries and territories from 1990 to 2021.

**Results:**

The disease burden of gastric cancer consistently exceeded that of esophageal cancer in most countries and regions. Spatially, the Eastern and Northern Hemispheres, including countries such as China and Mongolia, were identified as consistent high-burden regions for both cancers. In contrast, the Western and Southern Hemispheres were predominantly characterized by single-cancer dominance or low-burden patterns. Disease burden was negatively correlated with the Socio-demographic Index (SDI), with higher burden observed in low-SDI regions. Males and older populations faced elevated disease risks. Furthermore, population growth and aging were identified as major drivers increasing the overall burden. Predictions indicate that by 2031, the age-standardized rates of both cancers will continue to decline, yet the overall burden of gastric cancer will remain significantly higher than that of esophageal cancer.

**Conclusions:**

Gastric cancer imposes a heavier disease burden than esophageal cancer across most countries and regions. These findings underscore the necessity for sustained and targeted prevention strategies, such as the promotion of healthy lifestyles, enhanced early screening, and improved healthcare accessibility in high-burden regions, to effectively reduce the global burden of esophageal and gastric cancers.

## 1 Introduction

Esophageal cancer (EC) and Stomach cancer (SC) are common malignant tumors of the digestive tract worldwide, posing a serious threat to human health (1–3). According to the latest global cancer statistics GLOBOCAN 2022, EC is the 11th most common cancer and the 7th leading cause of cancer death in the world, while SC is the 5th most common cancer and the 5th leading cause of cancer death in the world (4, 5). Histologically, EC can be classified into two major subtypes: the disease burden of esophageal squamous cell carcinoma (ESCC) significantly exceeds that of esophageal adenocarcinoma (EAC) in most parts of the world (6). The vast majority of gastric cancers are histologically adenocarcinomas. The development of gastric cancer is closely associated with Helicobacter pylori infection (7). A meta-analysis and guidelines have indicated that H. pylori infection shows no association with ESCC, and may even be inversely associated with EAC (8, 9). However, the esophagus and stomach are directly connected anatomically, particularly at the esophagogastric junction, where esophageal adenocarcinoma and gastric cardia cancer exhibit significant overlap in terms of pathogenesis, clinical presentation, and even molecular characteristics. Studies have shown that multiple environmental and behavioral risk factors contribute to the development of both cancers, including smoking, alcohol consumption, high salt intake, consumption of preserved foods, and low fruit and vegetable intake (10). It is worth noting that the burden of esophageal and gastric cancers varies considerably across regions. EC is highly prevalent in economically underdeveloped areas of Asia, Africa, and South America, where dietary habits are often poor. Meanwhile, SC incidence is particularly high in East Asia, Eastern Europe, and South America, regions characterized by lower economic development, unbalanced diets, and high rates of Helicobacter pylori infection (11). Therefore, a combined analysis facilitates a more comprehensive evaluation of their overall burden and its relationship with socioeconomic development.

The GBD study is a comprehensive and systematic analysis of global health trends, offering critical insights into the incidence, prevalence, and mortality rates of various diseases (12, 13). Previous studies have analyzed the disease burden and future trends of SC and EC in China based on GBD 2019 (14), but there are limitations such as insufficient data timeliness, limited to specific regions, lack of discussion on the impact of socio-economic development level, and no analysis of age-period-cohort factors and the impact of aging, epidemiological changes and population growth. Although numerous GBD-based studies on individual cancers exist (15–19), this is the first systematic comparative analysis of EC and SC, which are highly related in public health strategies, using GBD 2021 data. The study not only delineates the burden of each cancer but also emphasizes the similarities and differences in their spatial distribution of co-occurrence patterns, gender disparities, age distribution, and temporal trends. Furthermore, this research projects the disease burden of cancers over the next decade, which is critical for evaluating the impact of public health interventions and guiding strategic directions for future cancer control (20, 21).

## 2 Materials and methods

### 2.1 Data Sources and Extraction

The GBD 2021 is the most extensive and comprehensive epidemiological assessment of global disease burdens and trends to date. Disease and study population data were retrieved from the Global Burden of Disease 2021 database (https://ghdx.healthdata.org/gbd-2021). Esophageal cancer and Stomach cancer are defined using 150 and 151 in the International Classification of Diseases, ninth edition (ICD-9) code, and C15 and C16 in the tenth edition (ICD-10) revision code (2). To facilitate meaningful comparisons across different populations, age standardization was performed using the world standard population developed by Segi and modified by Doll et al. (22, 23). All estimates are presented as age-standardized rates (ASR). The SDI, which ranges from 0 to 1, was incorporated to examine the relationship between trends in esophageal and gastric cancers and socioeconomic development. Countries were also grouped into five SDI-based quintiles (following the classification scheme established by the Institute for Health Metrics and Evaluation). The SDI serves as a composite measure of national development, integrating total fertility rate, per capita income, and average educational attainment.

In this study, we obtained and analyzed GBD 2021 data on gastric and esophageal cancers incidence, mortality and disability-adjusted life years (DALYs) by sex (male and female), age (20 age groups from<5 years to >95 years, at 5-year intervals), SDI quintiles, 204 countries and territories.

### 2.2 Analysis and statistical methods

#### 2.2.1 Definition and regional division of co-occurrence patterns of esophageal cancer and stomach cancer

To investigate the co-occurrence patterns of EC and SC, as well as their spatial variations across different regions, incidence, mortality, and DALYs for both cancers in 2021 were classified into four tiers according to quartile ranges: low (<25 percentile), lower-middle (25–50 percentiles), upper-middle (50–75 percentiles), and high (>75 percentile). Countries or territories where both cancers exhibited the same incidence level were categorized as consistent units; collectively, these constituted the consistent region. Those with a higher incidence level of EC relative to SC were identified as esophageal cancer-dominant units, forming the esophageal cancer-dominant region. Correspondingly, areas where stomach cancer incidence surpassed that of esophageal cancer were classified as stomach cancer-dominant units, aggregating into the stomach cancer-dominant region. Thus, the global study area was segmented into three distinct regions, each reflecting a distinct pattern of co-occurrence between EC and SC. Furthermore, Spearman rank correlation analysis was employed to quantify the bivariate association patterns. ASR were integrated and processed by “time-region type-metric type” with handling of missing values. The ranks of the two cancer burden metrics were then transformed, and stratum-specific ρ values were calculated for each region type between 1990 and 2021. A significance level of α = 0.05 was set, with P< 0.05 considered statistically significant. This analysis helped characterize the temporal evolution of co-occurrence patterns and supported regional clustering analysis. All analyses were conducted using R version 4.3.3.

#### 2.2.2 Descriptive statistics of disease burden

To account for potential age structure differences, ASR with corresponding 95% uncertainty intervals (UI) and the average annual percent change (AAPC) were employed to assess the burden of cancer by quantifying incidence, mortality and DALYs trends. The 95% confidence intervals (CI) for AAPC were computed on the basis of the standard errors of the regression slopes under the assumption of normally distributed residuals. These intervals reflect the degree of statistical uncertainty around the estimated rate of change in disease burden. If the CI of an AAPC estimate excludes zero, the trend is considered statistically significant. Conversely, intervals overlapping zero indicate a stable trend with no significant change.


$$ASR=\frac{\sum_{i=1}^{A}\alpha_{i}w_{i}}{\sum_{i=1}^{A}w_{i}}\times 100.00$$

(αi: age-specific ratio for age group i; w: population count for corresponding age group i in the standard population; A: total number of age groups).

The UI refers to the range of uncertainty in prediction results caused by various uncertain factors during the forecasting process. This uncertainty interval helps us better understand the possible range of predicted outcomes and supports more informed decision-making. In GBD 2021, the UI is calculated through 1,000 iterations of Monte Carlo simulations, reflecting the combined effects of data input, model parameters, and sampling variation. Given the inherent statistical modeling and computational uncertainties in GBD studies, all estimates reported in this study are presented as the median value along with the 95% UI.

#### 2.2.3 Time trend analysis

This study employed Joinpoint regression analysis (24). This approach calculates two key metrics: (1) the average annual percentage change (AAPC), which reflects the overall mean trend across the entire study period, (2) the annual percentage change (APC), describing trends within individual segments. In this study, we initially employed a logarithmic linear model for segmented regression, applied the grid search method to identify all potential joinpoints, calculated the mean squared error (MSE) for each scenario, and selected the grid point with the smallest MSE as the joinpoint. Subsequently, the Monte Carlo permutation test was used to identify turning points in trends, with the maximum number of potential joinpoints set to five. Ultimately, we identified the key years marking turning points in the temporal trends of incidence and mortality rates for EC and SC globally from 1990 to 2021.

#### 2.2.4 Age-period-cohort analysis

An age-period-cohort (APC) model was used to systematically analyze the various factors contributing to changes in disease burden, including age effects, period effects, and birth cohort effects. Age effects reflect the natural variation in disease risk over the life course; period effects capture the short-term impact of external factors (such as medical advancements or policy interventions) on disease burden; and birth cohort effects reveal long-term disease burden differences due to exposure to specific risk factors within different cohorts. The model was implemented in R (version 4.3.3), using orthogonal decomposition to separate linear and non-linear components, with parameter estimation performed using weighted least squares (WLS). The model fit was assessed using the Wald χ² test (25).

#### 2.2.5 Decomposition analysis of disease burden

To quantify the contributions of population aging, population growth, and epidemiological changes to esophageal and gastric cancers burden, a demographic decomposition method was employed. Specifically, the changes in disease burden were decomposed into three main factors: changes in age structure, population size, and epidemiological changes. The relative contributions of these factors to the overall disease burden change were then analyzed to identify the primary drivers of the disease burden shift (26, 27).

#### 2.2.6 Bayesian age-period-cohort analysis

To predict the trend of esophageal and gastric cancers burden from 2022 to 2031, this study employed the Bayesian age-period-cohort (BAPC) model. This model uses a second-order random walk prior to smooth age, period, and cohort effects, effectively avoiding overfitting. Using the Integrated Nested Laplace Approximation (INLA) method, the model efficiently computed the marginal posterior distribution, circumventing the computational bottleneck associated with traditional Markov Chain Monte Carlo (MCMC) methods. To ensure the reliability of the predictions, cross-validation and other methods were used to assess the robustness of the model (7).

#### 2.2.7 Correlation analysis

Spearman rank correlation analysis was used to examine the correlation between SDI and ASR. To control for potential false-positive results due to multiple comparisons, the Benjamini- Hochberg method was applied to adjust the false discovery rate (FDR< 0.05). Additionally, locally weighted scatterplot smoothing (LOWESS) was used to fit non-linear trends and further reveal the complex relationships between SDI and disease burden.

#### 2.2.8 Statistical methods

All data analyses were performed in Software R (version 4.3.3) and R studio, and the BAPC predictive model used the “nordpred (version 1.1)”, “BAPC (version 0.0.36)” and “INLA (version 22.05.07)” packages. P< 0.05 was considered statistically significant.

## 3 Results

### 3.1 Spatial distribution under co-occurrence patterns

**Figure 1:**
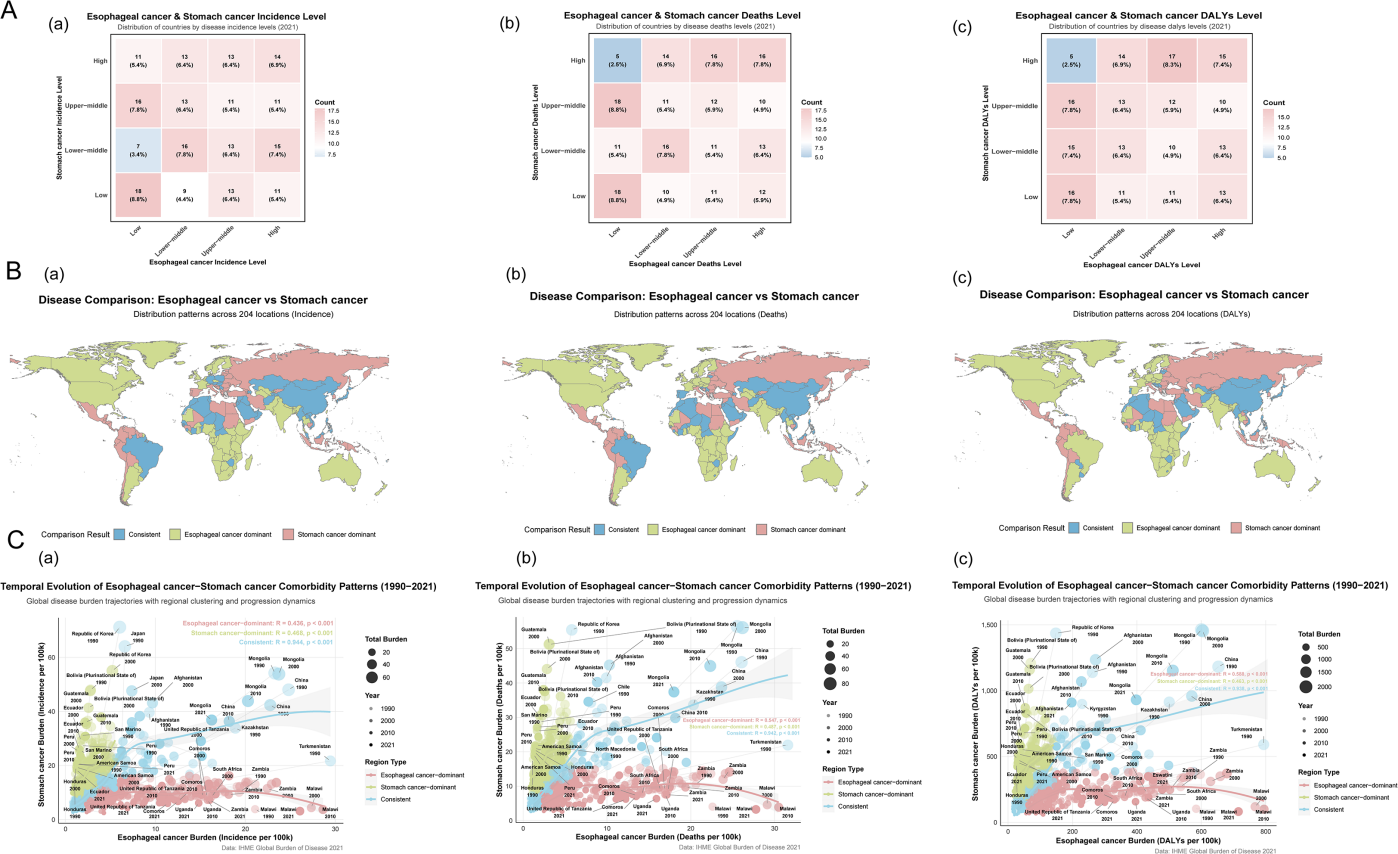
Characteristics of co-occurrence in the ASR of global esophageal and stomach cancer.The country or territory was classified as a consistent unit if the incidence rate levels of both diseases were identical. **(A)** a. Esophageal cancer & Stomach cancer Incidence Level; b. Esophageal cancer & Stomach cancer Deaths Level; c. Esophageal cancer & Stomach cancer DALYs Level. **(B)** a. Distribution patterns across 204 locations (Incidence) of Esophageal and Stomach cancer; b. Distribution patterns across 204 locations (Deaths) of Esophageal and Stomach cancer; c. Distribution patterns across 204 locations (DALYs) of Esophageal and Stomach cancer. **(C)** a. Temporal Evolution in the Incidence of Esophageal and Stomach cancer Comorbidity Patterns; b. Temporal Evolution in the Deaths of Esophageal and Stomach cancer Comorbidity Patterns; c. Temporal Evolution in the DALYs of Esophageal and Stomach cancer Comorbidity Patterns.

This study encompassed data on the incidence, mortality, and disability-adjusted life years (DALYs) of EC and SC across 204 countries and territories worldwide. As of 2021, the global age-standardized incidence rate (ASIR), age-standardized mortality rate (ASMR), and age-standardized DALYs rate (ASDR) of SC remained significantly higher than those of EC (**Table 1**). At the quartile level, the spatial distributions of two cancers showed substantial overlap (**Figure 1B**). Countries with three ASR classified as “consistent” accounted for 28.92%, 30.39%, and 27.45% of the global total respectively (**Figure 1A**; **Supplementary Tables 4–6**). The Eastern and Northern Hemispheres emerged as the regions with the highest concentration of this “consistent” pattern, particularly in Asian nations such as Afghanistan, China, North Korea, and Mongolia, which represent core areas of the highest disease burden globally (**Supplementary Figures 1, 2**, **Supplementary Table 2–3**). Notably, South Korea was identified as a region with the higher morbidity and mortality under the co-occurrence pattern (**Supplementary Figures 1, 2**), yet it demonstrated a remarkably significant decline in disease burden (DALYs: AAPC for EC: −3.502; SC: −5.059). In contrast, the Western and Southern Hemispheres were predominantly characterized by low-burden profiles or patterns dominated by one cancer type. However, several countries, primarily located in North Africa (such as Algeria, Morocco, and Tunisia) and the Middle East (including Iraq, Israel, Jordan, Kuwait, Lebanon, Oman, and Saudi Arabia), also exhibited the “consistent” pattern. Benefiting from relatively higher income levels, often associated with oil resources, along with continuously developing healthcare systems, these nations maintained their ASR at comparatively low levels (**Figure 1B**, **Supplementary Tables 4–6**). Spanning both hemispheres, Brazil stood out as the most prominent representative of the “consistent” pattern, accompanied by a considerable disease burden (**Supplementary Figures 1, 2**).

**Table 1:** ASR of esophageal cancer and stomach cancer at 5 SDI level between 1990 and 2021.

Cause	Location	Incidence			Mortality			DALYs		
		ASIR per 100,000, 1990 (95%UI)	ASIR per 100,000, 2021 (95%UI)	AAPC (95%CI) 1990-2021	ASMR per 100,000, 1990 (95%UI)	ASMR per 100,000, 2021 (95%UI)	AAPC (95%CI) 1990-2021	ASDR per 100,000, 1990 (95%UI)	ASDR per 100,000, 2021 (95%UI)	AAPC (95%CI) 1990-2021
Esophageal Cancer	Global	8.857 (7.962,9.694)	6.655 (5.883,7.45)	-0.940 (-0.982,-0.906)	9.022 (8.112,9.866)	6.255 (5.527,7.003)	-1.197 (-1.242,-1.162)	235.319 (210.517,258.681)	148.561 (131.709,166.818)	-1.500 (-1.543,-1.466)
	High SDI	5.358 (5.138,5.492)	4.943 (4.631,5.156)	-0.271 (-0.298,-0.253)	4.934 (4.732,5.062)	4.02 (3.746,4.205)	-0.670 (-0.714,-0.640)	123.988 (120.471,126.95)	93.953 (89.284,97.917)	-0.884 (-0.937,-0.836)
	High-middle SDI	11.17 (9.849,12.491)	8.841 (7.258,10.703)	-0.754 (-0.812,-0.697)	11.519 (10.191,12.867)	8.128 (6.719,9.766)	-1.157 (-1.240,-1.090)	303.104 (265.931,341.095)	192.562 (158.696,234.03)	-1.505 (-1.590,-1.438)
	Low SDI	6.692 (5.583,7.514)	5.494 (4.705,6.316)	-0.624 (-0.634,-0.614)	7.149 (5.968,8.013)	5.89 (5.023,6.797)	-0.610 (-0.621,-0.599)	185.383 (154.417,209.222)	148.672 (126.111,172.196)	-0.698 (-0.708,-0.687)
	Low-middle SDI	4.095 (3.681,4.698)	3.592 (3.241,4.151)	-0.411 (-0.452,-0.372)	4.36 (3.924,5.017)	3.793 (3.418,4.389)	-0.436 (-0.474,-0.400)	113.845 (102.949,130.891)	97.097 (87.736,111.836)	-0.500 (-0.531,-0.477)
	Middle SDI	13.679 (11.493,15.775)	8.103 (6.782,9.624)	-1.678 (-1.707,-1.644)	14.31 (12.195,16.447)	7.913 (6.65,9.337)	-1.899 (-1.931,-1.866)	365.58 (309.417,422.251)	180.646 (153.165,214.627)	-2.260 (-2.288,-2.228)
Stomach Cancer	Global	24.763 (22.58,27.002)	14.328 (12.226,16.408)	-1.763 (-1.812,-1.728)	22.006 (20.028,24.187)	11.199 (9.618,12.734)	-2.173 (-2.194,-2.151)	559.721 (499.087,615.772)	262.748 (226.079,301.024)	-2.424 (-2.452,-2.399)
	High SDI	23.133 (22.047,23.8)	11.155 (10.207,11.905)	-2.337 (-2.402,-2.310)	15.863 (15.01,16.371)	6.834 (6.18,7.336)	-2.690 (-2.762,-2.656)	381.131 (362.206,392.227)	146.104 (135.56,155.885)	-3.074 (-3.123,-3.038)
	High-middle SDI	33.327 (30.086,36.102)	19.62 (16.016,23.134)	-1.694 (-1.744,-1.638)	31.084 (28.093,33.705)	14.929 (12.397,17.364)	-2.348 (-2.395,-2.302)	802.75 (711.792,876.759)	353.179 (291.888,416.778)	-2.623 (-2.672,-2.578)
	Low SDI	11.408 (9.041,13.026)	8.132 (6.435,9.222)	-1.076 (-1.102,-1.051)	11.898 (9.41,13.573)	8.462 (6.71,9.601)	-1.084 (-1.115,-1.054)	311.981 (248.616,357.312)	209.77 (165.604,238.952)	-1.264 (-1.294,-1.239)
	Low-middle SDI	10.139 (8.979,12.416)	7.684 (6.715,8.771)	-0.896 (-0.929,-0.850)	10.448 (9.246,12.789)	7.709 (6.693,8.76)	-0.982 (-1.022,-0.934)	274.452 (239.886,333.491)	192.559 (169.425,219.14)	-1.132 (-1.159,-1.100)
	Middle SDI	28.887 (25.129,33.443)	16.913 (13.785,20.276)	-1.734 (-1.763,-1.704)	28.098 (24.614,32.65)	13.716 (11.309,16.218)	-2.318 (-2.349,-2.290)	721.809 (624.062,835.383)	320.242 (266.074,382.872)	-2.623 (-2.652,-2.596)

Regions with a high incidence of EC, categorized as EC-dominant regions (73 [35.78%] of 204), were predominantly located in Eastern and Southern Africa (e.g., Malawi: 26.064/100,000; Lesotho: 15.821/100,000; Somalia: 14.906/100,000) and North America (e.g., Greenland: 10.808/100,000). In contrast, regions with a high incidence of SC, classified as SC-dominant regions (72 [35.29%] of 204), were primarily found across the Americas (e.g., Bolivia: 30.813/100,000; Guatemala: 23.687/100,000), Oceania (e.g., Nauru: 21.091/100,000; Palau: 19.651/100,000), Eastern Europe (e.g., Russia: 15.693/100,000), and East Asia (e.g., Republic of Korea: 25.762/100,000). DALYs serve as a gold-standard metric for quantifying disease burden. High EC burden was observed in EC-dominant regions (80 [39.22%] of 204), concentrated mainly in Sub-Saharan Africa (e.g., Malawi: 715.282/100,000; Eswatini: 478.852/100,000; Zambia: 436.301/100,000), South America (e.g., Brazil: 132.78/100,000), and North America (e.g., Greenland: 272.77/100,000). Conversely, high SC burden in SC-dominant regions (68 [33.33%] of 204) was notably distributed throughout Oceania (e.g., Nauru: 557.026/100,000; Solomon Islands: 516.88/100,000), the Americas (e.g., Bolivia: 714.431/100,000; Peru: 455.295/100,000), and Eastern Europe (e.g., Belarus: 324.567/100,000; Russia: 368.392/100,000). Furthermore, a polarized distribution of disease burden was observed (**Supplementary Table 6**). Five countries (Botswana, Malawi, Netherlands, Pakistan, United Kingdom) exhibited a high EC burden coupled with a low SC burden. Among these, Malawi showed the most pronounced disparity in DALYs (EC: 715.28/100,000 vs. SC: 81.80/100,000). Conversely, 13 countries (including Guatemala, Honduras, Peru, Ecuador, American Samoa, El Salvador, among others) demonstrated a high SC burden alongside a low EC burden. Guatemala displayed the greatest difference in DALYs (EC: 40.657/100,000 vs. SC: 581.430/100,000).

### 3.2 Temporal trends in co-occurrence patterns

Based on the bivariate temporal and regional correlation patterns of disease burden (**Figure 1C**), the blue regions indicate a strong positive correlation between the two diseases, which facilitates the visualization and analysis of comorbidity-related burden. Among these areas, countries with the strongest correlated trends in incidence, including Japan, China, Mongolia, and Kazakhstan, showed a gradual decline in incidence over time. Similarly, the nations with the highest correlation in mortality and DALYs, namely China, Mongolia, and Kazakhstan, also exhibited a decreasing trend in these measures throughout the study period.

### 3.3 Global burden and trends of esophageal and gastric cancers

Compared to 1990 (**Table 1**, **Supplementary Table 1**), ASR for SC showed a more pronounced decline. The AAPC for SC (ASIR: −1.763, ASMR: −2.173, ASDR: −2.424) were all greater than those for EC (AAPC: ASIR: −0.940, ASMR: −1.197, ASDR: −1.500). Based on these estimates, the global number of new cases in 2021 was 576,529 (95% UI: 509,492, 645,648) for EC and 1,230,233 (95% UI: 1,052,350, 1,409,970) for SC. The recorded deaths were 538,602 (95% UI: 475,944, 603,406) and 954,374 (95% UI: 821,751, 1,089,577) for EC and SC, respectively. The total DALYs lost were 12,999,265 (95% UI: 11,522,861, 14,605,268) for EC and 22,786,633 (95% UI: 19,576,344, 26,118,869) for SC. Although SC exhibited a more marked decline in ASR, its overall global disease burden remains substantially heavier than that of EC.

### 3.4 Regional burden and trends of esophageal and gastric cancers

As of 2021, the ASIR for both EC and SC were observed in High-middle SDI regions, with values of 8.841 per 100,000 (95% UI: 7.258, 10.703) and 19.62 per 100,000 (95% UI: 16.016, 23.134), respectively. In contrast, the lowest ASIR was found in Low-middle SDI regions, at 3.592 per 100,000 (95% UI: 3.241, 4.151) for EC and 7.684 per 100,000 (95% UI: 6.715, 8.771) for SC. Similarly, the highest ASMR for both cancers were also recorded in High-middle SDI regions: 8.128 per 100,000 (95% UI: 6.719, 9.766) for EC and 14.929 per 100,000 (95% UI: 12.397, 17.364) for SC. The lowest ASMR was observed for EC in Low-middle SDI regions, at 3.793 per 100,000 (95% UI: 3.418, 4.389), and for SC in High SDI regions, at 6.834 per 100,000 (95% UI: 6.18, 7.336). Regarding ASDR, both cancers exhibited the highest burden in High SDI regions, with values of 192.562 per 100,000 (95% UI: 158.696, 234.030) for EC and 353.179 per 100,000 (95% UI: 291.888, 416.778) for SC. The lowest ASDR was observed in Low SDI regions, at 93.953 per 100,000 (95% UI: 89.284, 97.917) and 146.104 per 100,000 (95% UI: 135.56, 155.885) for esophageal and gastric cancer, respectively. Although ASR for both cancers showed a declining trend, with a more pronounced decrease observed for SC, the disease burden of SC remained higher than that of EC across all five SDI regions (**Table 1**).

### 3.5 National burden and trends of esophageal and gastric cancers

As of 2021, Asia represented the global epicenter of disease burden for both gastric and esophageal cancers. Mongolia exhibited the most severe comorbid burden, with an ASDR of 397.978 per 100,000 (95% UI: 317.659, 481.220) for EC and 930.449 per 100,000 (95% UI: 747.523, 1157.922) for SC. In contrast, Kuwait demonstrated the lightest comorbid burden, with an ASDR as low as 19.704 per 100,000 (95% UI: 15.891, 24.306) for EC and 54.865 per 100,000 (95% UI: 44.292, 68.485) for SC. Globally, the vast majority of countries exhibited a declining trend in ASR, with SC demonstrating a more pronounced reduction than EC (172 [84.31%] of 204). The most notable declines in ASDR for EC and SC were observed in Kazakhstan (AAPC: −4.422, 95% CI: −4.615, −4.253) and the Maldives (AAPC: −4.499, 95% CI: −4.598, −4.383), respectively. Exceptions to this trend were also identified. Chad and Egypt showed the most significant increases in ASDR for EC (AAPC: 2.56, 95% CI: 2.342, 2.784) and SC (AAPC: 1.274, 95% CI: 1.125, 1.421), respectively. It is important to emphasize that the disease burden of SC remained higher than that of EC in the majority of countries (147 [72.06%] of 204).

### 3.6 Age-sex-time trends in esophageal and gastric cancers

**Figure 2:**
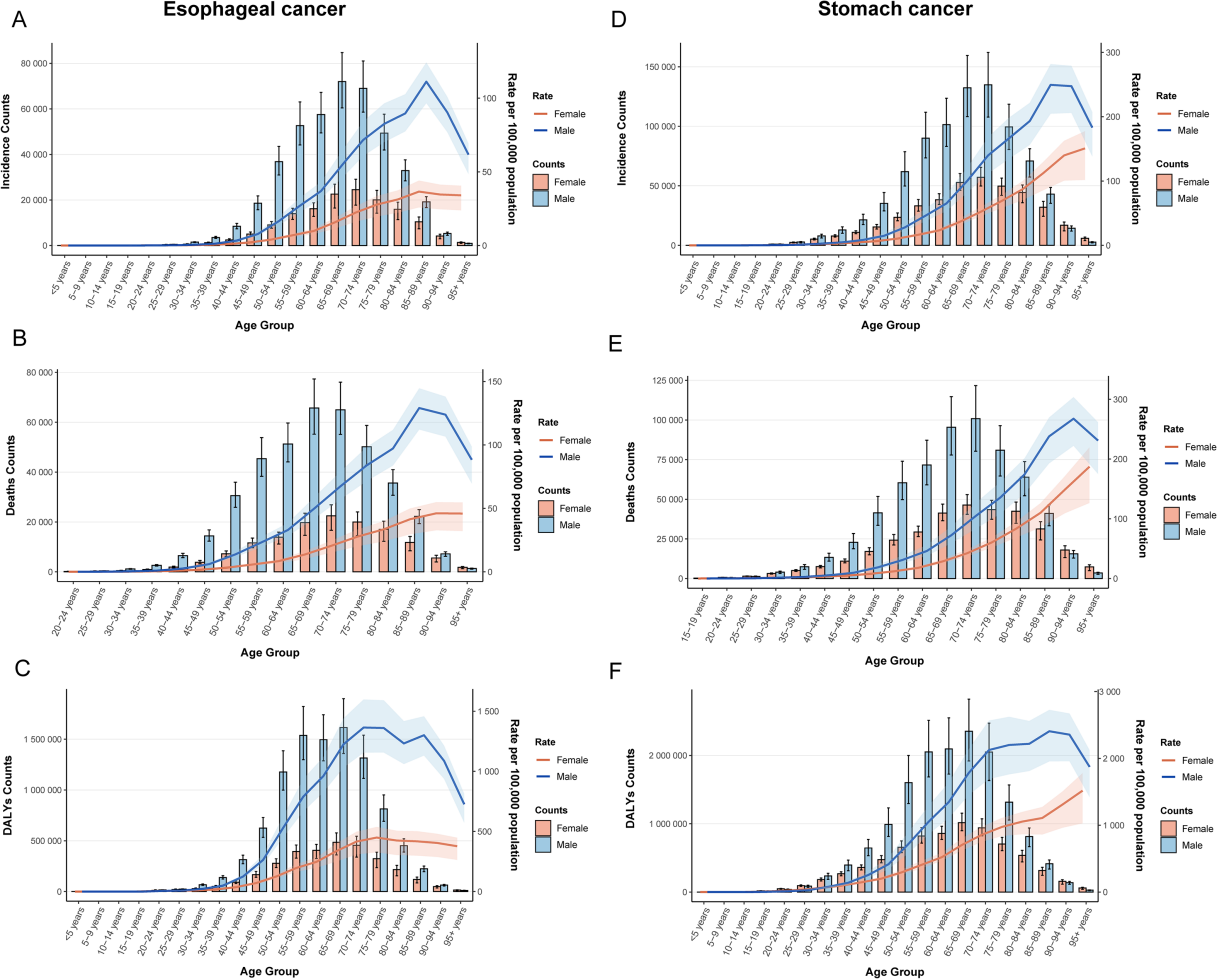
Age-sex dual-axis coordinates of ASR. **(A)** ASIR of esophageal cancer; **(B)** ASMR of esophageal cancer; **(C)** ASDR of esophageal cancer; **(D)** ASIR of stomach cancer; **(E)** ASMR of stomach cancer; **(F)** ASDR of stomach cancer.

Sex and age**-**stratified data revealed that the ASR and case numbers of both cancers increased with age before subsequently declining, with males consistently exhibiting higher rates than females (**Figure 2**). After adjusting for period and birth cohort effects, age-effect analysis reaffirmed this declining trend (**Supplementary Figures 3, 4: a, d, g**). Birth cohort analysis indicated that later birth cohorts were associated with lower ASR levels, suggesting a reduction in cumulative risk among more recently born populations (**Supplementary Figures 3, 4: c, f, i**).

**Figure 3:**
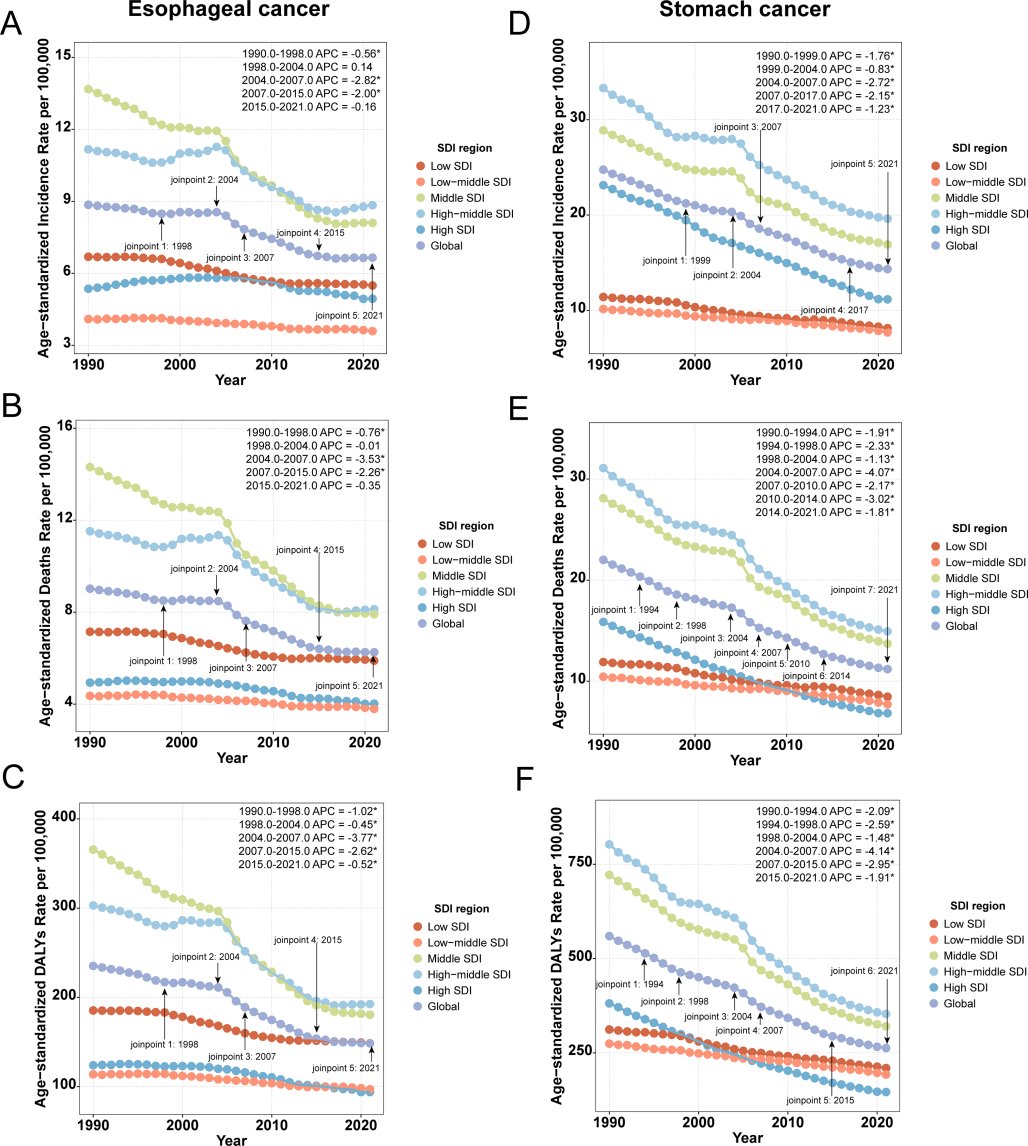
Joinpoint regression analysis of ASR. **(A)** ASIR of esophageal cancer; **(B)** ASMR of esophageal cancer; **(C)** ASDR of esophageal cancer; **(D)** ASIR of stomach cancer; **(E)** ASMR of stomach cancer; **(F)** ASDR of stomach cancer.

Period-effect analysis demonstrated a consistent downward trend in ASR across all time points (**Supplementary Figures 3, 4: b, e, h**), a finding further confirmed by Joinpoint regression. Between 1990 and 2021, significant declines in ASR were observed globally and across all five SDI regions. Notably, APC in global ASR for both cancers reached its lowest values during the period 2004–2007, indicating the most pronounced rate of decline (**Figure 3**).

### 3.7 The association between ASR of esophageal and gastric cancers and the SDI

**Figure 4:**
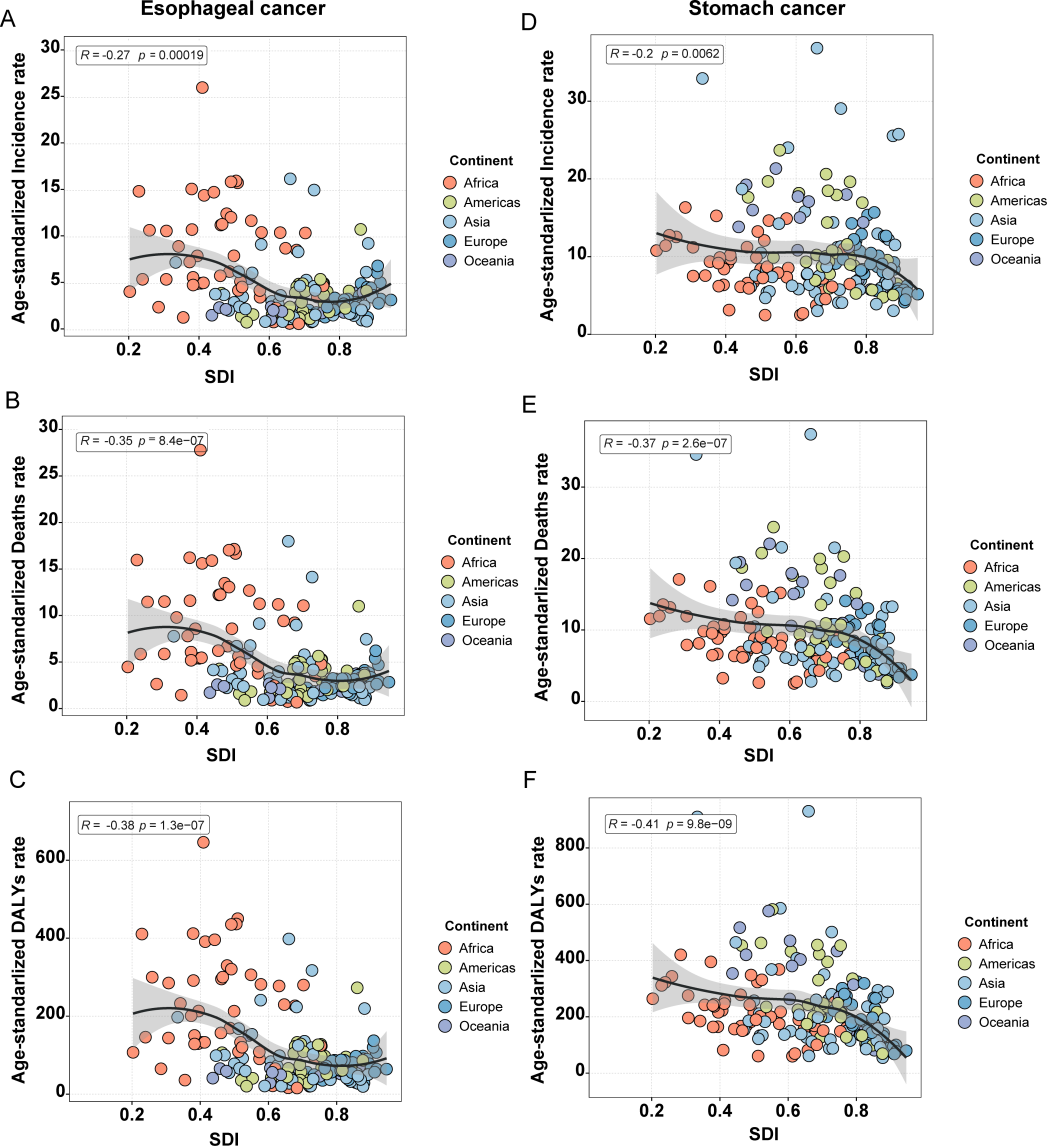
Correlation between ASR and the five continents for esophageal and stomach cancer. **(A)** ASIR of esophageal cancer; **(B)** ASMR of esophageal cancer; **(C)** ASDR of esophageal cancer; **(D)** ASIR of stomach cancer; **(E)** ASMR of stomach cancer; **(F)** ASDR of stomach cancer.

A general downward trend in ASR was observed with increasing SDI, although considerable regional disparities remain. For example, the burden of both cancers was significantly more severe in low SDI regions, particularly in Africa. As SDI levels rose, ASR gradually decreased across the other four continents; however, occasional increases were still observed in certain countries within these regions (**Figure 4**). As mentioned previously, each of these continents contains recognized high-risk countries for the respective cancers, such as China, Greenland, Brazil, Nauru, and Bolivia, which provides a plausible explanation for this phenomenon.

### 3.8 Decomposition analysis and future projections

**Figure 5:**
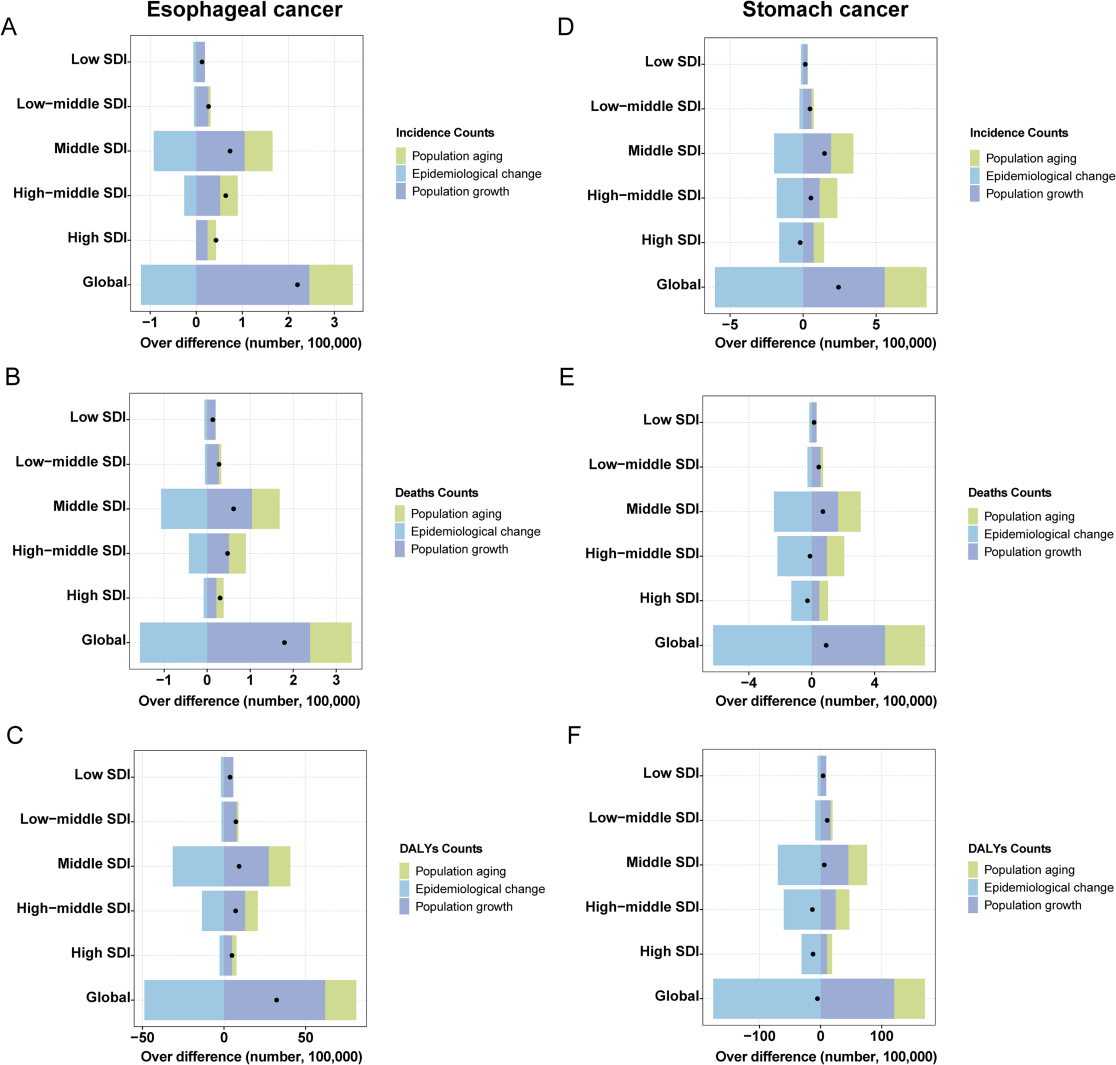
Decomposition analysis esophageal and stomach cancer. **(A)** Incidence Counts of esophageal cancer; **(B)** Deaths Counts of esophageal cancer; **(C)** DALYs Counts of esophageal cancer; **(D)** Incidence Counts of stomach cancer; **(E)** Deaths Counts of stomach cancer; **(F)** DALYs Counts of stomach cancer.

**Figure 6:**
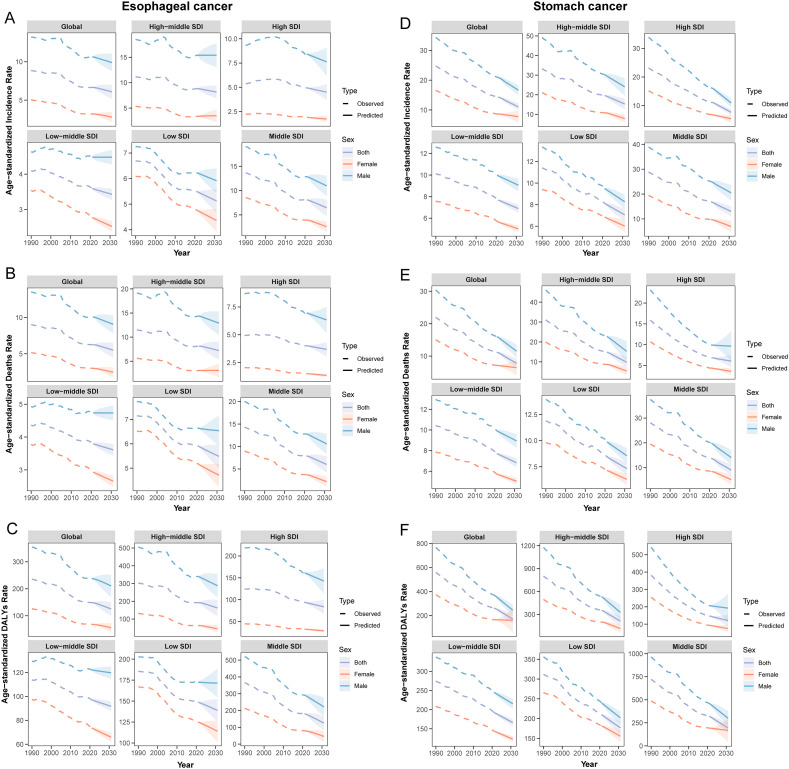
ASR predicted by BAPC for global Esophageal and Stomach cancer from 1990 to 2031. **(A)** ASIR of esophageal cancer; **(B)** ASMR of esophageal cancer; **(C)** ASDR of esophageal cancer; **(D)** ASIR of stomach cancer; **(E)** ASMR of stomach cancer; **(F)** ASDR of stomach cancer.

It is evident that in most regions, population growth and aging have contributed to an increased disease burden of both cancers, while epidemiological changes have reduced the burden. Notably, aging and population growth were the most significant drivers of increased burden (**Figure 5**). It is important to emphasize that, at the global level, although the data point for SC shows a leftward shift in DALYs counts, this only indicates a reduction in DALYs counts between 1990 and 2021. Thus, the trend reflected by this shift remains consistent with our earlier data (**Table 1**), and the overall burden of SC continues to exceed that of EC. By 2031, the ASR for both EC and SC are projected to decline significantly (**Figure 6**). Globally, there will be an estimated 659,133 (95% UI: 605,617, 712,647) new cases of EC, resulting in 606,469 (95% UI: 550,799, 662,138) deaths and 14,302,920 (95% UI: 12,857,279, 15,748,560) years of life lost. For SC, an estimated 1,314,282 (95% UI: 1,251,648, 1,376,914) new cases are projected, along with 988,626 (95% UI: 923,725, 1,053,526) deaths and 22,823,342 (95% UI: 21,253,631, 24,393,053) years of life lost (**Supplementary Tables 7, 8**). These findings indicate that the burden of SC will continue to substantially exceed that of EC in the foreseeable future.

## 4 Discussion

Previous studies have indicated that these two cancers share similar epidemiological features (28, 29). Our results demonstrate that the Eastern and Northern Hemispheres represent the regions with the highest concentration of co-occurrence patterns, while the Western and Southern Hemispheres are predominantly characterized by low-burden profiles or patterns dominated by one cancer type. These findings are largely consistent with earlier epidemiological studies focusing on either esophageal or gastric cancer individually, though some discrepancies remain. Most countries are influenced by a combination of multiple factors, and the specific composition and temporal variation of these factors significantly contribute to the global disparities and heterogeneity in the distribution and burden of both cancers. The intertwined effects of these risk factors underscore the importance of multifaceted intervention strategies.

First, cultural habits and medical level. Mongolia’s high co-occurrence burden is driven by a diet heavy in salty foods, frequent consumption of scalding-hot milk tea, limited intake of fresh fruits and vegetables, high prevalence of Helicobacter pylori infection, and widespread tobacco and alcohol use (30). In contrast, Kuwait, an affluent Gulf nation, exhibits the lowest co-occurrence burden, which can be attributed to its diverse and healthy diet, advanced healthcare system, and cultural norms influenced by Islam that prohibit alcohol consumption. Moreover, substantial disparities in the burden of the two cancers can exist within the same country. Malawi, one of Africa’s major tobacco-producing countries, has widespread availability of unfiltered, self-rolled tobacco products. Dietary factors including consumption of high-temperature foods, mycotoxin-contaminated staples, smoked products, and selenium-deficient diets, along with frequent exposure to smoke from wood-burning stoves, contribute to its high incidence of EC (1). Although H. pylori infection rates are high across Africa (31), the associated cancer risk remains relatively low. This phenomenon, often termed the “African enigma,” has been partly attributed to the predominant circulation of the hpAfrica2 strain, which confers lower carcinogenic potential (32, 33). Thus, Malawi is characterized by a high burden of EC but a low burden of SC, a pattern which has been corroborated by data from the Malawi National Cancer Registry (34). Conversely, Guatemala exhibits a high burden of SC alongside a low burden of EC. Latin America as a whole is not a high-risk region for EC, which may be partly explained by shared dietary and environmental conditions that reduce esophageal cancer risk. However, factors such as H. pylori infection, environmental exposures, and genetic susceptibility contribute to elevated SC risk (35), a pattern supported by findings from an ecological study (36). Therefore, adopting healthy lifestyle practices, such as maintaining a balanced diet, ceasing tobacco use, limiting alcohol consumption, and engaging in regular physical activity, can effectively reduce the risk of both cancers.

Second, disease screening and level of public participation. The rapid development of early diagnosis and treatment programs, particularly in the field of upper gastrointestinal cancers, has significantly improved the detection and management of early-stage gastric and esophageal cancers. For instance, national cancer screening programs in Japan and South Korea have achieved early gastric cancer detection rates of 70% and 50%, respectively (37, 38). Although South Korea is affected by common risk factors and represents the higher level in terms of both incidence and mortality under the co-occurrence pattern, it has experienced a remarkable decline in disease burden. This can be largely attributed to the National Cancer Screening Program (NCSP) implemented since 1999, which has facilitated the identification of a substantial number of early-stage cases. Coupled with advanced medical technology and treatment expertise, this initiative has significantly reduced mortality rates (39). Similarly, Japan initiated nationwide screening two decades earlier than South Korea. However, the coverage of endoscopic screening in Japan has been considerably lower. This is partly because Japanese screening guidelines did not recommend endoscopic screening until 2014. By 2015, only 19% of municipalities in Japan employed endoscopic screening, whereas as early as 2011, already 72.55% of participants in South Korea had opted for this method (40, 41). In addition, public participation in screening has been lower in Japan compared to South Korea (42), contributing to a higher burden of EC in Japan. Nevertheless, Japan has been proactive in combating Helicobacter pylori infection. In 2013, Japan became the first country to provide national health insurance coverage for the eradication therapy of H. pylori-associated gastritis (43). This policy is one of the key factors explaining why Japan maintains a lower burden of gastric cancer than South Korea, despite its delayed policy adoption and lower screening participation.

Third, economic development level. Globally, our findings indicate that among double high and upper-middle countries, a considerable number of high-burden regions are located in low or lower-middle-income African nations (25 [46.30%] of 54). In contrast, among double low and lower-middle countries, a significant proportion of low-burden regions are situated in high-income European and Middle Eastern countries (20 [36.36%] of 55). Furthermore, countries most affected by EC and SC exhibit distinct economic characteristics. Nations within EC-dominated or SC-dominated regions are predominantly low or lower-middle-income countries. This pattern of comorbidity across different economic strata underscores that socioeconomic development is a fundamental and primary factor in disease prevention and control. In high-income countries such as the United States, Canada, Singapore, and several developed nations in Europe, enhanced screening efforts have contributed to an approximate 5% improvement in the five-year survival rate for SC (44). Compared to high-incidence regions such as East Asia and high-income Asia-Pacific, regions like Australia and high-income North America demonstrate notable advantages in public health awareness and education. Increased public knowledge regarding the association between Hp infection and SC has facilitated the adoption of preventive measures, effectively reducing SC incidence. Studies indicate that the Hp infection rate in the United States is approximately 36%, significantly lower than the global average of 50% (45, 46). Therefore, strengthening risk factor control and expanding screening coverage are essential strategies for cancer prevention. Although our results suggest a relatively weak correlation between the burden of EC and SC and the SDI, a declining trend in ASIR, ASMR, and ASDR was observed with increasing SDI. Given the economic constraints in low-income regions that hinder large-scale screening, we recommend adopting a high-risk population screening model coupled with low-cost screening technologies. This approach would improve early diagnosis rates while reducing overall screening costs.

From a temporal trend perspective, the global ASR for both cancers have shown a consistent decline, with the most pronounced reduction observed during the period 2004–2007. This trend may be attributed to the following factors: In 1994, the National Institutes of Health (NIH) convened the first global consensus conference on Hp. Based on robust evidence, the Maastricht I Consensus was established in 1997 and subsequently refined, leading to the widespread adoption of eradication therapy in the early 2000s. Additionally, the process of urbanization accelerated globally during the 2000s. Developed regions such as Europe and the United States reached urbanization rates of nearly 80%, entering a mature stage of urban development, while developing countries including China and India underwent rapid urbanization. This period saw increased public awareness of healthy diets and the risks associated with smoking and alcohol consumption. Coupled with advancements in endoscopic technology (37, 38), these developments improved both the detection and treatment outcomes of these cancers. Therefore, the observed decline during this period represents a landmark achievement resulting from the synergistic effects of prevention, diagnostic advances, and progress in therapeutic interventions.

However, despite these encouraging trends, the absolute number of new cases and deaths continues to increase. This apparent contradiction is largely attributable to demographic changes, especially population growth and aging, which have contributed to a rise in the absolute disease burden (47). An exception was identified in the decomposition analysis: in Low SDI regions, population aging was associated with a reduction in disease burden. This may be explained by public health advances in certain countries within these regions (48), such as increased emphasis on infectious disease control and significant improvements in child survival rates. These developments have enabled more people to survive into older age while avoiding substantial years of life lost (YLL) due to premature death, ultimately leading to a reduction in overall disease burden. Nevertheless, public health progress presents a double-edged sword. Although advances in screening and treatment technologies have reduced the relative risk of cancer in many high-income countries, the growing elderly population and widespread adoption of diagnostic techniques have also led to the identification of more cases (49).

## 5 Conclusion

Based on national-level data, this study proposes the global co-occurrence pattern of EC and SC for the first time. It classifies 204 countries and territories into three distinct types of co-occurrence regions, delineates the spatial distribution of both cancers within these categories, and interprets their epidemiological characteristics from global, regional, and national perspectives. The results demonstrate a significant declining trend in the burden of both cancers across these 204 countries and regions from 1990 to 2021, with SC showing the most pronounced decrease (DALYs: 172 [84.31%] of 204). Nevertheless, the disease burden of SC remained higher than that of EC in the vast majority of countries (DALYs:147 [72.06%] of 204). Further predictive analysis indicates that the global burden of EC and SC is projected to continue declining significantly by 2031. Although this study provides a comprehensive and in-depth analysis of the disease burden of these two cancers, several limitations should be acknowledged. Despite the extensive coverage of the GBD database, health data remain missing for certain countries and territories, particularly in low and middle-income regions. Moreover, GBD estimates integrate data from multiple sources,including national health departments, international organizations, and medical institutions, which vary in quality and reliability. For example, data from some countries may rely on limited surveys or modeled estimates rather than comprehensive health surveillance systems.

## Data Availability

The original contributions presented in the study are included in the article/**Supplementary Material**. Further inquiries can be directed to the corresponding authors.

## References

[1] Cui ZX Suo C Zhao YD Wang S Zhao M Chen RL . Spatiotemporal correlation analysis for the incidence of esophageal and gastric cancer from 2010 to 2019: ecological study. JMIR Cancer. (2025) 11:12. doi: 10.2196/66655, PMID: 39885591 PMC11798535

[2] Sharma R . Burden of stomach cancer incidence, mortality, disability-adjusted life years, and risk factors in 204 countries, 1990-2019: an examination of global burden of disease 2019. J Gastrointest Cancer. (2024) 55:787–99. doi: 10.1007/s12029-023-01005-3, PMID: 38265570

[3] Sun LC Zhao KK Liu XL Meng X . Global, regional, and national burden of esophageal cancer using the 2019 global burden of disease study. Sci Rep. (2025) 15:18. doi: 10.1038/s41598-025-86244-z, PMID: 39865149 PMC11770103

[4] Jiang DL Wu YX Liu L Shen YJ Li TD Lu Y . Burden of gastrointestinal tumors in asian countries, 1990-2021: an analysis for the global burden of disease study 2021. Clin Epidemiol. (2024) 16:587–601. doi: 10.2147/clep.S472553, PMID: 39252850 PMC11381218

[5] He KJ Gong GY . Global Trends and Projections of Colorectal, Esophageal and Stomach Cancer Burden among Youth Associated with Diet: A Analysis of 204 Countries and Territories from 1990 to 2019 and until 2040. Transl Oncol. (2024) 46:12. doi: 10.1016/j.tranon.2024.101984, PMID: 38824874 PMC11170277

[6] Arnold M Ferlay J Henegouwen MIV Soerjomataram I . Global burden of oesophageal and gastric cancer by histology and subsite in 2018. Gut. (2020) 69:1564–71. doi: 10.1136/gutjnl-2020-321600, PMID: 32606208

[7] Hu W Fang L Zhang H Ni R Pan G . Global disease burden of copd from 1990 to 2019 and prediction of future disease burden trend in China. Public Health. (2022) 208:89–97. doi: 10.1016/j.puhe.2022.04.015, PMID: 35728417

[8] Islami F Kamangar F . <I>Helicobacter pylori</I> and esophageal cancer risk: A meta-analysis. Cancer Prev Res. (2008) 1:329–38. doi: 10.1158/1940-6207.Capr-08-0109, PMID: 19138977 PMC3501739

[9] Obermannová R Alsina M Cervantes A Leong T Lordick F Nilsson M . Oesophageal cancer: esmo clinical practice guideline for diagnosis, treatment and follow-up. Ann Oncol. (2022) 33:992–1004. doi: 10.1016/j.annonc.2022.07.003, PMID: 35914638

[10] Li MM Cao SM Xu RH . Global trends and epidemiological shifts in gastrointestinal cancers: insights from the past four decades. Cancer Commun. (2025) 45:774–88. doi: 10.1002/cac2.70017, PMID: 40151897 PMC12328094

[11] Diao XY Guo C Jin YK Li BW Gao XH Du X . Cancer situation in China: an analysis based on the global epidemiological data released in 2024. Cancer Commun. (2025) 45:178–97. doi: 10.1002/cac2.12627, PMID: 39659114 PMC11833671

[12] Bernabe E . Trends in the global, regional, and national burden of oral conditions from 1990 to 2021: A systematic analysis for the global burden of disease study 2021. Lancet. (2025) 405:897–910. doi: 10.1016/s0140-6736(24)02811-3, PMID: 40024264

[13] Liu HX Qi JL Ren WH Zhou ZF Guo XY Yin P . Disparities in trends and drivers of the burden of tracheal, bronchus, and lung cancer among chinese population during 1990-2021: A systematic analysis for the global burden of disease study 2021. Sci Bull. (2025) 70:496–9. doi: 10.1016/j.scib.2024.12.029, PMID: 39741105

[14] . (!!! INVALID CITATION!!! (10)).

[15] Nomura S Murakami M Rauniyar SK Kondo N Tabuchi T Sakamoto H . Three decades of population health changes in Japan, 1990-2021: A subnational analysis for the global burden of disease study 2021. Lancet Public Health. (2025) 10:E321–E32. doi: 10.1016/s2468-2667(25)00044-1, PMID: 40122087 PMC11959113

[16] Zhan ZW Chen BJ Zeng Y Huang R Yu JM Guo ZQ . Long-term trends and projections of stomach cancer burden in China: insights from the gbd 2021 study. PloS One. (2025) 20:17. doi: 10.1371/journal.pone.0320751, PMID: 40198592 PMC11978042

[17] Liu D Liu H Wu YH Wang WH . Time trends in stomach cancer mortality across the brics: an age-period-cohort analysis for the gbd 2021. Front Public Health. (2025) 13:1506925. doi: 10.3389/fpubh.2025.1506925, PMID: 40093718 PMC11906716

[18] Jiang JH Xie ZQ Wang QB Wang BK Huang R Xu WK . Epidemiological trends in gastrointestinal cancers and risk factors across us states from 2000 to 2021: A systematic analysis for the global burden of disease study 2021. BMC Public Health. (2025) 25:14. doi: 10.1186/s12889-024-21192-3, PMID: 39762826 PMC11702109

[19] Tang XW Wang P Huang S Peng JY Zhang W Shi XM . Trend of gastrointestinal and liver diseases in China: results of the global burden of disease study, 2019. Chin Med J. (2024) 137:2358–68. doi: 10.1097/cm9.0000000000002975, PMID: 39227355 PMC11441872

[20] Noravesh F Behbahan SEB Saeediankia A Bahadorimonfared A Looha MA Mohammadi G . Burden, trends, projections, and spatial patterns of lip and oral cavity cancer in Iran: A time-series analysis from 1990 to 2040. BMC Public Health. (2025) 25:1282. doi: 10.1186/s12889-025-22202-8, PMID: 40186161 PMC11971863

[21] Wang SQ Zhou HF Liu Y Li YM Nie L . Trends and projections of non-hodgkin lymphoma burden (1990-2040): A global burden of disease 2021 analysis. BMC Public Health. (2025) 25:17. doi: 10.1186/s12889-025-22376-1, PMID: 40165194 PMC11959821

[22] Yang YH Li YY Pei JF Cheng MN Xu WH Shi Y . Dynamic changes in metabolic health status in chinese adults: multiple population-based surveys in shanghai, China. J Diabetes Investig. (2021) 12:1784–96. doi: 10.1111/jdi.13556, PMID: 33787069 PMC8504919

[23] Wen DG Wen XD Yang Y Chen YT Wei LZ He YT . Urban rural disparity in female breast cancer incidence rate in China and the increasing trend in parallel with socioeconomic development and urbanization in a rural setting. Thorac Cancer. (2018) 9:262–72. doi: 10.1111/1759-7714.12575, PMID: 29280294 PMC5792727

[24] Zhang HW Zheng XY Huang PP Guo LJ Zheng Y Zhang DW . The burden and trends of heart failure caused by ischaemic heart disease at the global, regional, and national levels from 1990 to 2021. Eur Heart J-Qual Care Clin Outcomes. (2024) 11:186–96. doi: 10.1093/ehjqcco/qcae094, PMID: 39537193

[25] Lu YB Shang ZZ Zhang W Hu XC Shen RQ Zhang KN . Global, regional, and national burden of spinal cord injury from 1990 to 2021 and projections for 2050: A systematic analysis for the global burden of disease 2021 study. Ageing Res Rev. (2025) 103:13. doi: 10.1016/j.arr.2024.102598, PMID: 39603465

[26] Fitzmaurice C Allen C Barber RM Barregard L Bhutta ZA Brenner H . Global, regional, and national cancer incidence, mortality, years of life lost, years lived with disability, and disability-adjusted life-years for 32 cancer groups, 1990 to 2015 a systematic analysis for the global burden of disease study. JAMA Oncol. (2017) 3:524–48. doi: 10.1001/jamaoncol.2018.2706, PMID: 27918777 PMC6103527

[27] Luo LY . Assessing validity and application scope of the intrinsic estimator approach to the age-period-cohort problem. Demography. (2013) 50:1945–67. doi: 10.1007/s13524-013-0243-z, PMID: 24072610 PMC5129181

[28] Zhao YX Zhao HP Zhao MY Yu Y Qi X Wang JH . Latest insights into the global epidemiological features, screening, early diagnosis and prognosis prediction of esophageal squamous cell carcinoma. World J Gastroenterol. (2024) 30:20. doi: 10.3748/wjg.v30.i20.2638, PMID: 38855150 PMC11154680

[29] Huang RJ Laszkowska M In H Ha Hwang J Epplein M . Controlling gastric cancer in a world of heterogeneous risk. Gastroenterology. (2023) 164:736–51. doi: 10.1053/j.gastro.2023.01.018, PMID: 36706842 PMC10270664

[30] Yang XR Zhang TC Zhang H Sang SW Chen H Zuo XL . Temporal trend of gastric cancer burden along with its risk factors in China from 1990 to 2019, and projections until 2030: comparison with Japan, South Korea, and Mongolia. biomark Res. (2021) 9:15. doi: 10.1186/s40364-021-00340-6, PMID: 34784961 PMC8597246

[31] Malfertheiner P Camargo MC El-Omar E Liou JM Peek R Schulz C . <I>Helicobacter pylori</I> infection. Nat Rev Dis Primers. (2023) 9:24. doi: 10.1038/s41572-023-00431-8, PMID: 37081005 PMC11558793

[32] Holcombe C . Helicobacter-pylori - the african enigma. Gut. (1992) 33:429–31. doi: 10.1136/gut.33.4.429, PMID: 1582581 PMC1374052

[33] Graham DY Lu H Yamaoka Y . African, asian or Indian enigma, the east asian<I>Helicobacter pylori</I>: facts or medical myths. J Dig Dis. (2009) 10:77–84. doi: 10.1111/j.1751-2980.2009.00368.x, PMID: 19426388 PMC2846403

[34] Msyamboza KP Dzamalala C Mdokwe C Kamiza S Lemerani M Dzowela T . Burden of cancer in Malawi; common types, incidence and trends: national population-based cancer registry. BMC Res Notes. (2012) 5:149. doi: 10.1186/1756-0500-5-149, PMID: 22424105 PMC3327635

[35] Chiurillo MA . Role of gene polymorphisms in gastric cancer and its precursor lesions: current knowledge and perspectives in latin american countries. World J Gastroenterol. (2014) 20:4503–15. doi: 10.3748/wjg.v20.i16.4503, PMID: 24782603 PMC4000487

[36] Alfaro T Martinez-Folgar K Stern D Wilches-Mogollon MA Muñoz MP Quick H . Variability and social patterning of cancer mortality in 343 latin american cities: an ecological study. Lancet Glob Health. (2025) 13:e268–e76. doi: 10.1016/s2214-109x(24)00446-7, PMID: 39890227 PMC11782990

[37] Sumiyama K . Past and current trends in endoscopic diagnosis for early stage gastric cancer in Japan. Gastric Cancer. (2017) 20:S20–S7. doi: 10.1007/s10120-016-0659-4, PMID: 27734273

[38] Hong S Won YJ Jun J Jung KW Kong HJ Im JS . Cancer statistics in Korea: incidence, mortality, survival, and prevalence in 2018. Cancer Res Treat. (2021) 53:301–15. doi: 10.4143/crt.2021.291, PMID: 33735559 PMC8053867

[39] Sun DQ Muelder DT Li YG Nieboer D Park JY Suh M . The effect of nationwide organized cancer screening programs on gastric cancer mortality: A synthetic control study. Gastroenterology. (2024) 166:503–14. doi: 10.1053/j.gastro.2023.11.286, PMID: 38007053

[40] Lee S Jun JK Suh M Park B Noh DK Jung KW . Gastric cancer screening uptake trends in Korea: results for the national cancer screening program from 2002 to 2011<I>a prospective cross</I>-<I>Sectional study</I>. Med (Baltimore). (2015) 94:6. doi: 10.1097/md.0000000000000533, PMID: 25715251 PMC4554157

[41] Mabe K Inoue K Kamada T Kato K Kato M Haruma K . Endoscopic screening for gastric cancer in Japan: current status and future perspectives. Dig Endosc. (2022) 34:412–9. doi: 10.1111/den.14063, PMID: 34143908

[42] Sano H Goto R Hamashima C . What is the most effective strategy for improving the cancer screening rate in Japan? Asian Pac J Cancer Prev. (2014) 15:2607–12. doi: 10.7314/apjcp.2014.15.6.2607, PMID: 24761871

[43] Hiroi S Sugano K Tanaka S Kawakami K . Impact of health insurance coverage for<I>Helicobacter pylori</I> gastritis on the trends in eradication therapy in Japan: retrospective observational study and simulation study based on realworld data. BMJ Open. (2017) 7:10. doi: 10.1136/bmjopen-2017-015855, PMID: 28760790 PMC5642792

[44] Allemani C Matsuda T Di Carlo V Harewood R Matz M Niksic M . Global surveillance of trends in cancer survival 2000-14 (Concord-3): analysis of individual records for 37 513–025 patients diagnosed with one of 18 cancers from 322 population-based registries in 71 countries. Lancet. (2018) 391:1023–75. doi: 10.1016/s0140-6736(17)33326-3, PMID: 29395269 PMC5879496

[45] Shah SL Cappell K Sedgley R Pelletier C Jacob R Bonafede M . Diagnosis and treatment patterns among patients with newly diagnosed<I>Helicobacter pylori</I> infection in the United States 2016-2019. Sci Rep. (2023) 13:10. doi: 10.1038/s41598-023-28200-3, PMID: 36697470 PMC9876904

[46] Wroblewski LE Peek RM Wilson KT . <I>Helicobacter pylori</I> and gastric cancer: factors that modulate disease risk. Clin Microbiol Rev. (2010) 23:713–39. doi: 10.1128/cmr.00011-10, PMID: 20930071 PMC2952980

[47] Ono T Wada H Ishikawa H Tamamura H Tokumaru S . Clinical results of proton beam therapy for esophageal cancer: multicenter retrospective study in Japan. Cancers. (2019) 11:11. doi: 10.3390/cancers11070993, PMID: 31315281 PMC6679064

[48] du Preez K Gabardo BMA Kabra SK Triasih R Lestari T Kal M . Priority activities in child and adolescent tuberculosis to close the policy-practice gap in low- and middle-income countries. Pathogens. (2022) 11:26. doi: 10.3390/pathogens11020196, PMID: 35215139 PMC8878304

[49] Nolen SC Evans MA Fischer A Corrada MM Kawas CH Bota DA . Cancer-incidence, prevalence and mortality in the oldest-old. A comprehensive review. Mech Ageing Dev. (2017) 164:113–26. doi: 10.1016/j.mad.2017.05.002, PMID: 28502820 PMC7788911

