# Correction: Co-occurrence patterns of esophageal and stomach cancer across 204 countries and territories: a spatial correspondence and systematic analysis

**DOI:** 10.3389/fonc.2025.1728915

**Published:** 2025-11-07

**Authors:** Jiayao Xu, Jiabei Gong, Huiqiong Han, Zehua Wang, Wenjia Wang, Lei Wang, Xin Sui, Guanyu Chen, Yongxu Jia, Yanru Qin

**Affiliations:** 1 Department of Oncology, The First Affiliated Hospital of Zhengzhou University, Zhengzhou, China; 2 Department of Nephropathy, The First Affiliated Hospital of Zhengzhou University, Zhengzhou, China; 3 Department of Experimental Orofacial Medicine, University of Marburg, Marburg, Germany

**Keywords:** esophageal cancer, stomach cancer, co-occurrence patterns, age-period-cohort model, BAPC model, prediction

The order of authors in the author list of the published paper was erroneously given as:

“Yanru Qin^1*^, Jiayao Xu ^1†^, Jiabei Gong^2†^, Huiqiong Han^1^, Zehua Wang^1^, Wenjia Wang^1^, Lei Wang^1^, Xin Sui^1^, Guanyu Chen^3*^ and Yongxu Jia^1*^”

The correct author list reads:

“Jiayao Xu^1†^, Jiabei Gong^2†^, Huiqiong Han^1^, Zehua Wang^1^, Wenjia Wang^1^, Lei Wang^1^, Xin Sui^1^, Guanyu Chen^3*^, Yongxu Jia^1*^ and Yanru Qin^1*^”

An incorrect number was provided for “Key Development Project of the Department of Science and Technology”. The current number is “2015C03Bd051”.

The original version of this article has been updated.

