# Supplementary material for: Co-occurrence patterns of esophageal and stomach cancer across 204 countries and territories: a spatial correspondence and systematic analysis
Source: Front Oncol. 2025 Oct 17;15:1613839. doi: 10.3389/fonc.2025.1613839 (PMC12576481; doi:10.3389/fonc.2025.1613839)
Supplement: Supplementary file 1 [file DataSheet1.docx]

**Supplementary Figure 1.** Numeber and ASR of esophageal cancer in 204 countries and regions in 2021

**Supplementary Figure 2.** Numeber and ASR of stomach cancer in 204 countries and regions in 2021

**Supplementary Figure 3.** Age-period-cohort analysis results of esophageal cancer for ASR

**Supplementary Figure 4.** Age-period-cohort analysis results of stomach cancer for ASR

**Supplementary Figure 5.** Number predicted by BAPC for global Esophageal and Stomach cancer from 1990 to 2031

**Supplementary Table S1.** Age-standardised rate of esophageal cancer and stomach cancer at 5 SDI level between 1990 and 2021

**Supplementary Table S2.** Age-standardised rate of esophageal cancer at nation level between 1990 and 2021

**Supplementary Table S3.** Age-standardised rate of stomach cancer at nation level between 1990 and 2021

**Supplementary Table S4.** cocurrence pattern of incidence

**Supplementary Table S5.** cocurrence pattern of deaths

**Supplementary Table S6.** cocurrence pattern of dalys

**Supplementary Table S7.** ASR predicted by BAPC for global Esophageal and Stomach cancer from 1990 to 2031 (per 100,000)

**Supplementary Table S8.** Number predicted by BAPC for global Esophageal and Stomach cancer from 1990 to 2031

Supplementary Figure 1. Numeber and ASR of esophageal cancer in 204 countries and regions in 2021


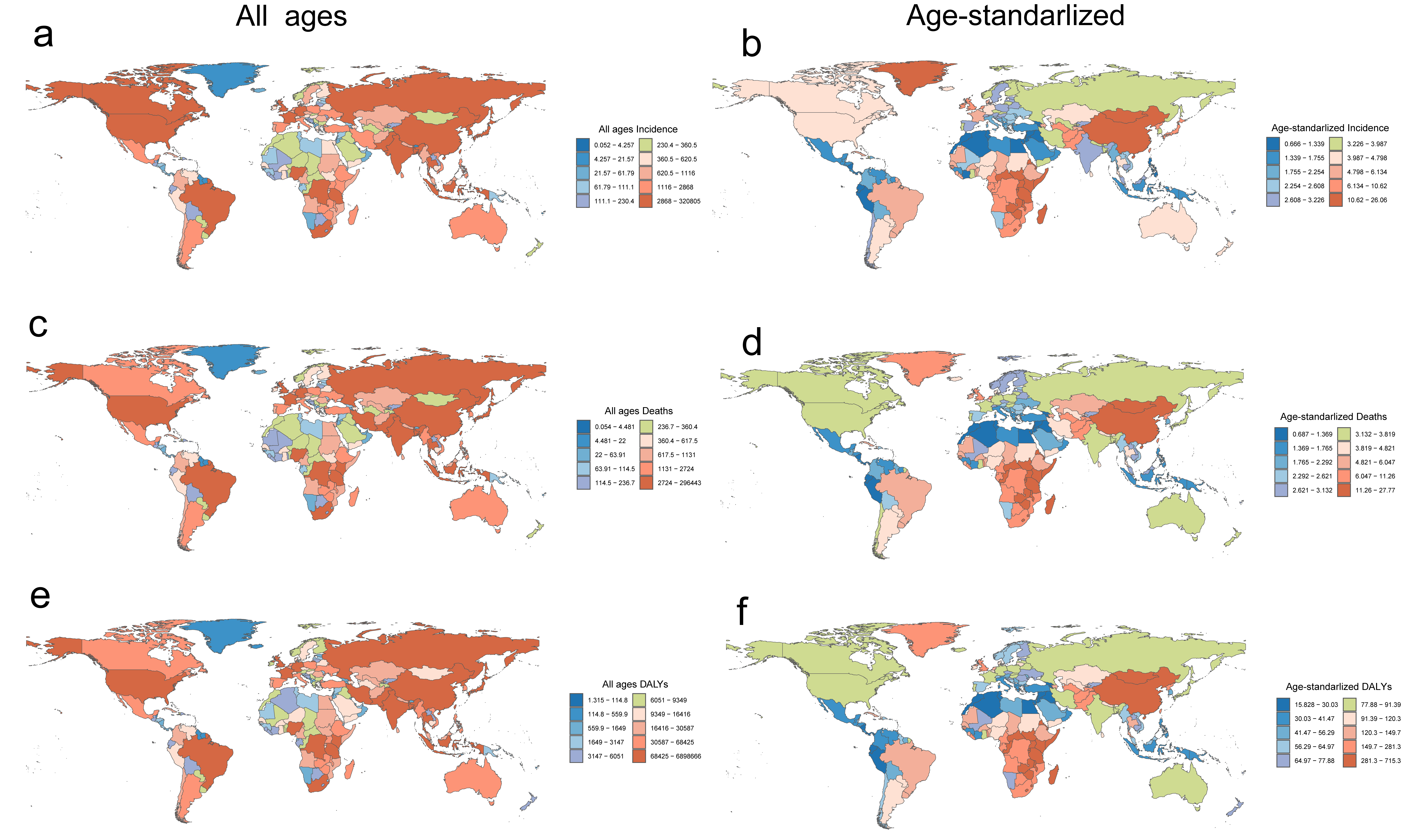


Supplementary Figure 2. Numeber and ASR of stomach cancer in 204 countries and regions in 2021


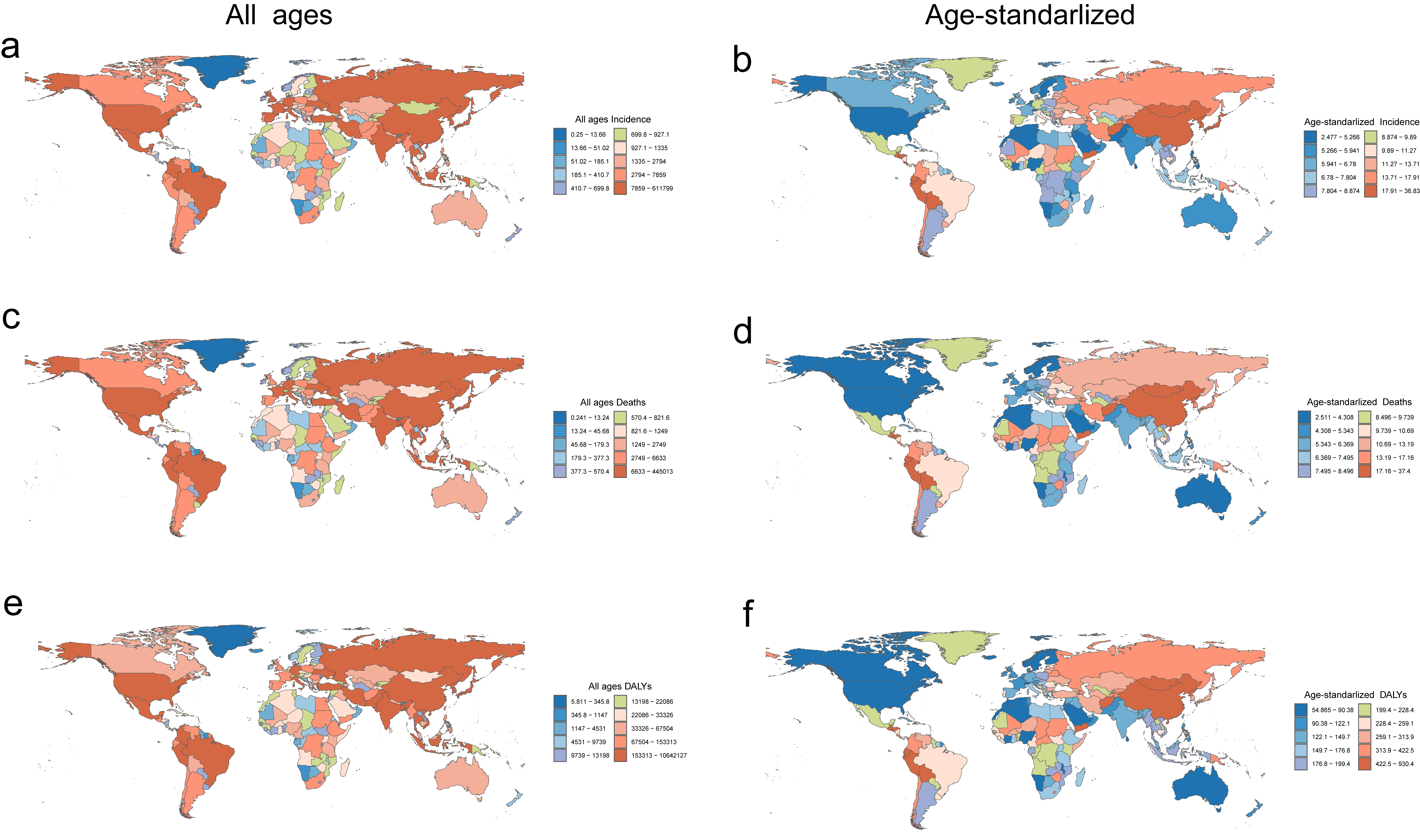


Supplementary Figure 3. Age-period-cohort analysis results of esophageal cancer for ASR


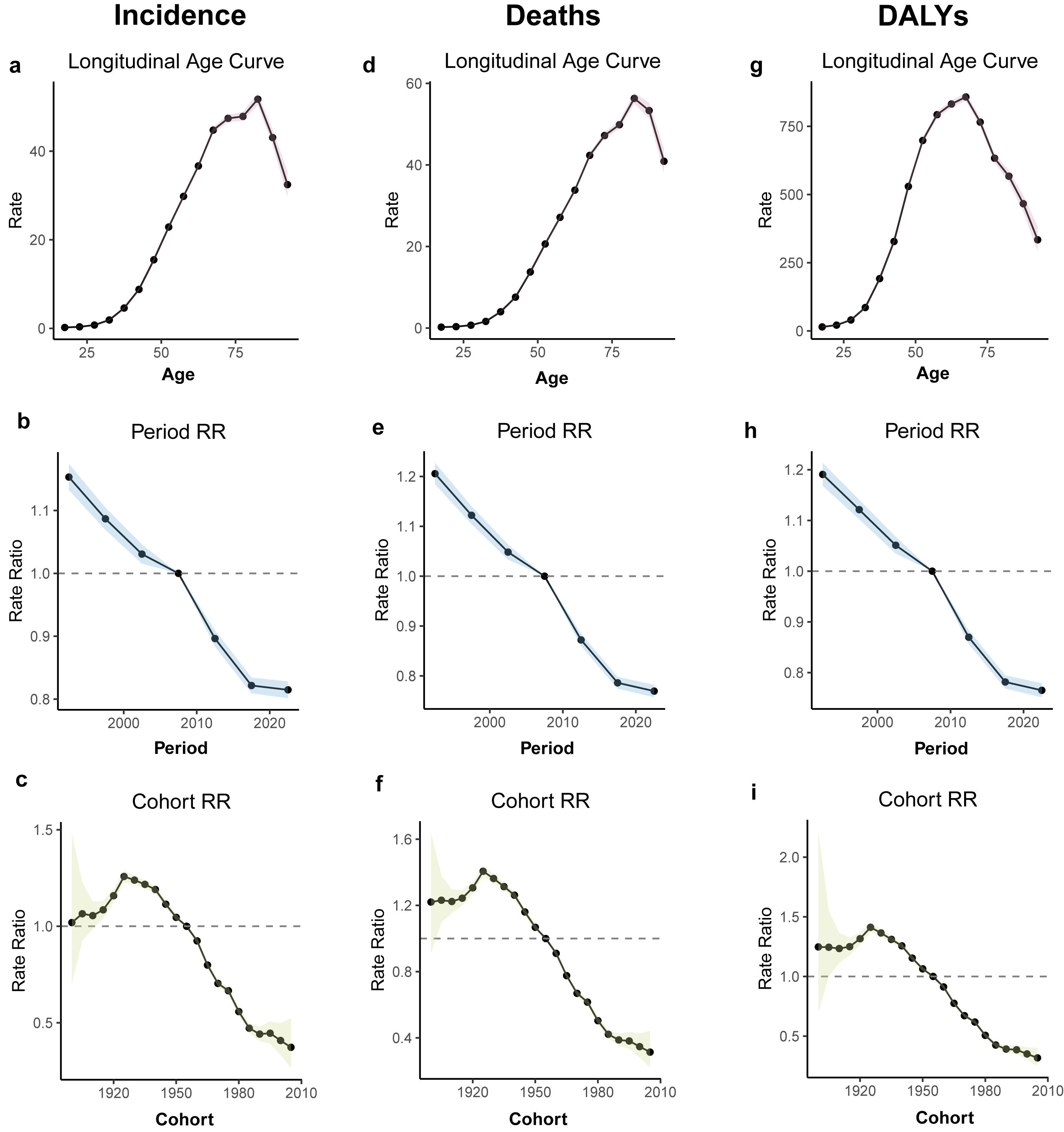


Supplementary Figure 4. Age-period-cohort analysis results of stomach cancer for ASR


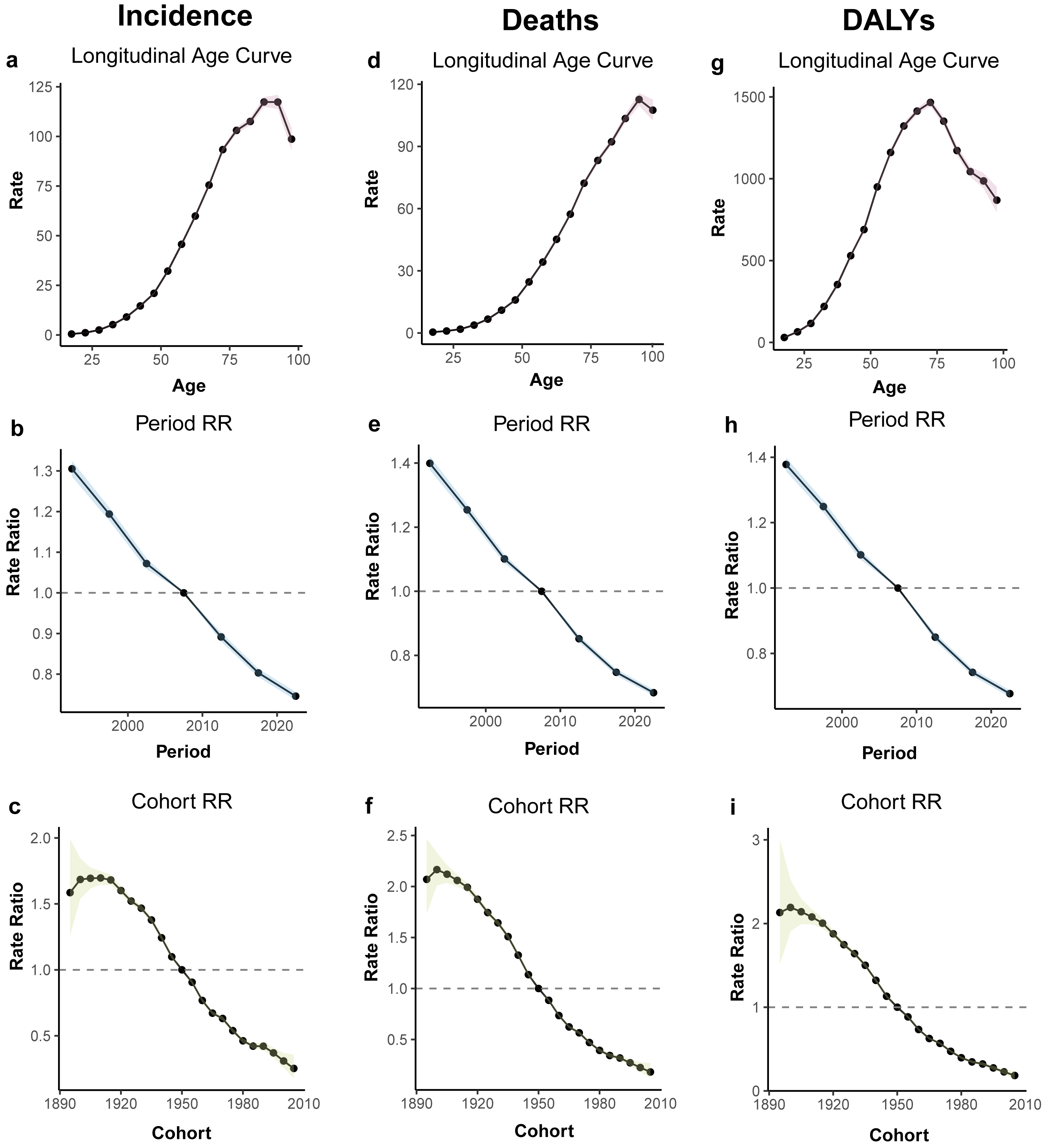


Supplementary Figure 5. Number predicted by BAPC for global Esophageal and Stomach cancer from 1990 to 2031


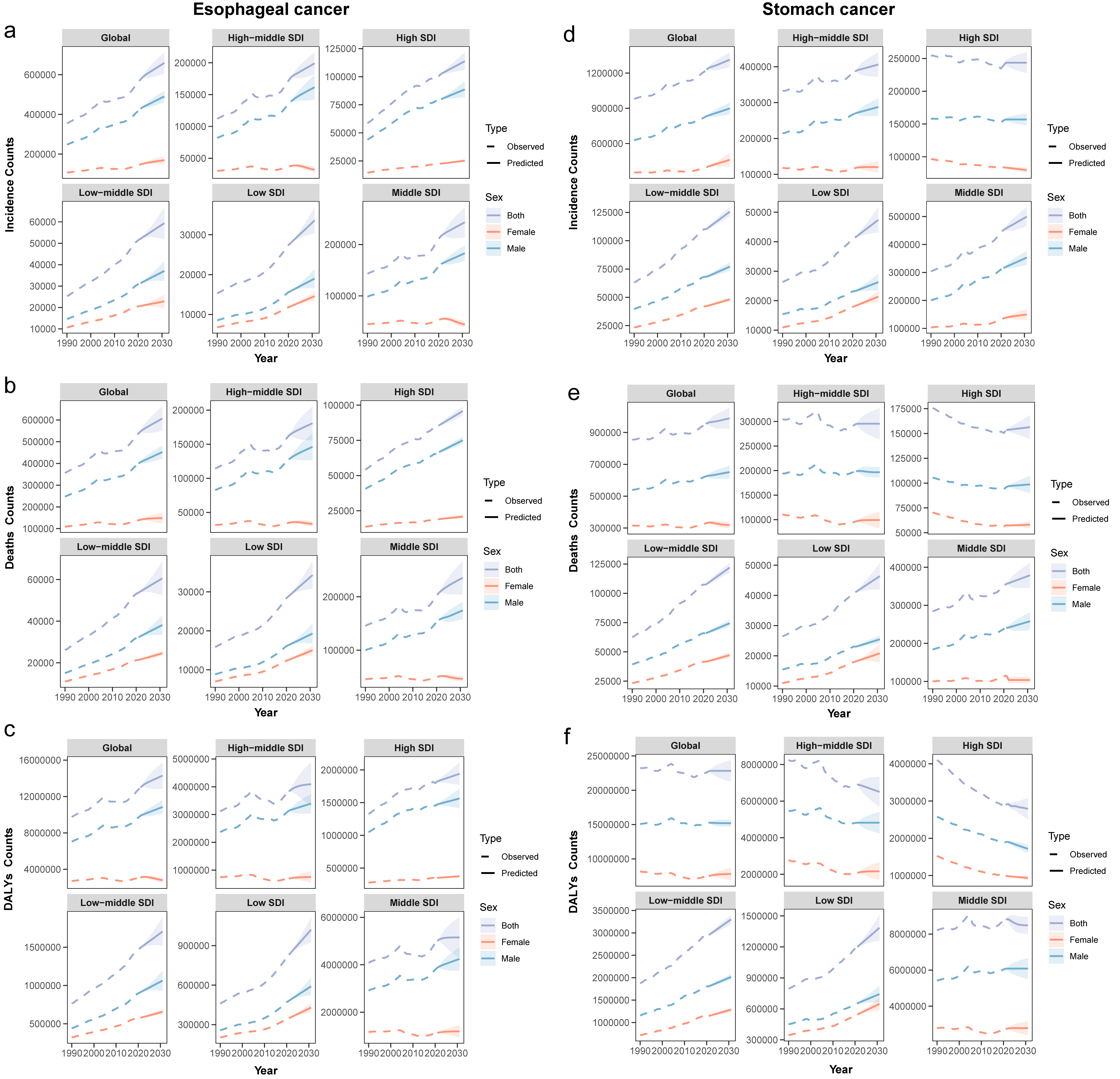


Supplementary Table S1. Age-standardised rate of esophageal cancer and stomach cancer at 5 SDI level between 1990 and 2021

| **Cause** | **Location** | **Incidence** | | | | | **Deaths** | | | | | **DALYs** | | | | |
| --- | --- | --- | --- | --- | --- | --- | --- | --- | --- | --- | --- | --- | --- | --- | --- | --- |
|  |  | **Number,1990  (thousands,95%UI)** | **ASIR per 100,000, 1990 (95%UI)** | **Number,2021  (thousands,95%UI)** | **ASIR per 100,000, 2021 (95%UI)** | **AAPC (95%CI) 1990-2021** | **Number,1990  (thousands,95%UI)** | **ASMR per 100,000, 1990 (95%UI)** | **Number,2021  (thousands,95%UI)** | **ASMR per 100,000, 2021 (95%UI)** | **AAPC (95%CI) 1990-2021** | **Number,1990  (thousands,95%UI)** | **ASDR per 100,000, 1990 (95%UI)** | **Number,2021 (thousands,95%UI)** | **ASDR per 100,000, 2021 (95%UI)** | **AAPC (95%CI) 1990-2021** |
| Esophageal  Cancer | Global | 354.731 (317.512,388.914) | 8.857 (7.962,9.694) | 576.529 (509.492,645.648) | 6.655 (5.883,7.45) | -0.940 (-0.982,-0.906) | 356.263 (319.363,390.154) | 9.022 (8.112,9.866) | 538.602 (475.944,603.406) | 6.255 (5.527,7.003) | -1.197 (-1.242,-1.162) | 9753.566 (8719.319,10739.561) | 235.319 (210.517,258.681) | 12999.265 (11522.861,14605.268) | 148.561 (131.709,166.818) | -1.500 (-1.543,-1.466) |
|  | High SDI | 58.596 (56.147,60.082) | 5.358 (5.138,5.492) | 102.51 (95.224,107.348) | 4.943 (4.631,5.156) | -0.271 (-0.298,-0.253) | 54.24 (51.962,55.653) | 4.934 (4.732,5.062) | 85.652 (79.16,89.95) | 4.02 (3.746,4.205) | -0.670 (-0.714,-0.640) | 1325.837 (1286.516,1357.512) | 123.988 (120.471,126.95) | 1825.459 (1719.026,1902.714) | 93.953 (89.284,97.917) | -0.884 (-0.937,-0.836) |
|  | High-middle SDI | 112.267 (98.613,125.911) | 11.17 (9.849,12.491) | 176.768 (145.141,214.116) | 8.841 (7.258,10.703) | -0.754 (-0.812,-0.697) | 114.332 (100.753,128.04) | 11.519 (10.191,12.867) | 162.43 (134.262,195.467) | 8.128 (6.719,9.766) | -1.157 (-1.240,-1.090) | 3116.679 (2731.977,3509.251) | 303.104 (265.931,341.095) | 3834.291 (3157.464,4667.629) | 192.562 (158.696,234.03) | -1.505 (-1.590,-1.438) |
|  | Low SDI | 15.305 (12.751,17.275) | 6.692 (5.583,7.514) | 27.96 (23.834,32.184) | 5.494 (4.705,6.316) | -0.624 (-0.634,-0.614) | 15.828 (13.205,17.869) | 7.149 (5.968,8.013) | 28.924 (24.611,33.445) | 5.89 (5.023,6.797) | -0.610 (-0.621,-0.599) | 460.138 (380.391,517.924) | 185.383 (154.417,209.222) | 830.121 (701.259,964.024) | 148.672 (126.111,172.196) | -0.698 (-0.708,-0.687) |
|  | Low-middle SDI | 25.326 (22.893,29.038) | 4.095 (3.681,4.698) | 52.104 (47.166,59.926) | 3.592 (3.241,4.151) | -0.411 (-0.452,-0.372) | 26.141 (23.617,30.035) | 4.36 (3.924,5.017) | 53.724 (48.513,61.806) | 3.793 (3.418,4.389) | -0.436 (-0.474,-0.400) | 763.111 (692.292,878.969) | 113.845 (102.949,130.891) | 1491.634 (1348.142,1724.084) | 97.097 (87.736,111.836) | -0.500 (-0.531,-0.477) |
|  | Middle SDI | 143.088 (120.408,165.385) | 13.679 (11.493,15.775) | 216.951 (182.212,258.446) | 8.103 (6.782,9.624) | -1.678 (-1.707,-1.644) | 145.568 (123.678,168.238) | 14.31 (12.195,16.447) | 207.634 (174.863,246.498) | 7.913 (6.65,9.337) | -1.899 (-1.931,-1.866) | 4083.703 (3438.091,4731.71) | 365.58 (309.417,422.251) | 5011.783 (4233.898,5964.294) | 180.646 (153.165,214.627) | -2.260 (-2.288,-2.228) |
| Stomach Cancer | Global | 980.899 (891.307,1072.236) | 24.763 (22.58,27.002) | 1230.233 (1052.35,1409.97) | 14.328 (12.226,16.408) | -1.763 (-1.812,-1.728) | 854.185 (772.885,939.973) | 22.006 (20.028,24.187) | 954.374 (821.751,1089.577) | 11.199 (9.618,12.734) | -2.173 (-2.194,-2.151) | 23237.292 (20605.349,25526.194) | 559.721 (499.087,615.772) | 22786.633 (19576.344,26118.869) | 262.748 (226.079,301.024) | -2.424 (-2.452,-2.399) |
|  | High SDI | 254.259 (242.222,261.672) | 23.133 (22.047,23.8) | 239.119 (215.071,256.938) | 11.155 (10.207,11.905) | -2.337 (-2.402,-2.310) | 175.683 (166.255,181.197) | 15.863 (15.01,16.371) | 153.539 (136.67,165.354) | 6.834 (6.18,7.336) | -2.690 (-2.762,-2.656) | 4099.422 (3902.053,4218.897) | 381.131 (362.206,392.227) | 2897.404 (2654.584,3101.177) | 146.104 (135.56,155.885) | -3.074 (-3.123,-3.038) |
|  | High-middle SDI | 332.614 (298.475,360.843) | 33.327 (30.086,36.102) | 387.196 (315.957,457.286) | 19.62 (16.016,23.134) | -1.694 (-1.744,-1.638) | 304.773 (274.911,330.96) | 31.084 (28.093,33.705) | 295.105 (244.787,343.611) | 14.929 (12.397,17.364) | -2.348 (-2.395,-2.302) | 8241.238 (7281.203,9010.822) | 802.75 (711.792,876.759) | 6901.607 (5703.61,8141.888) | 353.179 (291.888,416.778) | -2.623 (-2.672,-2.578) |
|  | Low SDI | 26.334 (20.975,30.114) | 11.408 (9.041,13.026) | 41.388 (32.6,46.956) | 8.132 (6.435,9.222) | -1.076 (-1.102,-1.051) | 26.41 (21.016,30.213) | 11.898 (9.41,13.573) | 41.192 (32.515,46.864) | 8.462 (6.71,9.601) | -1.084 (-1.115,-1.054) | 795.64 (636.078,910.967) | 311.981 (248.616,357.312) | 1198.903 (940.407,1371.29) | 209.77 (165.604,238.952) | -1.264 (-1.294,-1.239) |
|  | Low-middle SDI | 63.086 (55.336,76.805) | 10.139 (8.979,12.416) | 110.396 (97.098,126.222) | 7.684 (6.715,8.771) | -0.896 (-0.929,-0.850) | 62.598 (54.993,76.278) | 10.448 (9.246,12.789) | 107.456 (94.064,122.211) | 7.709 (6.693,8.76) | -0.982 (-1.022,-0.934) | 1874.805 (1629.366,2261.562) | 274.452 (239.886,333.491) | 2953.125 (2599.859,3357.038) | 192.559 (169.425,219.14) | -1.132 (-1.159,-1.100) |
|  | Middle SDI | 303.889 (262.41,351.115) | 28.887 (25.129,33.443) | 451.465 (368.224,542.292) | 16.913 (13.785,20.276) | -1.734 (-1.763,-1.704) | 284.016 (246.76,329.474) | 28.098 (24.614,32.65) | 356.459 (294.16,423.77) | 13.716 (11.309,16.218) | -2.318 (-2.349,-2.290) | 8208.596 (7034.443,9506.074) | 721.809 (624.062,835.383) | 8820.717 (7336.337,10567.624) | 320.242 (266.074,382.872) | -2.623 (-2.652,-2.596) |

Supplementary Table S2. Age-standardised rate of esophageal cancer at nation level between 1990 and 2021

| **location** | **Incidence** | | | | | **Deaths** | | | | | **DALYs** | | | | |
| --- | --- | --- | --- | --- | --- | --- | --- | --- | --- | --- | --- | --- | --- | --- | --- |
|  | **Number of cases, 1990  (thousands)** | **Age-standardised rate per 100,000 population, 1990** | **Number of cases, 2021  (thousands)** | **Age-standardised rate per 100,000 population, 2021** | **Average Annual Percent Change 1990 to 2021** | **Number of cases, 1990  (thousands)** | **Age-standardised rate per 100,000 population, 1990** | **Number of cases, 2021  (thousands)** | **Age-standardised rate per 100,000 population, 2021** | **Average Annual Percent Change 1990 to 2021** | **Number of cases, 1990  (thousands)** | **Age-standardised rate per 100,000 population, 1990** | **Number of cases, 2021  (thousands)** | **Age-standardised rate per 100,000 population, 2021** | **Average Annual Percent Change 1990 to 2021** |
| Afghanistan | 0.659 (0.318,0.95) | 9.32 (4.618,13.265) | 0.717 (0.331,1.082) | 7.262 (3.562,10.674) | -0.791 (-0.808,-0.772) | 0.685 (0.333,0.987) | 9.915 (4.936,14.043) | 0.733 (0.347,1.1) | 7.774 (3.853,11.291) | -0.770 (-0.785,-0.752) | 19.373 (8.994,28.546) | 261.911 (124.815,382.363) | 22.622 (10.197,34.778) | 197.42 (92.406,295.412) | -0.902 (-0.921,-0.877) |
| Albania | 0.038 (0.032,0.046) | 1.881 (1.577,2.248) | 0.061 (0.044,0.08) | 1.41 (1.033,1.854) | -1.023 (-1.177,-0.907) | 0.04 (0.034,0.048) | 2.048 (1.712,2.454) | 0.065 (0.048,0.086) | 1.515 (1.113,1.998) | -1.040 (-1.252,-0.864) | 1.058 (0.884,1.271) | 48.872 (40.836,58.739) | 1.492 (1.088,1.971) | 35.343 (25.916,46.37) | -1.150 (-1.384,-0.989) |
| Algeria | 0.082 (0.067,0.099) | 0.741 (0.607,0.881) | 0.234 (0.18,0.295) | 0.699 (0.546,0.874) | -0.171 (-0.190,-0.149) | 0.086 (0.07,0.104) | 0.821 (0.669,0.974) | 0.241 (0.185,0.303) | 0.753 (0.591,0.936) | -0.257 (-0.280,-0.229) | 2.244 (1.832,2.701) | 18.217 (14.881,21.724) | 5.983 (4.533,7.552) | 16.361 (12.544,20.522) | -0.341 (-0.356,-0.328) |
| American Samoa | 0 (0,0) | 1.193 (1.005,1.437) | 0.001 (0.001,0.001) | 1.565 (1.286,1.888) | 0.875 (0.739,1.042) | 0 (0,0) | 1.295 (1.092,1.555) | 0.001 (0.001,0.001) | 1.668 (1.382,2.006) | 0.815 (0.687,0.979) | 0.008 (0.006,0.009) | 30.778 (25.629,37.442) | 0.02 (0.016,0.024) | 39.924 (32.068,48.771) | 0.837 (0.688,1.015) |
| Andorra | 0.001 (0.001,0.002) | 1.996 (1.385,2.865) | 0.002 (0.001,0.003) | 1.347 (0.866,1.952) | -1.383 (-1.605,-1.226) | 0.001 (0.001,0.002) | 1.933 (1.339,2.749) | 0.002 (0.001,0.003) | 1.176 (0.764,1.69) | -1.731 (-1.941,-1.574) | 0.029 (0.02,0.041) | 48.794 (33.287,70.611) | 0.044 (0.028,0.064) | 28.598 (18.335,41.583) | -1.847 (-2.069,-1.686) |
| Angola | 0.467 (0.312,0.637) | 11.664 (8.071,15.619) | 0.937 (0.671,1.235) | 7.969 (5.666,10.366) | -1.233 (-1.299,-1.171) | 0.477 (0.321,0.646) | 12.444 (8.688,16.538) | 0.959 (0.689,1.264) | 8.581 (6.125,11.203) | -1.202 (-1.264,-1.141) | 14.593 (9.76,19.971) | 326.216 (220.626,440.802) | 28.518 (20.271,38.319) | 212.846 (153.258,279.877) | -1.388 (-1.463,-1.316) |
| Antigua and Barbuda | 0.002 (0.002,0.002) | 3.283 (3.049,3.514) | 0.003 (0.003,0.003) | 2.83 (2.65,3.024) | -0.628 (-0.853,-0.466) | 0.002 (0.002,0.002) | 3.505 (3.262,3.746) | 0.003 (0.003,0.003) | 2.993 (2.806,3.186) | -0.466 (-0.721,-0.230) | 0.043 (0.04,0.045) | 83.787 (78.072,89.387) | 0.076 (0.07,0.081) | 68.169 (63.536,73.048) | -0.744 (-0.943,-0.591) |
| Argentina | 2.231 (2.112,2.357) | 6.978 (6.611,7.366) | 2.327 (2.152,2.526) | 4.126 (3.823,4.471) | -1.564 (-1.652,-1.480) | 2.378 (2.256,2.517) | 7.521 (7.125,7.96) | 2.469 (2.278,2.673) | 4.343 (4.011,4.694) | -1.635 (-1.722,-1.552) | 56.905 (53.873,60.084) | 175.578 (166.222,185.247) | 53.733 (49.964,58.119) | 97.432 (90.624,105.336) | -1.816 (-1.945,-1.722) |
| Armenia | 0.075 (0.069,0.08) | 2.767 (2.539,2.997) | 0.045 (0.039,0.051) | 1.004 (0.882,1.149) | -3.353 (-3.697,-3.026) | 0.079 (0.072,0.085) | 2.977 (2.721,3.243) | 0.048 (0.042,0.055) | 1.075 (0.944,1.235) | -3.381 (-3.710,-3.054) | 2.065 (1.902,2.222) | 72.455 (66.544,78.156) | 1.109 (0.972,1.274) | 24.944 (21.879,28.623) | -3.474 (-3.718,-3.243) |
| Australia | 0.854 (0.804,0.909) | 4.348 (4.099,4.619) | 1.853 (1.655,1.996) | 4.059 (3.672,4.367) | -0.295 (-0.423,-0.186) | 0.831 (0.781,0.886) | 4.241 (3.996,4.512) | 1.757 (1.574,1.892) | 3.75 (3.367,4.025) | -0.495 (-0.594,-0.407) | 18.924 (17.861,20.075) | 97.449 (91.919,103.327) | 35.247 (32.133,37.878) | 82.268 (75.671,88.238) | -0.611 (-0.704,-0.529) |
| Austria | 0.301 (0.284,0.318) | 2.669 (2.518,2.816) | 0.467 (0.424,0.504) | 2.669 (2.438,2.872) | 0.022 (-0.159,0.210) | 0.294 (0.278,0.312) | 2.568 (2.432,2.712) | 0.417 (0.376,0.45) | 2.307 (2.107,2.483) | -0.311 (-0.462,-0.138) | 7.183 (6.753,7.602) | 67.101 (63.067,71.199) | 9.095 (8.314,9.795) | 55.39 (50.908,59.589) | -0.640 (-0.822,-0.471) |
| Azerbaijan | 0.417 (0.361,0.473) | 8.333 (7.245,9.558) | 0.534 (0.395,0.687) | 5.354 (3.957,6.87) | -1.403 (-1.524,-1.312) | 0.437 (0.378,0.497) | 8.931 (7.743,10.241) | 0.559 (0.412,0.718) | 5.787 (4.249,7.434) | -1.375 (-1.492,-1.284) | 12.063 (10.484,13.715) | 227.432 (197.38,260.2) | 14.739 (10.875,18.782) | 135.654 (99.945,174.282) | -1.686 (-1.799,-1.593) |
| Bahamas | 0.01 (0.009,0.011) | 6.399 (5.882,6.97) | 0.022 (0.017,0.027) | 5.126 (4.062,6.399) | -0.428 (-0.750,-0.224) | 0.01 (0.009,0.011) | 6.65 (6.114,7.249) | 0.022 (0.017,0.028) | 5.333 (4.252,6.629) | -0.431 (-0.783,-0.213) | 0.295 (0.269,0.324) | 179.949 (164.398,196.846) | 0.619 (0.487,0.782) | 139.843 (110.499,175.882) | -0.708 (-0.964,-0.481) |
| Bahrain | 0.006 (0.005,0.007) | 3.836 (3.286,4.447) | 0.016 (0.012,0.02) | 2.171 (1.743,2.669) | -1.831 (-1.928,-1.743) | 0.006 (0.005,0.007) | 4.223 (3.638,4.898) | 0.015 (0.012,0.019) | 2.315 (1.859,2.856) | -1.937 (-2.035,-1.853) | 0.163 (0.137,0.19) | 90.459 (76.758,105.134) | 0.418 (0.325,0.549) | 45.61 (36.181,57.243) | -2.222 (-2.296,-2.154) |
| Bangladesh | 2.462 (1.757,3.274) | 5.139 (3.657,6.807) | 4.508 (3.207,6.247) | 3.275 (2.335,4.512) | -1.328 (-1.432,-1.234) | 2.549 (1.821,3.398) | 5.435 (3.878,7.191) | 4.695 (3.318,6.525) | 3.484 (2.46,4.816) | -1.304 (-1.409,-1.205) | 74.614 (53.817,100.296) | 146.086 (104.465,197.089) | 124.793 (87.65,175.596) | 86.657 (61.035,121.663) | -1.576 (-1.661,-1.497) |
| Barbados | 0.016 (0.015,0.017) | 5.704 (5.357,6.08) | 0.026 (0.02,0.032) | 5.008 (3.937,6.183) | -0.463 (-0.624,-0.317) | 0.018 (0.017,0.019) | 6.085 (5.713,6.472) | 0.027 (0.022,0.033) | 5.276 (4.166,6.441) | -0.524 (-0.684,-0.379) | 0.393 (0.371,0.419) | 146.566 (137.373,156.583) | 0.622 (0.479,0.769) | 123.56 (95.661,153.383) | -0.530 (-0.688,-0.385) |
| Belarus | 0.377 (0.347,0.408) | 2.864 (2.629,3.104) | 0.487 (0.398,0.602) | 3.045 (2.497,3.75) | -0.271 (-0.474,-0.071) | 0.383 (0.352,0.414) | 2.912 (2.675,3.156) | 0.461 (0.373,0.573) | 2.861 (2.323,3.553) | -0.285 (-1.173,0.023) | 10.667 (9.863,11.553) | 81.188 (75.214,88.02) | 12.754 (10.232,15.908) | 81.52 (65.9,101.555) | -0.196 (-1.143,0.149) |
| Belgium | 0.611 (0.57,0.649) | 4.101 (3.836,4.35) | 1.031 (0.94,1.116) | 4.595 (4.233,4.95) | 0.381 (0.221,0.539) | 0.614 (0.573,0.652) | 4.063 (3.799,4.315) | 0.961 (0.871,1.047) | 4.114 (3.77,4.443) | 0.070 (-0.075,0.212) | 14.396 (13.515,15.32) | 101.204 (95.312,107.157) | 20.428 (18.839,22.068) | 97.697 (90.312,105.099) | -0.178 (-0.355,-0.016) |
| Belize | 0.002 (0.002,0.002) | 1.938 (1.807,2.069) | 0.006 (0.006,0.007) | 2.127 (1.851,2.434) | 0.392 (0.057,0.599) | 0.002 (0.002,0.002) | 2.069 (1.927,2.208) | 0.007 (0.006,0.008) | 2.243 (1.951,2.551) | 0.363 (-0.009,0.590) | 0.048 (0.045,0.052) | 51.42 (48.054,55.101) | 0.184 (0.158,0.21) | 56.643 (48.89,64.708) | 0.372 (0.063,0.560) |
| Benin | 0.062 (0.05,0.075) | 3.136 (2.584,3.803) | 0.257 (0.193,0.336) | 5.019 (3.753,6.503) | 1.541 (1.502,1.572) | 0.065 (0.054,0.079) | 3.372 (2.815,4.087) | 0.268 (0.201,0.349) | 5.406 (4.049,7.019) | 1.545 (1.511,1.574) | 1.73 (1.413,2.106) | 84.444 (69.36,102.554) | 7.436 (5.517,9.902) | 132.869 (99.505,174.308) | 1.493 (1.451,1.526) |
| Bermuda | 0.004 (0.004,0.005) | 6.976 (6.412,7.54) | 0.006 (0.005,0.007) | 4.185 (3.527,5.026) | -1.749 (-1.899,-1.634) | 0.004 (0.004,0.005) | 7.316 (6.744,7.88) | 0.006 (0.005,0.007) | 4.056 (3.424,4.844) | -1.969 (-2.123,-1.857) | 0.112 (0.103,0.121) | 176.391 (162.354,190.778) | 0.121 (0.101,0.145) | 95.355 (79.286,114.35) | -2.061 (-2.223,-1.945) |
| Bhutan | 0.012 (0.009,0.017) | 5.011 (3.576,6.791) | 0.021 (0.015,0.03) | 3.557 (2.598,4.863) | -1.089 (-1.115,-1.058) | 0.013 (0.009,0.017) | 5.337 (3.783,7.221) | 0.023 (0.016,0.031) | 3.821 (2.756,5.266) | -1.055 (-1.079,-1.026) | 0.387 (0.277,0.528) | 139.776 (100.362,189.612) | 0.577 (0.411,0.808) | 91.872 (65.633,128.496) | -1.348 (-1.377,-1.314) |
| Bolivia  (Plurinational State of) | 0.085 (0.065,0.108) | 2.776 (2.126,3.53) | 0.191 (0.14,0.257) | 2.21 (1.638,2.952) | -0.740 (-0.789,-0.700) | 0.091 (0.07,0.116) | 3.059 (2.347,3.877) | 0.207 (0.152,0.278) | 2.453 (1.823,3.269) | -0.716 (-0.768,-0.674) | 2.338 (1.78,3.019) | 70.783 (53.881,90.88) | 4.872 (3.51,6.694) | 52.851 (38.366,71.773) | -0.924 (-0.945,-0.904) |
| Bosnia and Herzegovina | 0.098 (0.084,0.116) | 2.31 (1.958,2.714) | 0.131 (0.098,0.167) | 2.13 (1.591,2.714) | -0.188 (-0.329,-0.069) | 0.102 (0.087,0.12) | 2.448 (2.076,2.88) | 0.138 (0.103,0.175) | 2.218 (1.654,2.816) | -0.192 (-0.337,-0.037) | 2.864 (2.443,3.397) | 63.474 (54.407,74.97) | 3.362 (2.482,4.283) | 56.568 (41.557,71.857) | -0.301 (-0.445,-0.167) |
| Botswana | 0.067 (0.048,0.09) | 12.025 (8.679,15.78) | 0.125 (0.094,0.163) | 8.586 (6.53,11.122) | -1.079 (-1.145,-1.022) | 0.069 (0.05,0.093) | 12.836 (9.278,16.853) | 0.129 (0.096,0.168) | 9.223 (7.013,11.947) | -1.055 (-1.117,-1.000) | 2.011 (1.436,2.755) | 330.63 (236.544,447.496) | 3.66 (2.69,4.834) | 226.602 (168.226,296.056) | -1.217 (-1.294,-1.154) |
| Brazil | 6.021 (5.776,6.196) | 6.715 (6.389,6.925) | 12.533 (11.855,13.069) | 4.928 (4.655,5.144) | -0.970 (-1.028,-0.901) | 6.225 (5.97,6.41) | 7.136 (6.772,7.369) | 12.87 (12.15,13.441) | 5.093 (4.802,5.322) | -1.050 (-1.109,-0.979) | 175.845 (170.378,180.735) | 183.522 (176.815,188.736) | 342.23 (325.427,356.455) | 132.78 (126.26,138.348) | -1.066 (-1.125,-1.014) |
| Brunei Darussalam | 0.003 (0.002,0.004) | 3.145 (2.496,3.817) | 0.008 (0.006,0.009) | 2.26 (1.806,2.733) | -1.160 (-1.280,-1.058) | 0.003 (0.002,0.004) | 3.202 (2.55,3.863) | 0.007 (0.006,0.008) | 2.145 (1.72,2.604) | -1.351 (-1.464,-1.250) | 0.079 (0.063,0.097) | 75.663 (59.977,92.821) | 0.192 (0.152,0.235) | 50.185 (40.086,61.351) | -1.370 (-1.479,-1.274) |
| Bulgaria | 0.307 (0.286,0.331) | 2.506 (2.347,2.691) | 0.295 (0.253,0.341) | 2.258 (1.929,2.603) | -0.438 (-0.584,-0.236) | 0.318 (0.296,0.343) | 2.633 (2.458,2.828) | 0.307 (0.263,0.352) | 2.302 (1.977,2.633) | -0.542 (-0.701,-0.317) | 8.662 (8.066,9.318) | 70.228 (65.638,75.443) | 7.725 (6.591,8.877) | 62.878 (53.549,72.666) | -0.432 (-0.579,-0.238) |
| Burkina Faso | 0.138 (0.105,0.173) | 3.224 (2.466,4.008) | 0.498 (0.347,0.639) | 5.438 (3.783,7.003) | 1.728 (1.694,1.758) | 0.145 (0.111,0.181) | 3.478 (2.698,4.311) | 0.522 (0.362,0.672) | 5.87 (4.101,7.557) | 1.720 (1.667,1.768) | 3.946 (2.982,4.955) | 86.246 (65.399,107.775) | 14.271 (9.912,18.428) | 143.938 (99.997,185.19) | 1.674 (1.640,1.704) |
| Burundi | 0.445 (0.321,0.558) | 19.068 (13.761,23.801) | 0.516 (0.37,0.67) | 10.639 (7.725,13.647) | -1.874 (-1.907,-1.841) | 0.463 (0.334,0.581) | 20.273 (14.649,25.337) | 0.534 (0.384,0.693) | 11.501 (8.293,14.727) | -1.821 (-1.853,-1.788) | 13.277 (9.417,16.824) | 536.743 (383.167,675.732) | 15.546 (11.159,20.454) | 285.032 (205.132,369.839) | -2.042 (-2.075,-2.007) |
| Cabo Verde | 0.02 (0.016,0.024) | 8.687 (7.209,10.291) | 0.067 (0.053,0.083) | 15.154 (11.901,18.905) | 1.845 (1.580,2.111) | 0.022 (0.018,0.026) | 9.388 (7.751,11.111) | 0.07 (0.055,0.086) | 15.927 (12.58,19.705) | 1.755 (1.488,2.021) | 0.503 (0.423,0.593) | 226.309 (191.194,266.422) | 1.856 (1.451,2.296) | 398.292 (311.281,491.881) | 1.877 (1.656,2.094) |
| Cambodia | 0.196 (0.151,0.247) | 4.23 (3.284,5.278) | 0.368 (0.268,0.486) | 2.96 (2.174,3.874) | -1.150 (-1.163,-1.135) | 0.201 (0.156,0.253) | 4.485 (3.462,5.553) | 0.375 (0.276,0.494) | 3.121 (2.318,4.074) | -1.155 (-1.171,-1.139) | 5.979 (4.579,7.537) | 118.874 (92.046,149.466) | 10.455 (7.574,13.84) | 77.764 (56.888,102.653) | -1.367 (-1.382,-1.349) |
| Cameroon | 0.156 (0.119,0.197) | 3.534 (2.731,4.411) | 0.798 (0.538,1.099) | 6.273 (4.329,8.521) | 1.896 (1.865,1.927) | 0.163 (0.124,0.205) | 3.801 (2.97,4.739) | 0.822 (0.56,1.14) | 6.709 (4.672,9.118) | 1.879 (1.848,1.911) | 4.57 (3.429,5.81) | 95.069 (72.194,119.957) | 23.887 (16.062,33.363) | 169.45 (115.013,235.516) | 1.894 (1.867,1.920) |
| Canada | 1.228 (1.154,1.302) | 3.787 (3.561,4.01) | 2.994 (2.743,3.214) | 4.199 (3.868,4.506) | 0.366 (0.223,0.481) | 1.147 (1.076,1.216) | 3.53 (3.314,3.741) | 2.611 (2.381,2.809) | 3.553 (3.271,3.818) | 0.024 (-0.142,0.156) | 26.66 (25.186,28.17) | 83.426 (78.874,88.062) | 55.597 (51.417,59.559) | 82.266 (76.514,88.311) | -0.024 (-0.171,0.095) |
| Central African Republic | 0.165 (0.115,0.204) | 13.805 (9.729,16.941) | 0.249 (0.167,0.343) | 10.716 (7.358,14.495) | -0.777 (-0.803,-0.751) | 0.168 (0.117,0.207) | 14.659 (10.374,17.777) | 0.252 (0.169,0.348) | 11.46 (7.835,15.354) | -0.752 (-0.780,-0.726) | 5.169 (3.553,6.452) | 392.403 (274.193,483.739) | 7.963 (5.243,11.169) | 299.914 (203.481,413.698) | -0.832 (-0.861,-0.806) |
| Chad | 0.071 (0.057,0.086) | 2.529 (2.051,3.072) | 0.314 (0.229,0.414) | 5.442 (4.002,7.044) | 2.554 (2.521,2.582) | 0.075 (0.061,0.091) | 2.725 (2.216,3.304) | 0.327 (0.243,0.428) | 5.852 (4.388,7.489) | 2.547 (2.515,2.575) | 1.969 (1.587,2.398) | 67.976 (54.727,82.531) | 9.244 (6.676,12.289) | 146.168 (107.522,192.432) | 2.560 (2.524,2.595) |
| Chile | 0.717 (0.678,0.753) | 7.417 (6.989,7.799) | 0.8 (0.724,0.862) | 3.073 (2.787,3.312) | -2.820 (-2.913,-2.732) | 0.77 (0.726,0.811) | 8.118 (7.641,8.554) | 0.833 (0.75,0.9) | 3.185 (2.873,3.436) | -2.997 (-3.074,-2.919) | 17.478 (16.577,18.351) | 173.878 (164.805,182.723) | 16.435 (15.099,17.667) | 63.987 (58.763,68.778) | -3.178 (-3.284,-3.094) |
| China | 207.495 (172.674,241.459) | 24.802 (20.709,28.731) | 320.805 (256.102,394.756) | 15.043 (12.036,18.429) | -1.601 (-1.665,-1.545) | 210.821 (176.081,244.587) | 26.061 (21.773,30.102) | 296.443 (236.648,362.831) | 14.131 (11.356,17.183) | -1.980 (-2.039,-1.927) | 5852.132 (4841.614,6818.927) | 653.309 (543.185,758.879) | 6898.666 (5471.181,8553.366) | 317.182 (252.465,392.419) | -2.311 (-2.370,-2.257) |
| Colombia | 0.684 (0.647,0.716) | 4.065 (3.827,4.281) | 0.973 (0.81,1.157) | 1.766 (1.468,2.1) | -2.950 (-3.181,-2.693) | 0.728 (0.688,0.766) | 4.448 (4.18,4.688) | 1.041 (0.861,1.239) | 1.884 (1.56,2.247) | -3.038 (-3.255,-2.776) | 18.456 (17.617,19.346) | 101.207 (96.244,106.157) | 22.601 (18.701,27.25) | 40.93 (33.945,49.21) | -3.156 (-3.377,-2.888) |
| Comoros | 0.033 (0.023,0.044) | 16.608 (11.979,21.698) | 0.061 (0.045,0.082) | 12.497 (9.285,16.789) | -0.961 (-1.036,-0.882) | 0.034 (0.024,0.046) | 17.738 (12.843,23.238) | 0.063 (0.047,0.085) | 13.467 (10.007,18.11) | -0.903 (-0.986,-0.823) | 0.996 (0.696,1.331) | 458.771 (323.171,611.408) | 1.729 (1.265,2.336) | 329.862 (242.641,444.83) | -1.110 (-1.227,-0.990) |
| Congo | 0.17 (0.126,0.224) | 15.68 (11.825,20.387) | 0.287 (0.202,0.391) | 10.484 (7.653,14.003) | -1.280 (-1.322,-1.221) | 0.174 (0.131,0.229) | 16.702 (12.724,21.645) | 0.293 (0.207,0.398) | 11.264 (8.254,14.923) | -1.249 (-1.288,-1.198) | 5.152 (3.778,6.814) | 439.789 (325.797,577.559) | 8.758 (6.05,12.181) | 281.501 (198.908,380.863) | -1.423 (-1.466,-1.373) |
| Cook Islands | 0 (0,0) | 2.938 (2.425,3.526) | 0.001 (0.001,0.001) | 2.536 (1.995,3.086) | -0.472 (-0.573,-0.396) | 0 (0,0) | 3.168 (2.622,3.79) | 0.001 (0.001,0.001) | 2.545 (2.006,3.058) | -0.708 (-0.807,-0.629) | 0.009 (0.008,0.012) | 73.564 (59.531,89.234) | 0.015 (0.012,0.019) | 59.743 (46.198,74.297) | -0.665 (-0.756,-0.598) |
| Costa Rica | 0.042 (0.039,0.045) | 2.471 (2.279,2.651) | 0.086 (0.075,0.096) | 1.564 (1.365,1.749) | -1.712 (-1.897,-1.536) | 0.045 (0.041,0.048) | 2.653 (2.444,2.844) | 0.09 (0.078,0.101) | 1.632 (1.419,1.826) | -1.619 (-1.931,-1.385) | 1.059 (0.979,1.132) | 59.941 (55.433,64.07) | 2.033 (1.769,2.292) | 36.832 (32.088,41.46) | -1.907 (-2.100,-1.721) |
| Côte d'Ivoire | 0.051 (0.041,0.062) | 1.292 (1.061,1.542) | 0.146 (0.107,0.198) | 1.3 (0.986,1.729) | 1.077 (0.893,1.194) | 0.053 (0.042,0.064) | 1.389 (1.154,1.652) | 0.15 (0.111,0.202) | 1.393 (1.061,1.844) | 0.861 (0.703,0.974) | 1.56 (1.224,1.914) | 34.79 (27.836,42.077) | 4.37 (3.16,6.061) | 34.696 (25.715,46.773) | 1.199 (1.020,1.316) |
| Croatia | 0.234 (0.218,0.251) | 3.762 (3.511,4.012) | 0.226 (0.196,0.256) | 2.675 (2.333,3.026) | 0.052 (-0.041,0.118) | 0.235 (0.218,0.252) | 3.813 (3.562,4.08) | 0.222 (0.193,0.25) | 2.578 (2.241,2.913) | -0.486 (-0.588,-0.408) | 6.477 (5.993,6.971) | 101.286 (94.127,108.65) | 5.249 (4.582,5.939) | 66.803 (58.142,75.871) | -0.194 (-0.280,-0.133) |
| Cuba | 0.377 (0.357,0.399) | 3.708 (3.517,3.92) | 0.973 (0.83,1.122) | 4.968 (4.218,5.715) | 0.438 (0.278,0.564) | 0.399 (0.378,0.422) | 3.95 (3.742,4.171) | 0.98 (0.831,1.135) | 4.954 (4.203,5.75) | 0.190 (-0.033,0.353) | 9.414 (8.929,9.945) | 92.2 (87.437,97.36) | 24.758 (20.904,28.812) | 128.612 (108.42,149.644) | -0.022 (-0.204,0.115) |
| Cyprus | 0.01 (0.008,0.013) | 1.43 (1.164,1.746) | 0.03 (0.023,0.038) | 1.47 (1.149,1.845) | 0.024 (-0.016,0.057) | 0.011 (0.009,0.013) | 1.556 (1.258,1.901) | 0.028 (0.022,0.035) | 1.367 (1.079,1.718) | 0.015 (-0.027,0.050) | 0.243 (0.196,0.298) | 31.562 (25.724,38.376) | 0.598 (0.465,0.765) | 29.768 (23.229,37.839) | -0.002 (-0.044,0.032) |
| Czechia | 0.387 (0.354,0.424) | 2.851 (2.608,3.124) | 0.663 (0.566,0.761) | 3.228 (2.751,3.71) | -1.076 (-1.307,-0.871) | 0.395 (0.362,0.434) | 2.9 (2.652,3.187) | 0.632 (0.542,0.727) | 3.022 (2.585,3.48) | -1.240 (-1.502,-1.022) | 10.312 (9.45,11.267) | 77.902 (71.246,85.327) | 14.92 (12.685,17.272) | 77.308 (65.689,89.431) | -1.349 (-1.666,-1.070) |
| Democratic People's Republic of Korea | 1.722 (1.232,2.303) | 10.493 (7.669,13.717) | 3.102 (2.304,4.093) | 9.19 (6.858,12.049) | -0.425 (-0.434,-0.416) | 1.745 (1.252,2.317) | 10.988 (8.107,14.308) | 3.046 (2.26,4.014) | 9.132 (6.804,11.982) | -0.586 (-0.595,-0.577) | 50.293 (35.683,68.123) | 283.369 (203.891,378.618) | 83.332 (60.441,111.996) | 241.082 (176.36,322.408) | -0.513 (-0.523,-0.505) |
| Democratic Republic of the Congo | 1.505 (1.045,1.999) | 9.63 (6.882,12.615) | 2.909 (1.971,3.907) | 7.962 (5.477,10.738) | -0.597 (-0.643,-0.549) | 1.552 (1.081,2.079) | 10.368 (7.44,13.652) | 2.991 (2.016,4.074) | 8.578 (5.911,11.647) | -0.596 (-0.639,-0.551) | 45.474 (31.044,61.628) | 262.819 (183.46,350.304) | 87.852 (58.113,121.144) | 213.24 (143.543,291.293) | -0.662 (-0.709,-0.612) |
| Denmark | 0.374 (0.355,0.396) | 4.819 (4.569,5.083) | 0.595 (0.538,0.64) | 5.097 (4.643,5.462) | 0.257 (0.114,0.400) | 0.383 (0.363,0.406) | 4.844 (4.585,5.118) | 0.566 (0.508,0.611) | 4.701 (4.256,5.054) | -0.063 (-0.199,0.081) | 8.968 (8.485,9.469) | 121.956 (115.497,128.492) | 11.68 (10.641,12.53) | 106.775 (97.8,114.862) | -0.366 (-0.505,-0.211) |
| Djibouti | 0.02 (0.014,0.029) | 14.72 (10.593,19.929) | 0.078 (0.051,0.114) | 12.112 (8.24,17.112) | -0.649 (-0.682,-0.617) | 0.021 (0.015,0.029) | 15.758 (11.489,21.075) | 0.079 (0.052,0.115) | 13.055 (8.887,18.523) | -0.624 (-0.654,-0.594) | 0.641 (0.441,0.909) | 401.325 (283.992,552.418) | 2.364 (1.53,3.493) | 320.565 (213.23,464.52) | -0.750 (-0.789,-0.710) |
| Dominica | 0.003 (0.002,0.003) | 4.83 (4.167,5.68) | 0.004 (0.003,0.005) | 4.911 (3.948,6.273) | 0.067 (0.029,0.094) | 0.003 (0.003,0.004) | 5.239 (4.522,6.186) | 0.004 (0.004,0.006) | 5.236 (4.241,6.605) | 0.012 (-0.019,0.036) | 0.071 (0.061,0.084) | 122.57 (104.64,144.721) | 0.11 (0.087,0.145) | 127.813 (101.27,167.103) | 0.138 (0.097,0.170) |
| Dominican Republic | 0.068 (0.055,0.083) | 1.891 (1.556,2.289) | 0.218 (0.165,0.282) | 2.172 (1.635,2.798) | 0.538 (0.448,0.612) | 0.072 (0.058,0.087) | 2.063 (1.694,2.476) | 0.229 (0.173,0.295) | 2.299 (1.737,2.962) | 0.577 (0.415,0.743) | 1.952 (1.585,2.374) | 49.946 (40.42,60.748) | 6.008 (4.529,7.753) | 58.465 (43.94,75.313) | 0.630 (0.545,0.703) |
| Ecuador | 0.103 (0.097,0.109) | 2.034 (1.899,2.156) | 0.191 (0.15,0.239) | 1.191 (0.939,1.49) | -1.651 (-1.847,-1.473) | 0.112 (0.105,0.119) | 2.265 (2.11,2.407) | 0.209 (0.166,0.261) | 1.324 (1.051,1.642) | -1.639 (-1.839,-1.452) | 2.711 (2.55,2.856) | 50.076 (46.981,52.82) | 4.496 (3.509,5.632) | 27.394 (21.398,34.224) | -1.919 (-2.116,-1.741) |
| Egypt | 0.309 (0.27,0.361) | 1.184 (1.023,1.428) | 0.611 (0.478,0.775) | 1.035 (0.821,1.299) | -0.377 (-0.471,-0.275) | 0.317 (0.278,0.374) | 1.288 (1.109,1.566) | 0.619 (0.486,0.783) | 1.11 (0.884,1.394) | -0.403 (-0.500,-0.299) | 9.419 (8.249,10.874) | 31.362 (27.388,36.695) | 17.043 (13.29,21.443) | 25.33 (19.971,31.926) | -0.676 (-0.775,-0.575) |
| El Salvador | 0.054 (0.048,0.06) | 1.83 (1.625,2.045) | 0.105 (0.084,0.132) | 1.685 (1.343,2.134) | -0.239 (-0.498,-0.064) | 0.058 (0.051,0.065) | 1.997 (1.766,2.238) | 0.112 (0.089,0.141) | 1.782 (1.419,2.242) | -0.349 (-0.593,-0.176) | 1.458 (1.306,1.621) | 47.605 (42.67,52.964) | 2.6 (2.072,3.294) | 42.499 (33.811,53.923) | -0.348 (-0.602,-0.175) |
| Equatorial Guinea | 0.026 (0.018,0.035) | 13.15 (9.282,17.189) | 0.044 (0.03,0.063) | 8.779 (6.126,12.5) | -1.253 (-1.344,-1.188) | 0.027 (0.019,0.036) | 13.966 (10.013,18.158) | 0.045 (0.031,0.065) | 9.423 (6.554,13.413) | -1.220 (-1.306,-1.158) | 0.805 (0.553,1.062) | 371.128 (257.164,487.553) | 1.314 (0.87,1.938) | 229.685 (155.889,333.53) | -1.496 (-1.587,-1.426) |
| Eritrea | 0.247 (0.16,0.33) | 20.189 (13.463,26.414) | 0.402 (0.279,0.58) | 14.471 (10.202,20.818) | -1.082 (-1.110,-1.053) | 0.248 (0.162,0.328) | 21.359 (14.306,27.899) | 0.41 (0.283,0.594) | 15.6 (10.929,22.482) | -1.021 (-1.048,-0.993) | 8.111 (5.216,10.825) | 578.217 (380.206,764.919) | 12.51 (8.539,18.171) | 391.027 (270.149,566.88) | -1.274 (-1.307,-1.240) |
| Estonia | 0.068 (0.064,0.073) | 3.319 (3.087,3.56) | 0.07 (0.059,0.08) | 2.75 (2.347,3.179) | -0.485 (-0.728,-0.245) | 0.07 (0.065,0.075) | 3.377 (3.147,3.616) | 0.067 (0.057,0.077) | 2.584 (2.211,2.989) | -0.749 (-1.018,-0.487) | 1.877 (1.723,2.024) | 91.85 (84.924,98.884) | 1.554 (1.325,1.814) | 65.919 (56.237,77.003) | -0.958 (-1.202,-0.696) |
| Eswatini | 0.048 (0.036,0.061) | 16.402 (12.256,20.695) | 0.099 (0.069,0.137) | 16.684 (11.804,22.424) | 0.106 (0.045,0.170) | 0.049 (0.037,0.063) | 17.387 (12.95,21.762) | 0.1 (0.07,0.139) | 17.472 (12.429,23.385) | 0.091 (0.032,0.150) | 1.464 (1.084,1.887) | 457.534 (339.532,581.335) | 3.105 (2.112,4.387) | 478.853 (332.559,665.205) | 0.163 (0.099,0.227) |
| Ethiopia | 1.903 (1.333,2.45) | 9.423 (6.651,12.213) | 2.105 (1.689,2.804) | 4.972 (4.013,6.658) | -2.039 (-2.059,-2.018) | 1.959 (1.375,2.518) | 10.061 (7.104,13.027) | 2.208 (1.784,2.947) | 5.385 (4.354,7.224) | -2.003 (-2.027,-1.981) | 58.05 (40.276,74.818) | 262.775 (183.928,339.388) | 60.063 (47.899,80.044) | 129.6 (104.332,172.946) | -2.254 (-2.274,-2.232) |
| Fiji | 0.008 (0.006,0.01) | 2.264 (1.808,2.829) | 0.019 (0.013,0.025) | 2.625 (1.877,3.416) | 0.411 (0.262,0.523) | 0.008 (0.006,0.01) | 2.461 (1.968,3.066) | 0.019 (0.014,0.025) | 2.86 (2.012,3.724) | 0.537 (0.374,0.715) | 0.235 (0.186,0.299) | 59.346 (47.162,74.886) | 0.532 (0.376,0.704) | 66.243 (46.717,86.765) | 0.290 (0.136,0.410) |
| Finland | 0.261 (0.245,0.274) | 3.688 (3.469,3.87) | 0.441 (0.401,0.474) | 3.617 (3.32,3.863) | 0.046 (-0.055,0.138) | 0.238 (0.223,0.251) | 3.345 (3.139,3.525) | 0.361 (0.327,0.389) | 2.821 (2.583,3.02) | -0.524 (-0.621,-0.432) | 5.457 (5.198,5.711) | 79.659 (75.909,83.409) | 7.368 (6.793,7.881) | 66.179 (61.739,70.664) | -0.572 (-0.691,-0.438) |
| France | 6.523 (6.119,6.913) | 8.425 (7.917,8.938) | 6.594 (5.937,7.184) | 4.988 (4.537,5.412) | -1.620 (-1.734,-1.538) | 6.28 (5.891,6.664) | 7.961 (7.481,8.429) | 5.108 (4.568,5.571) | 3.652 (3.302,3.974) | -2.399 (-2.529,-2.315) | 159.851 (150.364,169.864) | 216.096 (203.455,229.798) | 107.529 (97.129,117.304) | 88.638 (80.733,95.955) | -2.789 (-2.867,-2.728) |
| Gabon | 0.07 (0.053,0.088) | 12.32 (9.229,15.451) | 0.11 (0.08,0.142) | 10.462 (7.647,13.414) | -0.457 (-0.504,-0.411) | 0.074 (0.055,0.092) | 13.186 (9.872,16.497) | 0.114 (0.082,0.147) | 11.197 (8.243,14.385) | -0.456 (-0.501,-0.412) | 1.999 (1.486,2.542) | 337.158 (251.217,426.967) | 3.209 (2.281,4.263) | 277.424 (199.591,360.92) | -0.583 (-0.631,-0.525) |
| Gambia | 0.005 (0.004,0.007) | 1.495 (1.161,1.88) | 0.019 (0.014,0.024) | 1.931 (1.488,2.432) | 0.956 (0.800,1.130) | 0.005 (0.004,0.007) | 1.605 (1.251,2.017) | 0.019 (0.015,0.025) | 2.066 (1.585,2.619) | 0.943 (0.796,1.109) | 0.151 (0.114,0.194) | 40.257 (30.78,50.988) | 0.539 (0.407,0.697) | 51.538 (38.881,65.798) | 0.932 (0.785,1.113) |
| Georgia | 0.133 (0.116,0.152) | 2.095 (1.829,2.402) | 0.063 (0.053,0.074) | 1.06 (0.89,1.248) | -2.250 (-2.497,-2.023) | 0.14 (0.122,0.161) | 2.229 (1.938,2.563) | 0.068 (0.057,0.08) | 1.13 (0.959,1.33) | -2.252 (-2.495,-2.022) | 3.628 (3.162,4.156) | 56.364 (49.214,64.466) | 1.576 (1.339,1.849) | 27.433 (23.329,32.095) | -2.306 (-2.556,-2.098) |
| Germany | 4.309 (4.076,4.554) | 3.58 (3.386,3.788) | 8.29 (7.57,8.901) | 4.597 (4.209,4.912) | 0.910 (0.758,1.022) | 4.09 (3.869,4.314) | 3.345 (3.159,3.534) | 6.949 (6.331,7.477) | 3.686 (3.391,3.933) | 0.405 (0.282,0.504) | 107.035 (100.726,113.37) | 92.317 (86.777,98.157) | 152.265 (140.345,162.754) | 90.655 (84.219,96.824) | 0.025 (-0.120,0.149) |
| Ghana | 0.15 (0.115,0.19) | 2.413 (1.867,3.024) | 0.572 (0.387,0.737) | 3.511 (2.333,4.553) | 1.216 (1.170,1.250) | 0.155 (0.12,0.198) | 2.593 (2.012,3.263) | 0.595 (0.405,0.772) | 3.795 (2.533,4.966) | 1.238 (1.196,1.270) | 4.454 (3.369,5.776) | 65.058 (49.678,83.146) | 16.371 (11.321,21.334) | 90.518 (61.708,117.852) | 1.070 (1.027,1.104) |
| Global | 354.731 (317.512,388.914) | 8.857 (7.962,9.694) | 576.529 (509.492,645.648) | 6.655 (5.883,7.45) | -0.940 (-0.982,-0.906) | 356.263 (319.363,390.154) | 9.022 (8.112,9.866) | 538.602 (475.944,603.406) | 6.255 (5.527,7.003) | -1.197 (-1.241,-1.162) | 9753.566 (8719.319,10739.561) | 235.319 (210.517,258.681) | 12999.265 (11522.861,14605.268) | 148.561 (131.709,166.818) | -1.500 (-1.543,-1.466) |
| Greece | 0.345 (0.324,0.364) | 2.249 (2.109,2.371) | 0.409 (0.373,0.435) | 1.817 (1.689,1.928) | -0.634 (-0.737,-0.538) | 0.348 (0.325,0.367) | 2.268 (2.125,2.394) | 0.399 (0.362,0.426) | 1.664 (1.541,1.762) | -0.986 (-1.117,-0.855) | 7.438 (7.023,7.844) | 49.29 (46.575,51.862) | 8.176 (7.591,8.674) | 40.62 (38.215,43.135) | -0.541 (-0.642,-0.442) |
| Greenland | 0.006 (0.005,0.007) | 16.306 (14.01,18.847) | 0.008 (0.006,0.01) | 10.808 (8.592,13.414) | -1.213 (-1.267,-1.164) | 0.006 (0.005,0.007) | 17.009 (14.655,19.719) | 0.008 (0.006,0.01) | 11.002 (8.737,13.718) | -1.303 (-1.360,-1.251) | 0.17 (0.144,0.198) | 437.483 (374.319,506.614) | 0.211 (0.167,0.258) | 272.77 (217.033,334.957) | -1.362 (-1.524,-1.271) |
| Grenada | 0.004 (0.004,0.005) | 6.412 (5.728,7.127) | 0.005 (0.005,0.006) | 4.628 (3.925,5.322) | -1.161 (-1.810,-0.751) | 0.005 (0.004,0.005) | 6.782 (6.079,7.552) | 0.006 (0.005,0.007) | 4.844 (4.118,5.598) | -1.157 (-1.753,-0.754) | 0.115 (0.103,0.128) | 176.761 (157.847,197.804) | 0.15 (0.126,0.177) | 122.775 (103.704,143.293) | -1.398 (-1.993,-1.028) |
| Guam | 0.002 (0.001,0.002) | 2.322 (2.002,2.641) | 0.004 (0.003,0.005) | 1.883 (1.581,2.219) | -0.802 (-1.148,-0.520) | 0.002 (0.001,0.002) | 2.492 (2.138,2.829) | 0.004 (0.003,0.005) | 1.839 (1.545,2.166) | -1.076 (-1.329,-0.873) | 0.047 (0.04,0.054) | 57.083 (49.272,65.264) | 0.107 (0.089,0.126) | 51.705 (43.221,60.849) | -0.387 (-0.646,-0.148) |
| Guatemala | 0.079 (0.076,0.083) | 2.556 (2.453,2.664) | 0.177 (0.151,0.21) | 1.649 (1.413,1.952) | -1.447 (-1.632,-1.203) | 0.085 (0.081,0.088) | 2.893 (2.77,3.015) | 0.191 (0.163,0.223) | 1.815 (1.565,2.119) | -1.465 (-1.649,-1.233) | 2.283 (2.193,2.375) | 63.302 (60.726,65.798) | 4.583 (3.881,5.387) | 40.648 (34.541,47.758) | -1.455 (-1.648,-1.194) |
| Guinea | 0.04 (0.031,0.049) | 1.197 (0.932,1.465) | 0.075 (0.053,0.101) | 1.343 (0.958,1.804) | 0.389 (0.357,0.413) | 0.042 (0.033,0.052) | 1.287 (1.011,1.581) | 0.079 (0.056,0.107) | 1.442 (1.033,1.938) | 0.385 (0.353,0.408) | 1.11 (0.871,1.374) | 32.306 (25.389,39.969) | 2.162 (1.522,2.954) | 36.042 (25.477,49.303) | 0.369 (0.336,0.394) |
| Guinea-Bissau | 0.019 (0.014,0.024) | 4.711 (3.547,5.867) | 0.054 (0.04,0.068) | 7.307 (5.448,9.116) | 1.436 (1.404,1.465) | 0.02 (0.015,0.025) | 5.01 (3.782,6.233) | 0.055 (0.041,0.07) | 7.802 (5.811,9.721) | 1.478 (1.443,1.509) | 0.572 (0.419,0.726) | 131.96 (97.579,166.911) | 1.678 (1.235,2.154) | 201.128 (149.664,253.612) | 1.380 (1.349,1.410) |
| Guyana | 0.008 (0.007,0.009) | 2.096 (1.872,2.335) | 0.014 (0.01,0.018) | 2.036 (1.561,2.619) | 0.114 (-0.106,0.335) | 0.008 (0.007,0.009) | 2.24 (1.997,2.482) | 0.014 (0.011,0.018) | 2.145 (1.632,2.736) | 0.050 (-0.154,0.242) | 0.231 (0.205,0.26) | 57.26 (50.981,64.152) | 0.403 (0.303,0.523) | 57.175 (43.2,73.708) | 0.235 (0.008,0.472) |
| Haiti | 0.169 (0.12,0.221) | 5.234 (3.804,6.745) | 0.284 (0.196,0.4) | 3.946 (2.778,5.496) | -0.848 (-0.882,-0.814) | 0.175 (0.125,0.228) | 5.632 (4.124,7.211) | 0.294 (0.206,0.415) | 4.258 (3.023,5.884) | -0.872 (-0.910,-0.840) | 5.032 (3.589,6.623) | 143.741 (102.626,187.876) | 8.475 (5.894,12.126) | 106.31 (74.133,150.331) | -0.914 (-0.947,-0.880) |
| Honduras | 0.02 (0.016,0.024) | 1.004 (0.83,1.189) | 0.087 (0.068,0.111) | 1.452 (1.137,1.828) | 1.246 (1.183,1.301) | 0.021 (0.018,0.025) | 1.101 (0.913,1.299) | 0.094 (0.074,0.119) | 1.604 (1.263,1.998) | 1.273 (1.211,1.326) | 0.565 (0.464,0.675) | 26.181 (21.555,31.229) | 2.268 (1.767,2.938) | 35.29 (27.377,45.125) | 1.009 (0.946,1.069) |
| Hungary | 0.594 (0.55,0.637) | 4.204 (3.899,4.503) | 0.57 (0.503,0.645) | 3.172 (2.799,3.586) | -1.017 (-1.130,-0.887) | 0.604 (0.56,0.647) | 4.257 (3.951,4.551) | 0.583 (0.515,0.66) | 3.186 (2.811,3.603) | -1.032 (-1.138,-0.916) | 17.829 (16.519,19.166) | 128.985 (119.336,138.425) | 14.801 (13.071,16.774) | 87.025 (76.548,98.845) | -1.405 (-1.517,-1.290) |
| Iceland | 0.013 (0.012,0.014) | 4.711 (4.368,5.028) | 0.026 (0.023,0.029) | 4.532 (4.037,5.054) | -0.103 (-0.227,-0.019) | 0.013 (0.012,0.014) | 4.528 (4.194,4.823) | 0.023 (0.02,0.025) | 3.846 (3.409,4.274) | -0.361 (-0.495,-0.234) | 0.295 (0.274,0.315) | 107.793 (100.127,115.442) | 0.485 (0.435,0.539) | 89.549 (80.216,99.094) | -0.564 (-0.678,-0.483) |
| India | 16.821 (14.654,20.952) | 3.488 (3.019,4.383) | 37.007 (32.443,44.293) | 3.071 (2.695,3.692) | -0.414 (-0.461,-0.362) | 17.155 (14.895,21.464) | 3.685 (3.178,4.652) | 38.002 (33.296,45.562) | 3.227 (2.834,3.888) | -0.430 (-0.503,-0.339) | 525.762 (458.717,653.744) | 99.406 (86.451,124.395) | 1056.497 (927.426,1250.177) | 83.405 (73.108,99.107) | -0.572 (-0.622,-0.519) |
| Indonesia | 1.844 (1.369,2.236) | 1.843 (1.381,2.243) | 3.847 (2.949,4.855) | 1.621 (1.25,2.027) | -0.421 (-0.444,-0.401) | 1.882 (1.401,2.289) | 1.952 (1.468,2.386) | 3.889 (2.981,4.885) | 1.714 (1.323,2.142) | -0.428 (-0.451,-0.408) | 56.368 (41.756,67.914) | 50.905 (37.893,61.798) | 108.597 (83.024,136.774) | 41.436 (31.706,52.061) | -0.671 (-0.693,-0.652) |
| Iran  (Islamic Republic of) | 1.222 (1.049,1.367) | 5.023 (4.277,5.64) | 2.791 (2.528,3.035) | 3.775 (3.396,4.111) | -0.967 (-1.042,-0.909) | 1.263 (1.085,1.414) | 5.469 (4.646,6.143) | 2.87 (2.595,3.129) | 3.989 (3.58,4.352) | -1.061 (-1.137,-0.997) | 34.925 (29.951,39.099) | 126.689 (108.343,141.56) | 68.678 (62.63,74.957) | 86.706 (78.896,94.509) | -1.268 (-1.359,-1.182) |
| Iraq | 0.094 (0.072,0.118) | 1.184 (0.916,1.489) | 0.274 (0.197,0.354) | 1.179 (0.873,1.51) | 0.012 (-0.033,0.065) | 0.097 (0.076,0.122) | 1.246 (0.976,1.564) | 0.274 (0.198,0.352) | 1.23 (0.924,1.563) | -0.007 (-0.055,0.049) | 2.694 (2.088,3.415) | 32.278 (25.033,40.91) | 7.51 (5.368,9.849) | 29.088 (20.939,37.444) | -0.326 (-0.381,-0.271) |
| Ireland | 0.316 (0.297,0.338) | 7.768 (7.292,8.282) | 0.486 (0.43,0.536) | 6.135 (5.451,6.738) | -0.796 (-1.039,-0.634) | 0.323 (0.302,0.345) | 7.92 (7.4,8.438) | 0.435 (0.382,0.481) | 5.391 (4.763,5.956) | -1.257 (-1.423,-1.142) | 7.142 (6.728,7.61) | 179.704 (169.366,191.239) | 9.184 (8.234,10.069) | 120.154 (108.166,131.851) | -1.313 (-1.450,-1.208) |
| Israel | 0.096 (0.089,0.103) | 1.996 (1.854,2.133) | 0.19 (0.171,0.206) | 1.544 (1.406,1.668) | -0.789 (-0.903,-0.678) | 0.1 (0.093,0.108) | 2.102 (1.943,2.246) | 0.185 (0.165,0.201) | 1.465 (1.32,1.581) | -1.150 (-1.241,-1.064) | 2.179 (2.044,2.328) | 46.08 (43.356,49.147) | 3.806 (3.476,4.094) | 32.431 (29.735,34.821) | -1.038 (-1.168,-0.900) |
| Italy | 2.91 (2.763,3.012) | 3.336 (3.175,3.45) | 2.51 (2.268,2.658) | 1.773 (1.643,1.867) | -2.044 (-2.124,-1.974) | 2.92 (2.765,3.019) | 3.321 (3.147,3.431) | 2.395 (2.149,2.54) | 1.608 (1.484,1.691) | -2.337 (-2.427,-2.251) | 69.321 (66.589,71.519) | 82.26 (79.189,84.944) | 47.55 (44.308,50.005) | 37.131 (35.078,38.911) | -2.628 (-2.736,-2.553) |
| Jamaica | 0.052 (0.048,0.056) | 2.911 (2.71,3.126) | 0.092 (0.07,0.119) | 2.97 (2.255,3.845) | 0.172 (-0.474,0.802) | 0.056 (0.052,0.06) | 3.125 (2.909,3.355) | 0.097 (0.073,0.124) | 3.127 (2.36,4.011) | 0.103 (-0.512,0.713) | 1.265 (1.178,1.36) | 72.976 (67.923,78.47) | 2.321 (1.715,3.036) | 75.247 (55.67,98.339) | 0.102 (-0.991,0.736) |
| Japan | 11.335 (10.816,11.676) | 6.592 (6.276,6.796) | 21.933 (19.705,23.18) | 6.216 (5.726,6.477) | -0.246 (-0.302,-0.194) | 8.493 (8.105,8.716) | 4.986 (4.734,5.125) | 14.565 (12.957,15.419) | 3.815 (3.49,3.987) | -0.878 (-0.928,-0.838) | 204.567 (197.74,208.797) | 118.072 (113.933,120.6) | 267.046 (244.223,279.928) | 84.343 (78.985,87.574) | -1.098 (-1.168,-1.048) |
| Jordan | 0.014 (0.011,0.018) | 1.116 (0.882,1.369) | 0.061 (0.047,0.079) | 0.853 (0.663,1.079) | -0.871 (-0.973,-0.786) | 0.015 (0.012,0.018) | 1.189 (0.936,1.458) | 0.06 (0.046,0.077) | 0.882 (0.686,1.124) | -0.969 (-1.079,-0.877) | 0.421 (0.328,0.527) | 28.825 (22.638,35.832) | 1.637 (1.244,2.112) | 20.166 (15.53,25.91) | -1.149 (-1.222,-1.069) |
| Kazakhstan | 2.355 (2.188,2.537) | 18.883 (17.507,20.342) | 0.832 (0.712,0.961) | 4.689 (4.038,5.402) | -4.320 (-4.505,-4.098) | 2.49 (2.309,2.686) | 20.318 (18.802,21.936) | 0.868 (0.747,1.005) | 5.021 (4.342,5.799) | -4.413 (-4.663,-4.178) | 65.421 (60.929,69.984) | 500.498 (465.898,537.45) | 22.415 (19.347,25.881) | 119.336 (103.249,137.704) | -4.422 (-4.615,-4.253) |
| Kenya | 0.737 (0.53,1.059) | 8.971 (6.423,12.969) | 2.643 (1.923,3.713) | 11.746 (8.541,16.663) | 0.867 (0.832,0.902) | 0.765 (0.552,1.102) | 9.579 (6.9,13.918) | 2.736 (1.989,3.847) | 12.685 (9.196,17.966) | 0.906 (0.870,0.942) | 21.446 (15.566,30.697) | 242.784 (175.432,348.163) | 76.417 (55.651,107.057) | 306.118 (222.368,429.013) | 0.749 (0.709,0.786) |
| Kiribati | 0.002 (0.002,0.003) | 6.092 (4.804,7.538) | 0.004 (0.003,0.005) | 5.516 (3.95,7.072) | -0.313 (-0.345,-0.280) | 0.002 (0.002,0.003) | 6.566 (5.178,8.196) | 0.004 (0.003,0.005) | 5.962 (4.235,7.638) | -0.299 (-0.327,-0.271) | 0.069 (0.054,0.086) | 168.8 (133.194,209.921) | 0.123 (0.087,0.163) | 150.719 (108.336,197.392) | -0.355 (-0.386,-0.322) |
| Kuwait | 0.011 (0.01,0.012) | 1.82 (1.659,1.984) | 0.026 (0.021,0.031) | 0.938 (0.756,1.149) | -1.908 (-2.459,-1.366) | 0.01 (0.009,0.011) | 1.861 (1.689,2.031) | 0.024 (0.02,0.03) | 0.946 (0.763,1.168) | -1.942 (-2.462,-1.402) | 0.303 (0.279,0.328) | 44.598 (40.956,48.495) | 0.634 (0.51,0.78) | 19.704 (15.891,24.306) | -2.411 (-3.025,-1.784) |
| Kyrgyzstan | 0.248 (0.213,0.284) | 8.27 (7.169,9.441) | 0.132 (0.104,0.164) | 2.819 (2.255,3.438) | -3.382 (-3.511,-3.249) | 0.26 (0.225,0.297) | 8.797 (7.665,9.995) | 0.138 (0.109,0.169) | 3.029 (2.435,3.638) | -3.319 (-3.453,-3.186) | 7.117 (6.112,8.171) | 229.894 (198.381,264.046) | 3.694 (2.886,4.57) | 72.507 (56.986,88.941) | -3.725 (-3.848,-3.601) |
| Lao People's Democratic Republic | 0.093 (0.065,0.126) | 4.345 (3.067,5.825) | 0.115 (0.085,0.161) | 2.481 (1.861,3.419) | -1.809 (-1.826,-1.791) | 0.096 (0.068,0.129) | 4.595 (3.3,6.117) | 0.118 (0.087,0.165) | 2.636 (1.99,3.626) | -1.788 (-1.804,-1.770) | 2.848 (2.026,3.854) | 124.038 (88.6,167.067) | 3.387 (2.451,4.752) | 66.115 (48.581,92.566) | -2.022 (-2.042,-2.002) |
| Latvia | 0.11 (0.102,0.118) | 3.046 (2.823,3.276) | 0.106 (0.091,0.121) | 2.912 (2.496,3.329) | 0.115 (-0.175,0.472) | 0.113 (0.105,0.121) | 3.131 (2.911,3.365) | 0.108 (0.093,0.123) | 2.9 (2.486,3.334) | 0.022 (-0.330,0.384) | 3.069 (2.827,3.305) | 86.008 (79.215,92.716) | 2.669 (2.277,3.078) | 78.33 (66.705,90.886) | -0.397 (-0.651,-0.152) |
| Lebanon | 0.027 (0.021,0.035) | 1.284 (1.006,1.639) | 0.056 (0.044,0.069) | 0.917 (0.729,1.134) | -1.024 (-1.083,-0.971) | 0.028 (0.022,0.036) | 1.365 (1.077,1.734) | 0.058 (0.046,0.07) | 0.935 (0.749,1.142) | -1.163 (-1.230,-1.106) | 0.748 (0.578,0.957) | 32.877 (25.634,41.803) | 1.234 (0.982,1.517) | 20.853 (16.579,25.698) | -1.415 (-1.480,-1.360) |
| Lesotho | 0.086 (0.067,0.11) | 10.204 (7.892,13.05) | 0.176 (0.13,0.225) | 15.821 (11.668,20.121) | 1.509 (1.420,1.581) | 0.091 (0.07,0.116) | 10.918 (8.506,14.017) | 0.181 (0.134,0.234) | 16.666 (12.384,21.26) | 1.468 (1.387,1.540) | 2.447 (1.874,3.151) | 277.578 (213.012,357.188) | 5.308 (3.839,6.988) | 450.011 (327.622,588.489) | 1.613 (1.523,1.686) |
| Liberia | 0.035 (0.027,0.044) | 3.081 (2.439,3.807) | 0.12 (0.082,0.17) | 5.652 (3.967,7.88) | 1.947 (1.889,1.995) | 0.037 (0.029,0.046) | 3.328 (2.655,4.069) | 0.123 (0.085,0.176) | 6.068 (4.245,8.438) | 1.932 (1.878,1.977) | 0.981 (0.761,1.221) | 82.315 (64.483,102.163) | 3.618 (2.463,5.287) | 150.606 (103.606,214.96) | 1.901 (1.821,1.959) |
| Libya | 0.026 (0.019,0.035) | 1.392 (1.02,1.87) | 0.09 (0.063,0.122) | 1.697 (1.199,2.256) | 0.728 (0.617,0.829) | 0.027 (0.02,0.036) | 1.476 (1.08,1.968) | 0.09 (0.063,0.121) | 1.761 (1.253,2.327) | 0.657 (0.535,0.768) | 0.734 (0.529,1.002) | 36.798 (26.808,49.746) | 2.571 (1.756,3.512) | 43.789 (30.39,59.014) | 0.659 (0.567,0.763) |
| Lithuania | 0.145 (0.136,0.155) | 3.187 (2.982,3.41) | 0.195 (0.168,0.223) | 3.682 (3.17,4.212) | 0.225 (-0.040,0.395) | 0.146 (0.137,0.157) | 3.215 (3.016,3.438) | 0.191 (0.164,0.219) | 3.559 (3.057,4.081) | 0.111 (-0.148,0.281) | 3.996 (3.735,4.267) | 88.391 (82.73,94.342) | 4.86 (4.179,5.577) | 97.276 (83.823,111.388) | 0.302 (0.076,0.527) |
| Luxembourg | 0.026 (0.025,0.027) | 4.871 (4.595,5.14) | 0.039 (0.036,0.044) | 3.735 (3.366,4.127) | -0.985 (-1.310,-0.699) | 0.026 (0.025,0.028) | 4.888 (4.615,5.144) | 0.036 (0.033,0.04) | 3.354 (3.018,3.699) | -1.134 (-1.290,-1.009) | 0.642 (0.605,0.679) | 122.961 (115.867,130.073) | 0.8 (0.725,0.885) | 77.995 (70.6,86.249) | -1.470 (-1.648,-1.325) |
| Madagascar | 0.742 (0.547,0.906) | 14.453 (10.705,17.473) | 1.233 (0.85,1.701) | 10.791 (7.474,14.777) | -0.945 (-0.987,-0.902) | 0.769 (0.566,0.934) | 15.404 (11.328,18.623) | 1.257 (0.862,1.727) | 11.594 (7.93,15.785) | -0.920 (-0.961,-0.877) | 22.207 (16.247,27.218) | 401.584 (294.991,489.606) | 38.198 (26.089,52.767) | 291.339 (199.8,398.824) | -1.038 (-1.080,-0.994) |
| Malawi | 0.812 (0.66,0.982) | 21.075 (17.239,25.266) | 1.965 (1.579,2.479) | 26.064 (21.015,32.461) | 0.716 (0.676,0.757) | 0.838 (0.69,1.01) | 22.596 (18.546,27.085) | 2.017 (1.625,2.541) | 27.774 (22.45,34.722) | 0.731 (0.675,0.787) | 24.767 (20.298,29.765) | 584.024 (479.767,700.607) | 59.611 (47.284,76.806) | 715.282 (572.772,904.409) | 0.684 (0.644,0.725) |
| Malaysia | 0.237 (0.198,0.275) | 2.614 (2.184,3.035) | 0.749 (0.636,0.873) | 2.646 (2.248,3.08) | -0.024 (-0.285,0.218) | 0.246 (0.205,0.285) | 2.761 (2.32,3.194) | 0.743 (0.632,0.865) | 2.685 (2.297,3.114) | -0.280 (-0.440,-0.120) | 6.595 (5.524,7.661) | 68.38 (57.279,79.305) | 19.234 (16.301,22.633) | 64.895 (54.963,76.089) | -0.114 (-0.274,0.074) |
| Maldives | 0.003 (0.002,0.004) | 3.037 (2.23,3.874) | 0.004 (0.003,0.005) | 1.207 (0.95,1.475) | -3.054 (-3.158,-2.929) | 0.003 (0.002,0.004) | 3.201 (2.385,4.062) | 0.004 (0.003,0.005) | 1.221 (0.965,1.489) | -3.187 (-3.288,-3.065) | 0.088 (0.061,0.114) | 84.299 (59.822,108.354) | 0.106 (0.081,0.134) | 27.825 (21.732,34.754) | -3.659 (-3.764,-3.531) |
| Mali | 0.096 (0.08,0.114) | 2.414 (2.032,2.832) | 0.214 (0.163,0.276) | 2.446 (1.872,3.125) | 0.071 (0.036,0.101) | 0.1 (0.084,0.118) | 2.593 (2.204,3.03) | 0.223 (0.169,0.289) | 2.632 (2.015,3.39) | 0.097 (0.043,0.136) | 2.822 (2.355,3.356) | 65.48 (55.034,77.259) | 6.241 (4.725,8.158) | 65.075 (49.533,84.489) | 0.006 (-0.031,0.040) |
| Malta | 0.012 (0.011,0.013) | 2.873 (2.656,3.101) | 0.022 (0.02,0.025) | 2.411 (2.15,2.682) | -0.520 (-0.782,-0.290) | 0.012 (0.011,0.013) | 2.924 (2.703,3.152) | 0.021 (0.018,0.023) | 2.172 (1.927,2.414) | -0.940 (-1.199,-0.722) | 0.295 (0.273,0.32) | 68.929 (63.806,74.687) | 0.441 (0.394,0.489) | 52.064 (46.472,57.452) | -0.825 (-1.174,-0.551) |
| Marshall Islands | 0 (0,0.001) | 2.772 (1.941,3.629) | 0.001 (0.001,0.001) | 2.485 (1.862,3.331) | -0.347 (-0.422,-0.243) | 0 (0,0.001) | 2.998 (2.11,3.883) | 0.001 (0.001,0.001) | 2.667 (2.026,3.54) | -0.369 (-0.446,-0.263) | 0.013 (0.009,0.017) | 74.804 (51.586,97.956) | 0.027 (0.019,0.036) | 66.777 (49.641,89.363) | -0.362 (-0.424,-0.273) |
| Mauritania | 0.035 (0.027,0.045) | 3.539 (2.736,4.542) | 0.11 (0.078,0.152) | 5.212 (3.7,7.09) | 1.275 (1.212,1.328) | 0.037 (0.029,0.047) | 3.806 (2.95,4.906) | 0.116 (0.082,0.159) | 5.622 (4.004,7.628) | 1.285 (1.228,1.338) | 0.983 (0.741,1.256) | 94.911 (71.891,121.284) | 2.997 (2.119,4.203) | 132.933 (93.636,184.965) | 1.099 (1.036,1.150) |
| Mauritius | 0.026 (0.025,0.028) | 3.61 (3.416,3.797) | 0.066 (0.061,0.07) | 3.547 (3.295,3.746) | 0.255 (-0.179,0.736) | 0.027 (0.025,0.028) | 3.763 (3.56,3.963) | 0.065 (0.06,0.069) | 3.506 (3.257,3.7) | 0.070 (-0.351,0.575) | 0.741 (0.703,0.78) | 95.648 (90.859,100.537) | 1.738 (1.594,1.838) | 92.681 (85.245,97.679) | 0.238 (-0.220,0.643) |
| Mexico | 0.881 (0.856,0.897) | 2.211 (2.13,2.258) | 1.796 (1.553,2.051) | 1.433 (1.242,1.633) | -1.436 (-1.558,-1.316) | 0.951 (0.923,0.969) | 2.477 (2.381,2.532) | 1.888 (1.64,2.15) | 1.53 (1.331,1.738) | -1.581 (-1.704,-1.456) | 23.542 (23.054,23.947) | 53.72 (52.436,54.733) | 46.852 (40.311,53.726) | 36.045 (31.078,41.266) | -1.354 (-1.456,-1.252) |
| Micronesia  (Federated States of) | 0.001 (0.001,0.002) | 3.15 (2.484,3.935) | 0.002 (0.001,0.003) | 2.672 (2.058,3.502) | -0.530 (-0.545,-0.518) | 0.002 (0.001,0.002) | 3.395 (2.691,4.241) | 0.002 (0.001,0.003) | 2.849 (2.218,3.717) | -0.563 (-0.577,-0.551) | 0.044 (0.034,0.057) | 86.358 (67.349,110.451) | 0.06 (0.044,0.08) | 72.061 (54.082,95.924) | -0.585 (-0.597,-0.574) |
| Monaco | 0.003 (0.003,0.004) | 5.116 (3.879,6.398) | 0.006 (0.005,0.007) | 6.213 (4.914,7.699) | 0.628 (0.610,0.649) | 0.003 (0.003,0.004) | 4.922 (3.753,6.136) | 0.005 (0.004,0.007) | 5.481 (4.326,6.714) | 0.343 (0.325,0.366) | 0.071 (0.054,0.089) | 116.381 (89.728,145.106) | 0.108 (0.085,0.133) | 127.026 (98.32,157.381) | 0.275 (0.256,0.295) |
| Mongolia | 0.236 (0.194,0.289) | 23.266 (18.958,28.494) | 0.339 (0.27,0.411) | 16.248 (13.148,19.452) | -1.152 (-1.265,-1.035) | 0.254 (0.209,0.312) | 25.544 (20.868,31.408) | 0.359 (0.287,0.435) | 17.984 (14.504,21.489) | -1.139 (-1.261,-1.016) | 6.457 (5.243,7.955) | 600.557 (490.336,739.28) | 9.354 (7.333,11.601) | 397.978 (317.659,481.22) | -1.286 (-1.389,-1.182) |
| Montenegro | 0.013 (0.01,0.015) | 1.956 (1.607,2.406) | 0.022 (0.017,0.028) | 2.218 (1.709,2.847) | 0.488 (0.326,0.608) | 0.013 (0.011,0.016) | 1.996 (1.645,2.475) | 0.022 (0.017,0.028) | 2.288 (1.779,2.92) | 0.759 (0.656,0.906) | 0.363 (0.299,0.444) | 55.117 (45.341,67.475) | 0.558 (0.428,0.716) | 57.882 (44.638,74.133) | 0.237 (0.084,0.356) |
| Morocco | 0.114 (0.089,0.14) | 0.81 (0.633,0.997) | 0.29 (0.21,0.367) | 0.848 (0.621,1.06) | 0.159 (0.137,0.179) | 0.12 (0.094,0.147) | 0.869 (0.68,1.059) | 0.302 (0.219,0.38) | 0.9 (0.661,1.116) | 0.104 (0.076,0.136) | 3.173 (2.482,3.92) | 21.479 (16.755,26.496) | 7.681 (5.483,9.926) | 21.363 (15.36,27.304) | -0.006 (-0.028,0.014) |
| Mozambique | 0.423 (0.335,0.525) | 7.581 (5.986,9.25) | 0.95 (0.716,1.195) | 8.978 (6.9,11.19) | 0.581 (0.554,0.605) | 0.448 (0.357,0.551) | 8.353 (6.684,10.102) | 0.993 (0.749,1.248) | 9.791 (7.555,12.216) | 0.550 (0.521,0.577) | 11.761 (9.329,14.541) | 192.165 (153.19,236.152) | 27.406 (20.219,35.071) | 232.622 (175.471,293.128) | 0.623 (0.591,0.660) |
| Myanmar | 0.936 (0.698,1.201) | 3.922 (2.959,4.995) | 1.104 (0.847,1.5) | 2.253 (1.75,3.04) | -1.780 (-1.799,-1.759) | 0.961 (0.723,1.234) | 4.14 (3.17,5.269) | 1.134 (0.869,1.547) | 2.375 (1.828,3.221) | -1.792 (-1.814,-1.769) | 28.671 (20.983,37.268) | 111.848 (83.348,144.671) | 30.961 (23.497,42.55) | 59.51 (45.593,81.408) | -2.023 (-2.044,-2.001) |
| Namibia | 0.016 (0.013,0.019) | 2.347 (1.947,2.847) | 0.034 (0.026,0.045) | 2.35 (1.834,3.075) | 0.066 (-0.014,0.136) | 0.016 (0.013,0.019) | 2.488 (2.072,3) | 0.034 (0.026,0.046) | 2.464 (1.932,3.191) | 0.044 (-0.032,0.118) | 0.462 (0.371,0.568) | 65.414 (53.146,80.218) | 1.015 (0.744,1.408) | 65.513 (49.502,88.588) | 0.063 (-0.012,0.134) |
| Nauru | 0 (0,0) | 3.994 (2.944,5.166) | 0 (0,0) | 3.255 (2.415,4.192) | -0.661 (-0.675,-0.648) | 0 (0,0) | 4.274 (3.187,5.496) | 0 (0,0) | 3.438 (2.583,4.382) | -0.698 (-0.714,-0.682) | 0.006 (0.004,0.008) | 109.699 (80.337,143.944) | 0.006 (0.004,0.008) | 89.219 (64.206,115.436) | -0.664 (-0.679,-0.649) |
| Nepal | 0.462 (0.349,0.605) | 4.816 (3.66,6.219) | 0.873 (0.624,1.234) | 3.773 (2.71,5.315) | -0.762 (-0.796,-0.732) | 0.475 (0.361,0.617) | 5.127 (3.891,6.62) | 0.91 (0.648,1.298) | 4.041 (2.894,5.731) | -0.743 (-0.777,-0.713) | 14.212 (10.736,18.714) | 135.134 (102.535,176.698) | 24.539 (17.263,35.17) | 100.188 (70.668,142.84) | -0.954 (-0.986,-0.926) |
| Netherlands | 0.943 (0.884,0.998) | 4.785 (4.47,5.048) | 2.44 (2.219,2.635) | 6.884 (6.297,7.403) | 1.152 (1.014,1.255) | 0.959 (0.896,1.013) | 4.817 (4.503,5.08) | 2.263 (2.042,2.45) | 6.212 (5.633,6.693) | 0.779 (0.617,0.888) | 21.876 (20.653,23.065) | 114.562 (108.266,120.989) | 46.29 (42.367,49.718) | 137.078 (126.378,147.012) | 0.531 (0.369,0.633) |
| New Zealand | 0.201 (0.188,0.213) | 5.092 (4.763,5.393) | 0.349 (0.318,0.378) | 4.039 (3.691,4.362) | -0.817 (-1.085,-0.582) | 0.19 (0.178,0.201) | 4.823 (4.514,5.114) | 0.293 (0.265,0.317) | 3.328 (3.032,3.59) | -1.306 (-1.580,-1.052) | 4.124 (3.879,4.345) | 105.924 (99.662,111.83) | 5.77 (5.348,6.181) | 69.673 (64.872,74.5) | -1.486 (-1.726,-1.270) |
| Nicaragua | 0.015 (0.013,0.017) | 1.02 (0.875,1.151) | 0.039 (0.032,0.049) | 0.825 (0.663,1.017) | -0.722 (-0.898,-0.578) | 0.016 (0.014,0.018) | 1.122 (0.967,1.269) | 0.041 (0.034,0.051) | 0.886 (0.719,1.086) | -0.791 (-0.962,-0.651) | 0.413 (0.353,0.467) | 25.712 (22.019,29.046) | 1.031 (0.833,1.283) | 20.446 (16.567,25.236) | -0.791 (-0.993,-0.619) |
| Niger | 0.077 (0.057,0.096) | 2.795 (2.111,3.495) | 0.329 (0.227,0.441) | 4.114 (2.839,5.429) | 1.284 (1.224,1.329) | 0.079 (0.059,0.1) | 3.014 (2.301,3.759) | 0.345 (0.236,0.459) | 4.475 (3.084,5.89) | 1.310 (1.257,1.352) | 2.285 (1.693,2.911) | 75.406 (56.695,95.12) | 9.469 (6.441,12.924) | 107.271 (73.676,143.727) | 1.167 (1.101,1.220) |
| Nigeria | 1.16 (0.888,1.563) | 2.644 (2.04,3.526) | 3.764 (2.543,5.059) | 4.254 (2.916,5.576) | 1.551 (1.515,1.585) | 1.222 (0.938,1.644) | 2.856 (2.208,3.796) | 3.947 (2.695,5.274) | 4.643 (3.216,6.042) | 1.569 (1.526,1.602) | 33.104 (25.129,45.424) | 70.899 (54.161,96.327) | 107.905 (72.624,147.644) | 108.917 (74.14,146.089) | 1.396 (1.365,1.427) |
| Niue | 0 (0,0) | 2.299 (1.848,2.808) | 0 (0,0) | 2.408 (1.871,2.955) | 0.153 (0.134,0.170) | 0 (0,0) | 2.477 (1.994,3.012) | 0 (0,0) | 2.543 (1.988,3.1) | 0.076 (0.053,0.095) | 0.001 (0.001,0.002) | 60.021 (46.912,74.568) | 0.001 (0.001,0.002) | 60.595 (45.972,76.256) | 0.034 (0.014,0.053) |
| North Macedonia | 0.027 (0.023,0.032) | 1.412 (1.238,1.644) | 0.045 (0.034,0.058) | 1.372 (1.06,1.739) | -0.072 (-0.263,0.068) | 0.028 (0.024,0.033) | 1.5 (1.309,1.734) | 0.047 (0.036,0.059) | 1.459 (1.14,1.813) | -0.072 (-0.224,0.056) | 0.765 (0.659,0.904) | 38.651 (33.616,45.333) | 1.185 (0.893,1.521) | 35.282 (26.695,45.056) | -0.256 (-0.447,-0.112) |
| Northern Mariana Islands | 0 (0,0) | 1.373 (1.109,1.764) | 0.001 (0.001,0.002) | 2.596 (2.141,3.087) | 2.008 (1.866,2.143) | 0 (0,0) | 1.473 (1.197,1.891) | 0.001 (0.001,0.001) | 2.714 (2.219,3.235) | 1.918 (1.737,2.063) | 0.007 (0.006,0.01) | 34.356 (27.646,44.629) | 0.035 (0.029,0.041) | 62.76 (52.116,73.905) | 1.947 (1.801,2.070) |
| Norway | 0.174 (0.166,0.181) | 2.626 (2.513,2.72) | 0.335 (0.308,0.354) | 3.322 (3.087,3.496) | 0.905 (0.573,1.093) | 0.174 (0.165,0.181) | 2.559 (2.446,2.653) | 0.301 (0.277,0.318) | 2.916 (2.7,3.072) | 0.565 (0.337,0.748) | 3.767 (3.627,3.897) | 61.115 (59.137,63.181) | 6.094 (5.701,6.391) | 64.088 (60.459,67.036) | 0.290 (0.018,0.464) |
| Oman | 0.013 (0.009,0.017) | 1.925 (1.385,2.543) | 0.03 (0.022,0.039) | 1.587 (1.189,2.001) | -0.557 (-0.612,-0.503) | 0.013 (0.009,0.017) | 2.032 (1.48,2.71) | 0.029 (0.021,0.037) | 1.625 (1.221,2.042) | -0.657 (-0.723,-0.586) | 0.362 (0.258,0.488) | 49.98 (35.996,66.881) | 0.823 (0.599,1.086) | 36.965 (27.452,47.505) | -0.955 (-1.012,-0.897) |
| Pakistan | 3.465 (2.919,3.985) | 6.157 (5.178,7.064) | 7.673 (6.08,9.653) | 6.256 (4.969,7.787) | 0.067 (0.041,0.090) | 3.635 (3.052,4.181) | 6.601 (5.492,7.573) | 7.913 (6.283,9.969) | 6.703 (5.356,8.403) | 0.058 (0.032,0.088) | 99.797 (83.926,115.915) | 167.816 (141.757,193.557) | 228.391 (182.178,289.879) | 168 (133.537,212.345) | 0.022 (-0.006,0.046) |
| Palau | 0 (0,0) | 3.14 (2.507,3.974) | 0.001 (0,0.001) | 2.886 (2.346,3.583) | -0.291 (-0.329,-0.258) | 0 (0,0) | 3.448 (2.783,4.337) | 0.001 (0,0.001) | 3.128 (2.548,3.878) | -0.337 (-0.367,-0.313) | 0.008 (0.006,0.01) | 79.075 (62.523,100.578) | 0.017 (0.013,0.021) | 71.135 (56.17,89.511) | -0.352 (-0.387,-0.327) |
| Palestine | 0.011 (0.008,0.015) | 1.366 (1.011,1.758) | 0.021 (0.017,0.026) | 0.897 (0.728,1.104) | -1.392 (-1.473,-1.315) | 0.012 (0.009,0.015) | 1.502 (1.117,1.928) | 0.021 (0.017,0.026) | 0.965 (0.784,1.183) | -1.469 (-1.550,-1.389) | 0.292 (0.212,0.386) | 33.291 (24.417,43.628) | 0.546 (0.439,0.675) | 20.818 (16.83,25.572) | -1.536 (-1.609,-1.462) |
| Panama | 0.027 (0.025,0.028) | 1.84 (1.707,1.969) | 0.053 (0.042,0.064) | 1.205 (0.937,1.458) | -1.333 (-1.481,-1.219) | 0.029 (0.027,0.031) | 2.021 (1.874,2.162) | 0.057 (0.045,0.069) | 1.284 (1,1.552) | -1.405 (-1.556,-1.288) | 0.671 (0.63,0.712) | 44.505 (41.672,47.502) | 1.255 (0.97,1.519) | 28.404 (21.947,34.439) | -1.442 (-1.606,-1.315) |
| Papua New Guinea | 0.034 (0.023,0.05) | 1.927 (1.332,2.812) | 0.077 (0.053,0.109) | 1.581 (1.106,2.239) | -0.628 (-0.674,-0.580) | 0.034 (0.024,0.05) | 2.084 (1.461,2.997) | 0.078 (0.055,0.112) | 1.716 (1.201,2.425) | -0.606 (-0.650,-0.555) | 1.037 (0.709,1.538) | 51.52 (35.865,74.893) | 2.322 (1.613,3.322) | 41.068 (28.721,58.694) | -0.704 (-0.764,-0.651) |
| Paraguay | 0.068 (0.057,0.082) | 3.123 (2.587,3.747) | 0.234 (0.174,0.315) | 4.015 (2.993,5.397) | 0.959 (0.861,1.072) | 0.072 (0.06,0.087) | 3.352 (2.774,4.06) | 0.243 (0.18,0.325) | 4.22 (3.145,5.649) | 0.909 (0.808,1.030) | 1.879 (1.571,2.275) | 82.184 (68.751,99.245) | 6.314 (4.626,8.426) | 104.666 (77.004,139.442) | 0.884 (0.789,0.984) |
| Peru | 0.197 (0.163,0.239) | 1.717 (1.418,2.072) | 0.419 (0.312,0.557) | 1.266 (0.944,1.681) | -0.812 (-1.156,-0.523) | 0.215 (0.178,0.261) | 1.906 (1.579,2.295) | 0.45 (0.338,0.591) | 1.362 (1.025,1.787) | -0.931 (-1.281,-0.629) | 5.189 (4.262,6.332) | 42.462 (34.835,51.784) | 9.8 (7.157,13.139) | 29.096 (21.297,38.917) | -1.071 (-1.429,-0.769) |
| Philippines | 0.416 (0.367,0.485) | 1.371 (1.207,1.588) | 1.131 (0.923,1.346) | 1.339 (1.1,1.595) | -0.029 (-0.081,0.023) | 0.423 (0.372,0.492) | 1.459 (1.285,1.686) | 1.141 (0.931,1.364) | 1.391 (1.146,1.656) | -0.097 (-0.146,-0.049) | 13.013 (11.51,15.086) | 38.018 (33.469,44.339) | 33.043 (26.837,39.534) | 36.366 (29.658,43.522) | -0.123 (-0.171,-0.070) |
| Poland | 1.535 (1.477,1.585) | 3.54 (3.402,3.656) | 2.062 (1.864,2.249) | 2.983 (2.691,3.252) | -0.541 (-0.727,-0.379) | 1.633 (1.571,1.686) | 3.784 (3.633,3.909) | 2.201 (1.995,2.399) | 3.136 (2.839,3.417) | -0.608 (-0.785,-0.445) | 42.849 (41.396,44.178) | 99.089 (95.769,102.176) | 53.221 (48.09,58.063) | 80.973 (73.459,88.344) | -0.622 (-0.775,-0.469) |
| Portugal | 0.632 (0.592,0.669) | 4.638 (4.347,4.919) | 0.756 (0.689,0.82) | 3.439 (3.14,3.726) | -0.961 (-1.073,-0.843) | 0.663 (0.622,0.702) | 4.879 (4.573,5.165) | 0.745 (0.677,0.813) | 3.239 (2.955,3.524) | -1.337 (-1.452,-1.205) | 15.946 (15.023,16.935) | 118.923 (112.029,126.431) | 17.157 (15.728,18.607) | 85.411 (78.585,92.663) | -1.092 (-1.228,-0.942) |
| Puerto Rico | 0.218 (0.205,0.232) | 6.058 (5.717,6.442) | 0.164 (0.135,0.193) | 2.42 (1.977,2.85) | -3.096 (-3.479,-2.789) | 0.231 (0.216,0.245) | 6.439 (6.065,6.833) | 0.169 (0.138,0.198) | 2.385 (1.949,2.803) | -3.341 (-3.715,-3.052) | 5.462 (5.153,5.82) | 151.956 (143.543,161.674) | 3.635 (2.967,4.285) | 59.498 (48.617,70.747) | -3.142 (-3.539,-2.840) |
| Qatar | 0.004 (0.003,0.006) | 5.029 (3.979,6.221) | 0.02 (0.015,0.028) | 2.591 (1.93,3.433) | -2.127 (-2.400,-1.869) | 0.004 (0.004,0.006) | 5.534 (4.396,6.837) | 0.018 (0.013,0.025) | 2.645 (1.977,3.511) | -2.381 (-2.700,-2.076) | 0.135 (0.106,0.171) | 114.929 (90.516,143.107) | 0.569 (0.401,0.795) | 52.597 (38.833,70.862) | -2.507 (-2.720,-2.296) |
| Republic of Korea | 1.84 (1.491,2.23) | 6.021 (4.908,7.279) | 3.383 (2.699,4.139) | 3.524 (2.807,4.314) | -1.783 (-1.841,-1.717) | 1.716 (1.39,2.075) | 5.799 (4.724,6.978) | 2.196 (1.757,2.698) | 2.289 (1.831,2.807) | -3.030 (-3.088,-2.961) | 48.742 (39.279,59.171) | 148.528 (120.203,180.031) | 48.07 (38.608,59.205) | 50.149 (40.363,61.891) | -3.502 (-3.563,-3.436) |
| Republic of Moldova | 0.11 (0.104,0.116) | 2.46 (2.321,2.591) | 0.112 (0.1,0.126) | 1.865 (1.673,2.087) | -0.822 (-1.044,-0.557) | 0.113 (0.106,0.119) | 2.558 (2.413,2.696) | 0.111 (0.099,0.124) | 1.838 (1.647,2.048) | -1.001 (-1.233,-0.714) | 3.144 (2.944,3.328) | 68.159 (64.004,71.99) | 2.97 (2.652,3.329) | 50.036 (44.81,55.886) | -0.852 (-1.116,-0.541) |
| Romania | 0.419 (0.395,0.448) | 1.493 (1.41,1.585) | 0.856 (0.751,0.97) | 2.522 (2.204,2.863) | 1.754 (1.631,1.868) | 0.433 (0.409,0.462) | 1.563 (1.478,1.655) | 0.879 (0.775,0.991) | 2.545 (2.237,2.886) | 1.633 (1.508,1.748) | 12.329 (11.593,13.205) | 43.508 (40.916,46.471) | 23.274 (20.437,26.424) | 72.119 (63.272,82.083) | 1.672 (1.543,1.798) |
| Russian Federation | 9.162 (8.974,9.327) | 4.991 (4.879,5.08) | 7.919 (7.177,8.608) | 3.325 (3.015,3.611) | -1.149 (-1.338,-0.916) | 9.347 (9.15,9.516) | 5.139 (5.018,5.229) | 7.709 (7.022,8.377) | 3.215 (2.929,3.49) | -1.369 (-1.571,-1.099) | 252.62 (247.639,257.196) | 136.168 (133.427,138.668) | 201.901 (182.636,219.875) | 86.829 (78.567,94.586) | -1.310 (-1.497,-1.066) |
| Rwanda | 0.611 (0.418,0.774) | 21.067 (14.525,26.508) | 0.698 (0.507,0.925) | 11.279 (8.265,14.89) | -2.010 (-2.059,-1.957) | 0.628 (0.429,0.794) | 22.38 (15.439,28.058) | 0.724 (0.527,0.969) | 12.218 (9.015,16.334) | -1.946 (-1.993,-1.894) | 18.736 (12.72,23.69) | 596.514 (407.812,751.867) | 20.353 (14.498,27.402) | 295.316 (214.928,395.437) | -2.266 (-2.316,-2.211) |
| Saint Kitts and Nevis | 0.002 (0.002,0.002) | 4.433 (4.15,4.701) | 0.003 (0.002,0.003) | 3.806 (3.174,4.437) | -0.545 (-0.814,-0.376) | 0.002 (0.002,0.002) | 4.776 (4.483,5.059) | 0.003 (0.002,0.003) | 4.027 (3.353,4.664) | -0.602 (-0.879,-0.426) | 0.041 (0.038,0.044) | 118.416 (110.682,125.95) | 0.074 (0.061,0.089) | 97.616 (80.235,115.042) | -0.665 (-0.880,-0.527) |
| Saint Lucia | 0.004 (0.004,0.005) | 5.111 (4.821,5.44) | 0.01 (0.008,0.012) | 4.145 (3.391,4.979) | -0.490 (-0.753,-0.300) | 0.005 (0.004,0.005) | 5.536 (5.229,5.886) | 0.011 (0.009,0.013) | 4.38 (3.588,5.251) | -0.593 (-0.847,-0.401) | 0.116 (0.109,0.124) | 134.248 (126.241,143.141) | 0.267 (0.215,0.323) | 108.844 (87.96,131.404) | -0.421 (-0.728,-0.200) |
| Saint Vincent and the Grenadines | 0.002 (0.001,0.002) | 2.305 (2.116,2.483) | 0.004 (0.003,0.004) | 2.472 (2.172,2.808) | 0.134 (0.006,0.241) | 0.002 (0.002,0.002) | 2.475 (2.265,2.668) | 0.004 (0.003,0.004) | 2.618 (2.295,2.966) | 0.086 (-0.053,0.199) | 0.043 (0.039,0.047) | 61.234 (56.164,66.445) | 0.096 (0.084,0.111) | 66.245 (58.152,76.242) | 0.169 (0.053,0.268) |
| Samoa | 0.001 (0.001,0.001) | 1.255 (0.998,1.533) | 0.002 (0.001,0.002) | 1.116 (0.885,1.416) | -0.386 (-0.401,-0.372) | 0.001 (0.001,0.001) | 1.347 (1.064,1.635) | 0.002 (0.001,0.002) | 1.166 (0.921,1.466) | -0.471 (-0.483,-0.458) | 0.029 (0.023,0.037) | 32.791 (25.574,40.756) | 0.044 (0.034,0.056) | 28.914 (22.245,36.877) | -0.405 (-0.418,-0.392) |
| San Marino | 0.001 (0,0.001) | 1.667 (1.313,2.068) | 0.001 (0,0.001) | 0.959 (0.597,1.423) | -2.040 (-2.450,-1.798) | 0.001 (0,0.001) | 1.605 (1.267,1.986) | 0.001 (0,0.001) | 0.847 (0.534,1.254) | -2.392 (-2.843,-2.126) | 0.013 (0.01,0.016) | 38.563 (30.093,48.467) | 0.013 (0.008,0.02) | 20.321 (12.088,30.603) | -2.354 (-2.777,-2.105) |
| Sao Tome and Principe | 0.001 (0.001,0.002) | 2.314 (1.84,2.845) | 0.005 (0.004,0.007) | 4.615 (3.546,5.865) | 2.243 (2.189,2.296) | 0.002 (0.001,0.002) | 2.493 (2,3.05) | 0.005 (0.004,0.007) | 4.935 (3.76,6.258) | 2.230 (2.174,2.276) | 0.041 (0.032,0.051) | 61.397 (48.234,76.948) | 0.146 (0.109,0.194) | 120.415 (91.75,156.995) | 2.188 (2.133,2.257) |
| Saudi Arabia | 0.12 (0.085,0.161) | 2.152 (1.559,2.867) | 0.331 (0.253,0.446) | 1.752 (1.385,2.229) | -0.658 (-0.692,-0.624) | 0.125 (0.09,0.167) | 2.324 (1.702,3.073) | 0.311 (0.238,0.419) | 1.808 (1.435,2.325) | -0.774 (-0.809,-0.730) | 3.421 (2.431,4.663) | 54.803 (39.309,73.762) | 9.773 (7.331,13.484) | 41.632 (32.403,54.549) | -0.877 (-0.920,-0.834) |
| Senegal | 0.097 (0.078,0.118) | 3.025 (2.445,3.641) | 0.388 (0.292,0.506) | 5.061 (3.817,6.541) | 1.720 (1.567,1.831) | 0.102 (0.082,0.123) | 3.25 (2.644,3.891) | 0.407 (0.307,0.529) | 5.461 (4.092,7.051) | 1.735 (1.594,1.842) | 2.78 (2.214,3.398) | 81.553 (65.564,99.274) | 10.96 (8.178,14.608) | 132.947 (99.505,173.988) | 1.634 (1.456,1.758) |
| Serbia | 0.269 (0.201,0.364) | 2.372 (1.796,3.173) | 0.34 (0.246,0.465) | 2.142 (1.539,2.941) | -0.257 (-0.376,-0.131) | 0.278 (0.209,0.377) | 2.522 (1.921,3.357) | 0.35 (0.255,0.478) | 2.168 (1.573,2.981) | -0.436 (-0.589,-0.234) | 7.669 (5.682,10.486) | 64.34 (48.129,87.417) | 8.522 (6.122,11.827) | 56.207 (40.258,78.214) | -0.375 (-0.510,-0.222) |
| Seychelles | 0.003 (0.003,0.004) | 5.408 (4.545,6.383) | 0.006 (0.005,0.007) | 4.727 (3.845,5.732) | -0.474 (-0.610,-0.356) | 0.003 (0.003,0.004) | 5.589 (4.728,6.563) | 0.006 (0.005,0.007) | 4.767 (3.88,5.757) | -0.567 (-0.727,-0.434) | 0.085 (0.071,0.101) | 153.156 (127.598,181.983) | 0.157 (0.127,0.194) | 124.351 (100.166,152.454) | -0.679 (-0.802,-0.581) |
| Sierra Leone | 0.056 (0.044,0.07) | 2.779 (2.186,3.43) | 0.181 (0.13,0.242) | 4.829 (3.472,6.392) | 1.831 (1.793,1.865) | 0.06 (0.046,0.074) | 2.995 (2.35,3.686) | 0.189 (0.136,0.254) | 5.188 (3.752,6.889) | 1.819 (1.780,1.855) | 1.564 (1.188,1.976) | 74.495 (57.132,93.788) | 5.246 (3.778,7.196) | 128.687 (92.704,174.103) | 1.813 (1.773,1.849) |
| Singapore | 0.102 (0.096,0.109) | 4.715 (4.424,5.016) | 0.224 (0.202,0.244) | 2.588 (2.333,2.814) | -1.891 (-2.652,-1.237) | 0.091 (0.086,0.097) | 4.3 (4.047,4.567) | 0.146 (0.131,0.158) | 1.706 (1.532,1.843) | -3.023 (-3.671,-2.435) | 2.34 (2.205,2.494) | 102.434 (96.447,109.166) | 3.305 (2.996,3.574) | 37.685 (34.218,40.638) | -3.222 (-3.836,-2.675) |
| Slovakia | 0.244 (0.19,0.314) | 4.132 (3.231,5.315) | 0.3 (0.23,0.398) | 3.248 (2.474,4.328) | -0.762 (-0.882,-0.652) | 0.245 (0.193,0.316) | 4.144 (3.27,5.32) | 0.299 (0.231,0.394) | 3.212 (2.465,4.271) | -0.805 (-0.919,-0.699) | 7.007 (5.333,9.183) | 120.366 (92.093,157.575) | 7.864 (5.901,10.777) | 87.876 (65.22,121.552) | -1.000 (-1.138,-0.869) |
| Slovenia | 0.085 (0.08,0.091) | 3.417 (3.21,3.639) | 0.104 (0.089,0.117) | 2.435 (2.078,2.758) | -1.160 (-1.418,-0.989) | 0.086 (0.08,0.091) | 3.431 (3.224,3.655) | 0.096 (0.083,0.109) | 2.206 (1.881,2.49) | -1.484 (-1.747,-1.308) | 2.326 (2.171,2.49) | 93.811 (87.638,100.268) | 2.18 (1.849,2.486) | 55.027 (46.457,62.733) | -1.794 (-2.038,-1.632) |
| Solomon Islands | 0.004 (0.002,0.005) | 2.739 (1.948,3.602) | 0.008 (0.006,0.012) | 2.404 (1.649,3.326) | -0.465 (-0.523,-0.422) | 0.004 (0.002,0.005) | 2.96 (2.159,3.853) | 0.008 (0.006,0.012) | 2.579 (1.806,3.545) | -0.449 (-0.511,-0.395) | 0.114 (0.072,0.157) | 74.674 (49.916,101.061) | 0.261 (0.174,0.371) | 65.569 (44.226,91.53) | -0.465 (-0.521,-0.419) |
| Somalia | 0.51 (0.347,0.699) | 20.307 (14.23,27.19) | 0.923 (0.642,1.243) | 14.906 (10.246,19.715) | -0.987 (-1.002,-0.973) | 0.516 (0.355,0.709) | 21.525 (15.196,28.897) | 0.94 (0.657,1.264) | 15.965 (11.096,21.097) | -0.941 (-0.959,-0.923) | 16.532 (11.2,23.159) | 575.456 (400.098,782.864) | 29.091 (20.093,39.372) | 410.584 (287.394,546.215) | -1.073 (-1.088,-1.052) |
| South Africa | 2.379 (2.101,2.812) | 11.161 (9.824,13.424) | 4.857 (4.41,5.433) | 10.427 (9.452,11.604) | -0.336 (-0.518,-0.088) | 2.439 (2.15,2.918) | 11.75 (10.348,14.275) | 5.009 (4.544,5.609) | 11.061 (10.064,12.297) | -0.289 (-0.460,-0.058) | 73.766 (65.651,86.962) | 322.874 (286.317,380.879) | 138.651 (125.93,155.95) | 279.793 (254.979,313.694) | -0.530 (-0.701,-0.326) |
| South Sudan | 0.449 (0.331,0.611) | 17.525 (12.942,23.528) | 0.599 (0.43,0.799) | 15.156 (11.115,20.126) | -0.451 (-0.479,-0.422) | 0.469 (0.347,0.64) | 18.708 (13.922,25.25) | 0.614 (0.443,0.82) | 16.21 (11.98,21.421) | -0.439 (-0.463,-0.413) | 12.963 (9.508,17.892) | 481.422 (353.653,661.883) | 18.316 (13.028,24.573) | 411.795 (298.256,551.715) | -0.475 (-0.506,-0.442) |
| Spain | 2.247 (2.115,2.374) | 4.284 (4.028,4.518) | 2.563 (2.312,2.77) | 2.814 (2.574,3.031) | -1.399 (-1.501,-1.329) | 2.199 (2.069,2.322) | 4.15 (3.898,4.38) | 2.292 (2.055,2.476) | 2.405 (2.181,2.601) | -1.761 (-1.869,-1.688) | 55.592 (52.508,58.824) | 109.784 (103.328,116.073) | 51.241 (46.889,55.433) | 60.237 (55.217,65.155) | -2.003 (-2.110,-1.916) |
| Sri Lanka | 0.602 (0.508,0.727) | 5.669 (4.799,6.779) | 1.164 (0.764,1.582) | 4.258 (2.817,5.802) | -0.846 (-0.989,-0.717) | 0.618 (0.521,0.744) | 6.031 (5.104,7.174) | 1.122 (0.733,1.529) | 4.16 (2.763,5.687) | -1.104 (-1.246,-0.977) | 17.176 (14.415,20.695) | 148.155 (125.128,177.695) | 28.219 (18.168,39.075) | 101.745 (65.819,140.489) | -1.177 (-1.345,-1.027) |
| Sudan | 0.439 (0.274,0.585) | 4.744 (2.988,6.294) | 0.81 (0.498,1.154) | 4.237 (2.699,5.949) | -0.359 (-0.369,-0.349) | 0.459 (0.288,0.617) | 5.096 (3.2,6.877) | 0.839 (0.526,1.189) | 4.561 (2.974,6.31) | -0.351 (-0.361,-0.341) | 12.728 (7.721,17.328) | 128.601 (79.427,173.697) | 23.043 (13.704,33.22) | 108.348 (66.934,154.983) | -0.550 (-0.560,-0.541) |
| Suriname | 0.004 (0.004,0.005) | 1.742 (1.505,2.012) | 0.01 (0.008,0.014) | 1.605 (1.197,2.085) | -0.152 (-0.408,0.062) | 0.005 (0.004,0.005) | 1.863 (1.612,2.146) | 0.011 (0.008,0.014) | 1.688 (1.261,2.161) | -0.203 (-0.436,0.002) | 0.127 (0.107,0.147) | 47.037 (40.211,54.297) | 0.295 (0.219,0.381) | 44.119 (32.811,56.743) | -0.110 (-0.340,0.087) |
| Sweden | 0.416 (0.389,0.439) | 2.764 (2.607,2.898) | 0.625 (0.541,0.697) | 2.862 (2.496,3.193) | 0.414 (0.255,0.585) | 0.415 (0.385,0.439) | 2.687 (2.523,2.82) | 0.597 (0.516,0.666) | 2.625 (2.276,2.93) | 0.216 (0.072,0.392) | 8.488 (8.01,8.904) | 60.574 (57.533,63.511) | 11.286 (9.814,12.626) | 56.479 (49.485,63.531) | 0.097 (-0.116,0.304) |
| Switzerland | 0.46 (0.433,0.486) | 4.577 (4.307,4.831) | 0.575 (0.511,0.624) | 3.224 (2.898,3.492) | -1.136 (-1.379,-0.988) | 0.45 (0.422,0.475) | 4.384 (4.137,4.618) | 0.532 (0.467,0.578) | 2.84 (2.541,3.068) | -1.405 (-1.647,-1.229) | 10.511 (9.901,11.096) | 109.728 (103.023,116.113) | 10.709 (9.631,11.581) | 64.236 (58.655,69.189) | -1.738 (-2.036,-1.569) |
| Syrian Arab Republic | 0.043 (0.034,0.054) | 0.846 (0.665,1.058) | 0.11 (0.079,0.146) | 0.89 (0.659,1.154) | 0.114 (0.006,0.219) | 0.045 (0.035,0.056) | 0.918 (0.725,1.138) | 0.112 (0.081,0.145) | 0.951 (0.715,1.204) | 0.062 (-0.040,0.156) | 1.235 (0.954,1.559) | 21.996 (17.115,27.568) | 2.872 (2.027,3.834) | 20.901 (15.139,27.378) | -0.142 (-0.235,-0.046) |
| Taiwan  (Province of China) | 1.471 (1.395,1.557) | 9.052 (8.605,9.581) | 3.799 (3.434,4.12) | 9.302 (8.405,10.064) | 0.039 (-0.073,0.141) | 1.407 (1.336,1.491) | 8.896 (8.443,9.396) | 3.093 (2.778,3.356) | 7.455 (6.722,8.07) | -0.680 (-0.783,-0.583) | 39.78 (37.786,42.192) | 233.256 (221.894,247.002) | 87.762 (78.834,94.875) | 219.551 (198.135,237.206) | -0.254 (-0.383,-0.142) |
| Tajikistan | 0.308 (0.252,0.369) | 11.402 (9.403,13.621) | 0.328 (0.242,0.428) | 6.131 (4.579,7.87) | -2.079 (-2.182,-1.974) | 0.327 (0.269,0.392) | 12.339 (10.233,14.663) | 0.346 (0.257,0.445) | 6.769 (5.068,8.66) | -2.011 (-2.113,-1.907) | 8.646 (7.051,10.353) | 304.18 (249.153,365.233) | 9.341 (6.786,12.297) | 153.525 (113.813,197.765) | -2.276 (-2.380,-2.175) |
| Thailand | 1.76 (1.359,2.359) | 4.835 (3.685,6.453) | 5.144 (3.85,6.747) | 4.725 (3.556,6.184) | -0.153 (-0.239,-0.063) | 1.777 (1.385,2.385) | 5.033 (3.877,6.723) | 4.849 (3.619,6.301) | 4.437 (3.329,5.752) | -0.508 (-0.602,-0.408) | 51.5 (40.451,69.514) | 131.025 (102.865,175.999) | 134.35 (99.351,177.193) | 124.839 (92.388,164.752) | -0.220 (-0.311,-0.115) |
| Timor-Leste | 0.007 (0.005,0.01) | 2.577 (1.847,3.356) | 0.018 (0.013,0.024) | 2.094 (1.539,2.837) | -0.691 (-0.763,-0.637) | 0.007 (0.005,0.01) | 2.758 (1.991,3.581) | 0.019 (0.014,0.026) | 2.231 (1.647,3.046) | -0.703 (-0.765,-0.654) | 0.234 (0.159,0.318) | 70.597 (49.605,93.552) | 0.491 (0.35,0.691) | 54.953 (39.447,76.399) | -0.831 (-0.883,-0.782) |
| Togo | 0.037 (0.029,0.046) | 3.009 (2.389,3.725) | 0.227 (0.161,0.309) | 5.822 (4.099,7.744) | 2.195 (2.156,2.228) | 0.038 (0.03,0.047) | 3.232 (2.588,3.972) | 0.232 (0.164,0.315) | 6.224 (4.329,8.235) | 2.176 (2.139,2.209) | 1.088 (0.856,1.358) | 81.079 (63.988,101.204) | 6.835 (4.85,9.35) | 157.054 (111.162,213.04) | 2.202 (2.156,2.241) |
| Tokelau | 0 (0,0) | 2.144 (1.472,2.799) | 0 (0,0) | 1.818 (1.37,2.278) | -0.530 (-0.548,-0.513) | 0 (0,0) | 2.34 (1.614,3.038) | 0 (0,0) | 1.938 (1.458,2.421) | -0.607 (-0.625,-0.589) | 0.001 (0.001,0.001) | 55.38 (37.626,73.342) | 0.001 (0.001,0.001) | 45.866 (34.06,58.519) | -0.605 (-0.620,-0.590) |
| Tonga | 0.001 (0.001,0.001) | 2.007 (1.598,2.496) | 0.002 (0.001,0.002) | 1.982 (1.523,2.569) | -0.065 (-0.120,-0.007) | 0.001 (0.001,0.001) | 2.176 (1.739,2.708) | 0.002 (0.001,0.002) | 2.116 (1.63,2.737) | -0.110 (-0.166,-0.050) | 0.029 (0.024,0.037) | 51.387 (41.115,63.54) | 0.04 (0.03,0.053) | 49.663 (37.645,64.898) | -0.135 (-0.186,-0.081) |
| Trinidad and Tobago | 0.019 (0.018,0.02) | 2.334 (2.206,2.465) | 0.037 (0.029,0.048) | 1.926 (1.471,2.442) | -0.329 (-0.619,0.042) | 0.02 (0.019,0.021) | 2.51 (2.372,2.654) | 0.039 (0.03,0.049) | 2.006 (1.546,2.542) | -0.438 (-0.719,-0.074) | 0.522 (0.493,0.553) | 61.503 (58.057,65.122) | 1.003 (0.761,1.291) | 51.788 (39.322,66.652) | -0.216 (-0.591,0.234) |
| Tunisia | 0.032 (0.026,0.04) | 0.664 (0.53,0.815) | 0.088 (0.061,0.124) | 0.666 (0.458,0.93) | 0.022 (-0.029,0.074) | 0.034 (0.027,0.041) | 0.714 (0.571,0.878) | 0.089 (0.062,0.125) | 0.687 (0.475,0.955) | -0.113 (-0.215,-0.058) | 0.856 (0.679,1.059) | 16.521 (13.184,20.361) | 2.166 (1.459,3.059) | 15.828 (10.747,22.279) | -0.127 (-0.200,-0.071) |
| Turkey | 0.823 (0.67,0.989) | 2.386 (1.955,2.866) | 1.51 (1.143,1.918) | 1.627 (1.242,2.072) | -1.227 (-1.294,-1.162) | 0.855 (0.696,1.027) | 2.559 (2.109,3.078) | 1.526 (1.155,1.932) | 1.673 (1.277,2.121) | -1.339 (-1.419,-1.266) | 24.054 (19.013,29.225) | 64.079 (51.684,77.559) | 37.098 (27.842,46.777) | 38.743 (29.158,48.69) | -1.643 (-1.704,-1.581) |
| Turkmenistan | 0.557 (0.522,0.593) | 29.445 (27.591,31.341) | 0.337 (0.255,0.433) | 8.396 (6.421,10.728) | -3.810 (-4.240,-3.491) | 0.584 (0.547,0.621) | 31.576 (29.533,33.6) | 0.35 (0.268,0.451) | 8.978 (6.94,11.484) | -3.810 (-4.232,-3.493) | 15.948 (14.862,16.971) | 793.704 (742.551,842.696) | 9.667 (7.337,12.53) | 223.548 (170.846,288.612) | -3.886 (-4.237,-3.575) |
| Tuvalu | 0 (0,0) | 2.576 (2.053,3.125) | 0 (0,0) | 2.11 (1.667,2.631) | -0.640 (-0.652,-0.629) | 0 (0,0) | 2.791 (2.223,3.393) | 0 (0,0) | 2.26 (1.777,2.832) | -0.680 (-0.690,-0.670) | 0.005 (0.004,0.006) | 69.71 (54.67,85.325) | 0.006 (0.005,0.008) | 55.903 (43.153,70.415) | -0.715 (-0.723,-0.707) |
| Uganda | 0.924 (0.733,1.142) | 14.518 (11.608,17.731) | 2.181 (1.673,2.867) | 14.808 (11.468,19.565) | 0.048 (0.006,0.089) | 0.966 (0.765,1.19) | 15.62 (12.528,19.032) | 2.253 (1.721,2.972) | 15.901 (12.408,20.929) | 0.046 (0.005,0.087) | 26.81 (21.024,33.339) | 391.447 (309.401,485.101) | 64.89 (48.346,85.796) | 395.909 (302.087,521.131) | 0.019 (-0.027,0.065) |
| Ukraine | 2.423 (2.268,2.597) | 3.359 (3.15,3.594) | 1.821 (1.258,2.536) | 2.454 (1.683,3.423) | -0.704 (-1.038,-0.431) | 2.298 (2.156,2.464) | 3.183 (2.994,3.399) | 1.658 (1.139,2.299) | 2.213 (1.517,3.079) | -0.869 (-1.117,-0.652) | 65.567 (61.174,70.556) | 91.795 (85.833,98.631) | 47.424 (31.908,66.342) | 66.151 (44.537,92.635) | -0.770 (-1.034,-0.529) |
| United Arab Emirates | 0.01 (0.007,0.014) | 2.378 (1.747,3.193) | 0.063 (0.047,0.081) | 2.144 (1.652,2.671) | -0.465 (-0.990,0.035) | 0.01 (0.007,0.014) | 2.559 (1.903,3.395) | 0.06 (0.044,0.078) | 2.359 (1.848,2.926) | -0.394 (-0.926,0.114) | 0.323 (0.235,0.441) | 60.249 (44.32,80.997) | 1.972 (1.469,2.562) | 46.258 (35.71,58.382) | -0.956 (-1.382,-0.543) |
| United Kingdom | 6.609 (6.337,6.756) | 7.299 (7.028,7.452) | 9.949 (9.232,10.365) | 7.544 (7.079,7.834) | 0.111 (-0.000,0.201) | 6.748 (6.456,6.907) | 7.349 (7.055,7.515) | 9.746 (8.99,10.17) | 7.16 (6.693,7.445) | -0.090 (-0.197,0.012) | 143.874 (139.693,146.437) | 168.026 (163.682,170.777) | 189.121 (178.951,195.972) | 154.574 (147.549,159.752) | -0.287 (-0.402,-0.177) |
| United Republic of Tanzania | 1.748 (1.309,2.185) | 16.054 (12.16,20.06) | 2.877 (2.113,3.764) | 11.358 (8.382,14.774) | -1.105 (-1.126,-1.085) | 1.815 (1.352,2.263) | 17.193 (12.965,21.354) | 2.995 (2.202,3.928) | 12.241 (9.119,16.061) | -1.081 (-1.102,-1.062) | 51.398 (38.241,64.756) | 438.816 (327.366,549.773) | 83.556 (60.797,110.436) | 300.142 (220.091,394.935) | -1.216 (-1.241,-1.196) |
| United States of America | 12.883 (12.245,13.191) | 4.156 (3.967,4.251) | 24.329 (22.769,25.279) | 4.203 (3.954,4.363) | 0.008 (-0.042,0.057) | 11.774 (11.161,12.077) | 3.752 (3.571,3.844) | 21.341 (19.927,22.216) | 3.62 (3.398,3.761) | -0.153 (-0.198,-0.109) | 285.637 (275.508,291.098) | 95.827 (92.735,97.546) | 479.504 (457.854,495.41) | 86.551 (82.915,89.359) | -0.385 (-0.437,-0.334) |
| United States Virgin Islands | 0.003 (0.002,0.003) | 3.334 (2.729,3.996) | 0.004 (0.003,0.005) | 2.324 (1.773,2.999) | -1.209 (-1.327,-1.107) | 0.003 (0.002,0.003) | 3.549 (2.923,4.245) | 0.004 (0.003,0.005) | 2.424 (1.862,3.092) | -1.269 (-1.393,-1.170) | 0.079 (0.063,0.096) | 86.761 (69.875,105.049) | 0.1 (0.076,0.13) | 61.04 (46.481,80.439) | -1.171 (-1.268,-1.078) |
| Uruguay | 0.328 (0.309,0.348) | 8.391 (7.874,8.868) | 0.305 (0.28,0.329) | 5.445 (5.004,5.896) | -1.438 (-1.650,-1.235) | 0.352 (0.33,0.372) | 8.971 (8.41,9.491) | 0.325 (0.298,0.352) | 5.642 (5.194,6.106) | -1.538 (-1.746,-1.334) | 7.945 (7.461,8.441) | 206.969 (194.569,220.049) | 6.618 (6.08,7.188) | 126.681 (116.377,136.862) | -1.664 (-1.861,-1.458) |
| Uzbekistan | 1.623 (1.475,1.776) | 14.181 (12.848,15.509) | 0.964 (0.795,1.152) | 3.711 (3.085,4.417) | -4.341 (-4.677,-4.167) | 1.709 (1.546,1.87) | 15.101 (13.644,16.496) | 0.999 (0.826,1.201) | 3.977 (3.308,4.756) | -4.332 (-4.671,-4.150) | 46.168 (41.816,50.574) | 388.451 (351.907,426.793) | 28.052 (22.931,33.989) | 98.603 (81.206,118.148) | -4.418 (-4.748,-4.239) |
| Vanuatu | 0.002 (0.001,0.002) | 2.467 (1.851,3.264) | 0.004 (0.003,0.005) | 2.212 (1.733,2.823) | -0.400 (-0.434,-0.364) | 0.002 (0.001,0.002) | 2.673 (2.015,3.514) | 0.004 (0.003,0.005) | 2.39 (1.882,3.046) | -0.391 (-0.425,-0.353) | 0.046 (0.033,0.064) | 66.352 (48.884,90.051) | 0.114 (0.087,0.15) | 59.086 (45.86,76.541) | -0.423 (-0.468,-0.378) |
| Venezuela  (Bolivarian Republic of) | 0.232 (0.219,0.243) | 2.499 (2.358,2.624) | 0.49 (0.354,0.653) | 1.647 (1.197,2.188) | -1.540 (-2.445,-1.269) | 0.248 (0.234,0.26) | 2.739 (2.58,2.874) | 0.515 (0.376,0.685) | 1.755 (1.282,2.325) | -1.124 (-1.673,-0.605) | 6.197 (5.889,6.501) | 62.422 (59.1,65.515) | 12.625 (8.928,16.989) | 41.291 (29.447,55.366) | -1.576 (-2.423,-1.335) |
| Viet Nam | 0.823 (0.612,1.082) | 2.032 (1.523,2.66) | 2.426 (1.798,3.097) | 2.327 (1.775,2.944) | 0.450 (0.422,0.480) | 0.855 (0.642,1.12) | 2.134 (1.612,2.776) | 2.344 (1.757,2.997) | 2.298 (1.752,2.896) | 0.266 (0.236,0.298) | 22.972 (16.927,30.619) | 55.34 (40.989,73.591) | 66.14 (47.736,86.249) | 60.591 (44.432,77.887) | 0.303 (0.278,0.328) |
| Yemen | 0.243 (0.14,0.347) | 5.012 (2.862,7.182) | 0.526 (0.273,0.756) | 3.845 (2.035,5.5) | -0.832 (-0.862,-0.800) | 0.251 (0.145,0.358) | 5.371 (3.05,7.707) | 0.546 (0.283,0.783) | 4.156 (2.191,5.922) | -0.803 (-0.830,-0.774) | 7.211 (4.174,10.344) | 135.249 (78.308,193.009) | 15.082 (7.659,22.075) | 98.888 (51.163,142.832) | -0.982 (-1.019,-0.944) |
| Zambia | 0.558 (0.431,0.688) | 19.299 (14.973,23.877) | 1.133 (0.752,1.743) | 16.022 (10.946,24.445) | -0.594 (-0.636,-0.557) | 0.576 (0.442,0.712) | 20.603 (16.02,25.519) | 1.159 (0.77,1.801) | 17.127 (11.781,26.087) | -0.584 (-0.628,-0.548) | 16.957 (12.808,20.88) | 535.947 (408.878,662.052) | 34.919 (22.965,55.565) | 436.302 (290.09,677.098) | -0.658 (-0.699,-0.624) |
| Zimbabwe | 0.503 (0.404,0.607) | 12.581 (10.214,15.057) | 1.12 (0.844,1.402) | 15.925 (12.306,19.642) | 0.825 (0.763,0.903) | 0.525 (0.421,0.63) | 13.537 (10.919,16.139) | 1.148 (0.871,1.444) | 17.021 (13.229,21.113) | 0.816 (0.753,0.897) | 14.376 (11.465,17.332) | 333.043 (265.984,399.564) | 34.11 (25.272,43.725) | 435.15 (329.44,549.188) | 0.906 (0.843,0.979) |

Supplementary Table S3. Age-standardised rate of stomach cancer at nation level between 1990 and 2021

| **location** | **Incidence** | | | | | **Deaths** | | | | | **DALYs** | | | | |
| --- | --- | --- | --- | --- | --- | --- | --- | --- | --- | --- | --- | --- | --- | --- | --- |
|  | **Number of cases, 1990  (thousands)** | **Age-standardised rate per 100,000 population, 1990** | **Number of cases, 2021  (thousands)** | **Age-standardised rate per 100,000 population, 2021** | **Average Annual Percent Change 1990 to 2021** | **Number of cases, 1990  (thousands)** | **Age-standardised rate per 100,000 population, 1990** | **Number of cases, 2021  (thousands)** | **Age-standardised rate per 100,000 population, 2021** | **Average Annual Percent Change 1990 to 2021** | **Number of cases, 1990  (thousands)** | **Age-standardised rate per 100,000 population, 1990** | **Number of cases, 2021  (thousands)** | **Age-standardised rate per 100,000 population, 2021** | **Average Annual Percent Change 1990 to 2021** |
| Afghanistan | 2.759 (1.608,3.775) | 39.67 (23.323,53.665) | 3.479 (1.874,4.818) | 32.924 (17.93,44.962) | -0.593 (-0.611,-0.572) | 2.82 (1.683,3.85) | 41.531 (24.931,56.018) | 3.469 (1.888,4.78) | 34.567 (19.015,46.902) | -0.581 (-0.598,-0.562) | 82.524 (47.148,114.272) | 1137.847 (658.956,1575.8) | 115.055 (60.033,161.225) | 910.244 (498.856,1256.824) | -0.714 (-0.738,-0.687) |
| Albania | 0.337 (0.278,0.41) | 16.941 (14.035,20.824) | 0.462 (0.35,0.599) | 10.6 (8.044,13.661) | -1.619 (-1.759,-1.509) | 0.341 (0.282,0.415) | 17.624 (14.607,21.874) | 0.45 (0.341,0.587) | 10.353 (7.834,13.471) | -1.819 (-1.980,-1.684) | 8.729 (7.164,10.509) | 404.811 (334.408,490.337) | 9.576 (7.193,12.253) | 225.877 (170.216,289.898) | -1.994 (-2.150,-1.865) |
| Algeria | 0.664 (0.486,0.809) | 6.026 (4.381,7.215) | 1.239 (0.931,1.526) | 3.751 (2.836,4.598) | -1.508 (-1.531,-1.484) | 0.665 (0.489,0.815) | 6.442 (4.721,7.718) | 1.182 (0.895,1.455) | 3.787 (2.881,4.628) | -1.692 (-1.719,-1.666) | 18.349 (13.643,22.459) | 144.697 (107.062,176.081) | 30.001 (22.602,37.043) | 81.58 (61.976,100.254) | -1.844 (-1.863,-1.826) |
| American Samoa | 0.005 (0.004,0.006) | 20.579 (17.875,26.029) | 0.008 (0.007,0.01) | 17.99 (14.797,22.519) | -0.365 (-0.460,-0.272) | 0.004 (0.004,0.006) | 21.074 (18.313,26.467) | 0.008 (0.006,0.01) | 17.636 (14.509,21.861) | -0.542 (-0.613,-0.479) | 0.137 (0.116,0.174) | 519.644 (448.035,659.385) | 0.211 (0.172,0.269) | 433.99 (352.921,547.241) | -0.497 (-0.590,-0.400) |
| Andorra | 0.008 (0.006,0.012) | 15.111 (10.801,20.485) | 0.013 (0.009,0.018) | 8.368 (5.874,11.293) | -2.021 (-2.264,-1.836) | 0.007 (0.005,0.01) | 13.321 (9.575,17.965) | 0.011 (0.008,0.015) | 6.801 (4.836,9.232) | -2.236 (-2.408,-2.079) | 0.168 (0.118,0.23) | 289.59 (204.151,395.3) | 0.215 (0.147,0.294) | 140.993 (96.209,193.21) | -2.453 (-2.691,-2.287) |
| Angola | 0.525 (0.371,0.672) | 13.267 (9.515,16.684) | 0.983 (0.711,1.256) | 8.395 (6.124,10.626) | -1.437 (-1.524,-1.339) | 0.523 (0.369,0.666) | 13.926 (10.064,17.406) | 0.966 (0.696,1.241) | 8.778 (6.377,11.063) | -1.449 (-1.536,-1.353) | 16.32 (11.589,21.025) | 359.049 (254.881,456.593) | 29.449 (21.085,38.13) | 214.864 (155.009,275.865) | -1.685 (-1.817,-1.593) |
| Antigua and Barbuda | 0.009 (0.009,0.01) | 17.157 (15.786,18.598) | 0.01 (0.01,0.011) | 10.128 (9.271,11.04) | -1.708 (-2.047,-1.423) | 0.01 (0.009,0.01) | 17.276 (15.91,18.701) | 0.01 (0.009,0.011) | 10.027 (9.142,10.942) | -1.734 (-2.156,-1.398) | 0.21 (0.194,0.227) | 400.427 (368.185,433.037) | 0.234 (0.213,0.254) | 216.345 (198.028,234.698) | -1.966 (-2.276,-1.702) |
| Argentina | 4.522 (4.207,4.836) | 14.155 (13.192,15.157) | 4.96 (4.464,5.409) | 8.855 (7.988,9.654) | -1.455 (-1.564,-1.364) | 4.48 (4.187,4.794) | 14.196 (13.262,15.203) | 4.744 (4.254,5.186) | 8.387 (7.524,9.167) | -1.645 (-1.749,-1.555) | 107.142 (100.076,114.599) | 331.06 (309.243,353.723) | 107.976 (98.422,117.629) | 197.501 (180.345,214.948) | -1.599 (-1.707,-1.489) |
| Armenia | 0.616 (0.583,0.652) | 22.175 (20.784,23.615) | 0.486 (0.429,0.557) | 11.192 (9.874,12.818) | -2.225 (-2.480,-2.048) | 0.595 (0.559,0.631) | 21.826 (20.392,23.306) | 0.469 (0.411,0.539) | 10.789 (9.446,12.386) | -2.269 (-2.489,-2.099) | 17.234 (16.385,18.117) | 588.594 (556.78,621.032) | 11.096 (9.812,12.754) | 259.379 (229.402,297.958) | -2.720 (-2.947,-2.561) |
| Australia | 1.933 (1.799,2.054) | 9.879 (9.19,10.491) | 2.608 (2.296,2.871) | 5.744 (5.135,6.295) | -1.721 (-1.775,-1.655) | 1.431 (1.329,1.521) | 7.365 (6.825,7.823) | 1.796 (1.573,1.961) | 3.78 (3.342,4.117) | -2.127 (-2.180,-2.077) | 31.479 (29.434,33.454) | 162.336 (151.732,172.147) | 34.495 (31.141,37.489) | 81.328 (74.105,88.243) | -2.210 (-2.260,-2.163) |
| Austria | 2.334 (2.172,2.484) | 19.276 (17.945,20.5) | 1.223 (1.089,1.347) | 6.688 (6.022,7.361) | -3.325 (-3.419,-3.247) | 2.026 (1.887,2.153) | 16.473 (15.363,17.459) | 0.891 (0.791,0.985) | 4.602 (4.148,5.087) | -4.002 (-4.069,-3.944) | 40.433 (38.01,42.729) | 351.711 (331.401,370.92) | 17.05 (15.519,18.675) | 100.713 (91.878,110.14) | -3.905 (-3.988,-3.841) |
| Azerbaijan | 1.311 (1.073,1.541) | 25.633 (20.94,30.123) | 1.461 (1.125,1.974) | 14.403 (11.277,19.205) | -1.865 (-1.950,-1.800) | 1.294 (1.057,1.525) | 25.857 (21.133,30.443) | 1.404 (1.082,1.898) | 14.327 (11.248,19.172) | -1.909 (-1.995,-1.841) | 38.154 (31.198,44.783) | 702.38 (573.074,827.673) | 38.53 (29.426,52.567) | 350.127 (267.454,474.832) | -2.258 (-2.374,-2.166) |
| Bahamas | 0.022 (0.02,0.023) | 13.727 (12.667,14.881) | 0.036 (0.028,0.044) | 8.893 (7.152,10.818) | -1.100 (-1.244,-0.958) | 0.021 (0.019,0.023) | 13.666 (12.568,14.788) | 0.034 (0.027,0.042) | 8.8 (7.124,10.692) | -1.127 (-1.284,-0.968) | 0.585 (0.538,0.635) | 348.684 (321.386,379.081) | 0.905 (0.721,1.124) | 213.721 (170.69,263.876) | -1.339 (-1.479,-1.201) |
| Bahrain | 0.021 (0.017,0.025) | 13.574 (11.269,16.303) | 0.051 (0.041,0.062) | 6.994 (5.583,8.539) | -2.193 (-2.319,-2.087) | 0.02 (0.017,0.024) | 14.211 (11.75,16.944) | 0.044 (0.035,0.053) | 6.789 (5.441,8.31) | -2.410 (-2.556,-2.273) | 0.571 (0.478,0.678) | 304.763 (254.316,363.854) | 1.23 (0.979,1.502) | 132.85 (105.489,162.008) | -2.693 (-2.790,-2.613) |
| Bangladesh | 4.805 (3.274,5.927) | 9.854 (6.757,12.153) | 7.372 (5.007,9.488) | 5.389 (3.65,6.906) | -1.871 (-1.986,-1.693) | 4.8 (3.288,5.908) | 10.107 (6.892,12.472) | 7.243 (4.954,9.344) | 5.437 (3.702,6.952) | -1.908 (-2.032,-1.699) | 145.1 (97.659,179.537) | 275.276 (186.628,339.875) | 191.348 (132.084,248.025) | 133.04 (91.928,172.786) | -2.212 (-2.302,-2.129) |
| Barbados | 0.051 (0.047,0.056) | 17.021 (15.758,18.458) | 0.051 (0.039,0.063) | 9.896 (7.647,12.378) | -1.686 (-1.937,-1.505) | 0.053 (0.049,0.057) | 17.261 (15.995,18.658) | 0.051 (0.039,0.063) | 9.765 (7.528,12.081) | -1.665 (-1.897,-1.481) | 1.096 (1.016,1.188) | 387.864 (359.542,419.556) | 1.065 (0.8,1.346) | 213.668 (160.519,271.096) | -1.809 (-2.026,-1.652) |
| Belarus | 4.438 (4.047,4.862) | 34.281 (31.263,37.585) | 2.413 (1.913,2.917) | 15.356 (12.16,18.585) | -2.627 (-2.951,-2.323) | 4.051 (3.702,4.423) | 31.266 (28.566,34.174) | 1.969 (1.551,2.373) | 12.384 (9.742,14.929) | -3.026 (-3.447,-2.683) | 108.945 (99.454,119.181) | 846.796 (773.412,926.721) | 49.527 (38.412,60.674) | 324.568 (251.837,398.315) | -3.127 (-3.491,-2.825) |
| Belgium | 2.012 (1.836,2.172) | 12.883 (11.793,13.857) | 1.285 (1.126,1.418) | 5.375 (4.803,5.9) | -2.789 (-2.886,-2.680) | 1.889 (1.72,2.048) | 11.975 (10.921,12.926) | 1.133 (0.985,1.255) | 4.464 (3.942,4.927) | -3.124 (-3.207,-3.037) | 36.788 (34.162,39.307) | 245.904 (229.353,261.876) | 20.751 (18.57,22.733) | 95.622 (86.744,104.54) | -3.151 (-3.338,-3.035) |
| Belize | 0.011 (0.01,0.012) | 11.853 (11.019,12.827) | 0.028 (0.024,0.032) | 9.337 (8.223,10.692) | -0.639 (-0.822,-0.463) | 0.011 (0.011,0.012) | 12.205 (11.348,13.213) | 0.027 (0.024,0.031) | 9.375 (8.202,10.77) | -0.717 (-0.905,-0.530) | 0.278 (0.26,0.3) | 287.829 (268.745,310.501) | 0.726 (0.635,0.833) | 225.008 (197.973,258.812) | -0.668 (-0.835,-0.506) |
| Benin | 0.234 (0.185,0.282) | 11.911 (9.514,14.301) | 0.454 (0.313,0.569) | 9.139 (6.425,11.42) | -0.866 (-0.902,-0.834) | 0.242 (0.194,0.292) | 12.631 (10.178,15.203) | 0.461 (0.326,0.58) | 9.701 (7.021,12.065) | -0.875 (-0.906,-0.847) | 6.377 (5.003,7.725) | 304.283 (241.252,367.092) | 12.48 (8.582,15.924) | 222.722 (155.659,280.961) | -1.016 (-1.050,-0.987) |
| Bermuda | 0.008 (0.008,0.009) | 13.427 (12.428,14.463) | 0.008 (0.007,0.01) | 5.629 (4.769,6.953) | -2.964 (-3.097,-2.834) | 0.008 (0.007,0.009) | 13.114 (12.128,14.122) | 0.007 (0.006,0.009) | 4.879 (4.135,5.99) | -3.293 (-3.431,-3.167) | 0.186 (0.172,0.2) | 293.712 (271.96,317.089) | 0.136 (0.114,0.167) | 104.555 (87.381,128.656) | -3.450 (-3.588,-3.339) |
| Bhutan | 0.021 (0.013,0.029) | 8.095 (5.388,11.159) | 0.033 (0.023,0.045) | 5.523 (3.851,7.488) | -1.216 (-1.241,-1.183) | 0.021 (0.014,0.029) | 8.325 (5.594,11.487) | 0.033 (0.023,0.045) | 5.633 (3.976,7.589) | -1.230 (-1.253,-1.202) | 0.656 (0.429,0.918) | 225.034 (147.59,313.632) | 0.851 (0.58,1.164) | 134.109 (92.194,182.476) | -1.656 (-1.685,-1.621) |
| Bolivia  (Plurinational State of) | 1.493 (1.235,1.8) | 47.961 (39.765,57.57) | 2.696 (2.024,3.581) | 30.813 (23.131,40.509) | -1.421 (-1.447,-1.404) | 1.541 (1.276,1.859) | 51.205 (42.545,61.761) | 2.75 (2.062,3.63) | 32.41 (24.486,42.162) | -1.472 (-1.497,-1.453) | 41.148 (33.872,50.123) | 1208.505 (1000.541,1464.426) | 66.823 (49.378,89.01) | 714.431 (533.703,949.639) | -1.692 (-1.715,-1.670) |
| Bosnia and Herzegovina | 0.557 (0.474,0.657) | 13.745 (11.673,16.237) | 0.571 (0.441,0.731) | 9.115 (7.07,11.63) | -1.237 (-1.359,-1.111) | 0.545 (0.463,0.644) | 13.812 (11.742,16.37) | 0.548 (0.424,0.703) | 8.672 (6.702,11.11) | -1.428 (-1.544,-1.271) | 14.747 (12.451,17.279) | 340.321 (289.915,400.789) | 12.052 (9.249,15.571) | 199.662 (152.385,257.125) | -1.627 (-1.766,-1.464) |
| Botswana | 0.056 (0.038,0.072) | 10.282 (7.088,13.083) | 0.078 (0.055,0.101) | 5.501 (3.929,7.023) | -2.032 (-2.137,-1.939) | 0.056 (0.039,0.072) | 10.762 (7.517,13.753) | 0.077 (0.055,0.1) | 5.745 (4.117,7.423) | -2.035 (-2.127,-1.952) | 1.607 (1.11,2.123) | 264.072 (185.261,343.549) | 2.143 (1.503,2.826) | 133.766 (95.811,174.468) | -2.191 (-2.276,-2.116) |
| Brazil | 16.642 (15.894,17.271) | 19.143 (18.135,19.933) | 25.109 (23.427,26.398) | 10.024 (9.326,10.547) | -2.107 (-2.157,-2.050) | 16.579 (15.815,17.229) | 19.787 (18.686,20.642) | 24.375 (22.601,25.67) | 9.808 (9.071,10.342) | -2.268 (-2.321,-2.201) | 443.64 (427.05,458.959) | 469.392 (449.52,486.833) | 607.323 (576.036,635.516) | 238.766 (226.17,249.973) | -2.164 (-2.263,-2.096) |
| Brunei Darussalam | 0.029 (0.022,0.035) | 27.226 (20.017,32.596) | 0.045 (0.036,0.054) | 12.96 (10.634,15.43) | -2.402 (-2.511,-2.309) | 0.024 (0.018,0.029) | 23.573 (17.358,28.185) | 0.032 (0.026,0.038) | 10.144 (8.289,12.09) | -2.708 (-2.805,-2.618) | 0.712 (0.527,0.866) | 568.749 (417.205,683.898) | 0.928 (0.751,1.13) | 236.15 (193.202,283.777) | -2.802 (-2.884,-2.738) |
| Bulgaria | 2.654 (2.393,2.954) | 21.975 (19.973,24.298) | 1.699 (1.424,2.003) | 12.055 (10.074,14.275) | -1.802 (-1.950,-1.658) | 2.667 (2.39,2.978) | 22.594 (20.539,24.973) | 1.614 (1.369,1.881) | 11.294 (9.531,13.173) | -2.153 (-2.291,-1.993) | 66.236 (59.75,73.842) | 540.708 (489.236,600.239) | 35.847 (30.003,42.271) | 274.372 (228.571,325.403) | -2.126 (-2.296,-2.005) |
| Burkina Faso | 0.571 (0.431,0.694) | 13.608 (10.403,16.507) | 1 (0.676,1.258) | 11.201 (7.747,13.959) | -0.619 (-0.664,-0.586) | 0.584 (0.442,0.709) | 14.434 (11.187,17.469) | 1.021 (0.702,1.285) | 11.924 (8.392,14.839) | -0.603 (-0.664,-0.553) | 15.914 (11.923,19.527) | 347.694 (262.788,424.39) | 27.463 (18.687,35.045) | 275.669 (188.219,348.929) | -0.736 (-0.764,-0.707) |
| Burundi | 0.29 (0.199,0.367) | 12.181 (8.46,15.319) | 0.371 (0.264,0.476) | 7.497 (5.402,9.492) | -1.560 (-1.580,-1.541) | 0.294 (0.205,0.372) | 12.713 (8.95,15.887) | 0.37 (0.266,0.475) | 7.896 (5.75,10.093) | -1.530 (-1.560,-1.499) | 8.637 (5.863,11.099) | 334.85 (229.159,426.664) | 11.273 (7.949,14.671) | 195.946 (140.484,251.7) | -1.722 (-1.747,-1.698) |
| Cabo Verde | 0.072 (0.056,0.086) | 30.942 (24.067,36.983) | 0.101 (0.079,0.134) | 23.466 (18.381,30.817) | -0.888 (-1.158,-0.644) | 0.077 (0.06,0.092) | 32.582 (25.365,38.99) | 0.102 (0.08,0.131) | 24.117 (19.033,31.045) | -0.971 (-1.201,-0.739) | 1.668 (1.333,2.014) | 738.906 (591.119,893.979) | 2.303 (1.773,3.101) | 511.196 (396.103,675.945) | -1.199 (-1.412,-0.989) |
| Cambodia | 0.749 (0.561,0.95) | 16.06 (12.041,20.465) | 1.288 (0.95,1.685) | 10.473 (7.96,13.498) | -1.368 (-1.379,-1.354) | 0.747 (0.56,0.943) | 16.673 (12.501,21.201) | 1.229 (0.915,1.582) | 10.461 (8.064,13.382) | -1.490 (-1.505,-1.471) | 22.941 (17.346,28.996) | 443.219 (333.749,560.276) | 34.561 (25.006,45.438) | 257.374 (188.583,334.736) | -1.740 (-1.754,-1.725) |
| Cameroon | 0.523 (0.405,0.627) | 12.12 (9.494,14.526) | 1.2 (0.746,1.611) | 9.862 (6.406,13.008) | -0.664 (-0.694,-0.637) | 0.53 (0.416,0.636) | 12.82 (10.192,15.431) | 1.197 (0.752,1.596) | 10.377 (6.776,13.731) | -0.677 (-0.705,-0.651) | 14.893 (11.531,17.991) | 308.724 (241.597,371.346) | 33.966 (20.757,46.352) | 242.825 (152.148,325.187) | -0.782 (-0.806,-0.763) |
| Canada | 3.526 (3.258,3.773) | 10.807 (9.992,11.55) | 4.647 (4.134,5.148) | 6.503 (5.835,7.191) | -1.572 (-1.721,-1.460) | 2.513 (2.326,2.688) | 7.722 (7.15,8.255) | 2.912 (2.563,3.242) | 3.868 (3.447,4.271) | -2.191 (-2.349,-2.086) | 55.323 (51.846,58.813) | 171.845 (161.067,182.511) | 56.494 (51.325,61.956) | 84.427 (77.232,91.924) | -2.152 (-2.280,-2.032) |
| Central African Republic | 0.199 (0.144,0.244) | 16.886 (12.317,20.578) | 0.29 (0.2,0.385) | 12.537 (8.87,15.942) | -0.955 (-0.993,-0.924) | 0.198 (0.144,0.243) | 17.655 (12.999,21.418) | 0.287 (0.198,0.377) | 13.164 (9.345,16.568) | -0.940 (-0.974,-0.912) | 6.213 (4.487,7.704) | 467.661 (340.074,572.355) | 9.314 (6.323,12.538) | 343.784 (238.37,448.929) | -0.971 (-1.011,-0.941) |
| Chad | 0.314 (0.246,0.385) | 11.306 (8.875,13.849) | 0.718 (0.51,0.928) | 12.749 (9.111,16.266) | 0.396 (0.360,0.447) | 0.326 (0.256,0.401) | 12.008 (9.486,14.786) | 0.73 (0.524,0.941) | 13.554 (9.866,17.237) | 0.400 (0.367,0.450) | 8.563 (6.662,10.497) | 291.576 (227.235,356.175) | 20.522 (14.569,26.747) | 322.388 (231.128,417.184) | 0.351 (0.295,0.400) |
| Chile | 3.386 (3.134,3.638) | 34.325 (31.74,36.93) | 4.366 (3.907,4.823) | 16.944 (15.158,18.719) | -2.201 (-2.257,-2.149) | 3.308 (3.064,3.542) | 34.232 (31.59,36.704) | 3.933 (3.503,4.342) | 15.151 (13.51,16.73) | -2.545 (-2.603,-2.495) | 79.688 (74.249,85.565) | 776.913 (722.175,835.189) | 85.068 (76.888,93.897) | 335.426 (302.985,370.467) | -2.640 (-2.692,-2.594) |
| China | 407.471 (337.565,477.569) | 48.026 (40.215,56.685) | 611.799 (471.966,765.562) | 29.053 (22.423,36.2) | -1.610 (-1.648,-1.560) | 374.066 (310.921,442.251) | 46.048 (38.88,54.434) | 445.013 (344.736,555.834) | 21.509 (16.663,26.611) | -2.442 (-2.494,-2.394) | 10773.457 (8850.977,12638.919) | 1181.613 (978.381,1390.895) | 10642.127 (8222.106,13383.779) | 501.26 (387.291,627.976) | -2.750 (-2.800,-2.706) |
| Colombia | 5.272 (5.009,5.55) | 30.634 (28.928,32.279) | 7.913 (6.594,9.407) | 14.39 (11.995,17.122) | -2.484 (-2.633,-2.337) | 5.262 (4.995,5.541) | 31.591 (29.875,33.295) | 7.449 (6.186,8.862) | 13.514 (11.234,16.083) | -2.791 (-2.945,-2.637) | 138.8 (131.987,146.096) | 739.48 (702.402,778.058) | 181.689 (150.279,218.359) | 330.422 (273.367,396.494) | -2.642 (-2.794,-2.486) |
| Comoros | 0.019 (0.013,0.025) | 9.492 (6.571,12.363) | 0.032 (0.024,0.043) | 6.69 (5.02,8.736) | -1.177 (-1.252,-1.087) | 0.019 (0.013,0.025) | 9.896 (6.931,12.87) | 0.033 (0.024,0.043) | 6.99 (5.277,9.071) | -1.160 (-1.229,-1.078) | 0.569 (0.377,0.758) | 255.604 (173.242,334.399) | 0.909 (0.67,1.208) | 170.642 (127.376,224.925) | -1.345 (-1.460,-1.200) |
| Congo | 0.154 (0.118,0.187) | 14.477 (11.228,17.541) | 0.231 (0.162,0.298) | 8.536 (6.193,10.609) | -1.686 (-1.727,-1.641) | 0.154 (0.12,0.187) | 15.137 (11.883,18.286) | 0.226 (0.158,0.292) | 8.88 (6.439,10.956) | -1.699 (-1.736,-1.659) | 4.615 (3.563,5.634) | 390.91 (303.957,474.963) | 6.886 (4.7,9.149) | 218.1 (153.134,280.783) | -1.863 (-1.920,-1.800) |
| Cook Islands | 0.001 (0.001,0.002) | 11.598 (9.611,14.496) | 0.002 (0.001,0.002) | 7.024 (5.553,8.835) | -1.618 (-1.650,-1.591) | 0.001 (0.001,0.002) | 11.736 (9.819,14.527) | 0.002 (0.001,0.002) | 6.192 (4.857,7.73) | -2.051 (-2.085,-2.021) | 0.037 (0.03,0.047) | 279.055 (229.591,352.252) | 0.036 (0.028,0.045) | 146.554 (113.766,187.229) | -2.056 (-2.087,-2.029) |
| Costa Rica | 0.636 (0.59,0.677) | 36.627 (33.939,39.041) | 0.985 (0.865,1.121) | 17.934 (15.758,20.42) | -2.246 (-2.418,-2.073) | 0.616 (0.57,0.658) | 36.139 (33.439,38.598) | 0.916 (0.803,1.043) | 16.622 (14.604,18.925) | -2.416 (-2.583,-2.244) | 14.972 (13.937,15.95) | 824.946 (766.525,879.274) | 21.741 (18.924,24.718) | 395.261 (344.508,449.457) | -2.348 (-2.513,-2.181) |
| Côte d'Ivoire | 0.169 (0.126,0.206) | 4.294 (3.251,5.16) | 0.368 (0.254,0.487) | 3.323 (2.415,4.276) | -0.848 (-1.006,-0.687) | 0.167 (0.127,0.204) | 4.527 (3.454,5.45) | 0.362 (0.251,0.477) | 3.469 (2.531,4.478) | -1.079 (-1.183,-0.987) | 5.135 (3.821,6.355) | 110.635 (83.822,134.249) | 10.788 (7.141,14.452) | 83.246 (57.815,109.522) | -0.888 (-1.046,-0.739) |
| Croatia | 1.56 (1.402,1.723) | 26.471 (23.774,29.3) | 0.968 (0.813,1.144) | 10.699 (8.954,12.625) | -1.867 (-1.949,-1.787) | 1.391 (1.252,1.542) | 23.988 (21.535,26.665) | 0.789 (0.666,0.931) | 8.468 (7.131,10.023) | -2.361 (-2.457,-2.274) | 33.054 (29.99,36.367) | 541.944 (491.388,596.493) | 15.763 (13.243,18.792) | 186.767 (157.071,222.858) | -2.324 (-2.414,-2.252) |
| Cuba | 0.818 (0.76,0.882) | 7.999 (7.431,8.621) | 1.162 (0.984,1.349) | 5.937 (5.031,6.906) | -2.990 (-3.150,-2.856) | 0.799 (0.74,0.861) | 7.893 (7.319,8.487) | 1.092 (0.926,1.266) | 5.488 (4.633,6.378) | -3.598 (-3.728,-3.470) | 18.599 (17.259,20.028) | 180.312 (167.409,194.447) | 24.077 (20.375,27.906) | 126.854 (107.249,147.322) | -3.706 (-3.855,-3.580) |
| Cyprus | 0.082 (0.069,0.11) | 11.812 (9.924,15.825) | 0.136 (0.106,0.164) | 6.764 (5.215,8.089) | -0.820 (-0.876,-0.765) | 0.079 (0.066,0.106) | 11.957 (9.96,16.164) | 0.115 (0.09,0.139) | 5.892 (4.58,7.053) | -0.846 (-0.906,-0.787) | 1.698 (1.426,2.228) | 227.17 (192.555,302.264) | 2.24 (1.746,2.729) | 111.948 (86.231,135.106) | -0.905 (-0.961,-0.853) |
| Czechia | 2.541 (2.322,2.8) | 18.238 (16.727,20.004) | 1.518 (1.297,1.758) | 6.998 (5.947,8.128) | -2.862 (-2.999,-2.727) | 2.377 (2.173,2.612) | 17.027 (15.624,18.66) | 1.21 (1.035,1.404) | 5.458 (4.65,6.335) | -3.310 (-3.450,-3.169) | 52.952 (48.466,58.155) | 387.558 (355.484,425.239) | 24.911 (21.164,29.002) | 122.352 (103.815,142.959) | -3.394 (-3.534,-3.258) |
| Democratic People's Republic of Korea | 4.622 (3.365,6.013) | 27.885 (20.621,35.802) | 8.05 (6.046,10.116) | 24.018 (18.194,30.163) | -0.478 (-0.490,-0.467) | 4.428 (3.195,5.749) | 27.76 (20.485,35.602) | 7.135 (5.391,9.04) | 21.57 (16.407,27.084) | -0.807 (-0.816,-0.799) | 132.583 (94.428,174.137) | 739.729 (531.876,961.875) | 200.037 (148.083,255.936) | 585.749 (437.295,748.459) | -0.744 (-0.753,-0.736) |
| Democratic Republic of the Congo | 1.594 (1.165,2.009) | 10.416 (7.56,12.93) | 3.049 (2.177,3.899) | 8.4 (6.112,10.654) | -0.723 (-0.771,-0.671) | 1.598 (1.167,2.006) | 11.012 (8.013,13.631) | 3.02 (2.178,3.875) | 8.803 (6.417,11.203) | -0.750 (-0.795,-0.700) | 47.536 (34.971,59.998) | 273.446 (199.923,341.967) | 90.516 (65.007,117.102) | 216.057 (155.684,277.088) | -0.791 (-0.847,-0.738) |
| Denmark | 0.866 (0.805,0.924) | 10.718 (9.997,11.414) | 0.747 (0.67,0.826) | 6.269 (5.608,6.923) | -1.755 (-1.877,-1.660) | 0.76 (0.703,0.808) | 9.174 (8.537,9.748) | 0.57 (0.511,0.626) | 4.602 (4.148,5.045) | -2.262 (-2.387,-2.162) | 16.079 (15.068,17.1) | 210.988 (197.374,224.743) | 11.15 (10.098,12.225) | 100.221 (90.284,109.998) | -2.404 (-2.557,-2.304) |
| Djibouti | 0.012 (0.009,0.016) | 8.71 (6.42,11.482) | 0.046 (0.032,0.064) | 7.246 (5.132,9.711) | -0.601 (-0.637,-0.562) | 0.012 (0.008,0.016) | 9.105 (6.763,11.907) | 0.045 (0.031,0.062) | 7.565 (5.357,10.144) | -0.600 (-0.630,-0.567) | 0.378 (0.266,0.51) | 228.38 (164.544,304.292) | 1.372 (0.94,1.918) | 182.704 (127.114,250.03) | -0.729 (-0.775,-0.683) |
| Dominica | 0.017 (0.014,0.019) | 27.814 (23.806,32.186) | 0.016 (0.013,0.02) | 19.633 (16.176,24.417) | -1.122 (-1.162,-1.089) | 0.017 (0.015,0.02) | 29.16 (24.924,33.93) | 0.016 (0.014,0.02) | 20.288 (16.742,25.074) | -1.165 (-1.201,-1.132) | 0.38 (0.329,0.44) | 644.975 (559.339,742.465) | 0.379 (0.307,0.478) | 453.288 (370.098,569.697) | -1.133 (-1.170,-1.104) |
| Dominican Republic | 0.289 (0.244,0.355) | 7.978 (6.793,9.797) | 0.696 (0.538,0.887) | 6.938 (5.361,8.847) | -0.370 (-0.458,-0.301) | 0.291 (0.244,0.359) | 8.445 (7.057,10.353) | 0.695 (0.536,0.885) | 6.998 (5.386,8.897) | -0.503 (-0.690,-0.306) | 8.053 (6.735,9.953) | 199.623 (166.929,247.194) | 17.687 (13.732,22.395) | 171.855 (133.307,217.764) | -0.370 (-0.448,-0.301) |
| Ecuador | 1.539 (1.464,1.625) | 29.649 (28.048,31.341) | 2.99 (2.347,3.752) | 18.473 (14.528,23.057) | -1.650 (-1.927,-1.422) | 1.584 (1.506,1.675) | 31.396 (29.73,33.21) | 2.97 (2.337,3.714) | 18.642 (14.753,23.191) | -1.766 (-2.035,-1.544) | 40.304 (38.22,42.46) | 719.174 (681.873,759.858) | 70.258 (54.873,89.2) | 422.667 (330.613,535.453) | -1.927 (-2.212,-1.705) |
| Egypt | 1.203 (1.042,1.741) | 4.349 (3.694,6.761) | 4.326 (2.741,5.489) | 7.193 (4.707,8.908) | 1.658 (1.494,1.825) | 1.173 (1.009,1.714) | 4.506 (3.82,7.087) | 4.057 (2.586,5.158) | 7.205 (4.741,8.92) | 1.558 (1.391,1.737) | 37.006 (32.365,50.603) | 116.454 (100.703,168.709) | 116.055 (71.435,149.088) | 168.312 (106.758,213.405) | 1.274 (1.125,1.421) |
| El Salvador | 0.571 (0.514,0.701) | 19.049 (17.076,23.358) | 1.133 (0.895,1.417) | 18.164 (14.349,22.779) | -0.034 (-0.278,0.251) | 0.582 (0.522,0.714) | 19.724 (17.618,24.186) | 1.112 (0.878,1.391) | 17.61 (13.9,22.071) | -0.266 (-0.514,0.039) | 15.522 (14.087,19.131) | 491.479 (444.649,605.105) | 26.546 (20.84,33.493) | 432.357 (338.627,545.461) | -0.285 (-0.556,0.044) |
| Equatorial Guinea | 0.029 (0.02,0.037) | 14.652 (10.434,18.566) | 0.033 (0.021,0.047) | 6.413 (4.245,9.019) | -2.604 (-2.677,-2.550) | 0.029 (0.02,0.037) | 15.343 (10.965,19.413) | 0.031 (0.02,0.046) | 6.58 (4.403,9.223) | -2.639 (-2.700,-2.579) | 0.868 (0.611,1.135) | 400.649 (283.612,516.934) | 0.934 (0.579,1.401) | 156.166 (100.369,226.467) | -2.969 (-3.044,-2.910) |
| Eritrea | 0.167 (0.122,0.206) | 13.516 (10.111,16.327) | 0.278 (0.196,0.36) | 9.706 (7.125,12.151) | -1.107 (-1.147,-1.066) | 0.163 (0.118,0.199) | 13.972 (10.505,16.767) | 0.273 (0.194,0.35) | 10.157 (7.493,12.677) | -1.064 (-1.108,-1.010) | 5.552 (3.964,6.875) | 382.109 (281.556,465.057) | 8.754 (6.04,11.535) | 258.282 (185.183,329.305) | -1.307 (-1.353,-1.258) |
| Estonia | 0.586 (0.539,0.642) | 28.733 (26.448,31.434) | 0.328 (0.278,0.382) | 12.5 (10.532,14.543) | -2.863 (-3.030,-2.712) | 0.526 (0.484,0.575) | 25.69 (23.669,28.038) | 0.273 (0.23,0.316) | 9.852 (8.291,11.458) | -3.341 (-3.485,-3.210) | 13.437 (12.362,14.676) | 665.663 (611.884,726.583) | 5.635 (4.721,6.56) | 230.022 (191.648,268.743) | -3.569 (-4.063,-3.332) |
| Eswatini | 0.031 (0.023,0.039) | 10.923 (7.786,13.644) | 0.054 (0.036,0.073) | 9.393 (6.498,12.363) | -0.445 (-0.495,-0.398) | 0.031 (0.023,0.039) | 11.383 (8.249,14.109) | 0.053 (0.035,0.071) | 9.634 (6.622,12.587) | -0.477 (-0.519,-0.435) | 0.903 (0.657,1.148) | 282.172 (205.643,354.679) | 1.615 (1.041,2.205) | 248.35 (163.39,331.902) | -0.399 (-0.443,-0.358) |
| Ethiopia | 3.224 (2.373,3.951) | 15.246 (11.381,18.784) | 2.859 (2.259,3.707) | 6.368 (5.068,8.167) | -2.772 (-2.798,-2.749) | 3.202 (2.355,3.92) | 15.832 (11.842,19.54) | 2.845 (2.248,3.664) | 6.642 (5.3,8.481) | -2.779 (-2.805,-2.749) | 103.675 (74.955,126.591) | 436.494 (321.822,536.014) | 84.372 (65.778,109.428) | 164.777 (129.202,213.252) | -3.090 (-3.116,-3.068) |
| Fiji | 0.037 (0.02,0.046) | 10.177 (5.505,12.482) | 0.058 (0.027,0.08) | 8.16 (3.866,10.991) | -0.706 (-0.836,-0.595) | 0.036 (0.019,0.044) | 10.563 (5.727,12.906) | 0.056 (0.027,0.076) | 8.349 (4.151,10.985) | -0.751 (-0.867,-0.646) | 1.148 (0.615,1.43) | 265.448 (143.186,326.064) | 1.571 (0.752,2.186) | 196.616 (95.743,268.088) | -0.923 (-1.029,-0.821) |
| Finland | 1.147 (1.069,1.221) | 15.97 (14.847,17.006) | 0.761 (0.644,0.852) | 5.887 (5.142,6.557) | -3.212 (-3.289,-3.156) | 0.947 (0.879,1.012) | 13.078 (12.133,13.991) | 0.571 (0.479,0.636) | 4.168 (3.578,4.656) | -3.696 (-3.814,-3.625) | 20.595 (19.351,21.877) | 295.537 (277.891,313.773) | 10.346 (9.075,11.537) | 89.044 (79.477,98.985) | -3.860 (-3.950,-3.804) |
| France | 9.998 (9.261,10.636) | 11.824 (11.037,12.541) | 9.367 (8.196,10.43) | 6.565 (5.848,7.274) | -1.862 (-1.917,-1.822) | 9.297 (8.593,9.896) | 10.803 (10.048,11.478) | 6.84 (5.93,7.601) | 4.485 (3.97,4.971) | -2.756 (-2.841,-2.704) | 182.553 (171.322,193.178) | 228.455 (215.297,242.191) | 126.45 (112.576,140.144) | 100.957 (90.691,112.073) | -2.576 (-2.623,-2.546) |
| Gabon | 0.067 (0.049,0.084) | 11.963 (8.732,14.884) | 0.079 (0.053,0.103) | 7.69 (5.283,9.73) | -1.365 (-1.415,-1.315) | 0.069 (0.05,0.085) | 12.511 (9.21,15.521) | 0.077 (0.053,0.1) | 7.925 (5.576,9.93) | -1.411 (-1.457,-1.363) | 1.848 (1.359,2.299) | 311.575 (229.032,388.467) | 2.168 (1.438,2.885) | 189.509 (128.256,246.023) | -1.543 (-1.598,-1.484) |
| Gambia | 0.014 (0.01,0.018) | 4.018 (2.961,5.009) | 0.033 (0.025,0.043) | 3.41 (2.556,4.356) | -0.445 (-0.605,-0.309) | 0.014 (0.01,0.018) | 4.19 (3.1,5.217) | 0.033 (0.025,0.043) | 3.535 (2.643,4.535) | -0.465 (-0.616,-0.329) | 0.412 (0.284,0.524) | 104.534 (74.447,131.344) | 0.942 (0.682,1.26) | 86.262 (63.284,112.449) | -0.527 (-0.724,-0.351) |
| Georgia | 1.123 (1.026,1.219) | 17.861 (16.349,19.323) | 0.72 (0.644,0.801) | 12.209 (10.882,13.581) | -1.427 (-1.856,-1.135) | 1.086 (0.994,1.176) | 17.379 (15.981,18.803) | 0.712 (0.634,0.795) | 11.936 (10.648,13.326) | -1.422 (-1.823,-1.127) | 30.018 (27.355,32.894) | 475.158 (434.923,519.707) | 16.684 (14.82,18.638) | 294.269 (260.993,329.144) | -1.728 (-2.094,-1.442) |
| Germany | 23.746 (21.716,25.501) | 18.362 (16.874,19.687) | 17.69 (15.56,19.71) | 9.247 (8.265,10.248) | -2.104 (-2.175,-2.027) | 19.153 (17.462,20.504) | 14.619 (13.389,15.636) | 11.834 (10.353,13.211) | 5.795 (5.167,6.422) | -2.910 (-2.977,-2.841) | 392.756 (362.928,419.373) | 319.407 (295.87,340.938) | 226.844 (203.816,251.308) | 131.163 (118.499,144.157) | -2.761 (-2.829,-2.690) |
| Ghana | 0.559 (0.413,0.696) | 9.243 (6.816,11.398) | 1.158 (0.856,1.482) | 7.352 (5.521,9.322) | -0.726 (-0.761,-0.694) | 0.561 (0.416,0.699) | 9.778 (7.207,11.971) | 1.158 (0.861,1.493) | 7.765 (5.812,9.867) | -0.732 (-0.770,-0.698) | 16.191 (11.958,20.512) | 234.437 (174.337,292.761) | 31.603 (23.191,41.088) | 175.38 (130.481,226.791) | -0.930 (-0.965,-0.900) |
| Global | 980.899 (891.307,1072.236) | 24.763 (22.58,27.002) | 1230.233 (1052.35,1409.97) | 14.328 (12.226,16.408) | -1.763 (-1.812,-1.728) | 854.185 (772.885,939.973) | 22.006 (20.028,24.187) | 954.374 (821.751,1089.577) | 11.199 (9.618,12.734) | -2.187 (-2.211,-2.164) | 23237.292 (20605.349,25526.194) | 559.721 (499.087,615.772) | 22786.633 (19576.344,26118.869) | 262.748 (226.079,301.024) | -2.424 (-2.452,-2.399) |
| Greece | 2.796 (2.615,2.948) | 18.473 (17.287,19.43) | 2.134 (1.9,2.305) | 8.703 (7.962,9.328) | -2.412 (-2.478,-2.340) | 2.482 (2.32,2.62) | 16.474 (15.355,17.346) | 1.937 (1.716,2.098) | 7.354 (6.678,7.906) | -2.562 (-2.649,-2.476) | 51.85 (49.026,54.466) | 349.087 (330.466,366.09) | 34.862 (31.814,37.479) | 160.719 (149.222,172.005) | -2.459 (-2.525,-2.392) |
| Greenland | 0.007 (0.006,0.009) | 20.756 (16.662,24.385) | 0.007 (0.005,0.008) | 9.539 (7.744,12.089) | -2.356 (-2.424,-2.284) | 0.007 (0.005,0.008) | 20.123 (16.394,23.5) | 0.006 (0.005,0.007) | 8.735 (7.1,11.167) | -2.553 (-2.619,-2.485) | 0.208 (0.165,0.247) | 517.147 (411.403,609.399) | 0.158 (0.127,0.202) | 216.89 (175.569,279.338) | -2.662 (-2.725,-2.600) |
| Grenada | 0.01 (0.009,0.012) | 14.298 (12.73,15.955) | 0.009 (0.008,0.011) | 8.476 (7.379,9.599) | -1.449 (-1.622,-1.275) | 0.011 (0.01,0.012) | 14.669 (13.111,16.352) | 0.009 (0.008,0.01) | 8.586 (7.495,9.672) | -1.674 (-1.936,-1.510) | 0.246 (0.219,0.275) | 360.512 (320.962,403.838) | 0.225 (0.193,0.26) | 194.99 (167.415,223.122) | -1.932 (-2.225,-1.664) |
| Guam | 0.007 (0.006,0.008) | 9.266 (8.024,11.176) | 0.012 (0.01,0.014) | 5.768 (4.68,6.796) | -1.516 (-1.729,-1.370) | 0.006 (0.005,0.007) | 9.378 (8.132,11.157) | 0.01 (0.008,0.012) | 4.918 (3.996,5.789) | -2.092 (-2.279,-1.935) | 0.176 (0.153,0.221) | 209.446 (183.414,256.557) | 0.286 (0.234,0.336) | 144.73 (117.986,169.861) | -1.244 (-1.392,-1.138) |
| Guatemala | 0.952 (0.909,1) | 29.804 (28.353,31.305) | 2.595 (2.226,3.021) | 23.687 (20.399,27.504) | -0.708 (-0.937,-0.458) | 0.971 (0.927,1.021) | 32.609 (30.949,34.233) | 2.598 (2.224,3.008) | 24.404 (21.034,28.039) | -0.885 (-1.121,-0.618) | 27.611 (26.397,28.849) | 733.03 (701.249,767.282) | 67.674 (57.431,78.765) | 581.431 (493.998,675.719) | -0.734 (-0.938,-0.507) |
| Guinea | 0.346 (0.276,0.441) | 10.526 (8.379,13.395) | 0.53 (0.388,0.698) | 9.356 (6.936,12.137) | -0.368 (-0.393,-0.347) | 0.356 (0.284,0.456) | 11.111 (8.884,14.292) | 0.537 (0.393,0.71) | 9.83 (7.316,12.771) | -0.382 (-0.406,-0.360) | 9.688 (7.76,12.135) | 277.811 (222.655,351.429) | 15.231 (10.912,20.394) | 242.514 (176.045,322.583) | -0.431 (-0.461,-0.407) |
| Guinea-Bissau | 0.075 (0.057,0.095) | 18.751 (14.351,23.298) | 0.11 (0.08,0.137) | 15.249 (11.304,18.609) | -0.671 (-0.689,-0.653) | 0.076 (0.058,0.095) | 19.662 (15.14,24.292) | 0.109 (0.08,0.135) | 16.106 (11.976,19.577) | -0.631 (-0.663,-0.606) | 2.259 (1.726,2.865) | 508.786 (388.854,640.618) | 3.36 (2.436,4.278) | 395.283 (289.24,489.967) | -0.816 (-0.840,-0.797) |
| Guyana | 0.05 (0.045,0.056) | 13.354 (11.968,14.845) | 0.05 (0.038,0.064) | 7.773 (6.077,9.862) | -1.563 (-1.793,-1.344) | 0.051 (0.046,0.057) | 13.989 (12.57,15.546) | 0.049 (0.038,0.062) | 7.975 (6.255,9.975) | -1.639 (-1.880,-1.411) | 1.396 (1.243,1.562) | 341.134 (304.577,379.775) | 1.377 (1.04,1.781) | 201.146 (153.518,258.509) | -1.491 (-1.720,-1.268) |
| Haiti | 0.866 (0.638,1.136) | 27.208 (20.387,35.298) | 1.245 (0.85,1.646) | 17.63 (12.305,23.223) | -1.375 (-1.403,-1.347) | 0.882 (0.653,1.156) | 28.926 (21.723,37.236) | 1.257 (0.866,1.659) | 18.732 (13.143,24.698) | -1.378 (-1.404,-1.352) | 25.63 (18.537,33.85) | 723.617 (532.775,950.982) | 36.462 (24.533,48.381) | 453.143 (311.119,598.665) | -1.484 (-1.516,-1.452) |
| Honduras | 0.348 (0.284,0.412) | 16.904 (13.821,19.979) | 1.191 (0.922,1.569) | 19.682 (15.381,25.747) | 0.536 (0.451,0.607) | 0.351 (0.289,0.415) | 17.808 (14.606,20.875) | 1.21 (0.944,1.579) | 20.762 (16.113,26.842) | 0.550 (0.465,0.623) | 10.134 (8.32,11.909) | 442.306 (363.637,521.35) | 30.384 (23.276,40.467) | 462.631 (358.884,610.642) | 0.180 (0.097,0.251) |
| Hungary | 2.958 (2.589,3.332) | 20.15 (17.682,22.713) | 1.503 (1.274,1.763) | 7.786 (6.619,9.124) | -3.030 (-3.128,-2.931) | 2.964 (2.582,3.337) | 20.266 (17.646,22.887) | 1.401 (1.194,1.641) | 7.089 (6.037,8.301) | -3.339 (-3.465,-3.226) | 68.487 (59.834,77.153) | 474.504 (416.641,535.461) | 30.521 (25.912,35.88) | 169.143 (143.767,198.736) | -3.325 (-3.469,-3.206) |
| Iceland | 0.046 (0.042,0.051) | 15.952 (14.452,17.704) | 0.033 (0.028,0.038) | 5.549 (4.74,6.366) | -3.443 (-3.543,-3.356) | 0.04 (0.036,0.045) | 13.657 (12.349,15.205) | 0.027 (0.023,0.031) | 4.359 (3.696,4.998) | -3.658 (-3.762,-3.589) | 0.841 (0.766,0.931) | 300.565 (273.545,333.352) | 0.503 (0.437,0.575) | 90.159 (78.64,103.117) | -3.895 (-4.018,-3.821) |
| India | 38.091 (32.484,49.779) | 7.753 (6.577,10.314) | 70.163 (61.019,86.919) | 5.826 (5.057,7.194) | -0.895 (-0.983,-0.779) | 37.351 (31.721,48.997) | 7.899 (6.689,10.552) | 68.517 (59.455,84.302) | 5.834 (5.061,7.152) | -0.947 (-1.043,-0.816) | 1176.742 (999.343,1512.842) | 217.782 (185.19,284.097) | 1893.706 (1648.775,2347.625) | 149.276 (129.848,184.697) | -1.219 (-1.287,-1.137) |
| Indonesia | 9.455 (7.342,11.665) | 9.308 (7.14,11.676) | 17.5 (13.968,22.635) | 7.505 (6.019,9.795) | -0.689 (-0.701,-0.677) | 9.278 (7.221,11.513) | 9.572 (7.271,12.05) | 16.481 (13.147,21.387) | 7.483 (6,9.789) | -0.788 (-0.799,-0.774) | 288.529 (225.392,351.637) | 252.951 (196.217,313.477) | 463.367 (368.892,589.871) | 178.94 (143.168,229.904) | -1.108 (-1.120,-1.096) |
| Iran  (Islamic Republic of) | 5.741 (4.088,6.402) | 23.137 (16.367,25.807) | 10.381 (6.995,11.531) | 13.906 (9.28,15.405) | -1.683 (-1.752,-1.639) | 5.609 (3.993,6.255) | 23.965 (16.911,26.767) | 9.576 (6.554,10.537) | 13.259 (8.978,14.608) | -1.929 (-1.989,-1.881) | 158.851 (115.664,176.727) | 563.11 (405.638,624.913) | 233.778 (164.155,257.564) | 292.594 (202.252,322.089) | -2.139 (-2.217,-2.088) |
| Iraq | 0.543 (0.431,0.732) | 6.578 (5.219,8.874) | 1.288 (0.944,1.688) | 5.425 (4.041,6.885) | -0.600 (-0.665,-0.532) | 0.534 (0.423,0.717) | 6.601 (5.24,8.888) | 1.186 (0.874,1.516) | 5.267 (3.938,6.66) | -0.692 (-0.764,-0.616) | 15.988 (12.57,21.181) | 179.832 (142.018,240.48) | 33.949 (24.789,44.402) | 126.712 (93.094,162.312) | -1.122 (-1.185,-1.060) |
| Ireland | 0.6 (0.557,0.642) | 14.595 (13.584,15.571) | 0.46 (0.398,0.508) | 5.718 (4.987,6.3) | -3.030 (-3.169,-2.918) | 0.554 (0.515,0.594) | 13.559 (12.56,14.499) | 0.379 (0.324,0.42) | 4.616 (3.971,5.113) | -3.344 (-3.446,-3.259) | 11.607 (10.881,12.357) | 287.63 (270.094,305.347) | 7.315 (6.464,8.05) | 94.437 (84.202,103.387) | -3.441 (-3.570,-3.304) |
| Israel | 0.548 (0.506,0.587) | 11.429 (10.513,12.239) | 0.746 (0.648,0.824) | 5.956 (5.217,6.567) | -2.138 (-2.269,-1.992) | 0.522 (0.48,0.559) | 11.01 (10.105,11.777) | 0.661 (0.569,0.734) | 5.148 (4.474,5.697) | -2.367 (-2.525,-2.211) | 11.116 (10.399,11.878) | 233.01 (217.483,249.306) | 12.932 (11.497,14.24) | 108.361 (96.714,119.059) | -2.455 (-2.601,-2.313) |
| Italy | 20.301 (18.997,21.086) | 22.755 (21.315,23.634) | 15.173 (13.243,16.331) | 10.104 (9.061,10.758) | -2.596 (-2.650,-2.554) | 17.545 (16.249,18.264) | 19.552 (18.109,20.37) | 11.838 (10.194,12.829) | 7.273 (6.483,7.802) | -3.176 (-3.223,-3.127) | 365.126 (345.483,377.25) | 423.559 (402.174,437.168) | 206.915 (185.868,220.59) | 151.819 (140.072,159.992) | -3.305 (-3.366,-3.247) |
| Jamaica | 0.276 (0.254,0.298) | 15.198 (14.005,16.464) | 0.287 (0.221,0.377) | 9.246 (7.114,12.167) | -1.703 (-2.125,-1.290) | 0.282 (0.261,0.306) | 15.46 (14.279,16.775) | 0.286 (0.222,0.371) | 9.123 (7.089,11.868) | -1.802 (-2.206,-1.408) | 6.115 (5.63,6.632) | 345.082 (316.896,375.62) | 6.519 (4.943,8.602) | 211.059 (160.003,278.446) | -1.722 (-2.326,-1.319) |
| Japan | 108.286 (103.064,111.463) | 64.048 (60.738,65.998) | 99.035 (85.266,106.694) | 25.543 (23.036,26.965) | -2.950 (-2.997,-2.924) | 56.091 (52.923,57.893) | 33.758 (31.638,34.944) | 58.012 (48.988,63.019) | 13.197 (11.672,14.041) | -3.010 (-3.074,-2.976) | 1332.552 (1283.575,1367.533) | 790.754 (759.636,811.779) | 925.233 (815.855,984.384) | 270.172 (248.927,282.24) | -3.426 (-3.496,-3.391) |
| Jordan | 0.098 (0.079,0.122) | 7.24 (5.912,9.141) | 0.294 (0.222,0.381) | 4.078 (3.105,5.234) | -1.798 (-1.882,-1.715) | 0.095 (0.077,0.118) | 7.376 (6.033,9.301) | 0.26 (0.199,0.336) | 3.876 (2.971,4.948) | -2.077 (-2.187,-1.984) | 2.885 (2.325,3.594) | 184.668 (149.438,230.015) | 7.201 (5.506,9.422) | 87.574 (67.123,112.934) | -2.329 (-2.412,-2.250) |
| Kazakhstan | 4.359 (4.059,4.685) | 33.816 (31.442,36.503) | 2.07 (1.787,2.374) | 11.291 (9.763,12.906) | -3.411 (-3.549,-3.296) | 4.263 (3.969,4.599) | 33.628 (31.156,36.318) | 1.933 (1.663,2.219) | 10.796 (9.319,12.323) | -3.527 (-3.646,-3.404) | 123.28 (115.266,131.594) | 913.399 (853.684,980.271) | 53.832 (46.542,61.807) | 279.992 (241.984,321.137) | -3.674 (-3.818,-3.544) |
| Kenya | 0.639 (0.48,0.853) | 7.616 (5.745,10.237) | 1.819 (1.436,2.312) | 7.931 (6.304,10.004) | 0.110 (0.068,0.151) | 0.638 (0.48,0.857) | 7.887 (5.937,10.687) | 1.796 (1.416,2.272) | 8.241 (6.524,10.327) | 0.133 (0.093,0.176) | 18.58 (13.837,24.373) | 200.48 (149.625,266.837) | 51.985 (40.727,65.858) | 200.257 (157.77,252.887) | -0.034 (-0.073,0.006) |
| Kiribati | 0.009 (0.007,0.011) | 23.694 (18.309,28.813) | 0.016 (0.011,0.02) | 21.335 (15.529,26.858) | -0.343 (-0.358,-0.329) | 0.009 (0.007,0.011) | 24.79 (18.988,30.118) | 0.015 (0.011,0.019) | 22.06 (16.183,27.519) | -0.381 (-0.396,-0.368) | 0.293 (0.22,0.363) | 666.919 (509.802,816.528) | 0.491 (0.344,0.637) | 575.963 (414.865,736.002) | -0.478 (-0.495,-0.463) |
| Kuwait | 0.026 (0.024,0.03) | 4.313 (3.822,4.89) | 0.085 (0.068,0.106) | 3.04 (2.416,3.815) | -1.269 (-1.701,-0.832) | 0.024 (0.021,0.026) | 4.152 (3.681,4.672) | 0.067 (0.055,0.083) | 2.587 (2.086,3.211) | -1.645 (-2.074,-1.216) | 0.721 (0.652,0.806) | 97.854 (88.021,110.426) | 1.818 (1.486,2.262) | 54.865 (44.292,68.485) | -2.146 (-2.560,-1.746) |
| Kyrgyzstan | 0.962 (0.871,1.056) | 31.605 (28.642,34.646) | 0.792 (0.647,0.944) | 15.794 (12.96,18.853) | -2.367 (-2.632,-2.149) | 0.942 (0.854,1.034) | 31.371 (28.402,34.297) | 0.745 (0.607,0.892) | 15.301 (12.457,18.236) | -2.500 (-2.807,-2.269) | 28.326 (25.574,31.091) | 899.17 (813.209,987.397) | 22.056 (18.014,26.27) | 407.93 (333.513,486.719) | -2.664 (-2.941,-2.452) |
| Lao People's Democratic Republic | 0.363 (0.255,0.48) | 16.872 (11.979,22.187) | 0.389 (0.287,0.505) | 8.398 (6.234,10.872) | -2.255 (-2.274,-2.236) | 0.364 (0.256,0.482) | 17.506 (12.506,23.036) | 0.376 (0.274,0.488) | 8.524 (6.313,10.999) | -2.322 (-2.339,-2.303) | 11.162 (7.821,14.768) | 476.158 (338.483,633.016) | 10.942 (7.96,14.43) | 211.209 (154.307,275.638) | -2.614 (-2.639,-2.588) |
| Latvia | 0.947 (0.87,1.027) | 26.576 (24.407,28.825) | 0.481 (0.41,0.551) | 12.64 (10.68,14.568) | -2.383 (-2.575,-2.179) | 0.867 (0.8,0.942) | 24.223 (22.29,26.298) | 0.425 (0.361,0.488) | 10.735 (9.038,12.386) | -2.739 (-2.916,-2.575) | 22.451 (20.585,24.462) | 640.467 (587.08,698.322) | 9.352 (7.859,10.87) | 267.493 (223.74,312.439) | -3.031 (-3.239,-2.839) |
| Lebanon | 0.221 (0.178,0.281) | 10.469 (8.43,13.168) | 0.354 (0.285,0.431) | 5.755 (4.631,7.017) | -1.873 (-1.936,-1.821) | 0.219 (0.177,0.278) | 10.7 (8.665,13.467) | 0.338 (0.271,0.408) | 5.392 (4.317,6.503) | -2.156 (-2.217,-2.104) | 5.883 (4.741,7.522) | 259.811 (209.62,330.753) | 7.133 (5.764,8.65) | 118.729 (95.931,143.966) | -2.461 (-2.518,-2.411) |
| Lesotho | 0.075 (0.053,0.096) | 8.978 (6.332,11.494) | 0.132 (0.091,0.176) | 12.079 (8.458,15.792) | 1.035 (0.939,1.114) | 0.077 (0.054,0.098) | 9.448 (6.687,12.105) | 0.132 (0.093,0.176) | 12.514 (8.927,16.328) | 1.000 (0.881,1.094) | 2.004 (1.406,2.597) | 227.964 (160.158,294.772) | 3.841 (2.613,5.221) | 323.792 (223.586,435.007) | 1.172 (1.051,1.276) |
| Liberia | 0.12 (0.091,0.148) | 10.67 (8.174,13.004) | 0.2 (0.13,0.268) | 9.689 (6.455,12.839) | -0.291 (-0.384,-0.223) | 0.124 (0.095,0.153) | 11.394 (8.734,13.911) | 0.198 (0.13,0.268) | 10.191 (6.866,13.513) | -0.342 (-0.424,-0.280) | 3.274 (2.465,4.104) | 270.609 (205.399,335.405) | 5.743 (3.678,7.876) | 237.248 (154.916,320.291) | -0.410 (-0.493,-0.341) |
| Libya | 0.147 (0.111,0.188) | 7.812 (5.846,9.985) | 0.357 (0.261,0.472) | 6.774 (5.024,8.822) | -0.401 (-0.525,-0.302) | 0.146 (0.111,0.188) | 7.961 (5.903,10.274) | 0.333 (0.248,0.439) | 6.62 (4.947,8.671) | -0.535 (-0.669,-0.423) | 4.026 (3.104,5.209) | 195.396 (148.94,250.763) | 9.497 (6.904,12.74) | 160.128 (118.571,213.096) | -0.564 (-0.649,-0.471) |
| Lithuania | 1.211 (1.12,1.298) | 26.98 (24.906,28.949) | 0.704 (0.591,0.823) | 12.665 (10.632,14.825) | -2.543 (-2.721,-2.402) | 1.092 (1.01,1.169) | 24.244 (22.418,25.937) | 0.611 (0.515,0.713) | 10.625 (8.899,12.469) | -2.781 (-2.997,-2.652) | 28.167 (25.993,30.411) | 634.168 (584.582,684.589) | 13.427 (11.236,15.793) | 265.682 (220.288,311.911) | -2.941 (-3.132,-2.821) |
| Luxembourg | 0.078 (0.073,0.083) | 14.319 (13.453,15.188) | 0.058 (0.051,0.065) | 5.29 (4.667,5.87) | -3.098 (-3.193,-3.017) | 0.073 (0.068,0.077) | 13.323 (12.507,14.14) | 0.05 (0.044,0.056) | 4.456 (3.907,4.937) | -3.422 (-3.588,-3.274) | 1.563 (1.47,1.652) | 294.728 (278.065,311.643) | 0.932 (0.829,1.029) | 88.33 (78.692,97.579) | -3.806 (-3.899,-3.725) |
| Madagascar | 0.445 (0.33,0.533) | 8.587 (6.359,10.232) | 0.724 (0.496,0.959) | 6.2 (4.315,8.244) | -1.033 (-1.075,-0.982) | 0.446 (0.332,0.534) | 8.951 (6.629,10.689) | 0.706 (0.48,0.937) | 6.46 (4.485,8.548) | -1.032 (-1.073,-0.983) | 13.355 (10.005,16.054) | 232.961 (173.232,278.473) | 22.719 (15.419,30.289) | 164.152 (112.269,217.472) | -1.103 (-1.149,-1.046) |
| Malawi | 0.15 (0.116,0.177) | 3.849 (2.979,4.525) | 0.238 (0.174,0.3) | 3.125 (2.335,3.891) | -0.638 (-0.675,-0.596) | 0.15 (0.117,0.177) | 4.042 (3.178,4.755) | 0.235 (0.174,0.296) | 3.241 (2.449,4.034) | -0.674 (-0.707,-0.636) | 4.511 (3.52,5.33) | 103.066 (80.538,121.694) | 7.088 (5.195,9.088) | 81.802 (60.318,103.393) | -0.712 (-0.756,-0.665) |
| Malaysia | 0.807 (0.678,0.955) | 8.736 (7.28,10.256) | 1.892 (1.614,2.304) | 6.794 (5.783,8.155) | -0.986 (-1.134,-0.837) | 0.798 (0.667,0.934) | 8.889 (7.393,10.399) | 1.703 (1.458,2.054) | 6.343 (5.413,7.517) | -1.190 (-1.487,-0.915) | 21.958 (18.626,26.192) | 218.951 (184.756,259.33) | 43.838 (37.604,53.864) | 148.904 (127.831,181.122) | -1.224 (-1.397,-1.011) |
| Maldives | 0.008 (0.006,0.011) | 9.261 (7.134,11.71) | 0.01 (0.008,0.013) | 3.029 (2.34,3.855) | -3.662 (-3.756,-3.545) | 0.008 (0.006,0.01) | 9.512 (7.278,12.011) | 0.009 (0.007,0.011) | 2.801 (2.191,3.598) | -3.986 (-4.081,-3.872) | 0.255 (0.189,0.319) | 245.779 (189.118,306.462) | 0.239 (0.182,0.305) | 61.293 (47.06,78.026) | -4.499 (-4.598,-4.383) |
| Mali | 0.86 (0.7,1.021) | 21.595 (17.952,25.692) | 1.442 (1.089,1.843) | 16.327 (12.601,20.753) | -0.874 (-0.906,-0.839) | 0.863 (0.703,1.026) | 22.642 (18.813,27.064) | 1.437 (1.091,1.849) | 17.077 (13.4,21.833) | -0.863 (-0.902,-0.831) | 25.555 (20.386,30.553) | 578.525 (470.701,688.578) | 41.969 (31.351,54.627) | 420.318 (318.93,542.708) | -1.013 (-1.045,-0.979) |
| Malta | 0.061 (0.056,0.067) | 14.416 (13.193,15.726) | 0.056 (0.048,0.063) | 5.623 (4.88,6.315) | -3.129 (-3.320,-2.790) | 0.057 (0.052,0.062) | 13.476 (12.295,14.644) | 0.049 (0.041,0.055) | 4.69 (4.016,5.275) | -3.325 (-3.549,-3.076) | 1.232 (1.131,1.34) | 287.924 (264.465,313.269) | 0.917 (0.798,1.03) | 99.583 (87.13,111.739) | -3.523 (-3.682,-3.214) |
| Marshall Islands | 0.004 (0.003,0.005) | 22.685 (18.052,28.397) | 0.006 (0.005,0.008) | 17.73 (13.412,22.425) | -0.791 (-0.842,-0.724) | 0.004 (0.003,0.005) | 23.552 (18.714,29.582) | 0.006 (0.004,0.008) | 17.928 (13.718,22.59) | -0.875 (-0.928,-0.800) | 0.122 (0.096,0.154) | 617.872 (488.377,779.592) | 0.199 (0.145,0.257) | 470.292 (349.215,602.712) | -0.872 (-0.915,-0.816) |
| Mauritania | 0.117 (0.092,0.141) | 11.958 (9.548,14.381) | 0.179 (0.129,0.237) | 8.698 (6.219,11.414) | -1.031 (-1.103,-0.977) | 0.12 (0.096,0.144) | 12.658 (10.126,15.162) | 0.181 (0.129,0.239) | 9.106 (6.463,11.92) | -1.068 (-1.135,-1.016) | 3.166 (2.507,3.841) | 303.625 (240.207,366.898) | 4.516 (3.244,6.044) | 201.821 (144.279,268.896) | -1.325 (-1.405,-1.266) |
| Mauritius | 0.116 (0.109,0.123) | 15.936 (15.002,16.911) | 0.199 (0.181,0.215) | 11.022 (10.025,11.824) | -1.073 (-1.263,-0.891) | 0.112 (0.105,0.118) | 15.927 (14.969,16.879) | 0.179 (0.163,0.192) | 9.973 (9.124,10.687) | -1.397 (-1.586,-1.211) | 3.11 (2.921,3.306) | 394.273 (370.933,418.321) | 4.568 (4.124,4.939) | 252.404 (229.091,272.236) | -1.454 (-1.654,-1.245) |
| Mexico | 6.26 (6.069,6.417) | 15.536 (14.978,15.934) | 11.776 (10.431,13.168) | 9.376 (8.303,10.465) | -1.619 (-1.689,-1.542) | 6.411 (6.212,6.574) | 16.631 (16.006,17.066) | 11.389 (10.096,12.705) | 9.238 (8.2,10.293) | -1.880 (-1.953,-1.801) | 161.26 (157.525,165.115) | 359.713 (350.315,368.507) | 291.544 (256.836,326.244) | 223.468 (197.16,249.731) | -1.536 (-1.610,-1.448) |
| Micronesia  (Federated States of) | 0.011 (0.009,0.015) | 22.539 (17.794,29.331) | 0.012 (0.009,0.017) | 17.127 (13.159,22.237) | -0.889 (-0.902,-0.874) | 0.011 (0.009,0.015) | 23.446 (18.401,30.476) | 0.012 (0.009,0.016) | 17.172 (13.245,22.067) | -1.007 (-1.018,-0.993) | 0.34 (0.263,0.44) | 617.197 (483.085,808.027) | 0.367 (0.269,0.493) | 444.306 (332.543,589.038) | -1.059 (-1.068,-1.050) |
| Monaco | 0.01 (0.007,0.012) | 13.96 (10.309,17.334) | 0.009 (0.007,0.011) | 8.733 (6.891,11.425) | -1.495 (-1.520,-1.471) | 0.009 (0.006,0.011) | 12.052 (8.784,14.868) | 0.007 (0.006,0.01) | 7.105 (5.613,9.25) | -1.683 (-1.702,-1.663) | 0.17 (0.125,0.21) | 265.082 (196.73,329.384) | 0.134 (0.106,0.177) | 153.109 (117.844,202.748) | -1.770 (-1.796,-1.748) |
| Mongolia | 0.574 (0.466,0.715) | 54.22 (44.046,67.942) | 0.848 (0.68,1.062) | 36.827 (29.387,45.273) | -1.263 (-1.397,-1.141) | 0.585 (0.476,0.734) | 56.425 (45.753,71.226) | 0.825 (0.659,1.028) | 37.4 (29.358,45.86) | -1.352 (-1.497,-1.217) | 16.45 (13.255,20.644) | 1462.929 (1184.845,1839.723) | 24.064 (19.036,30.532) | 930.449 (747.523,1157.922) | -1.478 (-1.591,-1.367) |
| Montenegro | 0.057 (0.048,0.069) | 9.184 (7.765,11.088) | 0.081 (0.067,0.099) | 8.373 (6.941,10.179) | -0.214 (-0.348,-0.074) | 0.053 (0.045,0.065) | 8.677 (7.339,10.492) | 0.075 (0.062,0.092) | 7.897 (6.504,9.643) | -0.096 (-0.188,-0.001) | 1.351 (1.14,1.63) | 211.734 (178.705,255.21) | 1.632 (1.342,2.023) | 170.07 (140.51,209.856) | -0.597 (-0.713,-0.492) |
| Morocco | 0.445 (0.327,0.533) | 3.115 (2.283,3.731) | 0.85 (0.585,1.051) | 2.496 (1.737,3.066) | -0.701 (-0.723,-0.682) | 0.452 (0.333,0.545) | 3.234 (2.378,3.885) | 0.834 (0.583,1.03) | 2.511 (1.781,3.078) | -0.806 (-0.836,-0.775) | 12.445 (9.258,15.145) | 81.74 (60.138,99.177) | 21.561 (14.868,27.184) | 60.061 (41.747,75.105) | -0.978 (-1.001,-0.958) |
| Mozambique | 0.43 (0.317,0.514) | 7.852 (5.693,9.333) | 0.779 (0.54,0.996) | 7.557 (5.187,9.538) | -0.095 (-0.121,-0.071) | 0.445 (0.326,0.53) | 8.517 (6.154,10.069) | 0.792 (0.554,1.016) | 8.12 (5.665,10.292) | -0.131 (-0.170,-0.098) | 11.794 (8.991,14.232) | 191.799 (141.142,228.702) | 21.969 (15.604,28.822) | 185.06 (129.526,238.519) | -0.099 (-0.140,-0.063) |
| Myanmar | 3.635 (2.646,4.689) | 15.157 (11.13,19.38) | 3.568 (2.75,4.705) | 7.396 (5.745,9.742) | -2.296 (-2.314,-2.276) | 3.62 (2.639,4.648) | 15.644 (11.541,19.89) | 3.443 (2.668,4.54) | 7.38 (5.789,9.751) | -2.416 (-2.439,-2.390) | 111.607 (79.713,145.417) | 425.719 (309.959,549.353) | 94.025 (70.953,124.816) | 182.544 (139.064,242.118) | -2.699 (-2.722,-2.676) |
| Namibia | 0.022 (0.017,0.027) | 3.357 (2.68,4.1) | 0.038 (0.028,0.049) | 2.704 (2.05,3.419) | -0.638 (-0.689,-0.591) | 0.022 (0.017,0.027) | 3.472 (2.785,4.25) | 0.037 (0.027,0.047) | 2.741 (2.088,3.451) | -0.702 (-0.753,-0.655) | 0.641 (0.498,0.793) | 89.265 (69.792,110.225) | 1.076 (0.77,1.423) | 69.41 (50.422,89.76) | -0.750 (-0.806,-0.700) |
| Nauru | 0.001 (0.001,0.002) | 26.312 (20.276,34.591) | 0.001 (0.001,0.002) | 21.092 (15.773,26.58) | -0.720 (-0.737,-0.702) | 0.001 (0.001,0.002) | 27.024 (21.028,35.627) | 0.001 (0.001,0.002) | 20.853 (15.467,26.179) | -0.839 (-0.871,-0.802) | 0.041 (0.031,0.054) | 720.11 (545.583,955.602) | 0.039 (0.028,0.051) | 557.027 (409.518,709.528) | -0.834 (-0.859,-0.810) |
| Nepal | 0.838 (0.585,1.093) | 8.57 (6.126,10.983) | 1.408 (1.027,1.869) | 6.11 (4.466,8.063) | -1.069 (-1.102,-1.037) | 0.831 (0.583,1.072) | 8.848 (6.359,11.235) | 1.393 (1.027,1.84) | 6.243 (4.565,8.227) | -1.101 (-1.129,-1.073) | 25.85 (17.926,33.538) | 238.456 (166.721,308.059) | 37.672 (28.088,50.005) | 153.42 (113.741,202.796) | -1.401 (-1.431,-1.370) |
| Netherlands | 2.399 (2.218,2.537) | 11.835 (10.971,12.521) | 1.503 (1.317,1.66) | 4.14 (3.658,4.55) | -3.410 (-3.492,-3.341) | 2.63 (2.423,2.789) | 12.886 (11.899,13.641) | 1.717 (1.483,1.903) | 4.623 (4.049,5.105) | -3.326 (-3.405,-3.258) | 54.197 (50.69,57.353) | 276.269 (258.9,292.157) | 31.874 (28.473,34.827) | 94.737 (85.331,102.93) | -3.482 (-3.568,-3.407) |
| New Zealand | 0.475 (0.443,0.506) | 12.136 (11.287,12.899) | 0.579 (0.518,0.638) | 6.926 (6.214,7.611) | -1.665 (-1.878,-1.478) | 0.372 (0.346,0.396) | 9.533 (8.849,10.135) | 0.395 (0.352,0.434) | 4.586 (4.098,5.035) | -2.203 (-2.430,-2.005) | 8.388 (7.866,8.933) | 217.628 (204.34,231.717) | 8.116 (7.345,8.85) | 103.2 (93.897,112.589) | -2.204 (-2.452,-1.991) |
| Nicaragua | 0.207 (0.182,0.246) | 13.427 (11.76,15.973) | 0.482 (0.389,0.606) | 9.891 (7.992,12.447) | -0.996 (-1.183,-0.831) | 0.207 (0.182,0.245) | 13.929 (12.191,16.6) | 0.463 (0.374,0.582) | 9.776 (7.91,12.274) | -1.154 (-1.334,-0.993) | 5.75 (5.052,6.814) | 336.071 (295.609,399.448) | 12.197 (9.768,15.278) | 235.404 (189.301,294.174) | -1.213 (-1.392,-1.055) |
| Niger | 0.341 (0.253,0.424) | 12.619 (9.519,15.591) | 0.835 (0.573,1.107) | 10.778 (7.569,13.926) | -0.484 (-0.519,-0.455) | 0.345 (0.258,0.428) | 13.407 (10.15,16.435) | 0.85 (0.584,1.118) | 11.562 (8.162,14.839) | -0.463 (-0.495,-0.432) | 10.1 (7.587,12.615) | 326.682 (244.859,405.594) | 23.371 (15.897,31.135) | 264.309 (182.153,348.453) | -0.661 (-0.704,-0.625) |
| Nigeria | 1.513 (1.163,2.208) | 3.457 (2.66,5.135) | 2.188 (1.733,2.812) | 2.477 (2.002,3.204) | -1.075 (-1.095,-1.053) | 1.552 (1.193,2.265) | 3.661 (2.82,5.452) | 2.206 (1.785,2.821) | 2.627 (2.137,3.403) | -1.081 (-1.108,-1.046) | 42.668 (32.505,60.724) | 89.895 (68.812,129.734) | 61.381 (48.101,79.827) | 60.621 (48.518,77.952) | -1.266 (-1.297,-1.236) |
| Niue | 0 (0,0) | 14.52 (11.666,18.209) | 0 (0,0) | 11.992 (9.595,14.813) | -0.600 (-0.623,-0.579) | 0 (0,0) | 14.909 (12.015,18.56) | 0 (0,0) | 11.624 (9.291,14.227) | -0.787 (-0.814,-0.764) | 0.008 (0.006,0.01) | 367.446 (287.476,467.261) | 0.006 (0.005,0.007) | 279.534 (222.842,350.529) | -0.858 (-0.891,-0.828) |
| North Macedonia | 0.398 (0.336,0.464) | 21.68 (18.416,25.456) | 0.458 (0.359,0.576) | 14.274 (11.3,17.715) | -1.299 (-1.386,-1.207) | 0.396 (0.337,0.463) | 22 (18.695,25.905) | 0.437 (0.343,0.548) | 14.068 (11.166,17.443) | -1.413 (-1.498,-1.321) | 10.153 (8.48,11.744) | 527.37 (443.221,611.711) | 9.984 (7.761,12.594) | 301.736 (236.779,377.39) | -1.712 (-1.843,-1.607) |
| Northern Mariana Islands | 0.003 (0.002,0.004) | 17.544 (13.959,22.238) | 0.007 (0.006,0.008) | 14.401 (12.009,17.078) | -0.699 (-0.824,-0.580) | 0.003 (0.002,0.004) | 17.605 (14.043,22.122) | 0.006 (0.005,0.007) | 13.654 (11.45,16.074) | -0.907 (-1.105,-0.680) | 0.102 (0.075,0.137) | 417.018 (324.052,539.879) | 0.166 (0.139,0.201) | 314.416 (264.208,373.862) | -0.906 (-0.962,-0.862) |
| Norway | 0.92 (0.86,0.961) | 13.216 (12.444,13.773) | 0.552 (0.491,0.59) | 5.35 (4.824,5.697) | -2.884 (-2.953,-2.822) | 0.787 (0.73,0.821) | 11.01 (10.297,11.466) | 0.409 (0.362,0.437) | 3.801 (3.422,4.049) | -3.389 (-3.441,-3.325) | 15.609 (14.791,16.182) | 241.152 (230.467,249.867) | 7.483 (6.868,7.919) | 77.012 (71.405,81.123) | -3.635 (-3.760,-3.547) |
| Oman | 0.077 (0.056,0.103) | 11.19 (8.191,14.811) | 0.109 (0.083,0.137) | 5.75 (4.368,7.151) | -2.096 (-2.162,-2.012) | 0.074 (0.054,0.098) | 11.158 (8.248,14.729) | 0.092 (0.071,0.115) | 5.331 (4.11,6.545) | -2.385 (-2.477,-2.300) | 2.162 (1.571,2.886) | 277.011 (202.647,367.814) | 2.642 (2.027,3.334) | 115.737 (88.843,143.866) | -2.793 (-2.852,-2.743) |
| Pakistan | 2.957 (2.393,3.718) | 5.244 (4.252,6.615) | 5.827 (4.557,7.473) | 4.688 (3.71,5.953) | -0.340 (-0.366,-0.311) | 3.007 (2.443,3.787) | 5.494 (4.446,6.91) | 5.738 (4.491,7.323) | 4.852 (3.831,6.105) | -0.374 (-0.398,-0.347) | 84.312 (68.796,105.989) | 138.645 (112.844,175.574) | 172.298 (133.304,220.918) | 122.02 (95.35,156.35) | -0.389 (-0.415,-0.361) |
| Palau | 0.002 (0.002,0.003) | 24.973 (19.895,31.628) | 0.004 (0.003,0.005) | 19.652 (15.879,23.988) | -0.813 (-0.856,-0.768) | 0.002 (0.002,0.003) | 25.522 (20.32,32.058) | 0.004 (0.003,0.005) | 18.946 (15.352,23.278) | -1.002 (-1.042,-0.961) | 0.069 (0.054,0.09) | 634.285 (496.014,821.393) | 0.107 (0.083,0.134) | 472.608 (374.973,583.381) | -0.956 (-0.990,-0.928) |
| Palestine | 0.095 (0.073,0.122) | 11.472 (8.909,14.53) | 0.151 (0.119,0.18) | 6.39 (5.135,7.619) | -1.925 (-2.023,-1.843) | 0.097 (0.075,0.123) | 12.056 (9.464,15.195) | 0.141 (0.114,0.168) | 6.378 (5.127,7.557) | -2.090 (-2.161,-2.017) | 2.451 (1.88,3.155) | 272.361 (209.917,347.753) | 3.743 (2.985,4.486) | 139.102 (112.18,165.566) | -2.195 (-2.286,-2.115) |
| Panama | 0.206 (0.19,0.222) | 13.981 (12.827,15.069) | 0.464 (0.366,0.56) | 10.466 (8.244,12.637) | -0.643 (-0.823,-0.430) | 0.206 (0.189,0.223) | 14.294 (13.078,15.455) | 0.446 (0.349,0.54) | 10.03 (7.833,12.142) | -1.053 (-1.409,-0.814) | 5.095 (4.741,5.469) | 327.131 (303.682,351.51) | 10.368 (8.207,12.576) | 234.645 (185.623,284.672) | -1.002 (-1.228,-0.811) |
| Papua New Guinea | 0.33 (0.218,0.448) | 17.945 (11.97,24.216) | 0.707 (0.511,0.928) | 13.825 (10.112,17.948) | -0.825 (-0.886,-0.775) | 0.319 (0.213,0.435) | 18.659 (12.531,24.9) | 0.673 (0.486,0.886) | 14.187 (10.434,18.448) | -0.874 (-0.925,-0.831) | 10.384 (6.748,14.147) | 481.417 (322.46,655.069) | 21.621 (15.514,28.762) | 354.175 (255.786,465.585) | -1.001 (-1.056,-0.944) |
| Paraguay | 0.221 (0.185,0.269) | 10.09 (8.461,12.305) | 0.501 (0.372,0.668) | 8.734 (6.497,11.604) | -0.353 (-0.459,-0.239) | 0.224 (0.188,0.271) | 10.428 (8.776,12.699) | 0.492 (0.365,0.655) | 8.726 (6.504,11.574) | -0.437 (-0.543,-0.321) | 5.646 (4.745,6.812) | 243.27 (204.22,293.899) | 12.047 (8.883,16.136) | 201.166 (148.652,268.785) | -0.508 (-0.632,-0.385) |
| Peru | 3.255 (2.698,3.847) | 27.374 (22.748,32.3) | 6.906 (5.131,8.858) | 20.619 (15.356,26.471) | -0.544 (-0.953,-0.166) | 3.329 (2.756,3.927) | 28.671 (23.752,33.905) | 6.648 (4.976,8.597) | 19.949 (14.954,25.835) | -0.799 (-1.197,-0.406) | 87.09 (71.251,102.454) | 680.159 (560.256,802.192) | 155.593 (115.158,200.902) | 455.295 (336.568,586.035) | -0.935 (-1.374,-0.539) |
| Philippines | 1.663 (1.428,1.99) | 5.447 (4.702,6.632) | 3.734 (3.086,4.991) | 4.481 (3.717,5.94) | -0.561 (-0.615,-0.510) | 1.601 (1.374,1.924) | 5.617 (4.862,6.856) | 3.498 (2.894,4.61) | 4.379 (3.635,5.711) | -0.775 (-0.839,-0.678) | 50.711 (43.357,59.383) | 143.852 (123.309,172.169) | 101.402 (83.389,133.875) | 111.998 (92.801,147.561) | -0.744 (-0.796,-0.697) |
| Poland | 8.321 (8.006,8.584) | 19.096 (18.352,19.695) | 5.735 (5.202,6.218) | 8.05 (7.318,8.707) | -2.905 (-3.023,-2.823) | 8.93 (8.587,9.218) | 20.621 (19.789,21.287) | 6.111 (5.523,6.636) | 8.421 (7.635,9.125) | -3.023 (-3.265,-2.878) | 215.508 (208.708,221.741) | 493.404 (477.854,507.539) | 132.787 (120.904,143.601) | 196.831 (179.693,213) | -3.080 (-3.240,-3.000) |
| Portugal | 4.161 (3.878,4.413) | 30.75 (28.652,32.591) | 3.06 (2.745,3.313) | 12.902 (11.743,13.891) | -2.790 (-2.882,-2.710) | 4.072 (3.806,4.321) | 30.295 (28.148,32.115) | 2.784 (2.457,3.023) | 10.751 (9.714,11.595) | -3.259 (-3.352,-3.167) | 90.775 (85.2,96.571) | 678.867 (637.28,722.34) | 53.466 (48.542,57.575) | 242.986 (222.976,260.564) | -3.302 (-3.399,-3.216) |
| Puerto Rico | 0.436 (0.406,0.466) | 12.096 (11.254,12.928) | 0.337 (0.28,0.4) | 4.777 (3.998,5.68) | -3.144 (-3.645,-2.902) | 0.43 (0.4,0.46) | 12.042 (11.185,12.901) | 0.317 (0.266,0.378) | 4.241 (3.569,5.042) | -3.300 (-3.515,-3.069) | 9.546 (8.896,10.199) | 263.881 (245.698,281.789) | 6.242 (5.24,7.423) | 98.995 (82.583,118.102) | -3.232 (-3.390,-3.008) |
| Qatar | 0.015 (0.012,0.019) | 15.751 (12.32,19.524) | 0.054 (0.04,0.074) | 6.463 (4.859,8.444) | -2.842 (-3.117,-2.567) | 0.014 (0.011,0.017) | 16.13 (12.653,19.807) | 0.04 (0.03,0.054) | 5.817 (4.561,7.544) | -3.254 (-3.533,-2.973) | 0.431 (0.334,0.546) | 338.916 (264.456,428.31) | 1.286 (0.955,1.781) | 115.027 (87.776,151.838) | -3.442 (-3.663,-3.217) |
| Republic of Korea | 22.167 (16.777,25.107) | 71.183 (55.991,80.705) | 23.664 (19.71,29.807) | 25.762 (21.525,32.399) | -3.229 (-3.332,-3.155) | 16.261 (12.515,18.306) | 55.443 (44.066,62.448) | 12.27 (10.052,15.312) | 13.257 (10.906,16.485) | -4.525 (-4.602,-4.469) | 489.123 (356.246,549.576) | 1435.386 (1091.491,1613.828) | 260.065 (218.481,330.431) | 288.822 (242.926,366.071) | -5.059 (-5.127,-5.010) |
| Republic of Moldova | 0.956 (0.879,1.037) | 21.35 (19.62,23.16) | 0.575 (0.497,0.671) | 9.68 (8.382,11.266) | -2.599 (-2.802,-2.349) | 0.891 (0.818,0.97) | 20.238 (18.56,22.032) | 0.5 (0.427,0.584) | 8.377 (7.156,9.772) | -2.890 (-3.086,-2.649) | 25.151 (23.226,27.078) | 547.212 (504.997,590.694) | 12.969 (11.05,15.155) | 222.463 (189.748,259.619) | -2.956 (-3.179,-2.699) |
| Romania | 4.257 (3.881,4.625) | 15.173 (13.847,16.454) | 4.217 (3.655,4.824) | 11.57 (10.065,13.239) | -0.851 (-1.039,-0.703) | 4.155 (3.786,4.515) | 14.991 (13.66,16.244) | 3.855 (3.378,4.385) | 10.367 (9.083,11.814) | -1.187 (-1.356,-1.037) | 110.927 (101.501,119.823) | 391.972 (359.316,423.306) | 87.593 (76.635,99.987) | 256.885 (223.347,293.235) | -1.356 (-1.535,-1.199) |
| Russian Federation | 62.96 (61.419,64.15) | 34.535 (33.646,35.197) | 37.222 (34.092,40.262) | 15.693 (14.381,16.958) | -2.563 (-2.662,-2.449) | 57.112 (55.638,58.221) | 31.523 (30.645,32.142) | 31.124 (28.571,33.685) | 12.999 (11.945,14.05) | -2.915 (-3.125,-2.758) | 1551.46 (1516.41,1580.546) | 847.352 (827.389,862.974) | 738.009 (675.161,799.696) | 318.759 (291.778,345.001) | -3.236 (-3.408,-3.053) |
| Rwanda | 0.384 (0.277,0.471) | 13.066 (9.563,15.927) | 0.414 (0.302,0.549) | 6.603 (4.896,8.719) | -2.178 (-2.221,-2.136) | 0.384 (0.28,0.471) | 13.624 (10.023,16.512) | 0.41 (0.3,0.548) | 6.911 (5.162,9.179) | -2.166 (-2.207,-2.123) | 11.967 (8.707,14.805) | 364.626 (265.448,447.999) | 11.957 (8.681,16.102) | 167.117 (122.391,222.842) | -2.499 (-2.548,-2.449) |
| Saint Kitts and Nevis | 0.006 (0.006,0.007) | 16.361 (15.096,17.569) | 0.006 (0.005,0.007) | 8.981 (7.471,10.528) | -1.850 (-1.966,-1.724) | 0.006 (0.006,0.007) | 17.191 (15.88,18.38) | 0.006 (0.005,0.007) | 9.146 (7.63,10.643) | -1.946 (-2.077,-1.804) | 0.146 (0.134,0.156) | 405.126 (373.203,435.475) | 0.141 (0.112,0.17) | 199.053 (162.054,236.272) | -2.155 (-2.290,-2.008) |
| Saint Lucia | 0.018 (0.016,0.019) | 20.993 (19.551,22.518) | 0.026 (0.021,0.031) | 10.785 (8.941,13.067) | -2.236 (-2.349,-2.132) | 0.018 (0.017,0.019) | 22.078 (20.587,23.659) | 0.026 (0.021,0.031) | 10.913 (9.095,13.173) | -2.304 (-2.408,-2.215) | 0.439 (0.408,0.473) | 501.716 (467.112,540.413) | 0.59 (0.482,0.723) | 247.12 (202.123,302.372) | -2.303 (-2.412,-2.212) |
| Saint Vincent and the Grenadines | 0.012 (0.011,0.013) | 17.317 (15.777,18.992) | 0.014 (0.013,0.016) | 10.217 (8.997,11.77) | -1.787 (-1.904,-1.684) | 0.012 (0.011,0.014) | 17.98 (16.367,19.709) | 0.014 (0.013,0.017) | 10.435 (9.212,11.986) | -1.846 (-1.970,-1.735) | 0.301 (0.275,0.333) | 420.416 (383.853,463.562) | 0.343 (0.3,0.398) | 243.227 (212.792,281.425) | -1.852 (-1.981,-1.743) |
| Samoa | 0.011 (0.007,0.013) | 12.806 (8.996,15.392) | 0.016 (0.011,0.02) | 10.827 (7.594,13.603) | -0.542 (-0.557,-0.529) | 0.011 (0.007,0.013) | 13.204 (9.372,15.797) | 0.015 (0.01,0.018) | 10.56 (7.465,13.311) | -0.718 (-0.734,-0.704) | 0.292 (0.208,0.358) | 324.398 (229.08,395.771) | 0.402 (0.281,0.519) | 263.55 (183.958,337.254) | -0.667 (-0.685,-0.651) |
| San Marino | 0.011 (0.009,0.014) | 31.081 (25.006,37.74) | 0.009 (0.006,0.013) | 11.691 (7.726,16.506) | -3.362 (-3.779,-3.088) | 0.01 (0.008,0.012) | 27.019 (21.529,32.644) | 0.008 (0.005,0.011) | 9.529 (6.376,13.269) | -3.577 (-4.005,-3.275) | 0.191 (0.153,0.231) | 554.602 (445.387,669.033) | 0.14 (0.093,0.197) | 198.305 (127.513,283.964) | -3.501 (-3.911,-3.241) |
| Sao Tome and Principe | 0.01 (0.008,0.012) | 15.999 (12.97,18.773) | 0.015 (0.012,0.019) | 14.639 (11.564,18.153) | -0.254 (-0.290,-0.221) | 0.01 (0.008,0.012) | 17.2 (13.833,20.072) | 0.015 (0.012,0.019) | 15.438 (12.118,18.86) | -0.352 (-0.395,-0.313) | 0.247 (0.201,0.29) | 380.021 (309.399,445.787) | 0.38 (0.304,0.488) | 331.547 (264.496,420.047) | -0.436 (-0.475,-0.406) |
| Saudi Arabia | 0.364 (0.258,0.552) | 6.272 (4.525,9.244) | 0.796 (0.601,1.268) | 3.893 (3.092,5.831) | -1.539 (-1.579,-1.490) | 0.359 (0.258,0.537) | 6.496 (4.78,9.487) | 0.663 (0.505,1.039) | 3.606 (2.886,5.29) | -1.910 (-1.952,-1.862) | 10.336 (7.281,15.733) | 155 (110.493,232.595) | 22.106 (16.294,35.375) | 85.578 (66.695,131.186) | -1.921 (-1.951,-1.890) |
| Senegal | 0.379 (0.286,0.456) | 11.943 (9.098,14.333) | 0.759 (0.546,0.985) | 10.2 (7.335,13.038) | -0.491 (-0.603,-0.378) | 0.386 (0.299,0.465) | 12.608 (9.846,15.136) | 0.773 (0.553,0.997) | 10.795 (7.744,13.736) | -0.491 (-0.611,-0.395) | 10.556 (8.018,12.764) | 305.523 (234.376,368.994) | 20.241 (14.508,26.577) | 246.876 (176.323,321.283) | -0.656 (-0.798,-0.520) |
| Serbia | 1.426 (1.135,1.708) | 13.504 (11.044,16.189) | 1.355 (1.053,1.732) | 8.237 (6.374,10.461) | -1.522 (-1.647,-1.376) | 1.417 (1.147,1.71) | 13.913 (11.453,16.675) | 1.295 (1.006,1.666) | 7.718 (5.986,9.864) | -1.815 (-1.939,-1.673) | 36.373 (28.232,43.656) | 324.529 (258.381,387.776) | 28.349 (21.64,36.238) | 179.909 (137.296,228.553) | -1.821 (-1.950,-1.676) |
| Seychelles | 0.006 (0.005,0.008) | 10.77 (9.186,13.331) | 0.007 (0.006,0.009) | 6.474 (5.361,8.123) | -1.699 (-1.876,-1.557) | 0.006 (0.005,0.007) | 10.726 (9.158,13.163) | 0.007 (0.006,0.009) | 6.031 (5.021,7.546) | -1.885 (-2.038,-1.742) | 0.16 (0.137,0.198) | 282.891 (241.021,350.493) | 0.181 (0.149,0.227) | 148.49 (122.819,186.277) | -2.078 (-2.178,-1.995) |
| Sierra Leone | 0.221 (0.163,0.268) | 10.998 (8.195,13.336) | 0.365 (0.242,0.472) | 9.888 (6.636,12.691) | -0.344 (-0.375,-0.316) | 0.229 (0.17,0.278) | 11.702 (8.764,14.172) | 0.37 (0.25,0.482) | 10.45 (7.138,13.4) | -0.366 (-0.397,-0.336) | 5.93 (4.292,7.362) | 278.051 (202.394,343.517) | 10.143 (6.741,13.543) | 245.254 (164.513,322.913) | -0.410 (-0.442,-0.381) |
| Singapore | 0.51 (0.477,0.54) | 23.101 (21.626,24.492) | 0.708 (0.634,0.774) | 8.473 (7.579,9.255) | -3.114 (-3.224,-3.022) | 0.348 (0.329,0.368) | 16.548 (15.609,17.583) | 0.375 (0.336,0.41) | 4.534 (4.061,4.948) | -3.894 (-4.221,-3.655) | 9.205 (8.675,9.737) | 388.468 (367.272,410.205) | 7.795 (7.086,8.467) | 92.39 (83.997,100.473) | -4.465 (-4.581,-4.365) |
| Slovakia | 1.235 (1.011,1.459) | 20.538 (16.86,24.271) | 1 (0.803,1.231) | 10.461 (8.385,12.849) | -2.164 (-2.283,-2.046) | 1.078 (0.887,1.267) | 17.979 (14.774,21.116) | 0.796 (0.64,0.963) | 8.31 (6.683,10.06) | -2.497 (-2.621,-2.364) | 26.09 (21.487,30.881) | 437.806 (361.226,518.21) | 17.631 (14.212,21.787) | 190.736 (153.834,235.303) | -2.689 (-2.835,-2.548) |
| Slovenia | 0.533 (0.497,0.573) | 21.537 (20.076,23.136) | 0.406 (0.34,0.467) | 9.051 (7.565,10.484) | -2.741 (-2.928,-2.613) | 0.473 (0.441,0.508) | 19.069 (17.794,20.489) | 0.321 (0.268,0.371) | 6.783 (5.696,7.874) | -3.228 (-3.345,-3.096) | 11.203 (10.453,12.107) | 455.506 (424.575,492.78) | 6.105 (5.118,7.111) | 143.945 (120.282,167.681) | -3.586 (-3.712,-3.450) |
| Solomon Islands | 0.033 (0.021,0.045) | 23.85 (16.351,31.21) | 0.07 (0.052,0.094) | 19.238 (14.629,25.016) | -0.685 (-0.748,-0.619) | 0.032 (0.021,0.044) | 24.853 (17.862,32.457) | 0.066 (0.05,0.089) | 19.482 (14.999,25.158) | -0.779 (-0.837,-0.717) | 1.057 (0.663,1.445) | 654.376 (431.532,878.396) | 2.205 (1.603,2.972) | 516.88 (386.259,687.423) | -0.811 (-0.874,-0.752) |
| Somalia | 0.356 (0.249,0.462) | 13.966 (9.949,17.777) | 0.746 (0.499,0.975) | 11.413 (7.691,14.605) | -0.604 (-0.628,-0.577) | 0.35 (0.247,0.453) | 14.557 (10.337,18.539) | 0.735 (0.499,0.953) | 11.94 (8.178,15.243) | -0.586 (-0.614,-0.555) | 11.674 (8.12,15.36) | 386.31 (273.986,497.13) | 24.169 (16.341,32) | 311.72 (211.059,399.594) | -0.669 (-0.683,-0.654) |
| South Africa | 1.549 (1.174,1.73) | 7.257 (5.478,8.089) | 2.818 (2.38,3.183) | 6.137 (5.147,6.89) | -0.639 (-0.777,-0.509) | 1.52 (1.149,1.692) | 7.385 (5.592,8.266) | 2.747 (2.339,3.111) | 6.2 (5.176,6.967) | -0.646 (-0.779,-0.514) | 45.414 (35.47,50.624) | 194.213 (149.058,216.463) | 74.827 (64.451,86.4) | 151.301 (130.114,172.56) | -0.944 (-1.100,-0.777) |
| South Sudan | 0.268 (0.187,0.368) | 10.354 (7.404,14.129) | 0.363 (0.258,0.489) | 9.171 (6.601,12.181) | -0.364 (-0.392,-0.330) | 0.272 (0.193,0.37) | 10.819 (7.789,14.627) | 0.358 (0.255,0.479) | 9.531 (6.879,12.577) | -0.381 (-0.408,-0.352) | 7.592 (5.334,10.495) | 273.971 (193.839,375.392) | 10.972 (7.683,14.785) | 240.712 (171.44,322.971) | -0.390 (-0.417,-0.360) |
| Spain | 10.847 (10.077,11.525) | 20.055 (18.642,21.342) | 8.856 (7.717,9.734) | 9.057 (8.091,9.895) | -2.562 (-2.627,-2.505) | 8.941 (8.247,9.495) | 16.417 (15.178,17.448) | 6.401 (5.487,7.064) | 6.052 (5.323,6.596) | -3.151 (-3.215,-3.101) | 192.612 (180.253,204.003) | 367.112 (343.744,388.795) | 121.11 (108.295,131.056) | 134.365 (121.909,145.319) | -3.204 (-3.297,-3.134) |
| Sri Lanka | 0.919 (0.761,1.067) | 8.596 (7.114,10.006) | 1.099 (0.709,1.491) | 4.075 (2.647,5.533) | -2.242 (-2.390,-2.100) | 0.896 (0.742,1.042) | 8.804 (7.265,10.236) | 0.946 (0.618,1.285) | 3.573 (2.357,4.828) | -2.884 (-3.081,-2.736) | 25.487 (21.107,29.566) | 215.072 (178.019,249.704) | 23.982 (15.288,33.269) | 88.067 (56.472,121.77) | -2.749 (-2.882,-2.618) |
| Sudan | 1.887 (1.061,2.514) | 19.982 (11.18,26.516) | 2.932 (1.39,4.347) | 14.9 (6.975,21.748) | -0.940 (-0.954,-0.927) | 1.911 (1.076,2.54) | 20.898 (11.756,27.711) | 2.86 (1.36,4.185) | 15.188 (7.111,21.549) | -1.025 (-1.037,-1.014) | 55.572 (31.642,74.487) | 538.877 (305.168,720.781) | 82.63 (39.818,124.16) | 368.392 (175.774,544.073) | -1.220 (-1.236,-1.205) |
| Suriname | 0.027 (0.021,0.032) | 10.841 (8.63,12.586) | 0.044 (0.032,0.058) | 6.947 (5.12,9.163) | -1.311 (-1.522,-1.102) | 0.028 (0.022,0.032) | 11.238 (9.066,12.977) | 0.044 (0.032,0.058) | 7.066 (5.128,9.345) | -1.364 (-1.577,-1.145) | 0.731 (0.581,0.844) | 273.148 (217.437,314.28) | 1.127 (0.822,1.493) | 174.178 (127.353,231.084) | -1.349 (-1.589,-1.120) |
| Sweden | 1.723 (1.597,1.833) | 11.149 (10.395,11.831) | 1.002 (0.861,1.14) | 4.467 (3.883,5.055) | -2.905 (-3.042,-2.813) | 1.493 (1.384,1.586) | 9.389 (8.741,9.947) | 0.806 (0.687,0.915) | 3.376 (2.905,3.818) | -3.196 (-3.372,-3.086) | 28.889 (27.094,30.582) | 201.916 (190.012,212.861) | 14.02 (12.199,15.901) | 68.214 (59.872,77.04) | -3.456 (-3.616,-3.360) |
| Switzerland | 1.394 (1.275,1.506) | 13.232 (12.136,14.255) | 0.951 (0.82,1.073) | 5.172 (4.54,5.812) | -3.073 (-3.193,-2.993) | 1.144 (1.042,1.237) | 10.585 (9.683,11.429) | 0.733 (0.625,0.829) | 3.733 (3.25,4.216) | -3.376 (-3.481,-3.294) | 23.562 (21.813,25.346) | 235.768 (219.134,253.076) | 13.604 (11.936,15.322) | 79.79 (70.603,89.716) | -3.529 (-3.694,-3.405) |
| Syrian Arab Republic | 0.405 (0.312,0.509) | 7.775 (6.029,9.88) | 0.774 (0.57,1.035) | 6.254 (4.713,8.145) | -0.689 (-0.826,-0.581) | 0.403 (0.31,0.504) | 8.048 (6.238,10.145) | 0.716 (0.534,0.941) | 6.126 (4.652,7.85) | -0.843 (-0.966,-0.737) | 11.667 (8.932,14.646) | 198.961 (152.622,249.761) | 18.632 (13.606,24.805) | 137.063 (102.093,180.228) | -1.231 (-1.312,-1.148) |
| Taiwan  (Province of China) | 3.427 (2.983,3.756) | 21.459 (18.653,23.567) | 4.839 (4.272,5.352) | 11.509 (10.166,12.705) | -1.897 (-2.081,-1.688) | 2.941 (2.552,3.219) | 19.345 (16.76,21.148) | 3.799 (3.345,4.22) | 8.86 (7.839,9.811) | -2.579 (-2.740,-2.439) | 79.573 (69.145,87.025) | 469.625 (407.336,515.023) | 79.425 (71.168,87.813) | 193.696 (174.503,213.3) | -2.884 (-3.037,-2.751) |
| Tajikistan | 0.796 (0.648,0.935) | 28.493 (23.379,33.532) | 0.836 (0.65,1.093) | 14.24 (11.16,18.48) | -2.286 (-2.382,-2.181) | 0.795 (0.653,0.934) | 28.965 (23.881,34.092) | 0.817 (0.636,1.07) | 14.566 (11.451,18.935) | -2.230 (-2.343,-2.112) | 23.199 (18.954,27.237) | 784.076 (643.833,923.599) | 24.524 (19.04,32.616) | 368.199 (288.712,485.178) | -2.483 (-2.566,-2.396) |
| Thailand | 4.171 (2.93,5.096) | 11.473 (8.049,13.895) | 9.165 (5.448,12.17) | 8.711 (5.179,11.48) | -0.954 (-1.072,-0.855) | 3.968 (2.792,4.826) | 11.408 (8.048,13.656) | 7.748 (4.677,10.227) | 7.275 (4.377,9.581) | -1.487 (-1.603,-1.390) | 117.152 (82.369,142.948) | 293.115 (205.882,356.347) | 202.764 (117.04,270.598) | 197.231 (113.703,261.039) | -1.296 (-1.417,-1.191) |
| Timor-Leste | 0.03 (0.02,0.04) | 10.173 (7.031,13.319) | 0.064 (0.047,0.086) | 7.587 (5.55,10.176) | -0.968 (-1.034,-0.910) | 0.029 (0.02,0.039) | 10.665 (7.392,13.91) | 0.063 (0.046,0.085) | 7.732 (5.716,10.342) | -1.050 (-1.105,-1.002) | 0.946 (0.63,1.292) | 271.994 (185.384,361.909) | 1.669 (1.198,2.228) | 185.927 (133.825,248.75) | -1.240 (-1.308,-1.182) |
| Togo | 0.141 (0.108,0.17) | 11.618 (9.037,13.889) | 0.414 (0.273,0.549) | 11.217 (7.806,14.699) | -0.114 (-0.151,-0.082) | 0.142 (0.109,0.171) | 12.243 (9.612,14.619) | 0.41 (0.269,0.541) | 11.778 (8.136,15.347) | -0.122 (-0.157,-0.091) | 4.125 (3.091,5.01) | 297.836 (227.618,360.229) | 11.85 (7.57,15.729) | 278.329 (183.609,367.826) | -0.228 (-0.263,-0.196) |
| Tokelau | 0 (0,0) | 16.958 (13.112,21.393) | 0 (0,0) | 11.232 (8.842,14.291) | -1.313 (-1.337,-1.288) | 0 (0,0) | 17.77 (13.781,22.32) | 0 (0,0) | 10.965 (8.549,13.869) | -1.541 (-1.563,-1.521) | 0.006 (0.004,0.007) | 434.915 (332.473,561.06) | 0.004 (0.003,0.005) | 270.154 (212.628,346.708) | -1.514 (-1.557,-1.470) |
| Tonga | 0.011 (0.009,0.014) | 20.162 (16.65,25.651) | 0.014 (0.011,0.018) | 17.087 (13.468,22.228) | -0.533 (-0.591,-0.479) | 0.011 (0.009,0.014) | 20.746 (17.158,26.28) | 0.013 (0.01,0.017) | 16.745 (13.241,21.58) | -0.676 (-0.735,-0.621) | 0.295 (0.242,0.373) | 506.265 (418.141,642.177) | 0.331 (0.256,0.436) | 403.786 (313.384,530.182) | -0.725 (-0.786,-0.673) |
| Trinidad and Tobago | 0.091 (0.085,0.096) | 11.117 (10.475,11.768) | 0.1 (0.076,0.129) | 5.263 (4.003,6.759) | -2.135 (-2.385,-1.868) | 0.092 (0.087,0.098) | 11.611 (10.928,12.267) | 0.098 (0.075,0.126) | 5.174 (3.942,6.586) | -2.339 (-2.582,-2.067) | 2.249 (2.122,2.382) | 262.734 (248.145,277.731) | 2.421 (1.816,3.17) | 127.389 (95.663,166.514) | -2.037 (-2.291,-1.762) |
| Tunisia | 0.316 (0.24,0.389) | 6.512 (5.02,7.978) | 0.598 (0.419,0.826) | 4.553 (3.22,6.241) | -1.159 (-1.226,-1.096) | 0.313 (0.24,0.384) | 6.695 (5.196,8.181) | 0.553 (0.39,0.746) | 4.302 (3.068,5.77) | -1.488 (-1.533,-1.443) | 8.261 (6.367,10.166) | 157.017 (120.329,193.532) | 13.65 (9.456,18.618) | 100.655 (70.194,136.844) | -1.457 (-1.572,-1.401) |
| Türkiye | 8.422 (6.47,9.895) | 24.115 (18.743,28.26) | 11.57 (8.585,14.295) | 12.472 (9.305,15.381) | -2.424 (-2.623,-2.244) | 8.31 (6.43,9.757) | 24.625 (19.264,28.791) | 10.491 (7.917,12.827) | 11.551 (8.732,14.089) | -2.534 (-2.702,-2.367) | 240.297 (179.011,283.344) | 629.557 (478.373,739.301) | 254.283 (186.67,314.735) | 266.954 (196.482,329.885) | -2.485 (-2.728,-2.289) |
| Turkmenistan | 0.426 (0.393,0.461) | 21.694 (19.905,23.545) | 0.409 (0.31,0.536) | 9.77 (7.43,12.701) | -1.200 (-1.214,-1.185) | 0.421 (0.387,0.456) | 21.951 (20.09,23.839) | 0.39 (0.3,0.51) | 9.587 (7.406,12.472) | -1.333 (-1.349,-1.318) | 12.443 (11.513,13.4) | 590.618 (545.062,637.835) | 11.696 (8.982,15.329) | 260.441 (200.29,340.341) | -1.434 (-1.453,-1.415) |
| Tuvalu | 0.001 (0.001,0.002) | 21.833 (17.186,27.214) | 0.002 (0.001,0.002) | 15.032 (11.961,18.26) | -2.110 (-2.200,-2.040) | 0.001 (0.001,0.002) | 22.796 (18.024,28.337) | 0.001 (0.001,0.002) | 15.043 (12.012,18.264) | -2.434 (-2.515,-2.366) | 0.043 (0.033,0.054) | 594.757 (458.362,744.658) | 0.041 (0.031,0.051) | 380.599 (293.812,469.07) | -2.755 (-2.849,-2.680) |
| Uganda | 0.558 (0.434,0.7) | 8.737 (6.812,10.877) | 0.916 (0.681,1.189) | 6.117 (4.682,7.876) | -1.163 (-1.192,-1.132) | 0.565 (0.445,0.707) | 9.18 (7.27,11.466) | 0.905 (0.681,1.179) | 6.365 (4.957,8.248) | -1.185 (-1.215,-1.152) | 15.962 (12.428,20.36) | 227.087 (178.16,285.496) | 26.999 (19.597,35.632) | 156.636 (117.373,204.896) | -1.212 (-1.253,-1.171) |
| Ukraine | 21.645 (20.268,23.001) | 30.222 (28.374,32.059) | 9.282 (6.806,12.045) | 12.221 (8.91,15.847) | -3.155 (-3.316,-2.968) | 18.809 (17.66,19.943) | 26.296 (24.679,27.821) | 7.799 (5.722,10.109) | 10.216 (7.45,13.295) | -3.253 (-3.424,-3.069) | 514.431 (482.331,544.895) | 733.387 (688.037,776.963) | 202.545 (144.647,265.153) | 281.196 (199.656,369.746) | -3.398 (-3.558,-3.221) |
| United Arab Emirates | 0.064 (0.047,0.085) | 14.36 (10.79,18.679) | 0.23 (0.176,0.315) | 7.821 (6.14,10.052) | -2.051 (-2.709,-1.467) | 0.06 (0.045,0.08) | 14.817 (11.208,19.133) | 0.196 (0.152,0.264) | 8.056 (6.391,10.346) | -2.053 (-2.681,-1.490) | 1.99 (1.451,2.662) | 350.234 (260.516,458.276) | 6.484 (4.962,8.904) | 158.37 (126.2,206.884) | -2.608 (-3.093,-2.172) |
| United Kingdom | 13.3 (12.647,13.629) | 14.342 (13.68,14.684) | 8.021 (7.308,8.4) | 5.956 (5.497,6.209) | -2.766 (-2.814,-2.720) | 11.473 (10.882,11.775) | 12.189 (11.579,12.5) | 6.5 (5.839,6.828) | 4.606 (4.198,4.818) | -3.061 (-3.122,-3.006) | 227.837 (219.793,232.548) | 258.508 (250.338,263.366) | 115.891 (107.531,120.479) | 93.076 (87.827,96.361) | -3.222 (-3.281,-3.170) |
| United Republic of Tanzania | 0.989 (0.702,1.206) | 9.07 (6.507,10.972) | 1.522 (1.112,1.909) | 5.919 (4.38,7.407) | -1.356 (-1.378,-1.334) | 0.994 (0.708,1.206) | 9.476 (6.755,11.467) | 1.516 (1.127,1.907) | 6.161 (4.622,7.708) | -1.375 (-1.402,-1.355) | 28.702 (20.478,35.772) | 239.19 (169.446,293.386) | 43.637 (31.569,55.551) | 150.737 (111.507,190.014) | -1.488 (-1.514,-1.463) |
| United States of America | 25.711 (24.106,26.591) | 8.041 (7.568,8.301) | 28.458 (26.39,29.871) | 5.068 (4.743,5.3) | -1.508 (-1.545,-1.469) | 17.316 (16.087,17.977) | 5.355 (4.995,5.549) | 16.445 (15.03,17.351) | 2.836 (2.619,2.978) | -2.073 (-2.124,-2.015) | 383.157 (366.567,393.935) | 124.904 (120.009,128.284) | 363.139 (342.1,377.925) | 69.162 (65.836,71.769) | -1.911 (-1.972,-1.855) |
| United States Virgin Islands | 0.01 (0.008,0.012) | 11.808 (9.514,14.075) | 0.01 (0.007,0.013) | 5.708 (4.386,7.52) | -2.326 (-2.489,-2.218) | 0.009 (0.007,0.011) | 11.903 (9.63,14.213) | 0.01 (0.007,0.013) | 5.545 (4.245,7.246) | -2.440 (-2.596,-2.323) | 0.25 (0.199,0.302) | 279.168 (222.328,335.69) | 0.213 (0.161,0.28) | 133.18 (101.829,173.735) | -2.372 (-2.487,-2.294) |
| Uruguay | 0.654 (0.614,0.692) | 16.814 (15.775,17.811) | 0.638 (0.581,0.699) | 11.572 (10.574,12.638) | -1.167 (-1.268,-1.037) | 0.647 (0.606,0.682) | 16.57 (15.545,17.495) | 0.615 (0.556,0.678) | 10.724 (9.787,11.777) | -1.359 (-1.467,-1.216) | 14.376 (13.589,15.193) | 378.97 (358.355,400.752) | 12.708 (11.626,13.931) | 248.241 (227.826,272.449) | -1.333 (-1.423,-1.219) |
| Uzbekistan | 2.427 (2.203,2.647) | 20.701 (18.729,22.66) | 2.141 (1.723,2.63) | 7.793 (6.291,9.588) | -3.221 (-3.450,-3.072) | 2.385 (2.161,2.607) | 20.616 (18.609,22.629) | 2.036 (1.634,2.471) | 7.662 (6.168,9.313) | -3.256 (-3.476,-3.113) | 70.637 (64.66,76.684) | 574.865 (523.87,626.623) | 61.965 (49.85,75.39) | 207.263 (166.72,251.202) | -3.321 (-3.476,-3.222) |
| Vanuatu | 0.013 (0.009,0.017) | 20.011 (14.587,26.542) | 0.028 (0.022,0.036) | 15.993 (12.706,20.155) | -0.734 (-0.764,-0.693) | 0.012 (0.009,0.016) | 20.833 (15.486,27.6) | 0.027 (0.021,0.034) | 16.305 (12.83,20.56) | -0.797 (-0.826,-0.758) | 0.402 (0.285,0.539) | 536.986 (390.787,715.363) | 0.854 (0.661,1.101) | 420.578 (330.646,538.13) | -0.803 (-0.836,-0.756) |
| Venezuela  (Bolivarian Republic of) | 1.848 (1.741,1.956) | 19.481 (18.264,20.635) | 3.285 (2.503,4.207) | 11.114 (8.547,14.146) | -1.741 (-2.072,-1.444) | 1.853 (1.748,1.957) | 20.124 (18.795,21.297) | 3.179 (2.408,4.066) | 10.902 (8.334,13.857) | -1.887 (-2.208,-1.620) | 48.323 (45.794,50.796) | 470.733 (445.242,495.73) | 80.661 (60.142,104.673) | 266.011 (199.23,343.814) | -1.778 (-2.140,-1.460) |
| Viet Nam | 6.062 (4.537,7.706) | 14.855 (11.194,18.854) | 8.777 (6.72,11.483) | 8.645 (6.813,11.306) | -1.727 (-1.752,-1.698) | 5.983 (4.487,7.555) | 14.904 (11.348,18.833) | 7.561 (5.902,9.811) | 7.691 (6.104,9.914) | -2.089 (-2.119,-2.062) | 165.208 (122.343,213.101) | 391.417 (288.459,502.934) | 210.212 (157.4,276.307) | 196.803 (150.423,257.664) | -2.176 (-2.198,-2.156) |
| Yemen | 1.181 (0.62,1.622) | 23.693 (12.751,31.973) | 2.611 (1.155,3.686) | 18.693 (8.447,26.036) | -0.735 (-0.772,-0.699) | 1.176 (0.632,1.615) | 24.619 (13.484,33.211) | 2.583 (1.125,3.662) | 19.385 (8.712,27.138) | -0.740 (-0.774,-0.705) | 35.13 (18.481,49.121) | 634.926 (340.71,874.844) | 73.612 (31.02,106.548) | 464.577 (201.283,660.181) | -0.972 (-1.009,-0.935) |
| Zambia | 0.291 (0.209,0.346) | 10.008 (7.208,11.833) | 0.525 (0.355,0.717) | 7.252 (5.054,9.633) | -1.020 (-1.047,-0.993) | 0.292 (0.211,0.346) | 10.469 (7.586,12.374) | 0.513 (0.35,0.703) | 7.514 (5.238,9.95) | -1.046 (-1.077,-1.017) | 8.878 (6.447,10.59) | 269.874 (195.19,321.029) | 16.089 (10.669,23.053) | 189.576 (129.702,259.095) | -1.131 (-1.158,-1.108) |
| Zimbabwe | 0.445 (0.354,0.54) | 11.339 (9.08,13.824) | 0.935 (0.674,1.204) | 13.234 (9.775,16.534) | 0.530 (0.483,0.580) | 0.447 (0.353,0.547) | 11.904 (9.493,14.611) | 0.922 (0.671,1.185) | 13.763 (10.267,17.013) | 0.561 (0.491,0.640) | 12.268 (9.533,15.043) | 280.658 (219.627,345.24) | 28.282 (19.947,37.075) | 348.52 (253.69,447.942) | 0.711 (0.658,0.770) |

Supplementary Table S4. cocurrence pattern of incidence

| location | val_EC | EC_level | val_SC | SC_level | EC_num | SC_num | region_type |
| --- | --- | --- | --- | --- | --- | --- | --- |
| Afghanistan | 7.2617865 | High | 32.92408143 | High | 4 | 4 | Consistent |
| Albania | 1.410173952 | Low | 10.59968576 | Upper-middle | 1 | 3 | Stomach cancer-dominant |
| Algeria | 0.699030935 | Low | 3.751156912 | Low | 1 | 1 | Consistent |
| American Samoa | 1.564678668 | Low | 17.98974497 | High | 1 | 4 | Stomach cancer-dominant |
| Andorra | 1.346917161 | Low | 8.368272472 | Lower-middle | 1 | 2 | Stomach cancer-dominant |
| Angola | 7.969241305 | High | 8.394924664 | Lower-middle | 4 | 2 | Esophageal cancer-dominant |
| Antigua and Barbuda | 2.82987489 | Lower-middle | 10.12836756 | Upper-middle | 2 | 3 | Stomach cancer-dominant |
| Argentina | 4.125962687 | Upper-middle | 8.85451729 | Lower-middle | 3 | 2 | Esophageal cancer-dominant |
| Armenia | 1.004251523 | Low | 11.19181529 | Upper-middle | 1 | 3 | Stomach cancer-dominant |
| Australia | 4.058843276 | Upper-middle | 5.743620302 | Low | 3 | 1 | Esophageal cancer-dominant |
| Austria | 2.668813773 | Lower-middle | 6.688234302 | Lower-middle | 2 | 2 | Consistent |
| Azerbaijan | 5.354276593 | High | 14.4025598 | High | 4 | 4 | Consistent |
| Bahamas | 5.126096474 | Upper-middle | 8.893198168 | Lower-middle | 3 | 2 | Esophageal cancer-dominant |
| Bahrain | 2.170672552 | Lower-middle | 6.994254382 | Lower-middle | 2 | 2 | Consistent |
| Bangladesh | 3.274647476 | Upper-middle | 5.389321448 | Low | 3 | 1 | Esophageal cancer-dominant |
| Barbados | 5.007964912 | Upper-middle | 9.895875608 | Upper-middle | 3 | 3 | Consistent |
| Belarus | 3.045024456 | Lower-middle | 15.35557245 | High | 2 | 4 | Stomach cancer-dominant |
| Belgium | 4.594827105 | Upper-middle | 5.374742905 | Low | 3 | 1 | Esophageal cancer-dominant |
| Belize | 2.127198195 | Lower-middle | 9.336746488 | Upper-middle | 2 | 3 | Stomach cancer-dominant |
| Benin | 5.018821835 | Upper-middle | 9.138904949 | Upper-middle | 3 | 3 | Consistent |
| Bermuda | 4.185457794 | Upper-middle | 5.628778894 | Low | 3 | 1 | Esophageal cancer-dominant |
| Bhutan | 3.556597288 | Upper-middle | 5.523173886 | Low | 3 | 1 | Esophageal cancer-dominant |
| Bolivia (Plurinational State of) | 2.209514756 | Lower-middle | 30.81282357 | High | 2 | 4 | Stomach cancer-dominant |
| Bosnia and Herzegovina | 2.129885368 | Lower-middle | 9.114567041 | Upper-middle | 2 | 3 | Stomach cancer-dominant |
| Botswana | 8.586028351 | High | 5.501114499 | Low | 4 | 1 | Esophageal cancer-dominant |
| Brazil | 4.928399242 | Upper-middle | 10.02421212 | Upper-middle | 3 | 3 | Consistent |
| Brunei Darussalam | 2.26041849 | Lower-middle | 12.96014089 | High | 2 | 4 | Stomach cancer-dominant |
| Bulgaria | 2.257587843 | Lower-middle | 12.05544688 | Upper-middle | 2 | 3 | Stomach cancer-dominant |
| Burkina Faso | 5.438064386 | High | 11.20091573 | Upper-middle | 4 | 3 | Esophageal cancer-dominant |
| Burundi | 10.63904669 | High | 7.49734763 | Lower-middle | 4 | 2 | Esophageal cancer-dominant |
| Cabo Verde | 15.15394144 | High | 23.46628122 | High | 4 | 4 | Consistent |
| Cambodia | 2.960057729 | Lower-middle | 10.47330741 | Upper-middle | 2 | 3 | Stomach cancer-dominant |
| Cameroon | 6.273324722 | High | 9.861796408 | Upper-middle | 4 | 3 | Esophageal cancer-dominant |
| Canada | 4.199477118 | Upper-middle | 6.502902246 | Lower-middle | 3 | 2 | Esophageal cancer-dominant |
| Central African Republic | 10.71626589 | High | 12.5373756 | High | 4 | 4 | Consistent |
| Chad | 5.442274974 | High | 12.74939948 | High | 4 | 4 | Consistent |
| Chile | 3.07318096 | Lower-middle | 16.94429264 | High | 2 | 4 | Stomach cancer-dominant |
| China | 15.04251301 | High | 29.05268307 | High | 4 | 4 | Consistent |
| Colombia | 1.76615464 | Low | 14.39007611 | High | 1 | 4 | Stomach cancer-dominant |
| Comoros | 12.49690058 | High | 6.690228691 | Lower-middle | 4 | 2 | Esophageal cancer-dominant |
| Congo | 10.48425964 | High | 8.535650478 | Lower-middle | 4 | 2 | Esophageal cancer-dominant |
| Cook Islands | 2.536209898 | Lower-middle | 7.02351813 | Lower-middle | 2 | 2 | Consistent |
| Costa Rica | 1.563501613 | Low | 17.93363135 | High | 1 | 4 | Stomach cancer-dominant |
| C?te d'Ivoire | 1.300003553 | Low | 3.323152882 | Low | 1 | 1 | Consistent |
| Croatia | 2.674821511 | Lower-middle | 10.69878 | Upper-middle | 2 | 3 | Stomach cancer-dominant |
| Cuba | 4.967588789 | Upper-middle | 5.936807666 | Low | 3 | 1 | Esophageal cancer-dominant |
| Cyprus | 1.470321304 | Low | 6.764013147 | Lower-middle | 1 | 2 | Stomach cancer-dominant |
| Czechia | 3.22837926 | Upper-middle | 6.997536193 | Lower-middle | 3 | 2 | Esophageal cancer-dominant |
| Democratic People's Republic of Korea | 9.190109616 | High | 24.01774069 | High | 4 | 4 | Consistent |
| Democratic Republic of the Congo | 7.961934564 | High | 8.39993294 | Lower-middle | 4 | 2 | Esophageal cancer-dominant |
| Denmark | 5.096833073 | Upper-middle | 6.268577559 | Low | 3 | 1 | Esophageal cancer-dominant |
| Djibouti | 12.11180373 | High | 7.246201768 | Lower-middle | 4 | 2 | Esophageal cancer-dominant |
| Dominica | 4.910962888 | Upper-middle | 19.63256072 | High | 3 | 4 | Stomach cancer-dominant |
| Dominican Republic | 2.171946402 | Lower-middle | 6.93783753 | Lower-middle | 2 | 2 | Consistent |
| Ecuador | 1.191391603 | Low | 18.47311476 | High | 1 | 4 | Stomach cancer-dominant |
| Egypt | 1.034714787 | Low | 7.193258498 | Lower-middle | 1 | 2 | Stomach cancer-dominant |
| El Salvador | 1.68542938 | Low | 18.16449287 | High | 1 | 4 | Stomach cancer-dominant |
| Equatorial Guinea | 8.779428482 | High | 6.412625818 | Low | 4 | 1 | Esophageal cancer-dominant |
| Eritrea | 14.47131745 | High | 9.705632942 | Upper-middle | 4 | 3 | Esophageal cancer-dominant |
| Estonia | 2.750425644 | Lower-middle | 12.49958175 | High | 2 | 4 | Stomach cancer-dominant |
| Eswatini | 16.68361017 | High | 9.393072787 | Upper-middle | 4 | 3 | Esophageal cancer-dominant |
| Ethiopia | 4.971590843 | Upper-middle | 6.367564389 | Low | 3 | 1 | Esophageal cancer-dominant |
| Fiji | 2.62541692 | Lower-middle | 8.159884044 | Lower-middle | 2 | 2 | Consistent |
| Finland | 3.616584419 | Upper-middle | 5.887476405 | Low | 3 | 1 | Esophageal cancer-dominant |
| France | 4.988077642 | Upper-middle | 6.564575485 | Lower-middle | 3 | 2 | Esophageal cancer-dominant |
| Gabon | 10.46198729 | High | 7.690342182 | Lower-middle | 4 | 2 | Esophageal cancer-dominant |
| Gambia | 1.931227857 | Low | 3.410034141 | Low | 1 | 1 | Consistent |
| Georgia | 1.059727449 | Low | 12.20883693 | Upper-middle | 1 | 3 | Stomach cancer-dominant |
| Germany | 4.597323122 | Upper-middle | 9.247018737 | Upper-middle | 3 | 3 | Consistent |
| Ghana | 3.510607732 | Upper-middle | 7.351601297 | Lower-middle | 3 | 2 | Esophageal cancer-dominant |
| Global | 6.654785354 | High | 14.32755942 | High | 4 | 4 | Consistent |
| Greece | 1.817343807 | Low | 8.702947271 | Lower-middle | 1 | 2 | Stomach cancer-dominant |
| Greenland | 10.80842831 | High | 9.538983793 | Upper-middle | 4 | 3 | Esophageal cancer-dominant |
| Grenada | 4.628483634 | Upper-middle | 8.476131798 | Lower-middle | 3 | 2 | Esophageal cancer-dominant |
| Guam | 1.883032459 | Low | 5.768097536 | Low | 1 | 1 | Consistent |
| Guatemala | 1.648524454 | Low | 23.68723671 | High | 1 | 4 | Stomach cancer-dominant |
| Guinea | 1.3425197 | Low | 9.356148251 | Upper-middle | 1 | 3 | Stomach cancer-dominant |
| Guinea-Bissau | 7.3066806 | High | 15.24921487 | High | 4 | 4 | Consistent |
| Guyana | 2.035974202 | Low | 7.773329764 | Lower-middle | 1 | 2 | Stomach cancer-dominant |
| Haiti | 3.94569449 | Upper-middle | 17.63006729 | High | 3 | 4 | Stomach cancer-dominant |
| Honduras | 1.452085705 | Low | 19.6824163 | High | 1 | 4 | Stomach cancer-dominant |
| Hungary | 3.171741993 | Lower-middle | 7.785727669 | Lower-middle | 2 | 2 | Consistent |
| Iceland | 4.531573479 | Upper-middle | 5.548766348 | Low | 3 | 1 | Esophageal cancer-dominant |
| India | 3.071393078 | Lower-middle | 5.825850912 | Low | 2 | 1 | Esophageal cancer-dominant |
| Indonesia | 1.620966294 | Low | 7.505495632 | Lower-middle | 1 | 2 | Stomach cancer-dominant |
| Iran (Islamic Republic of) | 3.775397796 | Upper-middle | 13.90649735 | High | 3 | 4 | Stomach cancer-dominant |
| Iraq | 1.178735946 | Low | 5.425377357 | Low | 1 | 1 | Consistent |
| Ireland | 6.13478274 | High | 5.718221438 | Low | 4 | 1 | Esophageal cancer-dominant |
| Israel | 1.543966127 | Low | 5.956054652 | Low | 1 | 1 | Consistent |
| Italy | 1.772789224 | Low | 10.10430261 | Upper-middle | 1 | 3 | Stomach cancer-dominant |
| Jamaica | 2.970134578 | Lower-middle | 9.246282397 | Upper-middle | 2 | 3 | Stomach cancer-dominant |
| Japan | 6.216333614 | High | 25.54346142 | High | 4 | 4 | Consistent |
| Jordan | 0.853039335 | Low | 4.077798956 | Low | 1 | 1 | Consistent |
| Kazakhstan | 4.688953504 | Upper-middle | 11.29126153 | Upper-middle | 3 | 3 | Consistent |
| Kenya | 11.74602508 | High | 7.930758924 | Lower-middle | 4 | 2 | Esophageal cancer-dominant |
| Kiribati | 5.515858231 | High | 21.33476934 | High | 4 | 4 | Consistent |
| Kuwait | 0.938464588 | Low | 3.040454282 | Low | 1 | 1 | Consistent |
| Kyrgyzstan | 2.819432222 | Lower-middle | 15.79371194 | High | 2 | 4 | Stomach cancer-dominant |
| Lao People's Democratic Republic | 2.481068805 | Lower-middle | 8.397867161 | Lower-middle | 2 | 2 | Consistent |
| Latvia | 2.911666037 | Lower-middle | 12.64009169 | High | 2 | 4 | Stomach cancer-dominant |
| Lebanon | 0.916969948 | Low | 5.754700855 | Low | 1 | 1 | Consistent |
| Lesotho | 15.82144103 | High | 12.07880767 | Upper-middle | 4 | 3 | Esophageal cancer-dominant |
| Liberia | 5.651565548 | High | 9.688839529 | Upper-middle | 4 | 3 | Esophageal cancer-dominant |
| Libya | 1.697033588 | Low | 6.773951056 | Lower-middle | 1 | 2 | Stomach cancer-dominant |
| Lithuania | 3.682059038 | Upper-middle | 12.66496064 | High | 3 | 4 | Stomach cancer-dominant |
| Luxembourg | 3.734502277 | Upper-middle | 5.289528443 | Low | 3 | 1 | Esophageal cancer-dominant |
| Madagascar | 10.79134888 | High | 6.200161296 | Low | 4 | 1 | Esophageal cancer-dominant |
| Malawi | 26.06417649 | High | 3.125460479 | Low | 4 | 1 | Esophageal cancer-dominant |
| Malaysia | 2.646463947 | Lower-middle | 6.794075811 | Lower-middle | 2 | 2 | Consistent |
| Maldives | 1.207306017 | Low | 3.028762996 | Low | 1 | 1 | Consistent |
| Mali | 2.445998438 | Lower-middle | 16.3274299 | High | 2 | 4 | Stomach cancer-dominant |
| Malta | 2.411427683 | Lower-middle | 5.622790521 | Low | 2 | 1 | Esophageal cancer-dominant |
| Marshall Islands | 2.484684261 | Lower-middle | 17.72971871 | High | 2 | 4 | Stomach cancer-dominant |
| Mauritania | 5.211760055 | High | 8.697764254 | Lower-middle | 4 | 2 | Esophageal cancer-dominant |
| Mauritius | 3.547085873 | Upper-middle | 11.02194116 | Upper-middle | 3 | 3 | Consistent |
| Mexico | 1.433233605 | Low | 9.375811699 | Upper-middle | 1 | 3 | Stomach cancer-dominant |
| Micronesia (Federated States of) | 2.671966971 | Lower-middle | 17.12657567 | High | 2 | 4 | Stomach cancer-dominant |
| Monaco | 6.212620032 | High | 8.73306394 | Lower-middle | 4 | 2 | Esophageal cancer-dominant |
| Mongolia | 16.24761197 | High | 36.82652505 | High | 4 | 4 | Consistent |
| Montenegro | 2.217564507 | Lower-middle | 8.37318241 | Lower-middle | 2 | 2 | Consistent |
| Morocco | 0.848127681 | Low | 2.495523428 | Low | 1 | 1 | Consistent |
| Mozambique | 8.978148592 | High | 7.556828731 | Lower-middle | 4 | 2 | Esophageal cancer-dominant |
| Myanmar | 2.252907355 | Lower-middle | 7.395559369 | Lower-middle | 2 | 2 | Consistent |
| Namibia | 2.350444618 | Lower-middle | 2.704389079 | Low | 2 | 1 | Esophageal cancer-dominant |
| Nauru | 3.254941063 | Upper-middle | 21.09157605 | High | 3 | 4 | Stomach cancer-dominant |
| Nepal | 3.773475445 | Upper-middle | 6.110200476 | Low | 3 | 1 | Esophageal cancer-dominant |
| Netherlands | 6.884377258 | High | 4.140305642 | Low | 4 | 1 | Esophageal cancer-dominant |
| New Zealand | 4.039250403 | Upper-middle | 6.926320125 | Lower-middle | 3 | 2 | Esophageal cancer-dominant |
| Nicaragua | 0.824982595 | Low | 9.891253716 | Upper-middle | 1 | 3 | Stomach cancer-dominant |
| Niger | 4.114025451 | Upper-middle | 10.77806413 | Upper-middle | 3 | 3 | Consistent |
| Nigeria | 4.254177037 | Upper-middle | 2.476979434 | Low | 3 | 1 | Esophageal cancer-dominant |
| Niue | 2.408286154 | Lower-middle | 11.99232189 | Upper-middle | 2 | 3 | Stomach cancer-dominant |
| North Macedonia | 1.372203285 | Low | 14.27351581 | High | 1 | 4 | Stomach cancer-dominant |
| Northern Mariana Islands | 2.59577433 | Lower-middle | 14.40067219 | High | 2 | 4 | Stomach cancer-dominant |
| Norway | 3.322086811 | Upper-middle | 5.349891794 | Low | 3 | 1 | Esophageal cancer-dominant |
| Oman | 1.587214453 | Low | 5.750021118 | Low | 1 | 1 | Consistent |
| Pakistan | 6.255625565 | High | 4.687947437 | Low | 4 | 1 | Esophageal cancer-dominant |
| Palau | 2.886409379 | Lower-middle | 19.65150332 | High | 2 | 4 | Stomach cancer-dominant |
| Palestine | 0.897197923 | Low | 6.389956093 | Low | 1 | 1 | Consistent |
| Panama | 1.204949394 | Low | 10.46591802 | Upper-middle | 1 | 3 | Stomach cancer-dominant |
| Papua New Guinea | 1.580514223 | Low | 13.82458701 | High | 1 | 4 | Stomach cancer-dominant |
| Paraguay | 4.014914577 | Upper-middle | 8.734020422 | Lower-middle | 3 | 2 | Esophageal cancer-dominant |
| Peru | 1.265814432 | Low | 20.61870394 | High | 1 | 4 | Stomach cancer-dominant |
| Philippines | 1.338900636 | Low | 4.481447508 | Low | 1 | 1 | Consistent |
| Poland | 2.982648555 | Lower-middle | 8.049639409 | Lower-middle | 2 | 2 | Consistent |
| Portugal | 3.43937443 | Upper-middle | 12.90197706 | High | 3 | 4 | Stomach cancer-dominant |
| Puerto Rico | 2.420027495 | Lower-middle | 4.776588212 | Low | 2 | 1 | Esophageal cancer-dominant |
| Qatar | 2.591046765 | Lower-middle | 6.463315891 | Lower-middle | 2 | 2 | Consistent |
| Republic of Korea | 3.523664743 | Upper-middle | 25.76248228 | High | 3 | 4 | Stomach cancer-dominant |
| Republic of Moldova | 1.864627228 | Low | 9.68018873 | Upper-middle | 1 | 3 | Stomach cancer-dominant |
| Romania | 2.521781617 | Lower-middle | 11.57014437 | Upper-middle | 2 | 3 | Stomach cancer-dominant |
| Russian Federation | 3.325373065 | Upper-middle | 15.69329766 | High | 3 | 4 | Stomach cancer-dominant |
| Rwanda | 11.27913131 | High | 6.603262582 | Lower-middle | 4 | 2 | Esophageal cancer-dominant |
| Saint Kitts and Nevis | 3.806057392 | Upper-middle | 8.981198333 | Lower-middle | 3 | 2 | Esophageal cancer-dominant |
| Saint Lucia | 4.144980006 | Upper-middle | 10.78458413 | Upper-middle | 3 | 3 | Consistent |
| Saint Vincent and the Grenadines | 2.471644248 | Lower-middle | 10.21714193 | Upper-middle | 2 | 3 | Stomach cancer-dominant |
| Samoa | 1.116288207 | Low | 10.82749553 | Upper-middle | 1 | 3 | Stomach cancer-dominant |
| San Marino | 0.958610388 | Low | 11.69131007 | Upper-middle | 1 | 3 | Stomach cancer-dominant |
| Sao Tome and Principe | 4.615495764 | Upper-middle | 14.63872445 | High | 3 | 4 | Stomach cancer-dominant |
| Saudi Arabia | 1.752146184 | Low | 3.892637573 | Low | 1 | 1 | Consistent |
| Senegal | 5.061366399 | Upper-middle | 10.20036085 | Upper-middle | 3 | 3 | Consistent |
| Serbia | 2.142491296 | Lower-middle | 8.236994574 | Lower-middle | 2 | 2 | Consistent |
| Seychelles | 4.726589572 | Upper-middle | 6.474435543 | Lower-middle | 3 | 2 | Esophageal cancer-dominant |
| Sierra Leone | 4.828659289 | Upper-middle | 9.887589819 | Upper-middle | 3 | 3 | Consistent |
| Singapore | 2.588426816 | Lower-middle | 8.473360098 | Lower-middle | 2 | 2 | Consistent |
| Slovakia | 3.247869706 | Upper-middle | 10.4606101 | Upper-middle | 3 | 3 | Consistent |
| Slovenia | 2.435274407 | Lower-middle | 9.05123339 | Upper-middle | 2 | 3 | Stomach cancer-dominant |
| Solomon Islands | 2.404333126 | Lower-middle | 19.23812723 | High | 2 | 4 | Stomach cancer-dominant |
| Somalia | 14.90584306 | High | 11.41333202 | Upper-middle | 4 | 3 | Esophageal cancer-dominant |
| South Africa | 10.42678661 | High | 6.136521466 | Low | 4 | 1 | Esophageal cancer-dominant |
| South Sudan | 15.15607922 | High | 9.171009254 | Upper-middle | 4 | 3 | Esophageal cancer-dominant |
| Spain | 2.813779033 | Lower-middle | 9.057495435 | Upper-middle | 2 | 3 | Stomach cancer-dominant |
| Sri Lanka | 4.258092433 | Upper-middle | 4.075054828 | Low | 3 | 1 | Esophageal cancer-dominant |
| Sudan | 4.237415808 | Upper-middle | 14.89986455 | High | 3 | 4 | Stomach cancer-dominant |
| Suriname | 1.605331368 | Low | 6.947417738 | Lower-middle | 1 | 2 | Stomach cancer-dominant |
| Sweden | 2.862236724 | Lower-middle | 4.466998414 | Low | 2 | 1 | Esophageal cancer-dominant |
| Switzerland | 3.224038905 | Lower-middle | 5.172151796 | Low | 2 | 1 | Esophageal cancer-dominant |
| Syrian Arab Republic | 0.889621855 | Low | 6.253634578 | Low | 1 | 1 | Consistent |
| Taiwan (Province of China) | 9.302011934 | High | 11.50888551 | Upper-middle | 4 | 3 | Esophageal cancer-dominant |
| Tajikistan | 6.131337364 | High | 14.24043374 | High | 4 | 4 | Consistent |
| Thailand | 4.724689678 | Upper-middle | 8.710849693 | Lower-middle | 3 | 2 | Esophageal cancer-dominant |
| Timor-Leste | 2.094170745 | Low | 7.586789492 | Lower-middle | 1 | 2 | Stomach cancer-dominant |
| Togo | 5.822075143 | High | 11.21707833 | Upper-middle | 4 | 3 | Esophageal cancer-dominant |
| Tokelau | 1.818212037 | Low | 11.23177497 | Upper-middle | 1 | 3 | Stomach cancer-dominant |
| Tonga | 1.982411333 | Low | 17.08745508 | High | 1 | 4 | Stomach cancer-dominant |
| Trinidad and Tobago | 1.926426629 | Low | 5.262912071 | Low | 1 | 1 | Consistent |
| Tunisia | 0.666154107 | Low | 4.553141018 | Low | 1 | 1 | Consistent |
| Turkmenistan | 8.396298424 | High | 9.769678952 | Upper-middle | 4 | 3 | Esophageal cancer-dominant |
| Tuvalu | 2.110486767 | Lower-middle | 15.03180603 | High | 2 | 4 | Stomach cancer-dominant |
| Uganda | 14.80767177 | High | 6.116836785 | Low | 4 | 1 | Esophageal cancer-dominant |
| Ukraine | 2.45361839 | Lower-middle | 12.22071271 | Upper-middle | 2 | 3 | Stomach cancer-dominant |
| United Arab Emirates | 2.143848189 | Lower-middle | 7.820791025 | Lower-middle | 2 | 2 | Consistent |
| United Kingdom | 7.544198505 | High | 5.955788636 | Low | 4 | 1 | Esophageal cancer-dominant |
| United Republic of Tanzania | 11.35808648 | High | 5.918671977 | Low | 4 | 1 | Esophageal cancer-dominant |
| United States of America | 4.202698108 | Upper-middle | 5.067937486 | Low | 3 | 1 | Esophageal cancer-dominant |
| United States Virgin Islands | 2.324142603 | Lower-middle | 5.708484893 | Low | 2 | 1 | Esophageal cancer-dominant |
| Uruguay | 5.445460199 | High | 11.57168234 | Upper-middle | 4 | 3 | Esophageal cancer-dominant |
| Uzbekistan | 3.711295061 | Upper-middle | 7.793011623 | Lower-middle | 3 | 2 | Esophageal cancer-dominant |
| Vanuatu | 2.212082632 | Lower-middle | 15.99349569 | High | 2 | 4 | Stomach cancer-dominant |
| Venezuela (Bolivarian Republic of) | 1.646601643 | Low | 11.11431788 | Upper-middle | 1 | 3 | Stomach cancer-dominant |
| Viet Nam | 2.327388566 | Lower-middle | 8.644619326 | Lower-middle | 2 | 2 | Consistent |
| Yemen | 3.845028752 | Upper-middle | 18.69296925 | High | 3 | 4 | Stomach cancer-dominant |
| Zambia | 16.02215334 | High | 7.252192913 | Lower-middle | 4 | 2 | Esophageal cancer-dominant |
| Zimbabwe | 15.92519243 | High | 13.23425564 | High | 4 | 4 | Consistent |

Supplementary Table S5. cocurrence pattern of deaths

| location | val_EC | EC_level | val_SC | SC_level | EC_num | SC_num | region_type |
| --- | --- | --- | --- | --- | --- | --- | --- |
| Afghanistan | 7.774246475 | High | 34.56673556 | High | 4 | 4 | Consistent |
| Albania | 1.515097916 | Low | 10.35341621 | Upper-middle | 1 | 3 | Stomach cancer-dominant |
| Algeria | 0.752846387 | Low | 3.787033352 | Low | 1 | 1 | Consistent |
| American Samoa | 1.668251699 | Low | 17.63595586 | High | 1 | 4 | Stomach cancer-dominant |
| Andorra | 1.175918882 | Low | 6.801207447 | Lower-middle | 1 | 2 | Stomach cancer-dominant |
| Angola | 8.581071006 | High | 8.777823026 | Upper-middle | 4 | 3 | Esophageal cancer-dominant |
| Antigua and Barbuda | 2.992560458 | Lower-middle | 10.02689488 | Upper-middle | 2 | 3 | Stomach cancer-dominant |
| Argentina | 4.342552726 | Upper-middle | 8.386813803 | Lower-middle | 3 | 2 | Esophageal cancer-dominant |
| Armenia | 1.075329956 | Low | 10.78943922 | Upper-middle | 1 | 3 | Stomach cancer-dominant |
| Australia | 3.749961662 | Upper-middle | 3.779883473 | Low | 3 | 1 | Esophageal cancer-dominant |
| Austria | 2.307497145 | Lower-middle | 4.602266738 | Low | 2 | 1 | Esophageal cancer-dominant |
| Azerbaijan | 5.787059596 | High | 14.32745911 | High | 4 | 4 | Consistent |
| Bahamas | 5.3328323 | Upper-middle | 8.799953287 | Upper-middle | 3 | 3 | Consistent |
| Bahrain | 2.314808854 | Lower-middle | 6.789170665 | Lower-middle | 2 | 2 | Consistent |
| Bangladesh | 3.483709804 | Upper-middle | 5.436865979 | Low | 3 | 1 | Esophageal cancer-dominant |
| Barbados | 5.276477586 | Upper-middle | 9.765242752 | Upper-middle | 3 | 3 | Consistent |
| Belarus | 2.861370592 | Lower-middle | 12.38354529 | High | 2 | 4 | Stomach cancer-dominant |
| Belgium | 4.114385689 | Upper-middle | 4.464467432 | Low | 3 | 1 | Esophageal cancer-dominant |
| Belize | 2.242621478 | Lower-middle | 9.374562357 | Upper-middle | 2 | 3 | Stomach cancer-dominant |
| Benin | 5.406255064 | High | 9.700867519 | Upper-middle | 4 | 3 | Esophageal cancer-dominant |
| Bermuda | 4.056161085 | Upper-middle | 4.879199808 | Low | 3 | 1 | Esophageal cancer-dominant |
| Bhutan | 3.821091632 | Upper-middle | 5.632923028 | Low | 3 | 1 | Esophageal cancer-dominant |
| Bolivia (Plurinational State of) | 2.453467535 | Lower-middle | 32.40968557 | High | 2 | 4 | Stomach cancer-dominant |
| Bosnia and Herzegovina | 2.217993688 | Lower-middle | 8.67185605 | Upper-middle | 2 | 3 | Stomach cancer-dominant |
| Botswana | 9.22344571 | High | 5.744875923 | Low | 4 | 1 | Esophageal cancer-dominant |
| Brazil | 5.093468512 | Upper-middle | 9.807985427 | Upper-middle | 3 | 3 | Consistent |
| Brunei Darussalam | 2.145037596 | Low | 10.14366533 | Upper-middle | 1 | 3 | Stomach cancer-dominant |
| Bulgaria | 2.302332269 | Lower-middle | 11.29408705 | Upper-middle | 2 | 3 | Stomach cancer-dominant |
| Burkina Faso | 5.870208944 | High | 11.92426976 | High | 4 | 4 | Consistent |
| Burundi | 11.50127045 | High | 7.895601389 | Lower-middle | 4 | 2 | Esophageal cancer-dominant |
| Cabo Verde | 15.92694081 | High | 24.1166384 | High | 4 | 4 | Consistent |
| Cambodia | 3.121286532 | Lower-middle | 10.46136471 | Upper-middle | 2 | 3 | Stomach cancer-dominant |
| Cameroon | 6.709305297 | High | 10.37722276 | Upper-middle | 4 | 3 | Esophageal cancer-dominant |
| Canada | 3.552567344 | Upper-middle | 3.867920617 | Low | 3 | 1 | Esophageal cancer-dominant |
| Central African Republic | 11.45970776 | High | 13.16357938 | High | 4 | 4 | Consistent |
| Chad | 5.852209424 | High | 13.55360745 | High | 4 | 4 | Consistent |
| Chile | 3.184654613 | Upper-middle | 15.15127854 | High | 3 | 4 | Stomach cancer-dominant |
| China | 14.13107513 | High | 21.50936794 | High | 4 | 4 | Consistent |
| Colombia | 1.883743506 | Low | 13.51397958 | High | 1 | 4 | Stomach cancer-dominant |
| Comoros | 13.4665324 | High | 6.990496757 | Lower-middle | 4 | 2 | Esophageal cancer-dominant |
| Congo | 11.26411335 | High | 8.880036078 | Upper-middle | 4 | 3 | Esophageal cancer-dominant |
| Cook Islands | 2.544895065 | Lower-middle | 6.191798678 | Lower-middle | 2 | 2 | Consistent |
| Costa Rica | 1.631679527 | Low | 16.6217072 | High | 1 | 4 | Stomach cancer-dominant |
| C?te d'Ivoire | 1.393476239 | Low | 3.469144928 | Low | 1 | 1 | Consistent |
| Croatia | 2.577705879 | Lower-middle | 8.468219308 | Lower-middle | 2 | 2 | Consistent |
| Cuba | 4.953982906 | Upper-middle | 5.488277876 | Low | 3 | 1 | Esophageal cancer-dominant |
| Cyprus | 1.366783446 | Low | 5.891769059 | Low | 1 | 1 | Consistent |
| Czechia | 3.021838791 | Lower-middle | 5.457759664 | Low | 2 | 1 | Esophageal cancer-dominant |
| Democratic People's Republic of Korea | 9.132250987 | High | 21.56982837 | High | 4 | 4 | Consistent |
| Democratic Republic of the Congo | 8.577977739 | High | 8.803247097 | Upper-middle | 4 | 3 | Esophageal cancer-dominant |
| Denmark | 4.700743588 | Upper-middle | 4.602001091 | Low | 3 | 1 | Esophageal cancer-dominant |
| Djibouti | 13.05528218 | High | 7.564907283 | Lower-middle | 4 | 2 | Esophageal cancer-dominant |
| Dominica | 5.235624949 | Upper-middle | 20.28778037 | High | 3 | 4 | Stomach cancer-dominant |
| Dominican Republic | 2.299292035 | Lower-middle | 6.997976091 | Lower-middle | 2 | 2 | Consistent |
| Ecuador | 1.323566253 | Low | 18.64203717 | High | 1 | 4 | Stomach cancer-dominant |
| Egypt | 1.109602559 | Low | 7.205386158 | Lower-middle | 1 | 2 | Stomach cancer-dominant |
| El Salvador | 1.782320911 | Low | 17.61049304 | High | 1 | 4 | Stomach cancer-dominant |
| Equatorial Guinea | 9.422503836 | High | 6.58014037 | Lower-middle | 4 | 2 | Esophageal cancer-dominant |
| Eritrea | 15.59975515 | High | 10.15701576 | Upper-middle | 4 | 3 | Esophageal cancer-dominant |
| Estonia | 2.583982805 | Lower-middle | 9.852119463 | Upper-middle | 2 | 3 | Stomach cancer-dominant |
| Eswatini | 17.47194733 | High | 9.634197312 | Upper-middle | 4 | 3 | Esophageal cancer-dominant |
| Ethiopia | 5.384528827 | Upper-middle | 6.642024829 | Lower-middle | 3 | 2 | Esophageal cancer-dominant |
| Fiji | 2.860460377 | Lower-middle | 8.348775166 | Lower-middle | 2 | 2 | Consistent |
| Finland | 2.821290002 | Lower-middle | 4.167588103 | Low | 2 | 1 | Esophageal cancer-dominant |
| France | 3.652057386 | Upper-middle | 4.484844455 | Low | 3 | 1 | Esophageal cancer-dominant |
| Gabon | 11.19736891 | High | 7.925257661 | Lower-middle | 4 | 2 | Esophageal cancer-dominant |
| Gambia | 2.0662863 | Low | 3.53453163 | Low | 1 | 1 | Consistent |
| Georgia | 1.130198959 | Low | 11.93605693 | High | 1 | 4 | Stomach cancer-dominant |
| Germany | 3.686185813 | Upper-middle | 5.795044116 | Low | 3 | 1 | Esophageal cancer-dominant |
| Ghana | 3.795438849 | Upper-middle | 7.764961242 | Lower-middle | 3 | 2 | Esophageal cancer-dominant |
| Global | 6.25466261 | High | 11.19907152 | Upper-middle | 4 | 3 | Esophageal cancer-dominant |
| Greece | 1.663919852 | Low | 7.354086959 | Lower-middle | 1 | 2 | Stomach cancer-dominant |
| Greenland | 11.00234751 | High | 8.735487435 | Upper-middle | 4 | 3 | Esophageal cancer-dominant |
| Grenada | 4.844392498 | Upper-middle | 8.585557124 | Lower-middle | 3 | 2 | Esophageal cancer-dominant |
| Guam | 1.83860662 | Low | 4.918306689 | Low | 1 | 1 | Consistent |
| Guatemala | 1.81480894 | Low | 24.40388581 | High | 1 | 4 | Stomach cancer-dominant |
| Guinea | 1.441579951 | Low | 9.829903599 | Upper-middle | 1 | 3 | Stomach cancer-dominant |
| Guinea-Bissau | 7.801940433 | High | 16.10637105 | High | 4 | 4 | Consistent |
| Guyana | 2.145313356 | Lower-middle | 7.975003368 | Lower-middle | 2 | 2 | Consistent |
| Haiti | 4.258132783 | Upper-middle | 18.73236552 | High | 3 | 4 | Stomach cancer-dominant |
| Honduras | 1.603697034 | Low | 20.76238708 | High | 1 | 4 | Stomach cancer-dominant |
| Hungary | 3.185905494 | Upper-middle | 7.089075514 | Lower-middle | 3 | 2 | Esophageal cancer-dominant |
| Iceland | 3.846451859 | Upper-middle | 4.358635623 | Low | 3 | 1 | Esophageal cancer-dominant |
| India | 3.226665387 | Upper-middle | 5.83434002 | Low | 3 | 1 | Esophageal cancer-dominant |
| Indonesia | 1.713773949 | Low | 7.482936404 | Lower-middle | 1 | 2 | Stomach cancer-dominant |
| Iran (Islamic Republic of) | 3.989002411 | Upper-middle | 13.25904263 | High | 3 | 4 | Stomach cancer-dominant |
| Iraq | 1.230087522 | Low | 5.266953724 | Low | 1 | 1 | Consistent |
| Ireland | 5.391489581 | Upper-middle | 4.615617591 | Low | 3 | 1 | Esophageal cancer-dominant |
| Israel | 1.465363151 | Low | 5.147579141 | Low | 1 | 1 | Consistent |
| Italy | 1.607844042 | Low | 7.273190509 | Lower-middle | 1 | 2 | Stomach cancer-dominant |
| Jamaica | 3.126573128 | Lower-middle | 9.122691802 | Upper-middle | 2 | 3 | Stomach cancer-dominant |
| Japan | 3.814807595 | Upper-middle | 13.19707014 | High | 3 | 4 | Stomach cancer-dominant |
| Jordan | 0.882458055 | Low | 3.875511609 | Low | 1 | 1 | Consistent |
| Kazakhstan | 5.02081446 | Upper-middle | 10.79589436 | Upper-middle | 3 | 3 | Consistent |
| Kenya | 12.68506484 | High | 8.240687996 | Lower-middle | 4 | 2 | Esophageal cancer-dominant |
| Kiribati | 5.962105819 | High | 22.05953397 | High | 4 | 4 | Consistent |
| Kuwait | 0.946425424 | Low | 2.586891968 | Low | 1 | 1 | Consistent |
| Kyrgyzstan | 3.028956749 | Lower-middle | 15.30130914 | High | 2 | 4 | Stomach cancer-dominant |
| Lao People's Democratic Republic | 2.635812163 | Lower-middle | 8.523812963 | Lower-middle | 2 | 2 | Consistent |
| Latvia | 2.900486157 | Lower-middle | 10.73520444 | Upper-middle | 2 | 3 | Stomach cancer-dominant |
| Lebanon | 0.935169174 | Low | 5.392106051 | Low | 1 | 1 | Consistent |
| Lesotho | 16.666314 | High | 12.5136562 | High | 4 | 4 | Consistent |
| Liberia | 6.068285892 | High | 10.19094275 | Upper-middle | 4 | 3 | Esophageal cancer-dominant |
| Libya | 1.761053538 | Low | 6.620200602 | Lower-middle | 1 | 2 | Stomach cancer-dominant |
| Lithuania | 3.55944657 | Upper-middle | 10.62531181 | Upper-middle | 3 | 3 | Consistent |
| Luxembourg | 3.3536334 | Upper-middle | 4.455966998 | Low | 3 | 1 | Esophageal cancer-dominant |
| Madagascar | 11.59428809 | High | 6.459695467 | Lower-middle | 4 | 2 | Esophageal cancer-dominant |
| Malawi | 27.77425789 | High | 3.241401436 | Low | 4 | 1 | Esophageal cancer-dominant |
| Malaysia | 2.685010236 | Lower-middle | 6.343004626 | Lower-middle | 2 | 2 | Consistent |
| Maldives | 1.220636804 | Low | 2.801182784 | Low | 1 | 1 | Consistent |
| Mali | 2.632493429 | Lower-middle | 17.07669425 | High | 2 | 4 | Stomach cancer-dominant |
| Malta | 2.172490024 | Lower-middle | 4.690451163 | Low | 2 | 1 | Esophageal cancer-dominant |
| Marshall Islands | 2.667260216 | Lower-middle | 17.92799728 | High | 2 | 4 | Stomach cancer-dominant |
| Mauritania | 5.62151664 | High | 9.105590105 | Upper-middle | 4 | 3 | Esophageal cancer-dominant |
| Mauritius | 3.505800559 | Upper-middle | 9.973369285 | Upper-middle | 3 | 3 | Consistent |
| Mexico | 1.530224627 | Low | 9.238400116 | Upper-middle | 1 | 3 | Stomach cancer-dominant |
| Micronesia (Federated States of) | 2.849447149 | Lower-middle | 17.17211883 | High | 2 | 4 | Stomach cancer-dominant |
| Monaco | 5.480850134 | High | 7.104705297 | Lower-middle | 4 | 2 | Esophageal cancer-dominant |
| Mongolia | 17.98448619 | High | 37.39990378 | High | 4 | 4 | Consistent |
| Montenegro | 2.288148021 | Lower-middle | 7.897419895 | Lower-middle | 2 | 2 | Consistent |
| Morocco | 0.900136707 | Low | 2.510769819 | Low | 1 | 1 | Consistent |
| Mozambique | 9.79092866 | High | 8.120378048 | Lower-middle | 4 | 2 | Esophageal cancer-dominant |
| Myanmar | 2.375199265 | Lower-middle | 7.380316846 | Lower-middle | 2 | 2 | Consistent |
| Namibia | 2.463564599 | Lower-middle | 2.740996699 | Low | 2 | 1 | Esophageal cancer-dominant |
| Nauru | 3.438164626 | Upper-middle | 20.85282221 | High | 3 | 4 | Stomach cancer-dominant |
| Nepal | 4.041038111 | Upper-middle | 6.242534019 | Lower-middle | 3 | 2 | Esophageal cancer-dominant |
| Netherlands | 6.2122717 | High | 4.622511594 | Low | 4 | 1 | Esophageal cancer-dominant |
| New Zealand | 3.328093368 | Upper-middle | 4.586454284 | Low | 3 | 1 | Esophageal cancer-dominant |
| Nicaragua | 0.886095431 | Low | 9.776315736 | Upper-middle | 1 | 3 | Stomach cancer-dominant |
| Niger | 4.475167897 | Upper-middle | 11.56232318 | Upper-middle | 3 | 3 | Consistent |
| Nigeria | 4.643411865 | Upper-middle | 2.627395662 | Low | 3 | 1 | Esophageal cancer-dominant |
| Niue | 2.543319977 | Lower-middle | 11.62409015 | High | 2 | 4 | Stomach cancer-dominant |
| North Macedonia | 1.459409746 | Low | 14.06836211 | High | 1 | 4 | Stomach cancer-dominant |
| Northern Mariana Islands | 2.71420479 | Lower-middle | 13.65425799 | High | 2 | 4 | Stomach cancer-dominant |
| Norway | 2.915942766 | Lower-middle | 3.800858016 | Low | 2 | 1 | Esophageal cancer-dominant |
| Oman | 1.624573544 | Low | 5.330610087 | Low | 1 | 1 | Consistent |
| Pakistan | 6.703143945 | High | 4.851729233 | Low | 4 | 1 | Esophageal cancer-dominant |
| Palau | 3.128366757 | Lower-middle | 18.94560386 | High | 2 | 4 | Stomach cancer-dominant |
| Palestine | 0.964647344 | Low | 6.37845795 | Lower-middle | 1 | 2 | Stomach cancer-dominant |
| Panama | 1.284135123 | Low | 10.02973212 | Upper-middle | 1 | 3 | Stomach cancer-dominant |
| Papua New Guinea | 1.715768631 | Low | 14.1868387 | High | 1 | 4 | Stomach cancer-dominant |
| Paraguay | 4.21971995 | Upper-middle | 8.725722002 | Upper-middle | 3 | 3 | Consistent |
| Peru | 1.362437446 | Low | 19.94880819 | High | 1 | 4 | Stomach cancer-dominant |
| Philippines | 1.390866845 | Low | 4.378704818 | Low | 1 | 1 | Consistent |
| Poland | 3.136053119 | Upper-middle | 8.420949379 | Lower-middle | 3 | 2 | Esophageal cancer-dominant |
| Portugal | 3.238540529 | Upper-middle | 10.75149286 | Upper-middle | 3 | 3 | Consistent |
| Puerto Rico | 2.385077818 | Lower-middle | 4.241386546 | Low | 2 | 1 | Esophageal cancer-dominant |
| Qatar | 2.645041911 | Lower-middle | 5.816981126 | Low | 2 | 1 | Esophageal cancer-dominant |
| Republic of Korea | 2.288805106 | Lower-middle | 13.25727799 | High | 2 | 4 | Stomach cancer-dominant |
| Republic of Moldova | 1.837791152 | Low | 8.376549854 | Lower-middle | 1 | 2 | Stomach cancer-dominant |
| Romania | 2.544912263 | Lower-middle | 10.36704487 | Upper-middle | 2 | 3 | Stomach cancer-dominant |
| Russian Federation | 3.215395627 | Upper-middle | 12.99850106 | High | 3 | 4 | Stomach cancer-dominant |
| Rwanda | 12.21794843 | High | 6.911244968 | Lower-middle | 4 | 2 | Esophageal cancer-dominant |
| Saint Kitts and Nevis | 4.026800548 | Upper-middle | 9.145846081 | Upper-middle | 3 | 3 | Consistent |
| Saint Lucia | 4.379684136 | Upper-middle | 10.91325349 | Upper-middle | 3 | 3 | Consistent |
| Saint Vincent and the Grenadines | 2.617897403 | Lower-middle | 10.4350028 | Upper-middle | 2 | 3 | Stomach cancer-dominant |
| Samoa | 1.166217482 | Low | 10.55994591 | Upper-middle | 1 | 3 | Stomach cancer-dominant |
| San Marino | 0.847285104 | Low | 9.529087881 | Upper-middle | 1 | 3 | Stomach cancer-dominant |
| Sao Tome and Principe | 4.934914677 | Upper-middle | 15.43833748 | High | 3 | 4 | Stomach cancer-dominant |
| Saudi Arabia | 1.807741931 | Low | 3.606340193 | Low | 1 | 1 | Consistent |
| Senegal | 5.460953439 | High | 10.79519332 | Upper-middle | 4 | 3 | Esophageal cancer-dominant |
| Serbia | 2.167844724 | Lower-middle | 7.718091337 | Lower-middle | 2 | 2 | Consistent |
| Seychelles | 4.767241116 | Upper-middle | 6.030562173 | Lower-middle | 3 | 2 | Esophageal cancer-dominant |
| Sierra Leone | 5.187946134 | Upper-middle | 10.44954402 | Upper-middle | 3 | 3 | Consistent |
| Singapore | 1.706077545 | Low | 4.534059197 | Low | 1 | 1 | Consistent |
| Slovakia | 3.212231603 | Upper-middle | 8.310332796 | Lower-middle | 3 | 2 | Esophageal cancer-dominant |
| Slovenia | 2.20591495 | Lower-middle | 6.783234545 | Lower-middle | 2 | 2 | Consistent |
| Solomon Islands | 2.578597573 | Lower-middle | 19.48202786 | High | 2 | 4 | Stomach cancer-dominant |
| Somalia | 15.96524808 | High | 11.93978966 | High | 4 | 4 | Consistent |
| South Africa | 11.06064176 | High | 6.19956548 | Lower-middle | 4 | 2 | Esophageal cancer-dominant |
| South Sudan | 16.20991969 | High | 9.530637361 | Upper-middle | 4 | 3 | Esophageal cancer-dominant |
| Spain | 2.404918519 | Lower-middle | 6.051907003 | Lower-middle | 2 | 2 | Consistent |
| Sri Lanka | 4.160290927 | Upper-middle | 3.572608694 | Low | 3 | 1 | Esophageal cancer-dominant |
| Sudan | 4.561032974 | Upper-middle | 15.18820129 | High | 3 | 4 | Stomach cancer-dominant |
| Suriname | 1.687759623 | Low | 7.066050546 | Lower-middle | 1 | 2 | Stomach cancer-dominant |
| Sweden | 2.624870787 | Lower-middle | 3.376410836 | Low | 2 | 1 | Esophageal cancer-dominant |
| Switzerland | 2.839544393 | Lower-middle | 3.732800416 | Low | 2 | 1 | Esophageal cancer-dominant |
| Syrian Arab Republic | 0.950988143 | Low | 6.126173349 | Lower-middle | 1 | 2 | Stomach cancer-dominant |
| Taiwan (Province of China) | 7.454902572 | High | 8.86021045 | Upper-middle | 4 | 3 | Esophageal cancer-dominant |
| Tajikistan | 6.768946257 | High | 14.56557213 | High | 4 | 4 | Consistent |
| Thailand | 4.436570127 | Upper-middle | 7.27474294 | Lower-middle | 3 | 2 | Esophageal cancer-dominant |
| Timor-Leste | 2.230765104 | Lower-middle | 7.731737106 | Lower-middle | 2 | 2 | Consistent |
| Togo | 6.223589796 | High | 11.77752795 | High | 4 | 4 | Consistent |
| Tokelau | 1.937733149 | Low | 10.96528776 | Upper-middle | 1 | 3 | Stomach cancer-dominant |
| Tonga | 2.115636008 | Low | 16.74514643 | High | 1 | 4 | Stomach cancer-dominant |
| Trinidad and Tobago | 2.00600165 | Low | 5.17401881 | Low | 1 | 1 | Consistent |
| Tunisia | 0.686753098 | Low | 4.302468476 | Low | 1 | 1 | Consistent |
| Turkmenistan | 8.978267975 | High | 9.58681809 | Upper-middle | 4 | 3 | Esophageal cancer-dominant |
| Tuvalu | 2.259651564 | Lower-middle | 15.04317379 | High | 2 | 4 | Stomach cancer-dominant |
| Uganda | 15.90130704 | High | 6.364771008 | Lower-middle | 4 | 2 | Esophageal cancer-dominant |
| Ukraine | 2.212710555 | Lower-middle | 10.21624378 | Upper-middle | 2 | 3 | Stomach cancer-dominant |
| United Arab Emirates | 2.359204549 | Lower-middle | 8.056420907 | Lower-middle | 2 | 2 | Consistent |
| United Kingdom | 7.160028241 | High | 4.606311097 | Low | 4 | 1 | Esophageal cancer-dominant |
| United Republic of Tanzania | 12.24104926 | High | 6.160796531 | Lower-middle | 4 | 2 | Esophageal cancer-dominant |
| United States of America | 3.620098544 | Upper-middle | 2.835990713 | Low | 3 | 1 | Esophageal cancer-dominant |
| United States Virgin Islands | 2.423999836 | Lower-middle | 5.544949861 | Low | 2 | 1 | Esophageal cancer-dominant |
| Uruguay | 5.642282573 | High | 10.7238827 | Upper-middle | 4 | 3 | Esophageal cancer-dominant |
| Uzbekistan | 3.977042281 | Upper-middle | 7.661890929 | Lower-middle | 3 | 2 | Esophageal cancer-dominant |
| Vanuatu | 2.390246218 | Lower-middle | 16.30525091 | High | 2 | 4 | Stomach cancer-dominant |
| Venezuela (Bolivarian Republic of) | 1.754757179 | Low | 10.90165661 | Upper-middle | 1 | 3 | Stomach cancer-dominant |
| Viet Nam | 2.298188945 | Lower-middle | 7.691182761 | Lower-middle | 2 | 2 | Consistent |
| Yemen | 4.156051223 | Upper-middle | 19.38493618 | High | 3 | 4 | Stomach cancer-dominant |
| Zambia | 17.12664728 | High | 7.513553664 | Lower-middle | 4 | 2 | Esophageal cancer-dominant |
| Zimbabwe | 17.02113486 | High | 13.76276301 | High | 4 | 4 | Consistent |

Supplementary Table S6. cocurrence pattern of dalys

| location | val_EC | EC_level | val_SC | SC_level | EC_num | SC_num | region_type |
| --- | --- | --- | --- | --- | --- | --- | --- |
| Afghanistan | 197.4201805 | High | 910.2443137 | High | 4 | 4 | Consistent |
| Albania | 35.34257603 | Low | 225.8773693 | Upper-middle | 1 | 3 | Stomach cancer-dominant |
| Algeria | 16.36138334 | Low | 81.57996903 | Low | 1 | 1 | Consistent |
| American Samoa | 39.92405155 | Low | 433.9901742 | High | 1 | 4 | Stomach cancer-dominant |
| Andorra | 28.59839458 | Low | 140.9931545 | Lower-middle | 1 | 2 | Stomach cancer-dominant |
| Angola | 212.845753 | High | 214.8638741 | Upper-middle | 4 | 3 | Esophageal cancer-dominant |
| Antigua and Barbuda | 68.16853177 | Lower-middle | 216.344508 | Upper-middle | 2 | 3 | Stomach cancer-dominant |
| Argentina | 97.43195349 | Upper-middle | 197.5014394 | Lower-middle | 3 | 2 | Esophageal cancer-dominant |
| Armenia | 24.94386408 | Low | 259.379373 | Upper-middle | 1 | 3 | Stomach cancer-dominant |
| Australia | 82.26842463 | Upper-middle | 81.32799147 | Low | 3 | 1 | Esophageal cancer-dominant |
| Austria | 55.3895411 | Lower-middle | 100.7132157 | Low | 2 | 1 | Esophageal cancer-dominant |
| Azerbaijan | 135.6540393 | High | 350.1267937 | High | 4 | 4 | Consistent |
| Bahamas | 139.8427925 | High | 213.720592 | Upper-middle | 4 | 3 | Esophageal cancer-dominant |
| Bahrain | 45.60965173 | Low | 132.8501204 | Low | 1 | 1 | Consistent |
| Bangladesh | 86.65728084 | Upper-middle | 133.0397697 | Low | 3 | 1 | Esophageal cancer-dominant |
| Barbados | 123.5598754 | Upper-middle | 213.6677778 | Upper-middle | 3 | 3 | Consistent |
| Belarus | 81.52034906 | Upper-middle | 324.5677912 | High | 3 | 4 | Stomach cancer-dominant |
| Belgium | 97.69660993 | Upper-middle | 95.62204673 | Low | 3 | 1 | Esophageal cancer-dominant |
| Belize | 56.64282428 | Lower-middle | 225.0078195 | Upper-middle | 2 | 3 | Stomach cancer-dominant |
| Benin | 132.8687441 | High | 222.7215665 | Upper-middle | 4 | 3 | Esophageal cancer-dominant |
| Bermuda | 95.35457461 | Upper-middle | 104.5545469 | Low | 3 | 1 | Esophageal cancer-dominant |
| Bhutan | 91.87210679 | Upper-middle | 134.1094679 | Low | 3 | 1 | Esophageal cancer-dominant |
| Bolivia (Plurinational State of) | 52.85068893 | Lower-middle | 714.4307159 | High | 2 | 4 | Stomach cancer-dominant |
| Bosnia and Herzegovina | 56.56814782 | Lower-middle | 199.6619217 | Lower-middle | 2 | 2 | Consistent |
| Botswana | 226.6017198 | High | 133.7662161 | Low | 4 | 1 | Esophageal cancer-dominant |
| Brazil | 132.7800769 | High | 238.7664979 | Upper-middle | 4 | 3 | Esophageal cancer-dominant |
| Brunei Darussalam | 50.18525621 | Low | 236.1499184 | Upper-middle | 1 | 3 | Stomach cancer-dominant |
| Bulgaria | 62.87818846 | Lower-middle | 274.3718347 | Upper-middle | 2 | 3 | Stomach cancer-dominant |
| Burkina Faso | 143.9376035 | High | 275.6691052 | Upper-middle | 4 | 3 | Esophageal cancer-dominant |
| Burundi | 285.0324027 | High | 195.9458661 | Lower-middle | 4 | 2 | Esophageal cancer-dominant |
| Cabo Verde | 398.2922392 | High | 511.1962759 | High | 4 | 4 | Consistent |
| Cambodia | 77.76351292 | Lower-middle | 257.3741558 | Upper-middle | 2 | 3 | Stomach cancer-dominant |
| Cameroon | 169.4496785 | High | 242.8245098 | Upper-middle | 4 | 3 | Esophageal cancer-dominant |
| Canada | 82.2657631 | Upper-middle | 84.42655205 | Low | 3 | 1 | Esophageal cancer-dominant |
| Central African Republic | 299.9139359 | High | 343.7843713 | High | 4 | 4 | Consistent |
| Chad | 146.1679972 | High | 322.3884805 | High | 4 | 4 | Consistent |
| Chile | 63.98701441 | Lower-middle | 335.4258522 | High | 2 | 4 | Stomach cancer-dominant |
| China | 317.1824119 | High | 501.2599032 | High | 4 | 4 | Consistent |
| Colombia | 40.93045897 | Low | 330.4221227 | High | 1 | 4 | Stomach cancer-dominant |
| Comoros | 329.862383 | High | 170.6420839 | Lower-middle | 4 | 2 | Esophageal cancer-dominant |
| Congo | 281.5012947 | High | 218.0998361 | Upper-middle | 4 | 3 | Esophageal cancer-dominant |
| Cook Islands | 59.74269758 | Lower-middle | 146.5540559 | Lower-middle | 2 | 2 | Consistent |
| Costa Rica | 36.83210624 | Low | 395.2611988 | High | 1 | 4 | Stomach cancer-dominant |
| C?te d'Ivoire | 34.69644887 | Low | 83.2455414 | Low | 1 | 1 | Consistent |
| Croatia | 66.80290982 | Lower-middle | 186.7673352 | Lower-middle | 2 | 2 | Consistent |
| Cuba | 128.6119375 | Upper-middle | 126.8543073 | Low | 3 | 1 | Esophageal cancer-dominant |
| Cyprus | 29.7679676 | Low | 111.9483341 | Low | 1 | 1 | Consistent |
| Czechia | 77.30808907 | Lower-middle | 122.3516357 | Low | 2 | 1 | Esophageal cancer-dominant |
| Democratic People's Republic of Korea | 241.0815797 | High | 585.7494508 | High | 4 | 4 | Consistent |
| Democratic Republic of the Congo | 213.239906 | High | 216.0574162 | Upper-middle | 4 | 3 | Esophageal cancer-dominant |
| Denmark | 106.7753258 | Upper-middle | 100.2208075 | Low | 3 | 1 | Esophageal cancer-dominant |
| Djibouti | 320.5646948 | High | 182.7038838 | Lower-middle | 4 | 2 | Esophageal cancer-dominant |
| Dominica | 127.8129732 | Upper-middle | 453.2882816 | High | 3 | 4 | Stomach cancer-dominant |
| Dominican Republic | 58.46458222 | Lower-middle | 171.8550167 | Lower-middle | 2 | 2 | Consistent |
| Ecuador | 27.39383442 | Low | 422.6671634 | High | 1 | 4 | Stomach cancer-dominant |
| Egypt | 25.3295267 | Low | 168.3124074 | Lower-middle | 1 | 2 | Stomach cancer-dominant |
| El Salvador | 42.49884235 | Low | 432.3574904 | High | 1 | 4 | Stomach cancer-dominant |
| Equatorial Guinea | 229.6853335 | High | 156.1657964 | Lower-middle | 4 | 2 | Esophageal cancer-dominant |
| Eritrea | 391.0267288 | High | 258.2821898 | Upper-middle | 4 | 3 | Esophageal cancer-dominant |
| Estonia | 65.91940964 | Lower-middle | 230.0217495 | Upper-middle | 2 | 3 | Stomach cancer-dominant |
| Eswatini | 478.8525283 | High | 248.3498311 | Upper-middle | 4 | 3 | Esophageal cancer-dominant |
| Ethiopia | 129.5996055 | Upper-middle | 164.7770689 | Lower-middle | 3 | 2 | Esophageal cancer-dominant |
| Fiji | 66.24287824 | Lower-middle | 196.61568 | Lower-middle | 2 | 2 | Consistent |
| Finland | 66.1789487 | Lower-middle | 89.04413066 | Low | 2 | 1 | Esophageal cancer-dominant |
| France | 88.6376433 | Upper-middle | 100.9570731 | Low | 3 | 1 | Esophageal cancer-dominant |
| Gabon | 277.423906 | High | 189.5095 | Lower-middle | 4 | 2 | Esophageal cancer-dominant |
| Gambia | 51.5376899 | Lower-middle | 86.26193098 | Low | 2 | 1 | Esophageal cancer-dominant |
| Georgia | 27.43277869 | Low | 294.2692057 | High | 1 | 4 | Stomach cancer-dominant |
| Germany | 90.65545234 | Upper-middle | 131.1632027 | Low | 3 | 1 | Esophageal cancer-dominant |
| Ghana | 90.51764374 | Upper-middle | 175.3798881 | Lower-middle | 3 | 2 | Esophageal cancer-dominant |
| Global | 148.5612569 | High | 262.7480841 | Upper-middle | 4 | 3 | Esophageal cancer-dominant |
| Greece | 40.61993399 | Low | 160.7189223 | Lower-middle | 1 | 2 | Stomach cancer-dominant |
| Greenland | 272.770181 | High | 216.8896076 | Upper-middle | 4 | 3 | Esophageal cancer-dominant |
| Grenada | 122.7751793 | Upper-middle | 194.9904154 | Lower-middle | 3 | 2 | Esophageal cancer-dominant |
| Guam | 51.70482219 | Lower-middle | 144.7300008 | Lower-middle | 2 | 2 | Consistent |
| Guatemala | 40.64783476 | Low | 581.4308919 | High | 1 | 4 | Stomach cancer-dominant |
| Guinea | 36.04245821 | Low | 242.5136654 | Upper-middle | 1 | 3 | Stomach cancer-dominant |
| Guinea-Bissau | 201.1284183 | High | 395.2834482 | High | 4 | 4 | Consistent |
| Guyana | 57.17469677 | Lower-middle | 201.1459373 | Upper-middle | 2 | 3 | Stomach cancer-dominant |
| Haiti | 106.3101229 | Upper-middle | 453.1429917 | High | 3 | 4 | Stomach cancer-dominant |
| Honduras | 35.28966345 | Low | 462.6305693 | High | 1 | 4 | Stomach cancer-dominant |
| Hungary | 87.02524868 | Upper-middle | 169.1432966 | Lower-middle | 3 | 2 | Esophageal cancer-dominant |
| Iceland | 89.54855832 | Upper-middle | 90.15922824 | Low | 3 | 1 | Esophageal cancer-dominant |
| India | 83.40469627 | Upper-middle | 149.276115 | Lower-middle | 3 | 2 | Esophageal cancer-dominant |
| Indonesia | 41.43552404 | Low | 178.9403634 | Lower-middle | 1 | 2 | Stomach cancer-dominant |
| Iran (Islamic Republic of) | 86.70591404 | Upper-middle | 292.5939425 | High | 3 | 4 | Stomach cancer-dominant |
| Iraq | 29.08822438 | Low | 126.7123993 | Low | 1 | 1 | Consistent |
| Ireland | 120.1538826 | Upper-middle | 94.43679485 | Low | 3 | 1 | Esophageal cancer-dominant |
| Israel | 32.4307547 | Low | 108.3614945 | Low | 1 | 1 | Consistent |
| Italy | 37.13119372 | Low | 151.8191861 | Lower-middle | 1 | 2 | Stomach cancer-dominant |
| Jamaica | 75.24686012 | Lower-middle | 211.0593936 | Upper-middle | 2 | 3 | Stomach cancer-dominant |
| Japan | 84.34341986 | Upper-middle | 270.1724303 | Upper-middle | 3 | 3 | Consistent |
| Jordan | 20.16584493 | Low | 87.57421345 | Low | 1 | 1 | Consistent |
| Kazakhstan | 119.3358389 | Upper-middle | 279.9921711 | High | 3 | 4 | Stomach cancer-dominant |
| Kenya | 306.1183943 | High | 200.2566327 | Lower-middle | 4 | 2 | Esophageal cancer-dominant |
| Kiribati | 150.7193545 | High | 575.9626773 | High | 4 | 4 | Consistent |
| Kuwait | 19.70430275 | Low | 54.86524988 | Low | 1 | 1 | Consistent |
| Kyrgyzstan | 72.50679429 | Lower-middle | 407.9296749 | High | 2 | 4 | Stomach cancer-dominant |
| Lao People's Democratic Republic | 66.11497832 | Lower-middle | 211.2091161 | Upper-middle | 2 | 3 | Stomach cancer-dominant |
| Latvia | 78.32975289 | Upper-middle | 267.4925552 | Upper-middle | 3 | 3 | Consistent |
| Lebanon | 20.85306349 | Low | 118.7286763 | Low | 1 | 1 | Consistent |
| Lesotho | 450.0111153 | High | 323.791545 | High | 4 | 4 | Consistent |
| Liberia | 150.6063699 | High | 237.2483216 | Upper-middle | 4 | 3 | Esophageal cancer-dominant |
| Libya | 43.78903178 | Low | 160.1279303 | Lower-middle | 1 | 2 | Stomach cancer-dominant |
| Lithuania | 97.27583241 | Upper-middle | 265.6819963 | Upper-middle | 3 | 3 | Consistent |
| Luxembourg | 77.99516398 | Upper-middle | 88.32975514 | Low | 3 | 1 | Esophageal cancer-dominant |
| Madagascar | 291.3389373 | High | 164.1519221 | Lower-middle | 4 | 2 | Esophageal cancer-dominant |
| Malawi | 715.28158 | High | 81.80208951 | Low | 4 | 1 | Esophageal cancer-dominant |
| Malaysia | 64.89543216 | Lower-middle | 148.904216 | Lower-middle | 2 | 2 | Consistent |
| Maldives | 27.82547893 | Low | 61.2927278 | Low | 1 | 1 | Consistent |
| Mali | 65.0746487 | Lower-middle | 420.3184602 | High | 2 | 4 | Stomach cancer-dominant |
| Malta | 52.06379483 | Lower-middle | 99.5832048 | Low | 2 | 1 | Esophageal cancer-dominant |
| Marshall Islands | 66.77706953 | Lower-middle | 470.291568 | High | 2 | 4 | Stomach cancer-dominant |
| Mauritania | 132.9327274 | High | 201.8207991 | Upper-middle | 4 | 3 | Esophageal cancer-dominant |
| Mauritius | 92.68101177 | Upper-middle | 252.4040868 | Upper-middle | 3 | 3 | Consistent |
| Mexico | 36.04521469 | Low | 223.4684626 | Upper-middle | 1 | 3 | Stomach cancer-dominant |
| Micronesia (Federated States of) | 72.06078151 | Lower-middle | 444.3056795 | High | 2 | 4 | Stomach cancer-dominant |
| Monaco | 127.0255975 | Upper-middle | 153.1090829 | Lower-middle | 3 | 2 | Esophageal cancer-dominant |
| Mongolia | 397.9783307 | High | 930.4485401 | High | 4 | 4 | Consistent |
| Montenegro | 57.88240337 | Lower-middle | 170.0697008 | Lower-middle | 2 | 2 | Consistent |
| Morocco | 21.3628901 | Low | 60.06101773 | Low | 1 | 1 | Consistent |
| Mozambique | 232.6216104 | High | 185.060416 | Lower-middle | 4 | 2 | Esophageal cancer-dominant |
| Myanmar | 59.50978645 | Lower-middle | 182.5440033 | Lower-middle | 2 | 2 | Consistent |
| Namibia | 65.51323519 | Lower-middle | 69.40981657 | Low | 2 | 1 | Esophageal cancer-dominant |
| Nauru | 89.21893199 | Upper-middle | 557.0266948 | High | 3 | 4 | Stomach cancer-dominant |
| Nepal | 100.1879655 | Upper-middle | 153.4195177 | Lower-middle | 3 | 2 | Esophageal cancer-dominant |
| Netherlands | 137.0783714 | High | 94.73729082 | Low | 4 | 1 | Esophageal cancer-dominant |
| New Zealand | 69.67272343 | Lower-middle | 103.2000069 | Low | 2 | 1 | Esophageal cancer-dominant |
| Nicaragua | 20.4463061 | Low | 235.4039665 | Upper-middle | 1 | 3 | Stomach cancer-dominant |
| Niger | 107.2706831 | Upper-middle | 264.3093156 | Upper-middle | 3 | 3 | Consistent |
| Nigeria | 108.9169284 | Upper-middle | 60.62126555 | Low | 3 | 1 | Esophageal cancer-dominant |
| Niue | 60.59460599 | Lower-middle | 279.5340576 | High | 2 | 4 | Stomach cancer-dominant |
| North Macedonia | 35.28179614 | Low | 301.7364094 | High | 1 | 4 | Stomach cancer-dominant |
| Northern Mariana Islands | 62.76041411 | Lower-middle | 314.4157082 | High | 2 | 4 | Stomach cancer-dominant |
| Norway | 64.08768072 | Lower-middle | 77.01174818 | Low | 2 | 1 | Esophageal cancer-dominant |
| Oman | 36.96489457 | Low | 115.7369457 | Low | 1 | 1 | Consistent |
| Pakistan | 167.9996514 | High | 122.0196066 | Low | 4 | 1 | Esophageal cancer-dominant |
| Palau | 71.1347766 | Lower-middle | 472.6077109 | High | 2 | 4 | Stomach cancer-dominant |
| Palestine | 20.81763067 | Low | 139.1017006 | Lower-middle | 1 | 2 | Stomach cancer-dominant |
| Panama | 28.40437642 | Low | 234.6448203 | Upper-middle | 1 | 3 | Stomach cancer-dominant |
| Papua New Guinea | 41.06846244 | Low | 354.1746826 | High | 1 | 4 | Stomach cancer-dominant |
| Paraguay | 104.666169 | Upper-middle | 201.1657663 | Upper-middle | 3 | 3 | Consistent |
| Peru | 29.096363 | Low | 455.2952568 | High | 1 | 4 | Stomach cancer-dominant |
| Philippines | 36.36618412 | Low | 111.9983032 | Low | 1 | 1 | Consistent |
| Poland | 80.97308197 | Upper-middle | 196.8309888 | Lower-middle | 3 | 2 | Esophageal cancer-dominant |
| Portugal | 85.41056384 | Upper-middle | 242.9862905 | Upper-middle | 3 | 3 | Consistent |
| Puerto Rico | 59.4981081 | Lower-middle | 98.99548993 | Low | 2 | 1 | Esophageal cancer-dominant |
| Qatar | 52.59746422 | Lower-middle | 115.0265833 | Low | 2 | 1 | Esophageal cancer-dominant |
| Republic of Korea | 50.14878945 | Low | 288.8217455 | High | 1 | 4 | Stomach cancer-dominant |
| Republic of Moldova | 50.03624017 | Low | 222.4629825 | Upper-middle | 1 | 3 | Stomach cancer-dominant |
| Romania | 72.11870763 | Lower-middle | 256.88455 | Upper-middle | 2 | 3 | Stomach cancer-dominant |
| Russian Federation | 86.82855149 | Upper-middle | 318.7594662 | High | 3 | 4 | Stomach cancer-dominant |
| Rwanda | 295.3160975 | High | 167.117339 | Lower-middle | 4 | 2 | Esophageal cancer-dominant |
| Saint Kitts and Nevis | 97.61591 | Upper-middle | 199.0526547 | Lower-middle | 3 | 2 | Esophageal cancer-dominant |
| Saint Lucia | 108.8435586 | Upper-middle | 247.120086 | Upper-middle | 3 | 3 | Consistent |
| Saint Vincent and the Grenadines | 66.24468578 | Lower-middle | 243.2269508 | Upper-middle | 2 | 3 | Stomach cancer-dominant |
| Samoa | 28.91413389 | Low | 263.5499912 | Upper-middle | 1 | 3 | Stomach cancer-dominant |
| San Marino | 20.32101776 | Low | 198.3050666 | Lower-middle | 1 | 2 | Stomach cancer-dominant |
| Sao Tome and Principe | 120.4150548 | Upper-middle | 331.5469493 | High | 3 | 4 | Stomach cancer-dominant |
| Saudi Arabia | 41.6316087 | Low | 85.57795744 | Low | 1 | 1 | Consistent |
| Senegal | 132.9474208 | High | 246.8755373 | Upper-middle | 4 | 3 | Esophageal cancer-dominant |
| Serbia | 56.20668389 | Lower-middle | 179.9092146 | Lower-middle | 2 | 2 | Consistent |
| Seychelles | 124.3506883 | Upper-middle | 148.4897626 | Lower-middle | 3 | 2 | Esophageal cancer-dominant |
| Sierra Leone | 128.6868017 | Upper-middle | 245.2537588 | Upper-middle | 3 | 3 | Consistent |
| Singapore | 37.68450008 | Low | 92.39032641 | Low | 1 | 1 | Consistent |
| Slovakia | 87.87622704 | Upper-middle | 190.7362856 | Lower-middle | 3 | 2 | Esophageal cancer-dominant |
| Slovenia | 55.02650306 | Lower-middle | 143.9453207 | Lower-middle | 2 | 2 | Consistent |
| Solomon Islands | 65.56944805 | Lower-middle | 516.8804361 | High | 2 | 4 | Stomach cancer-dominant |
| Somalia | 410.5837764 | High | 311.720451 | High | 4 | 4 | Consistent |
| South Africa | 279.7925109 | High | 151.3005349 | Lower-middle | 4 | 2 | Esophageal cancer-dominant |
| South Sudan | 411.7949464 | High | 240.7119886 | Upper-middle | 4 | 3 | Esophageal cancer-dominant |
| Spain | 60.23740885 | Lower-middle | 134.3653395 | Low | 2 | 1 | Esophageal cancer-dominant |
| Sri Lanka | 101.7453379 | Upper-middle | 88.06686371 | Low | 3 | 1 | Esophageal cancer-dominant |
| Sudan | 108.3482956 | Upper-middle | 368.3919606 | High | 3 | 4 | Stomach cancer-dominant |
| Suriname | 44.11870037 | Low | 174.1777406 | Lower-middle | 1 | 2 | Stomach cancer-dominant |
| Sweden | 56.47912946 | Lower-middle | 68.21397053 | Low | 2 | 1 | Esophageal cancer-dominant |
| Switzerland | 64.23647405 | Lower-middle | 79.7901347 | Low | 2 | 1 | Esophageal cancer-dominant |
| Syrian Arab Republic | 20.90095372 | Low | 137.0630044 | Lower-middle | 1 | 2 | Stomach cancer-dominant |
| Taiwan (Province of China) | 219.5508 | High | 193.6955042 | Lower-middle | 4 | 2 | Esophageal cancer-dominant |
| Tajikistan | 153.524514 | High | 368.198558 | High | 4 | 4 | Consistent |
| Thailand | 124.8391996 | Upper-middle | 197.2305052 | Lower-middle | 3 | 2 | Esophageal cancer-dominant |
| Timor-Leste | 54.95278464 | Lower-middle | 185.9269676 | Lower-middle | 2 | 2 | Consistent |
| Togo | 157.0544301 | High | 278.3290956 | High | 4 | 4 | Consistent |
| Tokelau | 45.86581574 | Low | 270.1542366 | Upper-middle | 1 | 3 | Stomach cancer-dominant |
| Tonga | 49.66252447 | Low | 403.786466 | High | 1 | 4 | Stomach cancer-dominant |
| Trinidad and Tobago | 51.78787322 | Lower-middle | 127.3890314 | Low | 2 | 1 | Esophageal cancer-dominant |
| Tunisia | 15.82815325 | Low | 100.654661 | Low | 1 | 1 | Consistent |
| Turkmenistan | 223.5482348 | High | 260.4413231 | Upper-middle | 4 | 3 | Esophageal cancer-dominant |
| Tuvalu | 55.90261308 | Lower-middle | 380.5986951 | High | 2 | 4 | Stomach cancer-dominant |
| Uganda | 395.9094516 | High | 156.6363293 | Lower-middle | 4 | 2 | Esophageal cancer-dominant |
| Ukraine | 66.15080866 | Lower-middle | 281.1960993 | High | 2 | 4 | Stomach cancer-dominant |
| United Arab Emirates | 46.25840141 | Low | 158.3697588 | Lower-middle | 1 | 2 | Stomach cancer-dominant |
| United Kingdom | 154.5739037 | High | 93.07637255 | Low | 4 | 1 | Esophageal cancer-dominant |
| United Republic of Tanzania | 300.1422945 | High | 150.7374159 | Lower-middle | 4 | 2 | Esophageal cancer-dominant |
| United States of America | 86.55099319 | Upper-middle | 69.16187222 | Low | 3 | 1 | Esophageal cancer-dominant |
| United States Virgin Islands | 61.04040253 | Lower-middle | 133.1801931 | Low | 2 | 1 | Esophageal cancer-dominant |
| Uruguay | 126.6809729 | Upper-middle | 248.2411195 | Upper-middle | 3 | 3 | Consistent |
| Uzbekistan | 98.60260367 | Upper-middle | 207.26281 | Upper-middle | 3 | 3 | Consistent |
| Vanuatu | 59.08560505 | Lower-middle | 420.5783397 | High | 2 | 4 | Stomach cancer-dominant |
| Venezuela (Bolivarian Republic of) | 41.29068786 | Low | 266.0107922 | Upper-middle | 1 | 3 | Stomach cancer-dominant |
| Viet Nam | 60.59055963 | Lower-middle | 196.8025815 | Lower-middle | 2 | 2 | Consistent |
| Yemen | 98.88752475 | Upper-middle | 464.5770696 | High | 3 | 4 | Stomach cancer-dominant |
| Zambia | 436.3018508 | High | 189.5760189 | Lower-middle | 4 | 2 | Esophageal cancer-dominant |
| Zimbabwe | 435.1497948 | High | 348.5203066 | High | 4 | 4 | Consistent |

Supplementary Table S7. ASR predicted by BAPC for global Esophageal and Stomach cancer from 1990 to 2031 (per 100,000)

| **Location** | **Year** | **Esophageal cancer** | | | | | | | | | **Stomach cancer** | | | | | | | |  |
| --- | --- | --- | --- | --- | --- | --- | --- | --- | --- | --- | --- | --- | --- | --- | --- | --- | --- | --- | --- |
|  |  | **Incidence** | | | **Mortality** | | | **DALYs** | | | **Incidence** | | | **Mortality** | | | **DALYs** | | |
|  |  | **Male** | **Female** | **Both** | **Male** | **Female** | **Both** | **Male** | **Female** | **Both** | **Male** | **Female** | **Both** | **Male** | **Female** | **Both** | **Male** | **Female** | **Both** |
| **Global** | 2021 | 10.63 | 3.2 | 6.65 | 10.08 | 2.99 | 6.25 | 237.75 | 67.79 | 148.56 | 20.94 | 8.63 | 14.33 | 16.03 | 7.13 | 11.20 | 371.24 | 165.57 | 262.75 |
|  | 2022 | 10.61 | 3.19 | 6.65 | 10.03 | 2.97 | 6.23 | 236.57 | 67.39 | 147.61 | 20.62 | 8.60 | 14.11 | 15.66 | 7.09 | 10.98 | 359.87 | 165.13 | 256.01 |
|  | 2023 | 10.53 | 3.17 | 6.6 | 9.92 | 2.93 | 6.16 | 234.68 | 66.66 | 145.91 | 20.16 | 8.52 | 13.78 | 15.20 | 7.02 | 10.65 | 347.10 | 164.69 | 247.21 |
|  | 2024 | 10.45 | 3.13 | 6.54 | 9.82 | 2.88 | 6.09 | 232.31 | 65.67 | 143.67 | 19.67 | 8.42 | 13.40 | 14.74 | 6.94 | 10.27 | 334.32 | 164.25 | 237.86 |
|  | 2025 | 10.37 | 3.09 | 6.48 | 9.71 | 2.83 | 6.01 | 229.59 | 64.50 | 141.13 | 19.21 | 8.33 | 13.03 | 14.28 | 6.87 | 9.89 | 321.55 | 163.81 | 228.37 |
|  | 2026 | 10.29 | 3.05 | 6.42 | 9.60 | 2.77 | 5.92 | 226.65 | 63.19 | 138.47 | 18.78 | 8.24 | 12.68 | 13.81 | 6.80 | 9.52 | 308.77 | 163.37 | 218.84 |
|  | 2027 | 10.21 | 3 | 6.35 | 9.50 | 2.71 | 5.84 | 223.56 | 61.78 | 135.77 | 18.36 | 8.14 | 12.35 | 13.35 | 6.72 | 9.17 | 296.00 | 162.93 | 209.29 |
|  | 2028 | 10.13 | 2.95 | 6.28 | 9.39 | 2.65 | 5.76 | 220.35 | 60.29 | 133.07 | 17.93 | 8.05 | 12.03 | 12.89 | 6.65 | 8.83 | 283.22 | 162.49 | 199.75 |
|  | 2029 | 10.05 | 2.89 | 6.22 | 9.28 | 2.58 | 5.67 | 217.07 | 58.73 | 130.38 | 17.50 | 7.96 | 11.70 | 12.43 | 6.57 | 8.48 | 270.45 | 162.05 | 190.20 |
|  | 2030 | 9.97 | 2.84 | 6.15 | 9.18 | 2.52 | 5.59 | 213.74 | 57.14 | 127.70 | 17.06 | 7.87 | 11.36 | 11.97 | 6.50 | 8.14 | 257.68 | 161.61 | 180.66 |
|  | 2031 | 9.89 | 2.79 | 6.08 | 9.07 | 2.45 | 5.50 | 210.38 | 55.51 | 125.04 | 16.63 | 7.77 | 11.02 | 11.51 | 6.42 | 7.79 | 244.90 | 161.17 | 171.11 |
| **High SDI** | 2021 | 8.43 | 1.90 | 4.94 | 6.96 | 1.51 | 4.02 | 161.44 | 32.36 | 93.95 | 16.25 | 6.92 | 11.16 | 9.90 | 4.37 | 6.83 | 206.69 | 93.41 | 146.10 |
|  | 2022 | 8.35 | 1.88 | 4.90 | 6.90 | 1.50 | 3.99 | 159.55 | 32.09 | 92.92 | 15.97 | 6.77 | 11.07 | 9.87 | 4.29 | 6.83 | 205.25 | 91.56 | 145.27 |
|  | 2023 | 8.27 | 1.87 | 4.85 | 6.83 | 1.48 | 3.95 | 157.66 | 31.74 | 91.88 | 15.41 | 6.62 | 10.69 | 9.85 | 4.21 | 6.70 | 203.81 | 89.71 | 141.49 |
|  | 2024 | 8.19 | 1.85 | 4.81 | 6.77 | 1.47 | 3.92 | 155.76 | 31.37 | 90.85 | 14.85 | 6.47 | 10.32 | 9.82 | 4.13 | 6.58 | 202.37 | 87.86 | 138.50 |
|  | 2025 | 8.10 | 1.83 | 4.77 | 6.71 | 1.45 | 3.89 | 153.87 | 30.98 | 89.82 | 14.29 | 6.32 | 9.94 | 9.79 | 4.06 | 6.53 | 200.93 | 86.01 | 136.45 |
|  | 2026 | 8.02 | 1.82 | 4.72 | 6.65 | 1.44 | 3.85 | 151.98 | 30.59 | 88.78 | 13.74 | 6.17 | 9.56 | 9.77 | 3.98 | 6.47 | 199.49 | 84.16 | 133.82 |
|  | 2027 | 7.94 | 1.80 | 4.68 | 6.58 | 1.42 | 3.82 | 150.09 | 30.21 | 87.75 | 13.18 | 6.02 | 9.19 | 9.74 | 3.90 | 6.37 | 198.04 | 82.31 | 130.98 |
|  | 2028 | 7.86 | 1.78 | 4.63 | 6.52 | 1.40 | 3.79 | 148.19 | 29.82 | 86.71 | 12.62 | 5.87 | 8.81 | 9.72 | 3.82 | 6.28 | 196.60 | 80.45 | 128.43 |
|  | 2029 | 7.78 | 1.77 | 4.59 | 6.46 | 1.38 | 3.75 | 146.30 | 29.43 | 85.68 | 12.07 | 5.72 | 8.43 | 9.69 | 3.74 | 6.21 | 195.16 | 78.60 | 125.88 |
|  | 2030 | 7.70 | 1.75 | 4.54 | 6.40 | 1.37 | 3.72 | 144.41 | 29.04 | 84.64 | 11.51 | 5.57 | 8.06 | 9.67 | 3.66 | 6.13 | 193.72 | 76.75 | 123.22 |
|  | 2031 | 7.62 | 1.73 | 4.50 | 6.33 | 1.35 | 3.69 | 142.52 | 28.65 | 83.61 | 10.95 | 5.42 | 7.68 | 9.64 | 3.59 | 6.05 | 192.28 | 74.90 | 120.59 |
| **High-middle  SDl** | 2021 | 15.39 | 3.38 | 8.84 | 14.37 | 3.07 | 8.13 | 340.01 | 63.32 | 192.56 | 30.25 | 10.83 | 19.62 | 22.73 | 8.72 | 14.93 | 531.44 | 198.90 | 353.18 |
|  | 2022 | 15.40 | 3.41 | 8.82 | 14.26 | 3.08 | 8.10 | 335.19 | 63.25 | 191.71 | 29.62 | 10.73 | 19.26 | 22.10 | 8.58 | 14.49 | 513.34 | 196.08 | 341.44 |
|  | 2023 | 15.40 | 3.42 | 8.74 | 14.11 | 3.08 | 8.02 | 329.97 | 61.82 | 189.60 | 29.00 | 10.46 | 18.82 | 21.37 | 8.28 | 13.96 | 492.45 | 188.72 | 326.99 |
|  | 2024 | 15.40 | 3.44 | 8.67 | 13.96 | 3.09 | 7.93 | 324.69 | 59.73 | 186.64 | 28.38 | 10.14 | 18.37 | 20.62 | 7.93 | 13.44 | 471.56 | 179.37 | 312.53 |
|  | 2025 | 15.40 | 3.45 | 8.60 | 13.81 | 3.09 | 7.83 | 319.40 | 57.64 | 183.28 | 27.76 | 9.81 | 17.93 | 19.86 | 7.57 | 12.92 | 450.67 | 170.03 | 298.07 |
|  | 2026 | 15.40 | 3.46 | 8.52 | 13.66 | 3.09 | 7.73 | 314.10 | 55.55 | 179.77 | 27.14 | 9.49 | 17.49 | 19.10 | 7.22 | 12.40 | 429.78 | 160.69 | 283.62 |
|  | 2027 | 15.40 | 3.48 | 8.45 | 13.50 | 3.09 | 7.62 | 308.81 | 53.46 | 176.25 | 26.53 | 9.16 | 17.05 | 18.34 | 6.86 | 11.88 | 408.89 | 151.34 | 269.16 |
|  | 2028 | 15.40 | 3.49 | 8.38 | 13.35 | 3.10 | 7.52 | 303.52 | 51.37 | 172.77 | 25.91 | 8.84 | 16.61 | 17.59 | 6.51 | 11.36 | 388.00 | 142.00 | 254.70 |
|  | 2029 | 15.40 | 3.49 | 8.31 | 13.20 | 3.10 | 7.41 | 298.23 | 49.28 | 169.32 | 25.29 | 8.51 | 16.17 | 16.83 | 6.15 | 10.84 | 367.11 | 132.66 | 240.25 |
|  | 2030 | 15.40 | 3.50 | 8.23 | 13.05 | 3.10 | 7.31 | 292.93 | 47.19 | 165.89 | 24.67 | 8.19 | 15.72 | 16.07 | 5.79 | 10.32 | 346.22 | 123.31 | 225.79 |
|  | 2031 | 15.40 | 3.51 | 8.16 | 12.90 | 3.10 | 7.20 | 287.64 | 45.10 | 162.47 | 24.05 | 7.86 | 15.28 | 15.31 | 5.44 | 9.80 | 325.33 | 113.97 | 211.34 |
| **Low SDI** | 2021 | 6.23 | 4.77 | 5.49 | 6.63 | 5.15 | 5.89 | 172.61 | 125.03 | 148.67 | 9.50 | 6.85 | 8.13 | 9.87 | 7.15 | 8.46 | 239.38 | 181.59 | 209.77 |
|  | 2022 | 6.20 | 4.72 | 5.46 | 6.62 | 5.10 | 5.85 | 172.43 | 124.04 | 147.81 | 9.38 | 6.76 | 8.01 | 9.74 | 7.07 | 8.33 | 235.62 | 179.17 | 206.32 |
|  | 2023 | 6.17 | 4.68 | 5.42 | 6.61 | 5.06 | 5.80 | 172.28 | 123.02 | 146.91 | 9.26 | 6.68 | 7.90 | 9.61 | 6.98 | 8.22 | 231.86 | 176.64 | 202.97 |
|  | 2024 | 6.13 | 4.64 | 5.38 | 6.60 | 5.01 | 5.76 | 172.13 | 121.96 | 145.97 | 9.13 | 6.59 | 7.79 | 9.48 | 6.90 | 8.11 | 228.11 | 174.06 | 199.66 |
|  | 2025 | 6.10 | 4.60 | 5.35 | 6.59 | 4.97 | 5.72 | 172.00 | 120.88 | 145.00 | 9.01 | 6.51 | 7.69 | 9.35 | 6.81 | 7.99 | 224.35 | 171.43 | 196.36 |
|  | 2026 | 6.07 | 4.56 | 5.31 | 6.58 | 4.92 | 5.68 | 171.89 | 119.78 | 144.01 | 8.88 | 6.43 | 7.58 | 9.22 | 6.73 | 7.88 | 220.60 | 168.79 | 193.06 |
|  | 2027 | 6.04 | 4.52 | 5.27 | 6.57 | 4.88 | 5.64 | 171.78 | 118.66 | 143.00 | 8.76 | 6.35 | 7.47 | 9.09 | 6.64 | 7.77 | 216.84 | 166.13 | 189.77 |
|  | 2028 | 6.01 | 4.48 | 5.24 | 6.56 | 4.84 | 5.60 | 171.68 | 117.52 | 141.98 | 8.64 | 6.26 | 7.37 | 8.95 | 6.55 | 7.66 | 213.08 | 163.46 | 186.47 |
|  | 2029 | 5.98 | 4.44 | 5.20 | 6.55 | 4.79 | 5.56 | 171.59 | 116.38 | 140.95 | 8.51 | 6.18 | 7.26 | 8.82 | 6.47 | 7.55 | 209.33 | 160.79 | 183.18 |
|  | 2030 | 5.95 | 4.40 | 5.16 | 6.55 | 4.75 | 5.52 | 171.51 | 115.22 | 139.92 | 8.39 | 6.10 | 7.16 | 8.69 | 6.38 | 7.44 | 205.57 | 158.11 | 179.89 |
|  | 2031 | 5.91 | 4.36 | 5.13 | 6.54 | 4.71 | 5.48 | 171.43 | 114.06 | 138.87 | 8.26 | 6.02 | 7.05 | 8.56 | 6.30 | 7.33 | 201.82 | 155.43 | 176.60 |
| **Low-middle  SDl** | 2021 | 4.48 | 2.77 | 3.59 | 4.74 | 2.93 | 3.79 | 122.13 | 73.57 | 97.10 | 9.93 | 5.64 | 7.68 | 9.92 | 5.73 | 7.71 | 245.08 | 143.95 | 192.56 |
|  | 2022 | 4.48 | 2.75 | 3.58 | 4.74 | 2.90 | 3.77 | 121.89 | 72.80 | 96.56 | 9.84 | 5.58 | 7.60 | 9.82 | 5.66 | 7.62 | 242.10 | 141.86 | 189.92 |
|  | 2023 | 4.48 | 2.72 | 3.56 | 4.74 | 2.87 | 3.76 | 121.66 | 72.03 | 96.02 | 9.76 | 5.52 | 7.53 | 9.72 | 5.59 | 7.53 | 239.11 | 139.76 | 187.28 |
|  | 2024 | 4.48 | 2.70 | 3.54 | 4.74 | 2.85 | 3.74 | 121.42 | 71.26 | 95.48 | 9.67 | 5.45 | 7.45 | 9.62 | 5.52 | 7.44 | 236.13 | 137.67 | 184.63 |
|  | 2025 | 4.48 | 2.67 | 3.53 | 4.74 | 2.82 | 3.72 | 121.19 | 70.48 | 94.94 | 9.58 | 5.39 | 7.37 | 9.53 | 5.45 | 7.36 | 233.15 | 135.57 | 181.99 |
|  | 2026 | 4.48 | 2.65 | 3.51 | 4.74 | 2.79 | 3.70 | 120.95 | 69.71 | 94.40 | 9.50 | 5.33 | 7.29 | 9.43 | 5.38 | 7.27 | 230.16 | 133.48 | 179.35 |
|  | 2027 | 4.48 | 2.62 | 3.49 | 4.74 | 2.77 | 3.68 | 120.72 | 68.94 | 93.86 | 9.41 | 5.26 | 7.21 | 9.33 | 5.31 | 7.18 | 227.18 | 131.39 | 176.71 |
|  | 2028 | 4.48 | 2.60 | 3.48 | 4.74 | 2.74 | 3.66 | 120.48 | 68.17 | 93.32 | 9.32 | 5.20 | 7.13 | 9.23 | 5.24 | 7.09 | 224.19 | 129.29 | 174.07 |
|  | 2029 | 4.48 | 2.57 | 3.46 | 4.74 | 2.71 | 3.65 | 120.24 | 67.40 | 92.78 | 9.24 | 5.14 | 7.05 | 9.13 | 5.17 | 7.00 | 221.21 | 127.20 | 171.43 |
|  | 2030 | 4.48 | 2.55 | 3.45 | 4.74 | 2.68 | 3.63 | 120.01 | 66.62 | 92.24 | 9.15 | 5.07 | 6.97 | 9.04 | 5.10 | 6.91 | 218.23 | 125.10 | 168.78 |
|  | 2031 | 4.48 | 2.52 | 3.43 | 4.74 | 2.66 | 3.61 | 119.77 | 65.85 | 91.70 | 9.06 | 5.01 | 6.89 | 8.94 | 5.03 | 6.83 | 215.24 | 123.01 | 166.14 |
| **Middle  SDI** | 2021 | 12.86 | 3.92 | 8.10 | 12.79 | 3.71 | 7.91 | 291.81 | 79.40 | 180.65 | 25.03 | 9.74 | 16.91 | 19.83 | 8.45 | 13.72 | 460.07 | 192.96 | 320.24 |
|  | 2022 | 12.72 | 3.88 | 7.99 | 12.62 | 3.65 | 7.78 | 285.90 | 77.85 | 176.59 | 24.63 | 9.59 | 16.59 | 19.33 | 8.29 | 13.34 | 444.82 | 190.42 | 309.60 |
|  | 2023 | 12.52 | 3.75 | 7.83 | 12.39 | 3.51 | 7.60 | 278.92 | 75.44 | 171.47 | 24.13 | 9.36 | 16.15 | 18.69 | 8.00 | 12.83 | 428.66 | 187.88 | 296.65 |
|  | 2024 | 12.33 | 3.61 | 7.67 | 12.17 | 3.34 | 7.41 | 271.80 | 72.47 | 165.94 | 23.63 | 9.08 | 15.70 | 18.05 | 7.64 | 12.28 | 412.50 | 185.34 | 283.71 |
|  | 2025 | 12.14 | 3.46 | 7.50 | 11.94 | 3.18 | 7.21 | 264.66 | 69.11 | 160.26 | 23.16 | 8.78 | 15.27 | 17.45 | 7.29 | 11.76 | 396.35 | 182.80 | 270.77 |
|  | 2026 | 11.94 | 3.31 | 7.32 | 11.72 | 3.01 | 7.01 | 257.51 | 65.49 | 154.52 | 22.72 | 8.48 | 14.89 | 16.89 | 6.93 | 11.30 | 380.19 | 180.25 | 257.83 |
|  | 2027 | 11.75 | 3.17 | 7.15 | 11.49 | 2.84 | 6.81 | 250.36 | 61.70 | 148.76 | 22.29 | 8.17 | 14.53 | 16.34 | 6.58 | 10.85 | 364.03 | 177.71 | 244.88 |
|  | 2028 | 11.56 | 3.02 | 6.98 | 11.26 | 2.67 | 6.61 | 243.22 | 57.80 | 142.99 | 21.84 | 7.86 | 14.15 | 15.78 | 6.22 | 10.41 | 347.88 | 175.17 | 231.94 |
|  | 2029 | 11.36 | 2.87 | 6.81 | 11.04 | 2.51 | 6.41 | 236.07 | 53.81 | 137.22 | 21.39 | 7.54 | 13.76 | 15.20 | 5.87 | 9.95 | 331.72 | 172.63 | 219.00 |
|  | 2030 | 11.17 | 2.72 | 6.63 | 10.81 | 2.34 | 6.21 | 228.93 | 49.78 | 131.45 | 20.93 | 7.23 | 13.37 | 14.62 | 5.52 | 9.47 | 315.56 | 170.09 | 206.06 |
|  | 2031 | 10.98 | 2.58 | 6.46 | 10.59 | 2.17 | 6.01 | 221.78 | 45.70 | 125.68 | 20.48 | 6.92 | 12.98 | 14.05 | 5.16 | 9.00 | 299.40 | 167.54 | 193.11 |

Supplementary Table S8. Number predicted by BAPC for global Esophageal and Stomach cancer from 1990 to 2031

| **Location** | **Year** | **Esophageal cancer** | | | | | | | | | **Stomach cancer** | | | | | | | |  |
| --- | --- | --- | --- | --- | --- | --- | --- | --- | --- | --- | --- | --- | --- | --- | --- | --- | --- | --- | --- |
|  |  | **Incidence** | | | **Mortality** | | | **DALYs** | | | **Incidence** | | | **Mortality** | | | **DALYs** | | |
|  |  | **Male** | **Female** | **Both** | **Male** | **Female** | **Both** | **Male** | **Female** | **Both** | **Male** | **Female** | **Both** | **Male** | **Female** | **Both** | **Male** | **Female** | **Both** |
| **Global** | 2021 | 428,387 | 148,142 | 576,529 | 399,796 | 138,806 | 538,602 | 9,889,701 | 3,109,564 | 12,999,265 | 832,921 | 397,312 | 1,230,233 | 624,551 | 329,822 | 954,374 | 15,274,347 | 7,512,286 | 22,786,633 |
|  | 2022 | 436,978 | 151,510 | 589,083 | 406,841 | 141,315 | 548,810 | 10,009,668 | 3,153,015 | 13,213,590 | 839,753 | 405,654 | 1,244,077 | 627,964 | 334,098 | 961,704 | 15,261,642 | 7,588,651 | 22,823,342 |
|  | 2023 | 442,930 | 154,315 | 598,464 | 411,872 | 143,223 | 556,502 | 10,103,579 | 3,166,840 | 13,381,704 | 846,345 | 411,805 | 1,252,325 | 629,979 | 334,091 | 965,095 | 15,244,164 | 7,646,329 | 22,823,342 |
|  | 2024 | 448,882 | 156,727 | 606,638 | 416,902 | 144,674 | 563,228 | 10,197,490 | 3,154,214 | 13,518,258 | 852,938 | 417,956 | 1,258,505 | 631,963 | 332,057 | 966,886 | 15,231,184 | 7,689,893 | 22,823,342 |
|  | 2025 | 454,833 | 158,862 | 614,354 | 421,933 | 145,777 | 569,583 | 10,291,401 | 3,120,180 | 13,637,730 | 859,530 | 424,107 | 1,265,237 | 634,462 | 329,291 | 968,939 | 15,221,543 | 7,722,798 | 22,823,342 |
|  | 2026 | 460,785 | 160,804 | 621,895 | 426,964 | 146,616 | 575,795 | 10,385,312 | 3,070,922 | 13,749,831 | 866,123 | 430,257 | 1,273,082 | 637,324 | 326,488 | 971,815 | 15,214,384 | 7,747,650 | 22,823,342 |
|  | 2027 | 466,737 | 162,610 | 629,370 | 431,995 | 147,254 | 581,953 | 10,479,223 | 3,013,053 | 13,859,742 | 872,716 | 436,408 | 1,281,502 | 640,232 | 323,980 | 975,232 | 15,209,066 | 7,766,422 | 22,823,342 |
|  | 2028 | 472,688 | 164,321 | 636,820 | 437,026 | 147,739 | 588,090 | 10,573,133 | 2,952,964 | 13,969,658 | 879,308 | 442,559 | 1,289,924 | 643,034 | 321,893 | 978,758 | 15,205,117 | 7,780,600 | 22,823,342 |
|  | 2029 | 478,640 | 165,966 | 644,261 | 442,057 | 148,107 | 594,219 | 10,667,044 | 2,896,296 | 14,080,208 | 885,901 | 448,710 | 1,298,146 | 645,746 | 320,238 | 982,160 | 15,202,184 | 7,791,309 | 22,823,342 |
|  | 2030 | 484,591 | 167,563 | 651,697 | 447,088 | 148,388 | 600,344 | 10,760,955 | 2,847,547 | 14,191,364 | 892,493 | 454,861 | 1,306,226 | 648,437 | 318,972 | 985,424 | 15,200,006 | 7,799,397 | 22,823,342 |
|  | 2031 | 490,543 | 169,128 | 659,133 | 452,119 | 148,601 | 606,469 | 10,854,866 | 2,809,852 | 14,302,920 | 899,086 | 461,011 | 1,314,282 | 651,149 | 318,031 | 988,626 | 15,198,388 | 7,805,506 | 22,823,342 |
| **High SDI** | 2021 | 79,924 | 22,585 | 102,510 | 66,614 | 19,038 | 85,652 | 1,474,142 | 351,318 | 1,825,459 | 155,312 | 83,807 | 239,119 | 95,941 | 57,598 | 153,539 | 1,900,402 | 997,002 | 2,897,404 |
|  | 2022 | 80,804 | 22,843 | 103,640 | 67,455 | 19,244 | 86,665 | 1,483,068 | 353,702 | 1,836,941 | 156,973 | 83,399 | 243,524 | 96,985 | 57,287 | 153,827 | 1,903,110 | 981,886 | 2,924,141 |
|  | 2023 | 81,683 | 23,100 | 104,770 | 68,296 | 19,428 | 87,679 | 1,491,994 | 356,086 | 1,848,422 | 156,973 | 82,991 | 243,524 | 97,176 | 57,374 | 154,115 | 1,882,228 | 976,585 | 2,878,251 |
|  | 2024 | 82,562 | 23,358 | 105,900 | 69,136 | 19,604 | 88,692 | 1,500,920 | 358,471 | 1,859,904 | 156,973 | 82,583 | 243,524 | 97,368 | 57,460 | 154,403 | 1,861,345 | 971,284 | 2,858,726 |
|  | 2025 | 83,441 | 23,616 | 107,031 | 69,977 | 19,779 | 89,705 | 1,509,845 | 360,855 | 1,871,385 | 156,973 | 82,174 | 243,524 | 97,559 | 57,547 | 154,690 | 1,840,462 | 965,982 | 2,867,189 |
|  | 2026 | 84,320 | 23,874 | 108,161 | 70,818 | 19,954 | 90,719 | 1,518,771 | 363,240 | 1,882,867 | 156,973 | 81,766 | 243,524 | 97,750 | 57,634 | 154,978 | 1,819,579 | 960,681 | 2,853,946 |
|  | 2027 | 85,200 | 24,131 | 109,291 | 71,659 | 20,128 | 91,732 | 1,527,697 | 365,624 | 1,894,348 | 156,973 | 81,358 | 243,524 | 97,941 | 57,720 | 155,266 | 1,798,697 | 955,380 | 2,833,466 |
|  | 2028 | 86,079 | 24,389 | 110,422 | 72,499 | 20,302 | 92,745 | 1,536,623 | 368,008 | 1,905,829 | 156,973 | 80,949 | 243,524 | 98,133 | 57,807 | 155,554 | 1,777,814 | 950,078 | 2,825,998 |
|  | 2029 | 86,958 | 24,647 | 111,552 | 73,340 | 20,476 | 93,758 | 1,545,549 | 370,393 | 1,917,311 | 156,973 | 80,541 | 243,524 | 98,324 | 57,893 | 155,842 | 1,756,931 | 944,777 | 2,818,126 |
|  | 2030 | 87,837 | 24,904 | 112,682 | 74,181 | 20,650 | 94,772 | 1,554,475 | 372,777 | 1,928,792 | 156,973 | 80,133 | 243,524 | 98,515 | 57,980 | 156,129 | 1,736,048 | 939,476 | 2,803,912 |
|  | 2031 | 88,716 | 25,162 | 113,813 | 75,021 | 20,824 | 95,785 | 1,563,401 | 375,162 | 1,940,274 | 156,973 | 79,725 | 243,524 | 98,706 | 58,066 | 156,417 | 1,715,166 | 934,174 | 2,791,849 |
| **High-middle  SDl** | 2021 | 138,880 | 37,887 | 176,768 | 127,643 | 34,788 | 162,430 | 3,139,724 | 694,567 | 3,834,291 | 269,803 | 117,393 | 387,196 | 198,606 | 96,499 | 295,105 | 4,822,268 | 2,079,339 | 6,901,607 |
|  | 2022 | 142,055 | 38,506 | 179,969 | 130,311 | 35,374 | 165,366 | 3,170,283 | 708,978 | 3,898,068 | 271,793 | 119,115 | 390,145 | 199,178 | 97,753 | 295,553 | 4,819,823 | 2,111,108 | 6,886,280 |
|  | 2023 | 144,777 | 38,686 | 182,088 | 132,571 | 35,698 | 167,508 | 3,195,372 | 719,996 | 3,947,266 | 273,566 | 119,878 | 391,931 | 199,101 | 98,412 | 295,553 | 4,819,823 | 2,130,994 | 6,844,150 |
|  | 2024 | 147,210 | 38,452 | 184,207 | 134,571 | 35,769 | 169,335 | 3,220,462 | 728,420 | 3,985,216 | 275,340 | 120,216 | 393,717 | 198,627 | 98,759 | 295,553 | 4,819,823 | 2,143,443 | 6,802,020 |
|  | 2025 | 149,458 | 37,860 | 186,327 | 136,405 | 35,619 | 171,037 | 3,245,551 | 734,860 | 4,014,491 | 277,113 | 120,365 | 395,503 | 198,002 | 98,942 | 295,553 | 4,819,823 | 2,151,235 | 6,759,891 |
|  | 2026 | 151,587 | 36,993 | 188,446 | 138,132 | 35,295 | 172,690 | 3,270,640 | 739,783 | 4,037,073 | 278,886 | 120,432 | 397,289 | 197,411 | 99,038 | 295,553 | 4,819,823 | 2,156,114 | 6,717,761 |
|  | 2027 | 153,641 | 35,947 | 190,565 | 139,791 | 34,851 | 174,323 | 3,295,729 | 743,547 | 4,054,492 | 280,660 | 120,461 | 399,075 | 196,966 | 99,089 | 295,553 | 4,819,823 | 2,159,167 | 6,675,631 |
|  | 2028 | 155,646 | 34,827 | 192,684 | 141,406 | 34,347 | 175,948 | 3,320,818 | 746,425 | 4,067,929 | 282,433 | 120,474 | 400,860 | 196,708 | 99,115 | 295,553 | 4,819,823 | 2,161,079 | 6,633,502 |
|  | 2029 | 157,621 | 33,733 | 194,804 | 142,994 | 33,837 | 177,570 | 3,345,908 | 748,625 | 4,078,294 | 284,206 | 120,480 | 402,646 | 196,623 | 99,129 | 295,553 | 4,819,823 | 2,162,275 | 6,591,372 |
|  | 2030 | 159,576 | 32,754 | 196,923 | 144,563 | 33,367 | 179,190 | 3,370,997 | 750,307 | 4,086,290 | 285,980 | 120,482 | 404,432 | 196,666 | 99,136 | 295,553 | 4,819,823 | 2,163,024 | 6,549,242 |
|  | 2031 | 161,518 | 31,961 | 199,042 | 146,122 | 32,975 | 180,810 | 3,396,086 | 751,593 | 4,092,458 | 287,753 | 120,483 | 406,218 | 196,781 | 99,140 | 295,553 | 4,819,823 | 2,163,493 | 6,507,112 |
| **Low SDI** | 2021 | 15,896 | 12,064 | 27,960 | 16,360 | 12,564 | 28,924 | 481,370 | 348,752 | 830,121 | 23,213 | 18,176 | 41,388 | 23,024 | 18,168 | 41,192 | 653,460 | 545,443 | 1,198,903 |
|  | 2022 | 16,206 | 12,316 | 28,523 | 16,653 | 12,808 | 29,462 | 492,310 | 356,853 | 849,162 | 23,521 | 18,494 | 41,989 | 23,268 | 18,433 | 41,711 | 662,425 | 555,775 | 1,217,459 |
|  | 2023 | 16,517 | 12,568 | 29,085 | 16,947 | 13,053 | 30,000 | 503,250 | 364,953 | 868,203 | 23,829 | 18,812 | 42,590 | 23,512 | 18,697 | 42,231 | 671,390 | 565,996 | 1,236,014 |
|  | 2024 | 16,828 | 12,820 | 29,648 | 17,241 | 13,297 | 30,538 | 514,190 | 373,054 | 887,244 | 24,137 | 19,130 | 43,190 | 23,756 | 18,962 | 42,750 | 680,355 | 576,255 | 1,254,570 |
|  | 2025 | 17,139 | 13,071 | 30,210 | 17,534 | 13,541 | 31,075 | 525,131 | 381,155 | 906,286 | 24,445 | 19,448 | 43,791 | 24,000 | 19,227 | 43,270 | 689,320 | 586,501 | 1,273,125 |
|  | 2026 | 17,450 | 13,323 | 30,773 | 17,828 | 13,786 | 31,613 | 536,071 | 389,256 | 925,327 | 24,754 | 19,766 | 44,391 | 24,244 | 19,491 | 43,789 | 698,285 | 596,752 | 1,291,681 |
|  | 2027 | 17,761 | 13,575 | 31,335 | 18,121 | 14,030 | 32,151 | 547,011 | 397,356 | 944,368 | 25,062 | 20,084 | 44,992 | 24,489 | 19,756 | 44,309 | 707,249 | 607,001 | 1,310,237 |
|  | 2028 | 18,072 | 13,826 | 31,898 | 18,415 | 14,274 | 32,689 | 557,951 | 405,457 | 963,409 | 25,370 | 20,402 | 45,593 | 24,733 | 20,021 | 44,828 | 716,214 | 617,250 | 1,328,792 |
|  | 2029 | 18,382 | 14,078 | 32,460 | 18,708 | 14,519 | 33,227 | 568,892 | 413,558 | 982,450 | 25,678 | 20,720 | 46,193 | 24,977 | 20,285 | 45,348 | 725,179 | 627,500 | 1,347,348 |
|  | 2030 | 18,693 | 14,330 | 33,023 | 19,002 | 14,763 | 33,765 | 579,832 | 421,659 | 1,001,491 | 25,986 | 21,038 | 46,794 | 25,221 | 20,550 | 45,867 | 734,144 | 637,749 | 1,365,903 |
|  | 2031 | 19,004 | 14,581 | 33,585 | 19,295 | 15,008 | 34,303 | 590,772 | 429,760 | 1,020,532 | 26,294 | 21,356 | 47,394 | 25,465 | 20,814 | 46,387 | 743,109 | 647,998 | 1,384,459 |
| **Low-middle  SDl** | 2021 | 31,385 | 20,719 | 52,104 | 32,307 | 21,417 | 53,724 | 911,822 | 579,811 | 1,491,634 | 68,245 | 42,151 | 110,396 | 65,856 | 41,600 | 107,456 | 1,806,852 | 1,146,274 | 2,953,125 |
|  | 2022 | 31,955 | 20,940 | 52,869 | 32,891 | 21,680 | 54,444 | 927,616 | 586,728 | 1,514,098 | 68,918 | 42,756 | 111,922 | 66,714 | 42,023 | 108,903 | 1,827,783 | 1,160,127 | 2,987,910 |
|  | 2023 | 32,524 | 21,151 | 53,580 | 33,475 | 21,982 | 55,110 | 942,536 | 593,758 | 1,534,655 | 69,830 | 43,360 | 113,448 | 67,571 | 42,555 | 110,350 | 1,848,713 | 1,173,981 | 3,022,694 |
|  | 2024 | 33,093 | 21,366 | 54,312 | 34,059 | 22,299 | 55,796 | 957,753 | 601,304 | 1,556,116 | 70,741 | 43,964 | 114,975 | 68,428 | 43,119 | 111,797 | 1,869,644 | 1,187,835 | 3,057,479 |
|  | 2025 | 33,663 | 21,579 | 55,036 | 34,643 | 22,623 | 56,475 | 972,868 | 608,999 | 1,577,149 | 71,653 | 44,568 | 116,501 | 69,286 | 43,693 | 113,244 | 1,890,575 | 1,201,689 | 3,092,263 |
|  | 2026 | 34,232 | 21,794 | 55,763 | 35,227 | 22,949 | 57,156 | 988,019 | 616,878 | 1,598,385 | 72,564 | 45,173 | 118,027 | 70,143 | 44,270 | 114,691 | 1,911,505 | 1,215,542 | 3,127,048 |
|  | 2027 | 34,802 | 22,007 | 56,488 | 35,811 | 23,277 | 57,837 | 1,003,157 | 624,842 | 1,619,525 | 73,476 | 45,777 | 119,553 | 71,001 | 44,849 | 116,138 | 1,932,436 | 1,229,396 | 3,161,832 |
|  | 2028 | 35,371 | 22,221 | 57,215 | 36,395 | 23,605 | 58,517 | 1,018,300 | 632,878 | 1,640,710 | 74,388 | 46,381 | 121,079 | 71,858 | 45,427 | 117,585 | 1,953,367 | 1,243,250 | 3,196,617 |
|  | 2029 | 35,940 | 22,435 | 57,941 | 36,979 | 23,934 | 59,198 | 1,033,441 | 640,955 | 1,661,873 | 75,299 | 46,985 | 122,605 | 72,715 | 46,006 | 119,032 | 1,974,298 | 1,257,104 | 3,231,402 |
|  | 2030 | 36,510 | 22,649 | 58,667 | 37,563 | 24,262 | 59,879 | 1,048,582 | 649,062 | 1,683,047 | 76,211 | 47,590 | 124,131 | 73,573 | 46,585 | 120,479 | 1,995,228 | 1,270,958 | 3,266,186 |
|  | 2031 | 37,079 | 22,863 | 59,393 | 38,147 | 24,591 | 60,559 | 1,063,724 | 657,188 | 1,704,216 | 77,122 | 48,194 | 125,657 | 74,430 | 47,164 | 121,926 | 2,016,159 | 1,284,811 | 3,300,971 |
| **Middle  SDI** | 2021 | 162,124 | 54,827 | 216,951 | 156,695 | 50,938 | 207,634 | 3,878,041 | 1,133,742 | 5,011,783 | 315,930 | 135,535 | 451,465 | 240,743 | 115,717 | 356,459 | 6,081,976 | 2,738,741 | 8,820,717 |
|  | 2022 | 164,386 | 55,614 | 220,330 | 159,192 | 51,568 | 211,679 | 3,932,970 | 1,152,546 | 5,073,853 | 319,601 | 138,179 | 456,768 | 241,715 | 103,783 | 358,225 | 6,083,777 | 2,767,944 | 8,824,253 |
|  | 2023 | 166,472 | 55,543 | 223,202 | 160,624 | 51,498 | 214,920 | 3,974,297 | 1,165,563 | 5,108,928 | 323,339 | 140,078 | 461,523 | 243,544 | 103,783 | 360,531 | 6,083,777 | 2,782,933 | 8,759,477 |
|  | 2024 | 168,557 | 54,815 | 225,854 | 161,798 | 50,926 | 217,919 | 4,010,654 | 1,174,574 | 5,128,749 | 327,076 | 141,586 | 466,278 | 245,373 | 103,783 | 362,837 | 6,083,777 | 2,787,884 | 8,679,353 |
|  | 2025 | 170,643 | 53,554 | 228,411 | 163,208 | 50,062 | 220,833 | 4,045,196 | 1,180,813 | 5,139,950 | 330,813 | 142,890 | 471,033 | 247,201 | 103,783 | 365,144 | 6,083,777 | 2,787,730 | 8,608,796 |
|  | 2026 | 172,728 | 51,926 | 230,927 | 164,943 | 49,096 | 223,578 | 4,079,074 | 1,185,132 | 5,146,279 | 334,551 | 144,087 | 475,788 | 249,030 | 103,783 | 367,450 | 6,083,777 | 2,785,921 | 8,556,198 |
|  | 2027 | 174,814 | 50,114 | 233,425 | 166,870 | 48,178 | 226,119 | 4,112,710 | 1,188,122 | 5,149,856 | 338,288 | 145,227 | 480,543 | 250,858 | 103,783 | 369,756 | 6,083,777 | 2,784,193 | 8,521,458 |
|  | 2028 | 176,899 | 48,305 | 235,915 | 168,829 | 47,413 | 228,526 | 4,146,258 | 1,190,192 | 5,151,877 | 342,025 | 146,338 | 485,298 | 252,687 | 103,783 | 372,062 | 6,083,777 | 2,783,102 | 8,500,988 |
|  | 2029 | 178,984 | 46,666 | 238,402 | 170,731 | 46,856 | 230,880 | 4,179,773 | 1,191,624 | 5,153,020 | 345,763 | 147,434 | 490,053 | 254,516 | 103,783 | 374,369 | 6,083,777 | 2,782,608 | 8,490,475 |
|  | 2030 | 181,070 | 45,335 | 240,887 | 172,569 | 46,524 | 233,212 | 4,213,276 | 1,192,616 | 5,153,665 | 349,500 | 148,522 | 494,808 | 256,344 | 103,783 | 376,675 | 6,083,777 | 2,782,487 | 8,486,160 |
|  | 2031 | 183,155 | 44,409 | 243,372 | 174,372 | 46,396 | 235,516 | 4,246,775 | 1,193,303 | 5,154,030 | 353,237 | 149,606 | 499,563 | 258,173 | 103,783 | 378,981 | 6,083,777 | 2,782,535 | 8,485,262 |
